# Mucosal associated Lymphoid Tissue Lymphoma of the uvea: an analysis of 3 cases

**DOI:** 10.1186/s12886-022-02598-2

**Published:** 2022-09-19

**Authors:** Zhenyu Wang, Yueming Liu, Jing Mo, Xusheng Cao, Xiaolin Xu, Lin Shen, Hong Wang, Wenbin Wei

**Affiliations:** Beijing Ophthalmology & Visual Science Key Lab, Beijing Tongren Eye Center, Beijing Tongren Hospital, Capital Medical University, No.1 Dong Jiao Min Xiang, Dongcheng District, Beijing, 100730 China

**Keywords:** MALT lymphoma, Uvea, Posterior scleritis

## Abstract

**Background:**

Ocular mucosa-associated lymphoid tissue (MALT) lymphoma involving orbit and conjunctiva usually has an indolent clinical course with “salmon patch” mass as typical presentation. This study is to report a series of rare cases and investigate the clinical and pathological features of ocular MALT lymphoma that involved uveal tissue primarily and presented as posterior scleritis.

**Methods:**

This retrospective, observational study was conducted at Beijing Tongren Hospital. From 2018 to 2020, 3 cases of 3 eyes (2 female patients and 1 male patient) with ocular MALT lymphoma that involved uveal tissue primarily and presented as posterior scleritis were included in the study. All patients had complaints of red eyes with blurred vision. The average age was 56.33 ± 2.08 years old and the average time from initial diagnosis to pathological diagnosis was 3.00 ± 1.73 months. Ophthalmic examinations including best-corrected visual acuity (BCVA), intraocular pressure (IOP), slit lamp microscope examinations, fundus photography, B-scan ultrasonography, ultrasound biomicroscope (UBM), optical coherence tomography (OCT), fundus fluorescein angiography (FFA) and indocyanine green angiography (ICGA) were conducted. Systemic workups including orbital magnetic resonance imaging (MRI), positron emission tomography-computed tomography (PET-CT) and blood autoimmune antibody tests were also conducted. Pathological tissue from patients were obtained through surgeries. Biopsy examinations were performed to accurately determine pathological diagnosis. All the information of clinical, imaging and pathological changes were collected and analyzed.

**Results:**

At the initial diagnosis, the BCVA of involved eyes decreased seriously while the IOP were normal. All involved eyeball showed extensive hyperemia and local thickening in the wall of eyeballs. B-scan ultrasonography showed mass with abundant blood and irregular cysts inside the eyeball wall and in the retrobulbar orbit, surrounding the ocular wall and optic nerve. UBM showed solid lesions with low and medium echo under the conjunctiva and inside the ciliary body of 2 cases. OCT showed posterior polar wavy rise of RPE and local neuroepithelial detachment in all cases. FFA and ICGA showed vascular abnormalities (patch-like strong fluorescence and fluorescence leakage) and local thickening in retina and choroid (Rectangle-like weak fluorescence below the macula). The posterior wall of the eyeball was thickened and enhanced in MRI. PET-CT also showed thickening of posterior wall of eyeballs and increased metabolic activity but there was no sign of autoimmune disease. All patients were diagnosed as MALT lymphoma through pathologic examinations of biopsy tissue.

**Conclusions:**

The onset of primary ocular MALT lymphoma in uvea is hidden. The early clinical manifestations are lack of specificity and misleading. B-scan ultrasonography has characteristic manifestations and is valuable in diagnosis. However, pathological diagnosis through tissue biopsy is irreplaceable.

**Supplementary Information:**

The online version contains supplementary material available at 10.1186/s12886-022-02598-2.

## Background

Lymphoma is a group of lymphoproliferative malignancies of B- or T-lymphocytes with multiple subtypes and varying clinical features. It can occur in lymph nodes or spleen, or in other sites outside the lymph nodes (extranodal). Among all the extranodal presentations, mucosa-associated lymphoid tissue (MALT) lymphoma is the most common type. Even in ocular presented lymphoma, a rare manifestation which accounts for 5% of all extranodal lymphoma, MALT lymphoma accounts for 80 to 85% [1, 2].

According to the WHO classification, MALT lymphoma is defined as an extranodal lymphoma composed of morphologically heterogeneous small B-cells including marginal zone cells, cells resembling monocitoid cells, small lymphocytes, scattered immunoblast cells and centroblast-like cells [3, 4]. MALT lymphoma was first described and characterized by the presence of small B-lymphocytes of low-grade malignancy by Issac and Wright in 1983 [4]. According to several studies, female patients in their 50 s to 70 s are easier to be affected while young adult and children are hardly [5–7]. MALT lymphoma can arise in a variety of sites. To the best of our knowledge, it mainly involves extranodal tissue of gastrointestinal tract, salivary gland, thyroid, lung, lacrimal gland, orbit, and conjunctiva [3, 8–10]. Among them, ocular MALT lymphoma usually involves ocular adnexa primarily and was defined as the MALT type of primary ocular adnexal lymphoma (POAML). It generally develops in females after the fourth decades of life [11]. Except for the "salmon patch" symptom related to conjunctival involvement, most of the other clinical features are related to orbital invasion, including exophthalmia (27%), palpable mass (19%), decreased visual acuity and ptosis (6%) and diplopia (2%) [11]. POAML has been reported to account for 35 to 90% of ocular lymphoma [12–15].

However, in a very few cases, it can also originate in the site of uveal tissue primarily and the clinical manifestations can be diverse and difficult to diagnose. Unfortunately, it is often misdiagnosed as uveitis, scleritis and choroidal hemangioma. Up till now, there is still no sufficient relevant research.

Here we report a series of rare cases of ocular MALT lymphoma that involved uveal tissue primarily and presented as posterior scleritis. The clinical and imaging features of the 3 patients were analyzed retrospectively.

## Methods

This retrospective, observational study was conducted at Beijing Tongren Hospital. All patients have consented for their anonymized data to be published.

From 2018 to 2020, 3 cases of 3 eyes (3 patients) with ocular MALT lymphoma that involved uveal tissue primarily and presented as posterior scleritis were included in the study. All patients were diagnosed by pathological examination in Beijing Tongren Hospital. The basic information was shown in Table 1. The mean age of the patients was 56.33 ± 2.08 years old. The average time from initial diagnosis to pathological diagnosis was 3.00 ± 1.73 months.

**Table 1:** The basic information of involved patients

Patient No	Gender	Age (years)	Left/ Right Eyes	Time from initial diagnosis to pathological diagnosis (months)
1	Female	57	Left	2
2	Male	58	Right	2
3	Female	54	Right	5

All patients had complaints of red eyes with blurred vision, which were previously diagnosed as posterior scleritis and were treated with oral glucocorticoids. Unfortunately, the patient's symptoms were not relieved. All patients declared no previous history of systemic lymphoma. Primary lymphoma was excluded by systemic examination. Several ophthalmic examinations were conducted including best-corrected visual acuity (BCVA) using Snellen chart, intraocular pressure (IOP), slit lamp microscope examinations and fundus photography. B-scan ultrasonography and ultrasound biomicroscope (UBM, conducted in 2 cases) examinations were conducted to reveal the extent, shape and internal blood flow of the lesion. Optical coherence tomography (OCT), fundus fluorescein angiography (FFA) and indocyanine green angiography (ICGA, conducted in 2 cases) examinations were conducted to detect the damage of the lesion to the retinal and choroidal structures and blood vessels. Systemic workups, including orbital magnetic resonance imaging (MRI), positron emission tomography-computed tomography (PET-CT, conducted in 2 cases) and blood autoimmune antibody tests were also conducted to exclude systemic autoimmune diseases and neoplasm. In order to accurately determine pathological diagnosis, the clinicians and oncologists conducted diagnostic surgeries. Thickened pathological conjunctiva and abnormal tissue beneath it were removed and obtained from patients. Biopsy and immunohistochemistry tests were performed to provide pathological diagnosis. All the information of clinical, imaging and pathological changes were collected and analyzed.

## Results

**Fig. 1:**
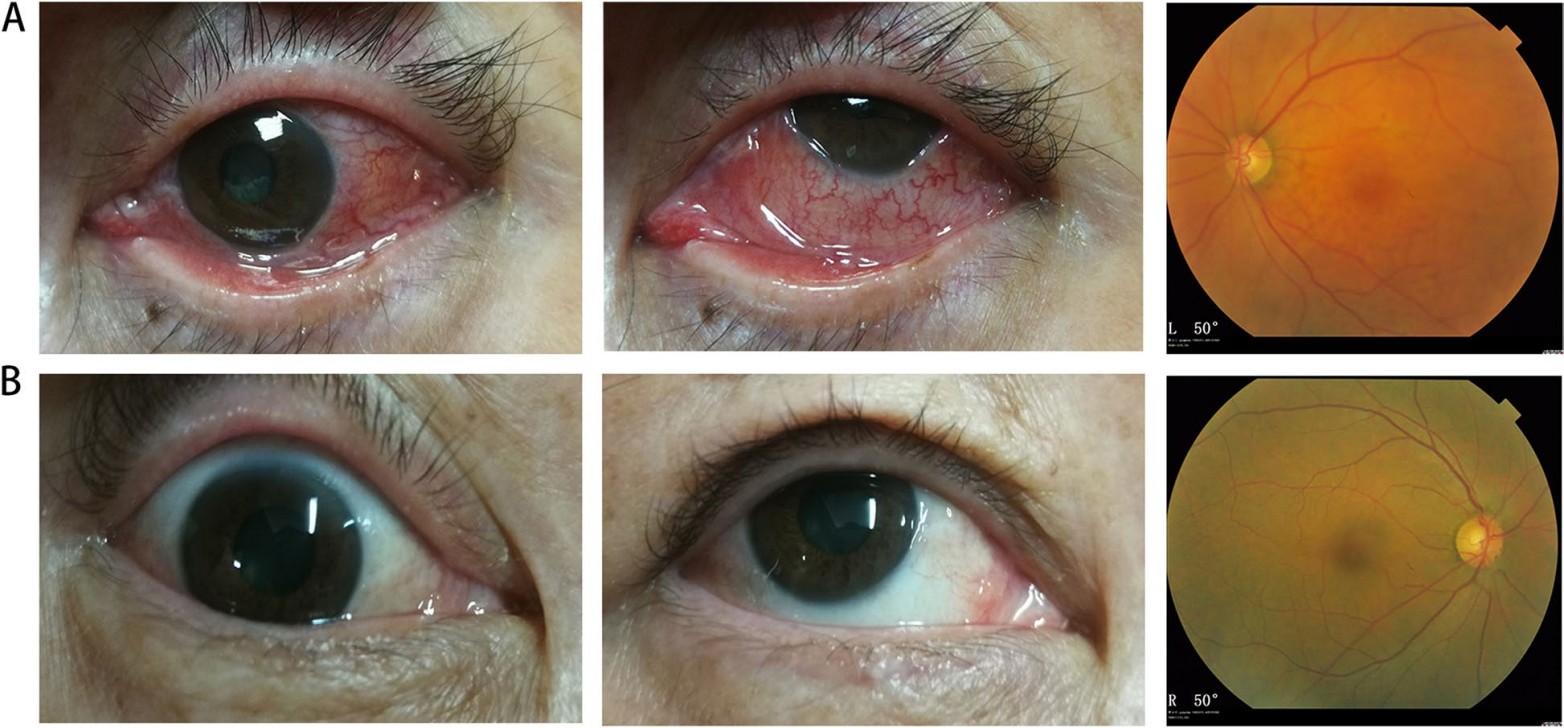
The photographs of the patients’ eyes. **A** The photographs that show hyperemia in the whole wall of the involved eyeballs. **B** The photographs of their normal eyes

The initial ophthalmic examinations revealed that the BCVA of their involved eyes were 20/63, HM/20 cm and 20/200 (Snellen chart). The IOP were 16 mmHg, 14 mmHg and 16 mmHg. The slit-lamp examination and dilated fundus of all these 3 eyes was unmarkable except for extensive hyperemia in the whole wall and local thickening of their involved eyeball compared with their normal eye (Fig. 1). There was no sign of inflammation in the anterior chamber.

**Fig. 2:**
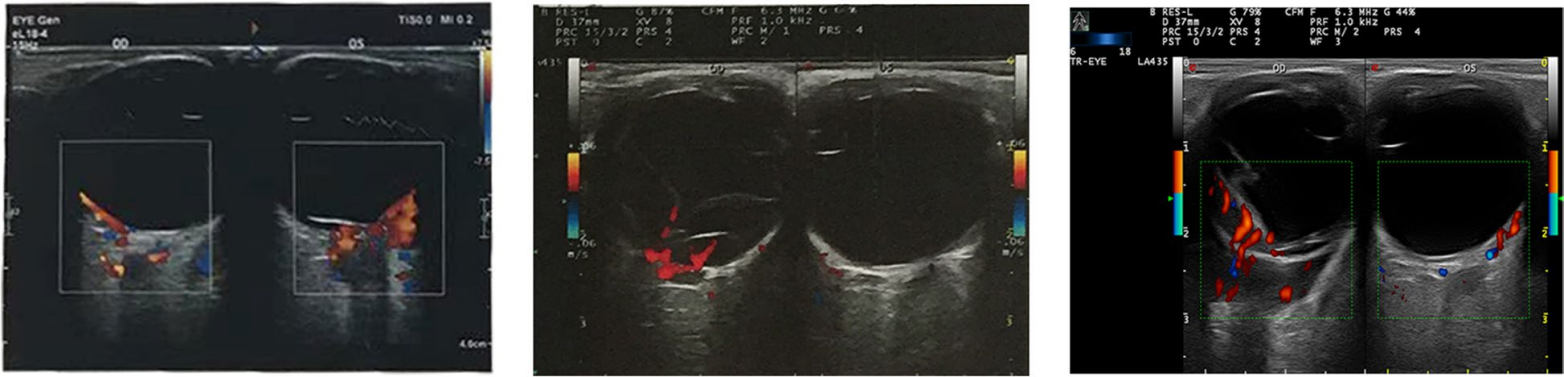
The B-scan ultrasonography results of all the patients’ involved eyes. All the results showed mass and irregular cysts inside and around the eyeball wall and optic nerve. The color doppler flow imaging (CDFI) showed abundant blood flow inside the mass

**Fig. 3:**
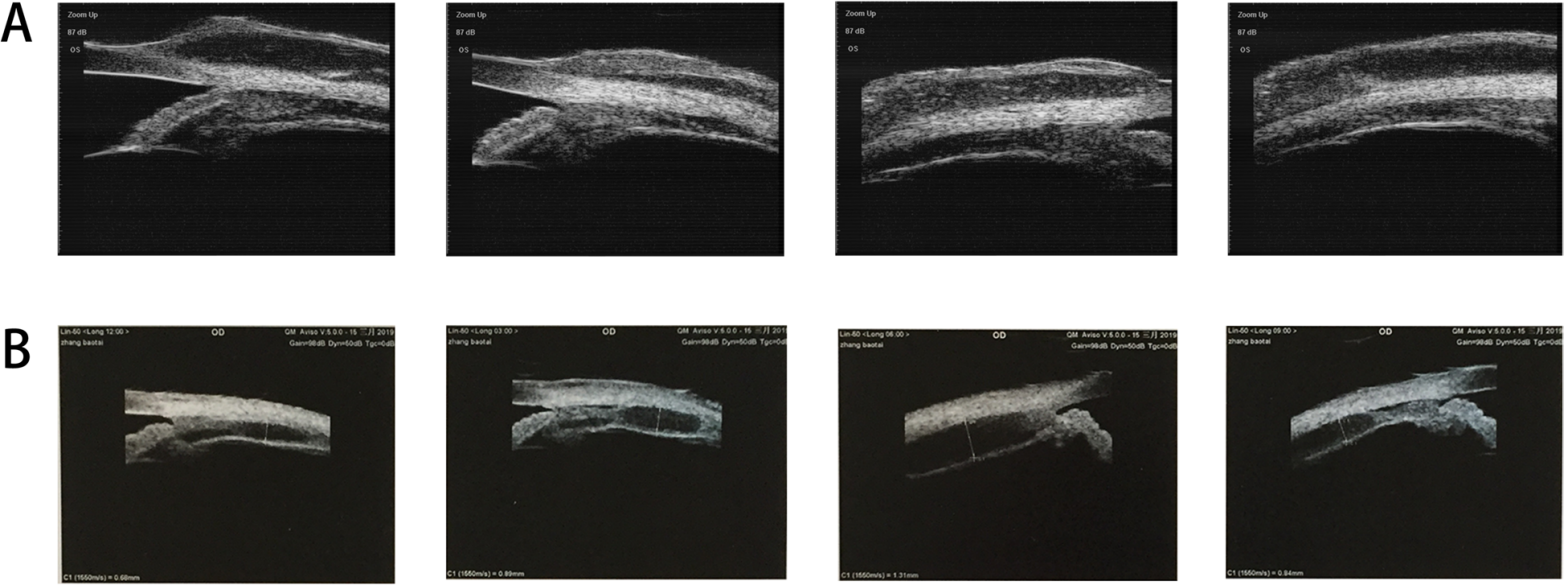
The UBM results of the patients’ involved eyes. **A** UBM showed solid lesions with low and medium echo under the conjunctiva (The thickest part was about 1.25 mm). The internal echo was not uniform, and the boundary between the lesion and sclera was still clear. **B** UBM showed solid lesions with low and medium echo inside the ciliary body. The internal echo was uniform and the boundary with sclera was clear

**Fig. 4:**
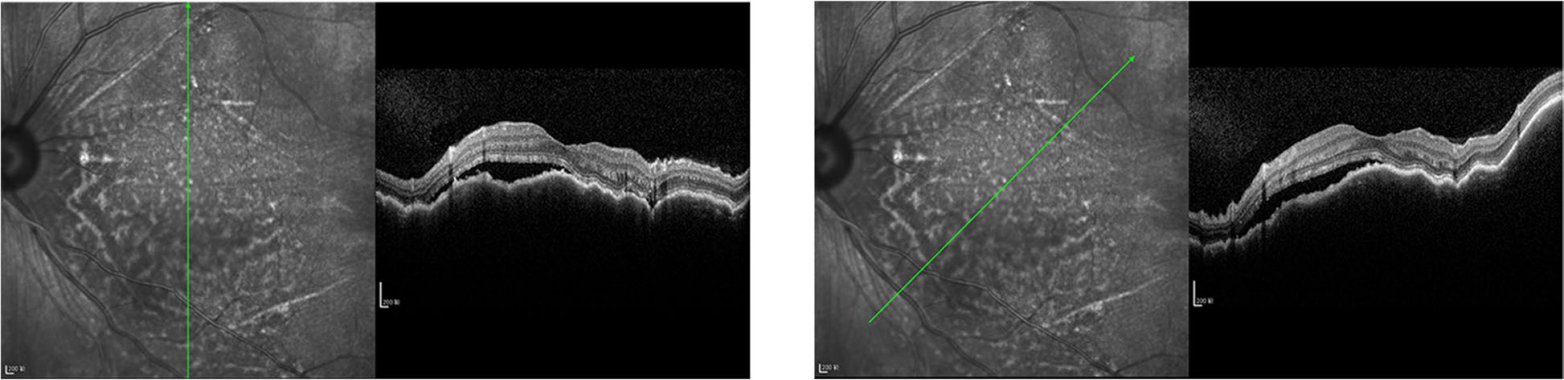
The OCT results of the patients’ eyes showing posterior polar wavy rise of RPE and local neuroepithelial detachment

**Fig. 5:**
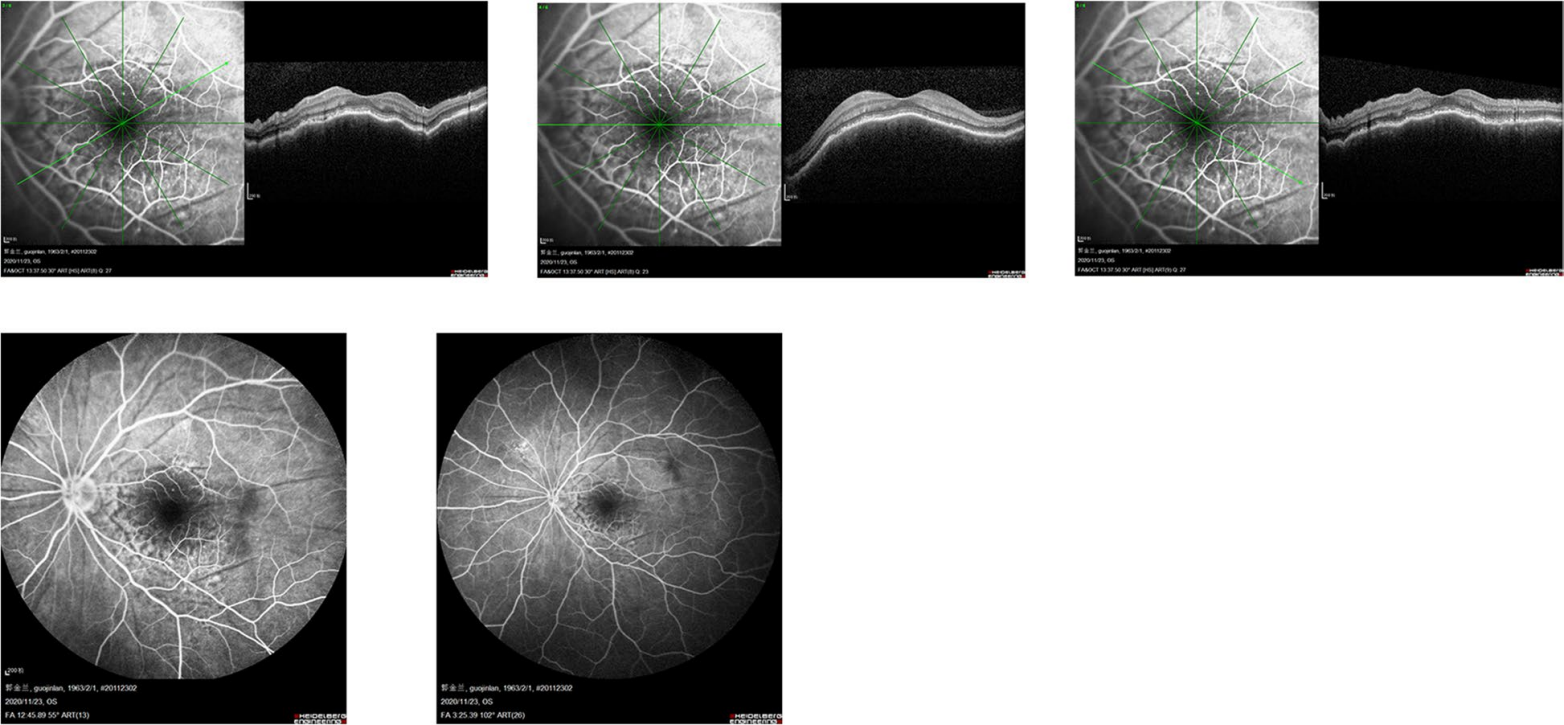
The FFA results of the patients’ eyes showing local thickening and patch-like strong fluorescence around the optic disc, among which there was radial weak fluorescence (venous phase)

**Fig. 6:**
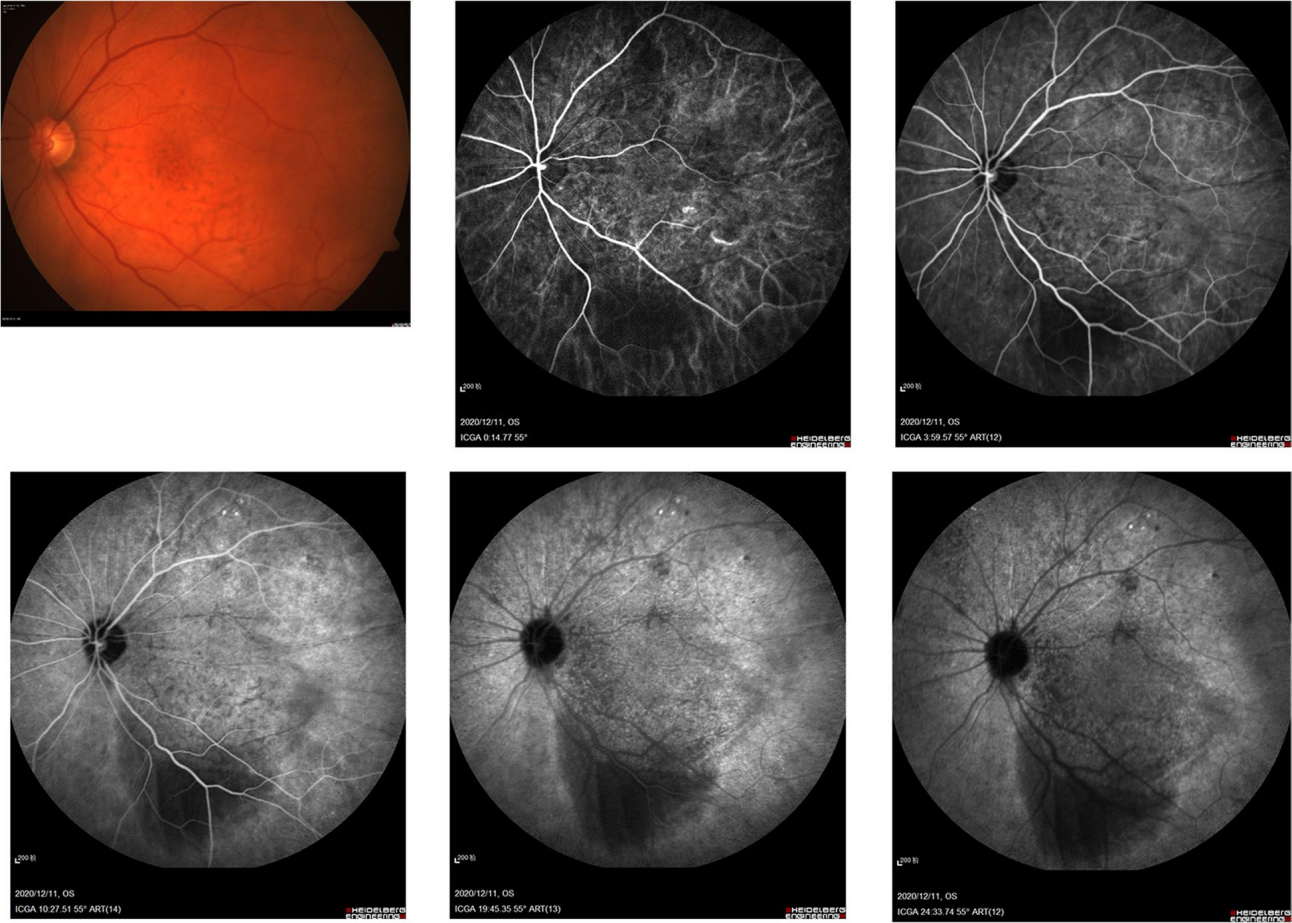
The ICGA results of the patients’ eyes. Rectangle-like weak fluorescence below the macula was shown during the early phase. Rectangle-like weak fluorescence below the macula and dot-like strong fluorescence above it were shown in the late phase

Besides, further examinations provided evidence for precise diagnosis (Table 2). B-scan ultrasonography showed mass with abundant blood and irregular cysts inside the eyeball wall. Irregular hypoechoic areas could also be detected in the retrobulbar orbit, surrounding the ocular wall and optic nerve (Fig. 2). In addition, UBM showed solid lesions with low and medium echo under the conjunctiva and inside the ciliary body of 2 cases (Fig. 3). To further reveal the damage of the lesion to the retinal and choroidal structures and blood vessels, the OCT examination was conducted. The results showed posterior polar wavy rise of retinal pigment epithelium (RPE) and local neuroepithelial detachment in all 3 cases (Fig. 4). Local thickening of choroidal were clearly shown in 2 cases. As shown in Figs. 5 and  6, the results of FFA and ICGA showed changes of eyeballs which mainly involved vascular abnormalities (patch-like strong fluorescence and fluorescence leakage in the FFA images and rectangle-like weak fluorescence below the macula in the ICGA images). However, no obvious abnormal blood vessels were found in the ICGA images.

**Table 2:** The examination results of the patients

Patient No	B-scan	UBM	OCT	FFA	ICGA	MRI	PET-CT
1	Significantly thickened eyeball with reduced internal echo could be seen. Irregular hypoechoic areas with abundant blood flow surrounded the bulbar wall and optic nerve.	Hypoechoic lesions under the conjunctiva with uneven internal echo could be seen. The boundary between the lesion and sclera was still clear.	Wavy rise of RPE and local neuroepithelial detachment could be seen.	Patch-like strong fluorescence could be seen around the optic disc in the venous phase, with radial weak fluorescent strips between them.	Patch-like strong fluorescence could be seen in the early stage, mixed with radial low fluorescence (choroidal folds); rectangular low fluorescence below the macula could be seen in both early and late stage.	The posterior wall of the eyeball was unevenly thickened (equal signal intensity on T1and T2 weight images); the anterior wall was thickened (equal T1 and higher T2); irregular soft tissue image could be seen around the eyeball and the optic nerve (equal T1 and equal / higher T2 signals), the boundary is unclear and the mass could be enhanced.	/
2	Irregular bulge lesions on the global wall of eyeball could be seen. Irregular hypoechoic lesions with abundant blood flow could be seen in the orbit.	Hypoechoic solid lesions could be seen in the ciliary body, with uniform internal echo and clear boundary with sclera.	Wavy rise of RPE and local neuroepithelial detachment could be seen. Local thickening of choroidal could be seen.	Strong fluorescence appeared on the nasal side of the optic disc during the venous phase. Fluorescence leakage occurred in the late stage.	ICGA showed strong fluorescence, but no obvious abnormal blood vessels were found. The lower half of retina was raised.	Arc and spindle signal shadow (equal T1 and equal T2) could be detected on the posterior wall of the eyeball. The mass could be moderately enhanced.	The soft tissue behind the vitreous body of the eyeball was thickened and the metabolism was enhanced.
3	Thickened eyeball with uniform reduced internal echo and abundant blood flow signals could be seen. Irregular hypoechoic areas surrounding the eyeball wall and optic nerve could be seen in the retrobulbar orbit. CDFI showed rich blood flow signals.	/	Wavy rise of RPE and local neuroepithelial detachment could be seen. Local thickening of choroidal could be seen.	Rising lesions with patch-like strong fluorescence could be seen in the inferior temporal side of the macula. Fluorescence leakage occurred in the late stage around the optic disc.	/	The eyeball wall was diffusely thickened, especially the posterior wall. Arc soft tissue shadows were seen in the space within the retrobulbar muscle cone, showing equal T1 and equal T2 signal shadows. The optic disc was involved in the lesion, which surrounded the intraorbital segment of the optic nerve. The optic nerve was compressed and narrowed in the front. The anterior chamber became shallow.	The soft tissue behind the vitreous body of the eyeball was thickened and the metabolism was enhanced.

Systemic workups, including orbital MRI and PET-CT imaging tests, were also conducted. According to results of MRI, all the eyeball walls were diffusely thickened and were surrounded by irregular soft tissue. In 2 cases, the lesion surrounded the intraorbital segment of the optic nerve, which was compressed and narrowed. PET-CT also showed thickening of posterior wall of eyeballs and increased metabolic activity, indicating the possibility of malignant tumors. However, there was no other evidence and the result of blood test showed no evidence of infection and systemic autoimmune diseases.

**Fig. 7:**
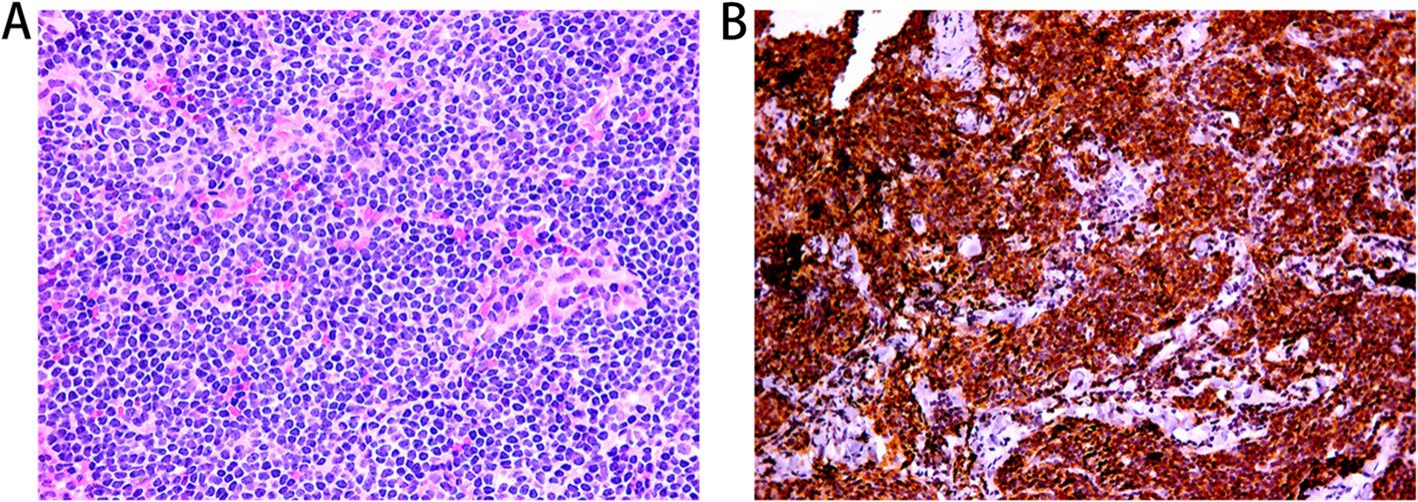
The pathology report of the surgical biopsy confirmed the diagnosis of the lesion as choroidal MALT lymphoma. **A** The HE staining figure of biopsy. **B** The figure of immunohistochemical staining of CD20

Due to the suspicion of uveal neoplasm, all patients were referred to an oncologist and underwent diagnostic surgeries with conjunctiva biopsy of their eyes. The pathology report of the surgical biopsy confirmed the diagnosis of the lesion as extranodal marginal zone B-cell lymphoma of mucosa-associated lymphoid tissue in choroidal (Fig. 7). Immunohistology of conjunctiva was positive for CD10( +), CD20(+ +), CD79a( +), κ( +),LCA( +) and bcl-2( +). Because there was no evidence of MALT lymphoma in other sites of this patient, the tumor stage was IE according to the Ann Arbor grading system.

All the patients received intravitreal injection of 400 μg/0.1 ml of methotrexate (MTX) monthly for 3 months and were referred to an oncologist for further treatments. The patients’ visual acuity did not improve. There was no sign of progress in their eyeball and systemic lymphoma on the latest follow-up.

## Discussion

Ocular MALT lymphoma involving orbit and conjunctiva usually has an indolent clinical course during which “salmon patch” mass is the typical presentation [16–18]. However, in a very few cases, MALT lymphoma can involve uvea, which makes these series of cases special. Clinically, it can masquerade as posterior scleritis and can also be easily misdiagnosed as uveitis and choroidal hemangioma. Posterior scleritis is mostly a unilateral disease with strong female predominance. The most common presenting symptoms of this disease are pain and decreased vision [19]. In our study, the patients complained about their red eyes with blurred vision but had no typical symptoms like “salmon patch” initially. Unfortunately, they were initially misdiagnosed as scleritis and were treated with corticosteroid before the first visit in our hospital. Their symptoms were not relieved.

Among all the clinical examinations of ocular MALT lymphoma in uvea, B-scan ultrasonography is the most valuable imaging modality [20]. Typical ultrasonographic features are hollow (hypoechoic) choroidal thickening and local hypoechoic extrascleral extension (ESE). The combination of these two signs is highly suggestive of ocular MALT lymphoma in uvea, but it should be noted that similar ultrasound findings can also be seen in diffuse choroidal melanoma with ESE [21]. They still need to be differentiated through pathologic examinations of biopsy. In our study, the result of B-scan ultrasonography showed significantly thickened eyeball with reduced internal echo in all cases. Besides, irregular hypoechoic lesions with abundant blood flow were found in the orbit (ESE in all cases). The lesions surrounded the bulbar wall and optic nerve in 2 cases. These results of ultrasonography not only indicated the mass with abundant blood and irregular cysts in the eyeball wall, but were helpful to the extent of involvement of ocular lymphoma in uveal tissue.

Compared with ultrasonography, FFA, ICGA and MRI are of limited value in the diagnosis of ocular MALT lymphoma in uvea. FFA could show weak fluorescence at early phase and strong fluorescence at middle and late phase in the lesions of ocular MALT lymphoma in uvea [22]. Both early and late ICGA could show weak choroidal fluorescence (choroidal folds) in the lesions. In our cases, all the FFA results showed patch-like strong fluorescence during the venous phase and fluorescein leakage in the late stage. The ICGA results of 2 cases both showed strong fluorescence. Both of these examinations lacked characteristics. Conversely, MRI is helpful to find orbital involvement of ocular MALT lymphoma by showing thickened and enhanced wall of the eyeballs [4, 23]. Also, it is much easier to detect lesions with different shapes and invasive growth through MRI, which often surround the eyeball, extraocular muscles and optic nerve. However, the signal of the lesions in our case is irregular and lack of characteristics, and the resolution is lower than that of ocular ultrasonography.

Although the accuracy of typical ocular manifestations and ultrasonography in the diagnosis of ocular MALT lymphoma in uvea is high, the gold standard of diagnosis are histopathology examination and immunophenotyping examination of surgical biopsy [21, 24]. Histologically, Suzuki et al. proposed several main pathological features for the diagnosis of MALT lymphoma, including (1) infiltrating lymphocytes are centrocyte-like cells (marginal zone cells) with irregular nuclei and abundant pale cytoplasm, (2) plasmocytic differentiation with or without Dutcher bodies (intracytoplasmic inclusions), (3) follicular colonization with infiltration of germinal centers by centrocyte-like cells or atrophic germinal center, (4) lymphoepithelial lesion (LEL) [25]. However, the characterization of ocular MALT lymphoma in uvea is not as simple. Similar to POAML, some of the histological features observed in this special lymphoma, such as invasive lesions, reactive B-cell follicles and LELs overlap with the histological features of reactive lymphoepithelial infiltration in the ocular adnexa and uvea, which may make it difficult to diagnose precisely [26]. In our study, the diagnosis of all the patients in our study were eventually confirmed by biopsy of their involved tissue. Furthermore, compared with the histological examination, immunophenotyping and molecular diagnostic techniques have greatly improved the diagnostic level of ocular MALT lymphoma. The characteristic immunophenotypic profiles are CD10^neg^, CD20^pos^, CD23^neg^, BCL2^neg^, BCL6^neg^ and Cyclin D1^neg^with a few interspersed CD3^pos^T lymphocytes [3]. However, there is still a lack of specific markers and more research is needed.

We must admit that there were still some limitations in our study. Due to the rare incidence of ocular MALT lymphoma in uvea, only 3 cases were included in our study. The follow-up period was short and the data were incomplete. More studies will be conducted to summarize the clinical manifestations, ocular imaging features and pathological changes ocular MALT lymphoma in uvea in the future.

## Conclusions

In conclusion, the onset of primary ocular MALT lymphoma in uvea is hidden. The early clinical manifestations are lack of specificity, which may mislead the diagnosis. Although the accuracy of diagnosis of ocular MALT lymphoma in uvea is high through typical ocular manifestations and ultrasonography, the gold standard of diagnosis is pathological examination of tissue biopsy.

## Supplementary Information

**Additional file 1:** The raw data of this study. **Table 1.** The basic information of involved patients.

## Data Availability

The raw data of this study was included in the supplementary file 1.
