# Supplementary material for: Mucosal associated Lymphoid Tissue Lymphoma of the uvea: an analysis of 3 cases
Source: BMC Ophthalmol. 2022 Sep 19;22:371. doi: 10.1186/s12886-022-02598-2 (PMC9484074; doi:10.1186/s12886-022-02598-2)
Supplement: Supplementary file 1 — Additional file 1: The raw data of this study. Table 1. The basic information of involved patients. [file 12886_2022_2598_MOESM1_ESM.zip › 1/τùàσÄåμò┤τÉå/τùàσÄåμò┤τÉå.docx]

病历整理：郭金兰 女 1963-2-1

- 2020-11-11

VOD 1.0 VOS 0.3 IOP OD 16mmHg OS 18mmHg

左眼视力下降半年。

外院诊断为葡萄膜炎，曾口服激素哦治疗有效，停药后复发。

外院B超示：左眼视网膜脱离，脉络膜脱离，“T”形征（+），PMI：无

PE：右眼

左眼

验光：1.0 R：PL 1.0

0.4-L：+1.50DS/+0.50DC*10 0.7+1 （PHNI）

追加正散，诉实力变模糊

L：+1.50DS 0.7(PH0.8-)

Add: +2.75DS R:0.7- L: 0.5

Rx：百力特q2h，普南扑灵tid

- 2020-11-18

VOD 1.0 VOS 0.5 IOP OD 16.8mmHg OS 17.1mmHg

左眼红伴视力下降半年余，劳累时左眼异物感，

外院“葡萄膜炎”，否认全身病史，曾口菇及点用激素

PE：左后极部网脱

诊断：巩膜炎

治疗：左眼曲安奈德半球后注射，血常规，尿常规，免疫四项，结核干扰素释放试验，FFA

- 2020-11-25

VOD 1.2 VOS 0.6 IOP OD 18.0mmHg OS 19.3mmHg

FFA

PE：左后极部网脱

B超示：左眼内葡萄膜淋巴瘤可能大

胸部，双肺及纵隔CT平扫未见异常

眶MRI示左侧眼球后壁、眼环前壁、球筋膜囊增厚强化，视神经眶内段鞘膜强化，并肌椎内间隙软组织强化影，非特异性炎症可能性大。

UBM：左眼结膜下可见中低回声病变（最后处约1.25mm），内回升欠均匀，病变与巩膜分界尚清晰，上方根部虹膜膨隆遮挡巩膜突，睫状体可见，睫状体未见异常回声可见不均匀低回声病变，部分增厚。

- 2020-12-14血液科会诊
- 2020-12-17魏院长会诊

主诉：左眼视力下降2月，左眼红2月

既往：否认其他全身疾病

外院诊左眼葡萄膜炎，口服泼尼松片（8-7-6-5-4-3-2-1片/日）

PE：左眼球结膜鼻侧，上方，颞侧增厚，表面呈粉色鱼肉样，角膜清，前房中深，瞳孔圆，晶体略混，右眼晶体略混，余前节（-）左眼后极部弥漫扁平隆起，无色素，表面散在分布斑点
