# Supplementary figures and images for: Mucosal associated Lymphoid Tissue Lymphoma of the uvea: an analysis of 3 cases (part 2 of 2)
Source: BMC Ophthalmol. 2022 Sep 19;22:371. doi: 10.1186/s12886-022-02598-2 (PMC9484074; doi:10.1186/s12886-022-02598-2)

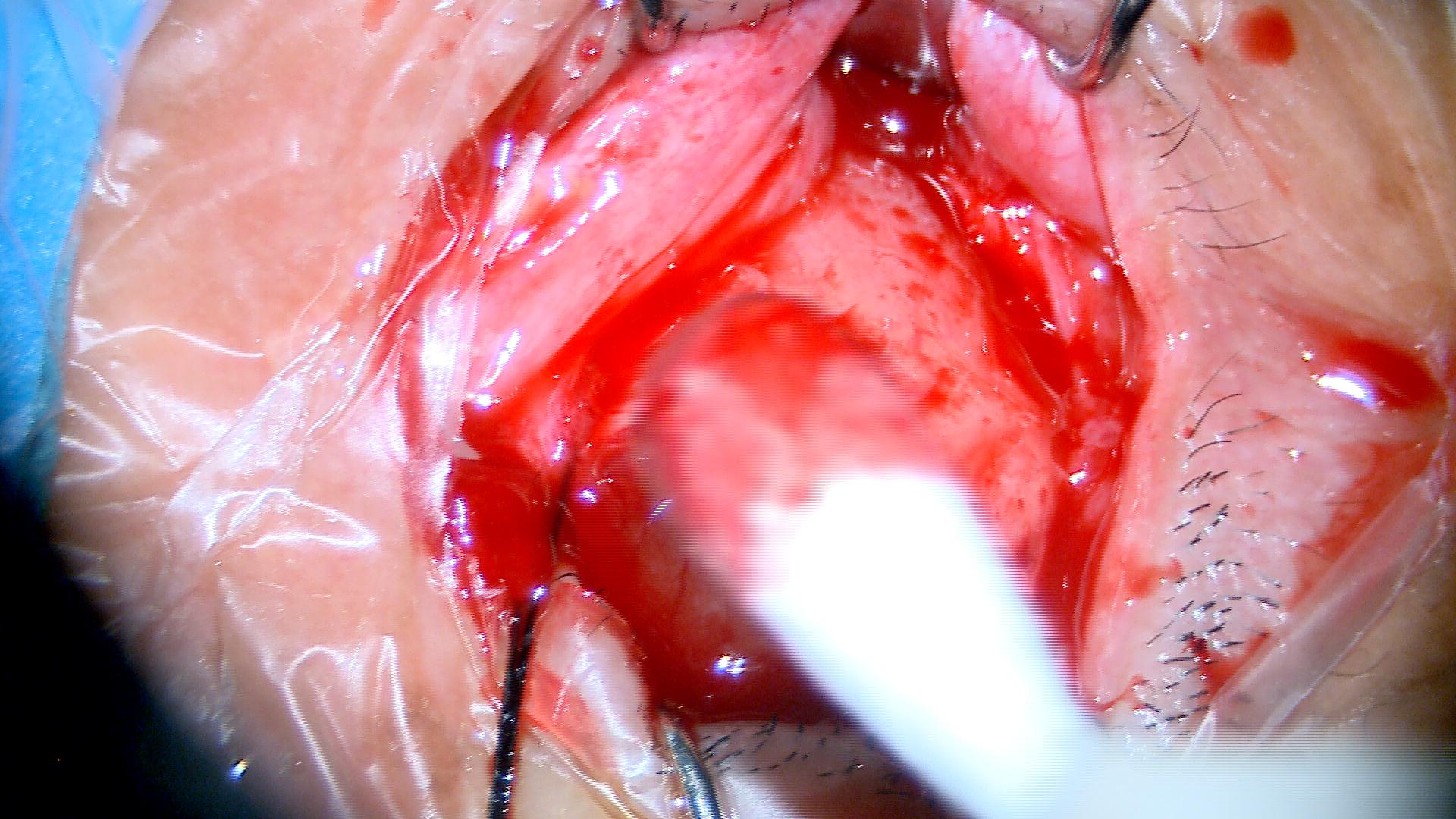

Supplement: Supplementary file 1 — Additional file 1: The raw data of this study. Table 1. The basic information of involved patients. [file 12886_2022_2598_MOESM1_ESM.zip › 3/μ£»Σ╕¡σñoΣ╜ôσâÅ/0123180228346.jpg]

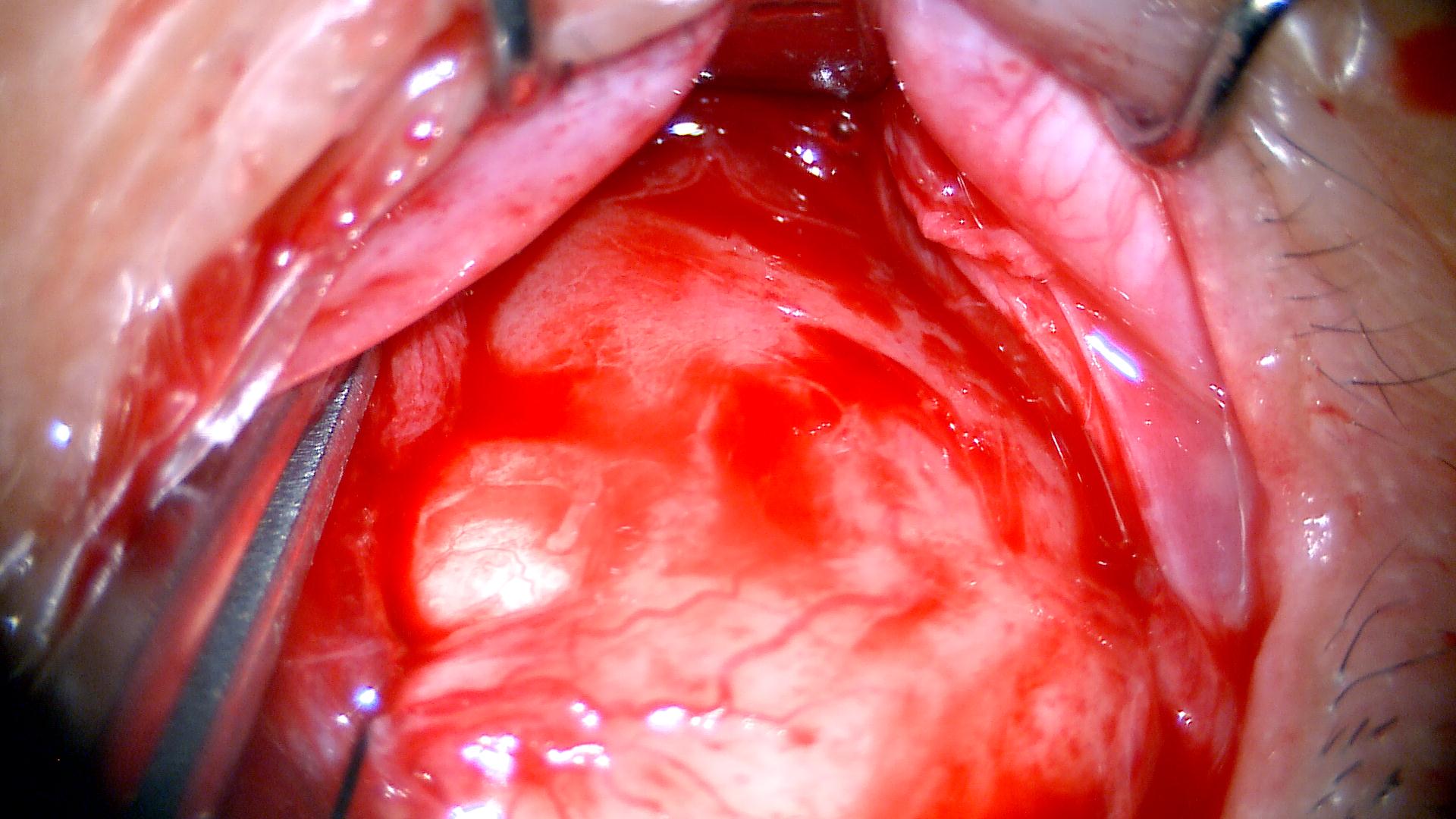

Supplement: Supplementary file 1 — Additional file 1: The raw data of this study. Table 1. The basic information of involved patients. [file 12886_2022_2598_MOESM1_ESM.zip › 3/μ£»Σ╕¡σñoΣ╜ôσâÅ/0123180346499.jpg]

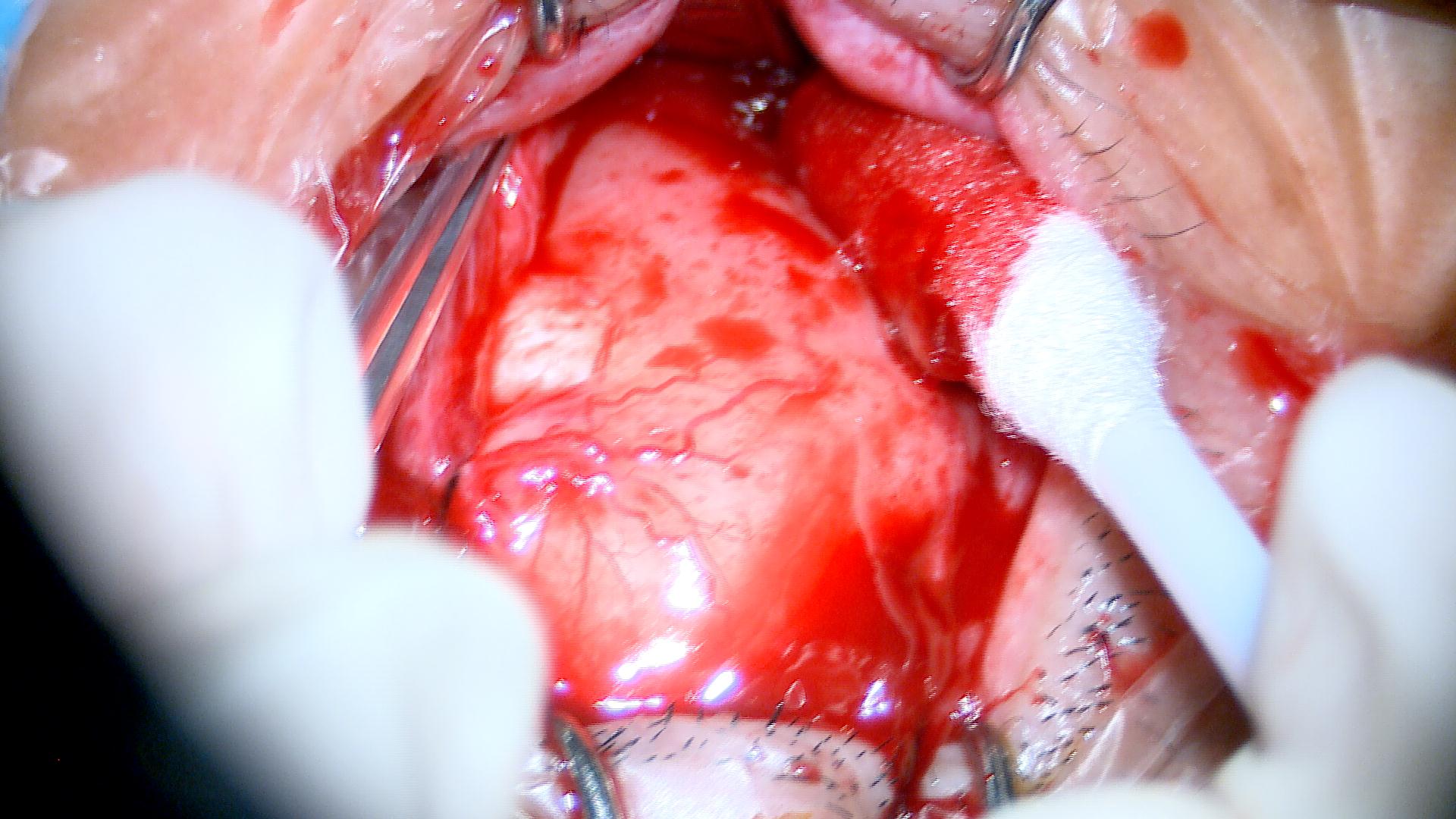

Supplement: Supplementary file 1 — Additional file 1: The raw data of this study. Table 1. The basic information of involved patients. [file 12886_2022_2598_MOESM1_ESM.zip › 3/μ£»Σ╕¡σñoΣ╜ôσâÅ/0123180312340.jpg]

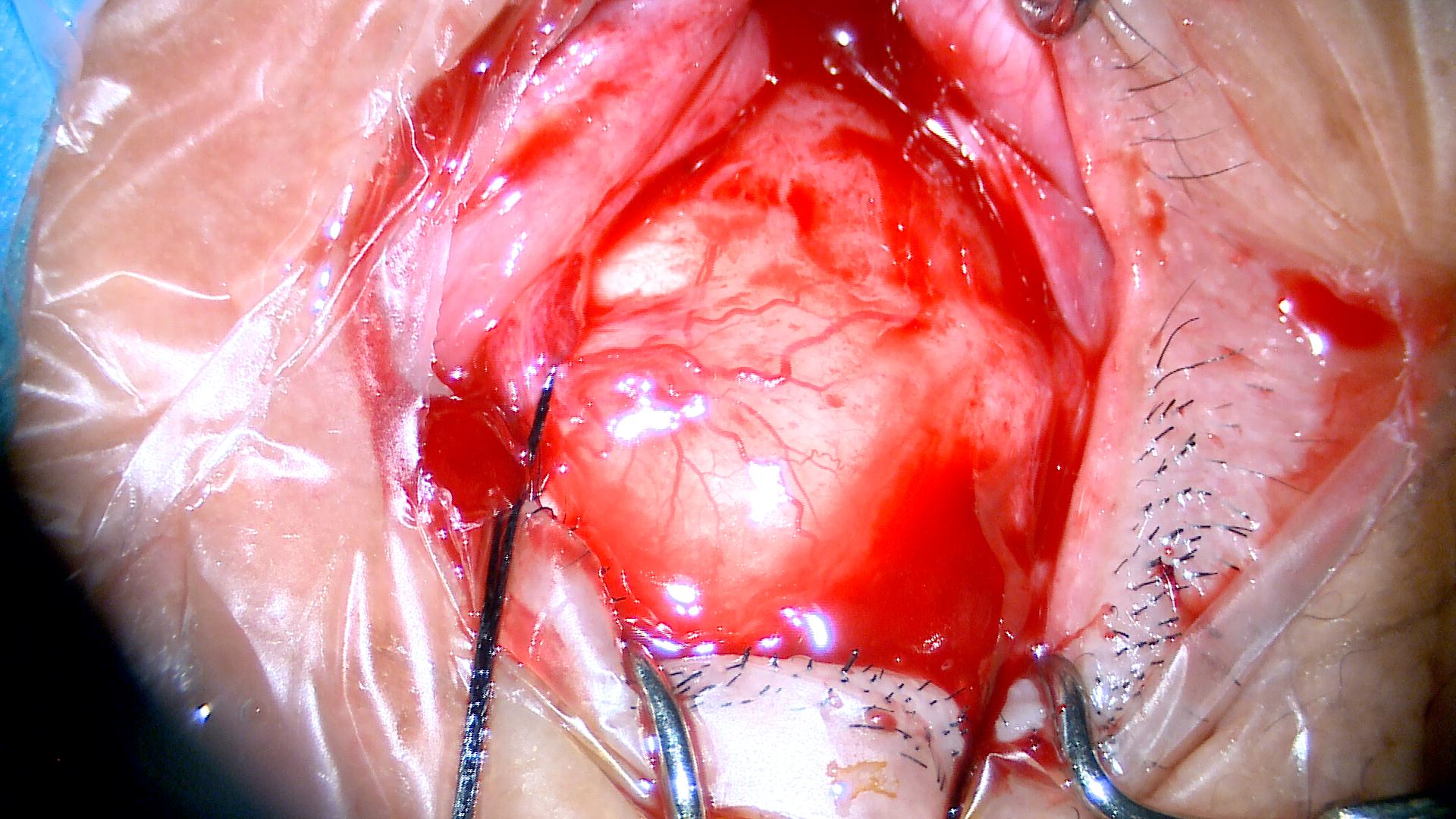

Supplement: Supplementary file 1 — Additional file 1: The raw data of this study. Table 1. The basic information of involved patients. [file 12886_2022_2598_MOESM1_ESM.zip › 3/μ£»Σ╕¡σñoΣ╜ôσâÅ/0123180248916.jpg]

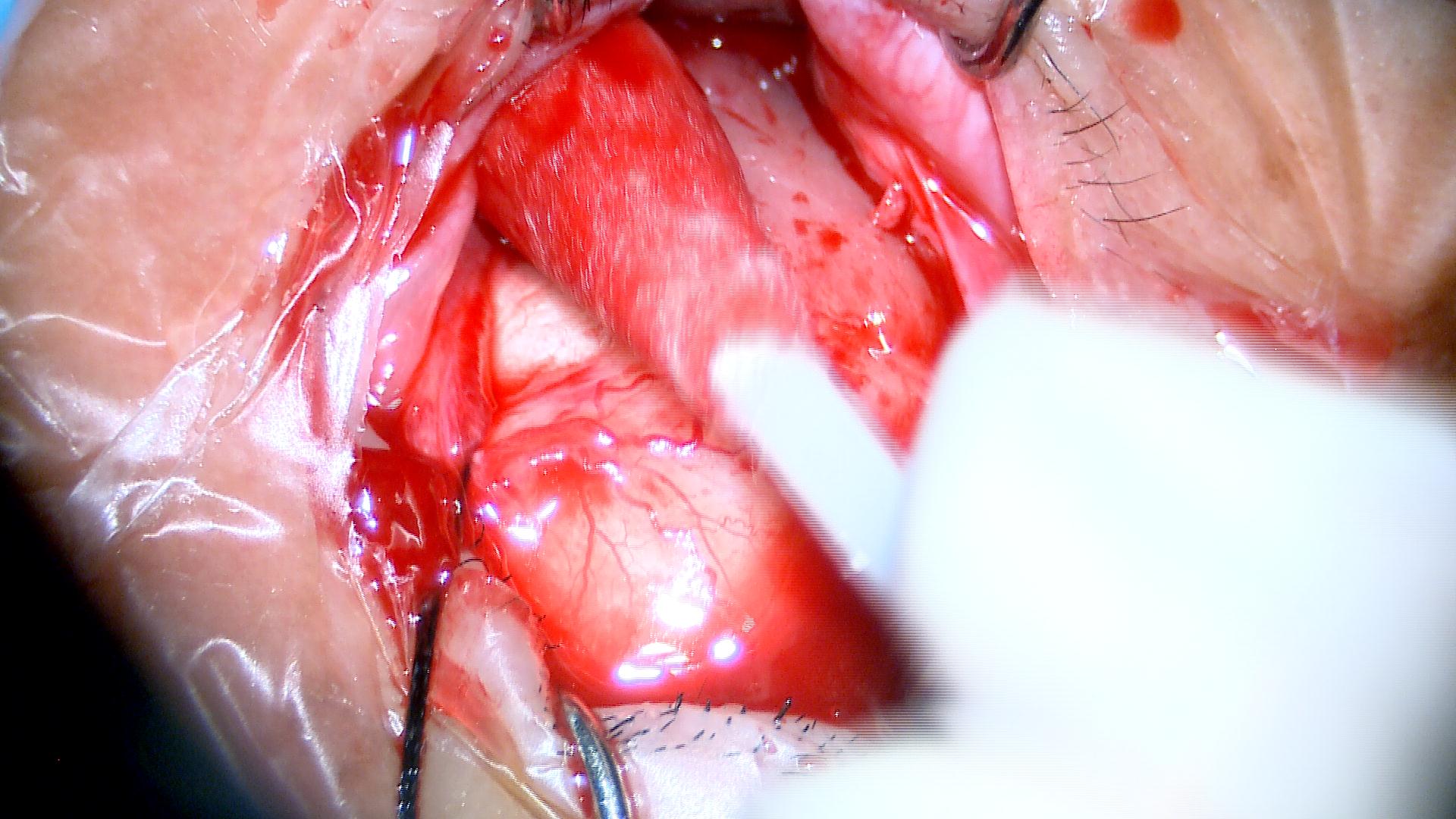

Supplement: Supplementary file 1 — Additional file 1: The raw data of this study. Table 1. The basic information of involved patients. [file 12886_2022_2598_MOESM1_ESM.zip › 3/μ£»Σ╕¡σñoΣ╜ôσâÅ/0123180243499.jpg]

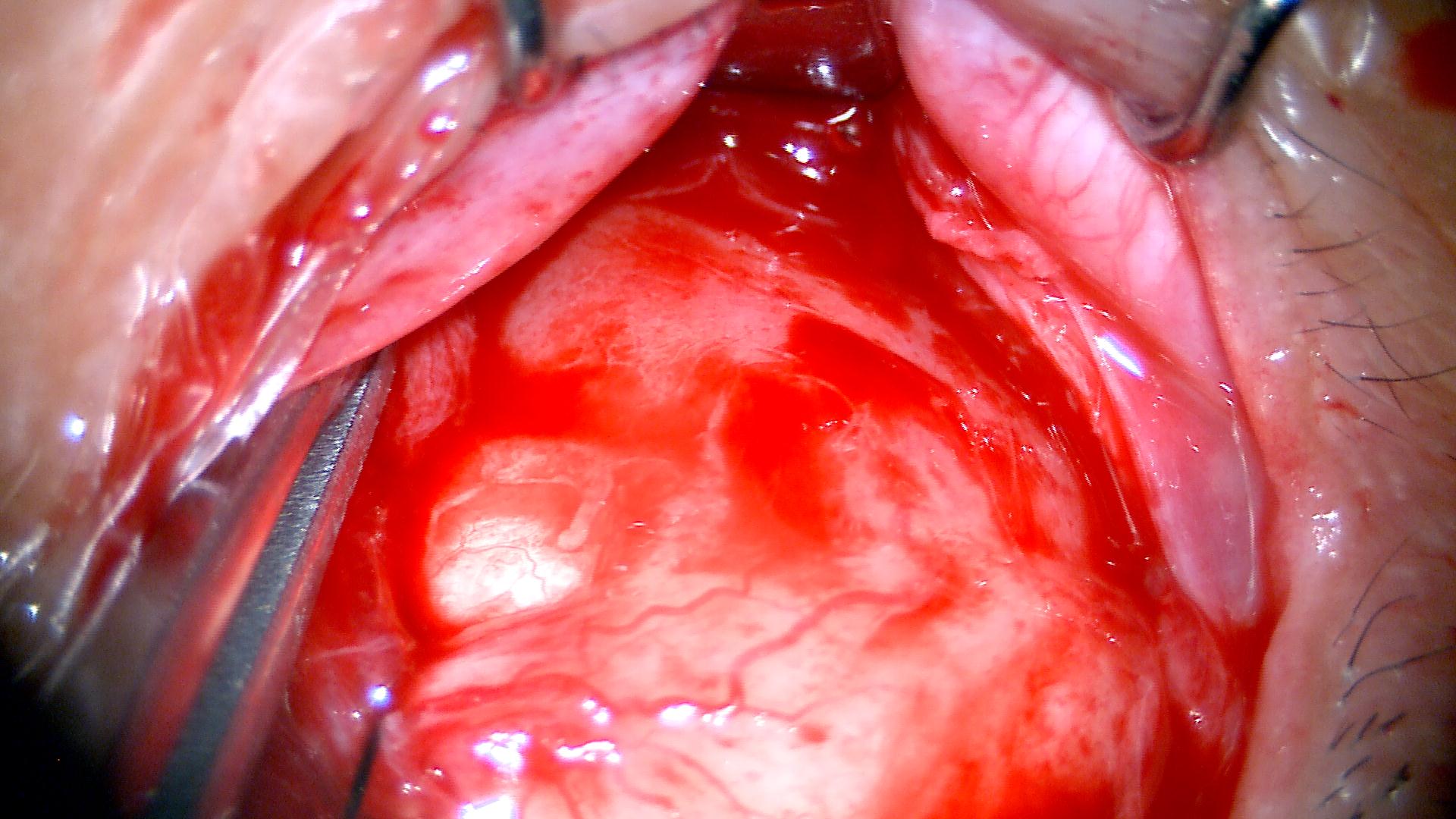

Supplement: Supplementary file 1 — Additional file 1: The raw data of this study. Table 1. The basic information of involved patients. [file 12886_2022_2598_MOESM1_ESM.zip › 3/μ£»Σ╕¡σñoΣ╜ôσâÅ/0123180347563.jpg]

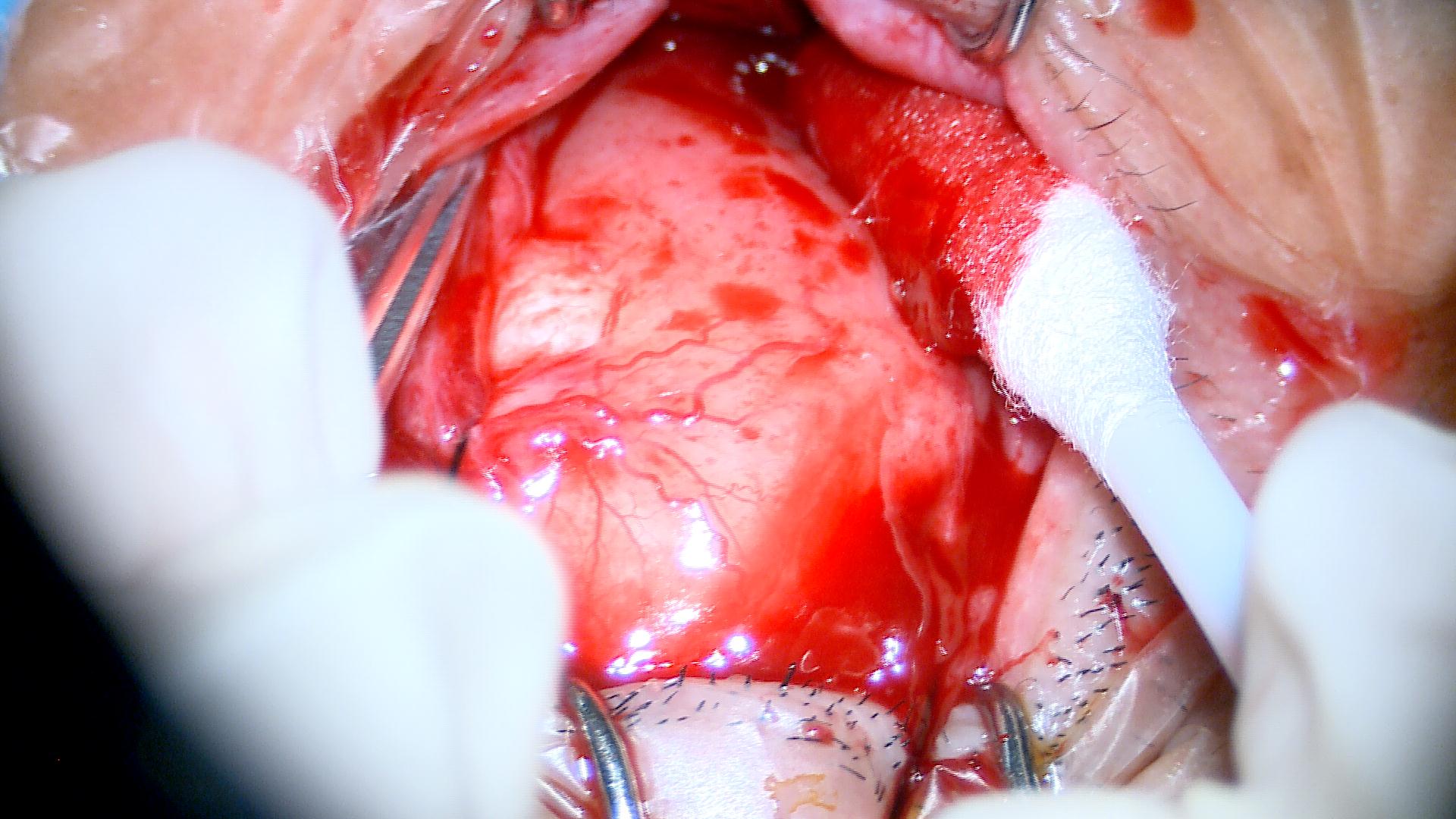

Supplement: Supplementary file 1 — Additional file 1: The raw data of this study. Table 1. The basic information of involved patients. [file 12886_2022_2598_MOESM1_ESM.zip › 3/μ£»Σ╕¡σñoΣ╜ôσâÅ/0123180311282.jpg]

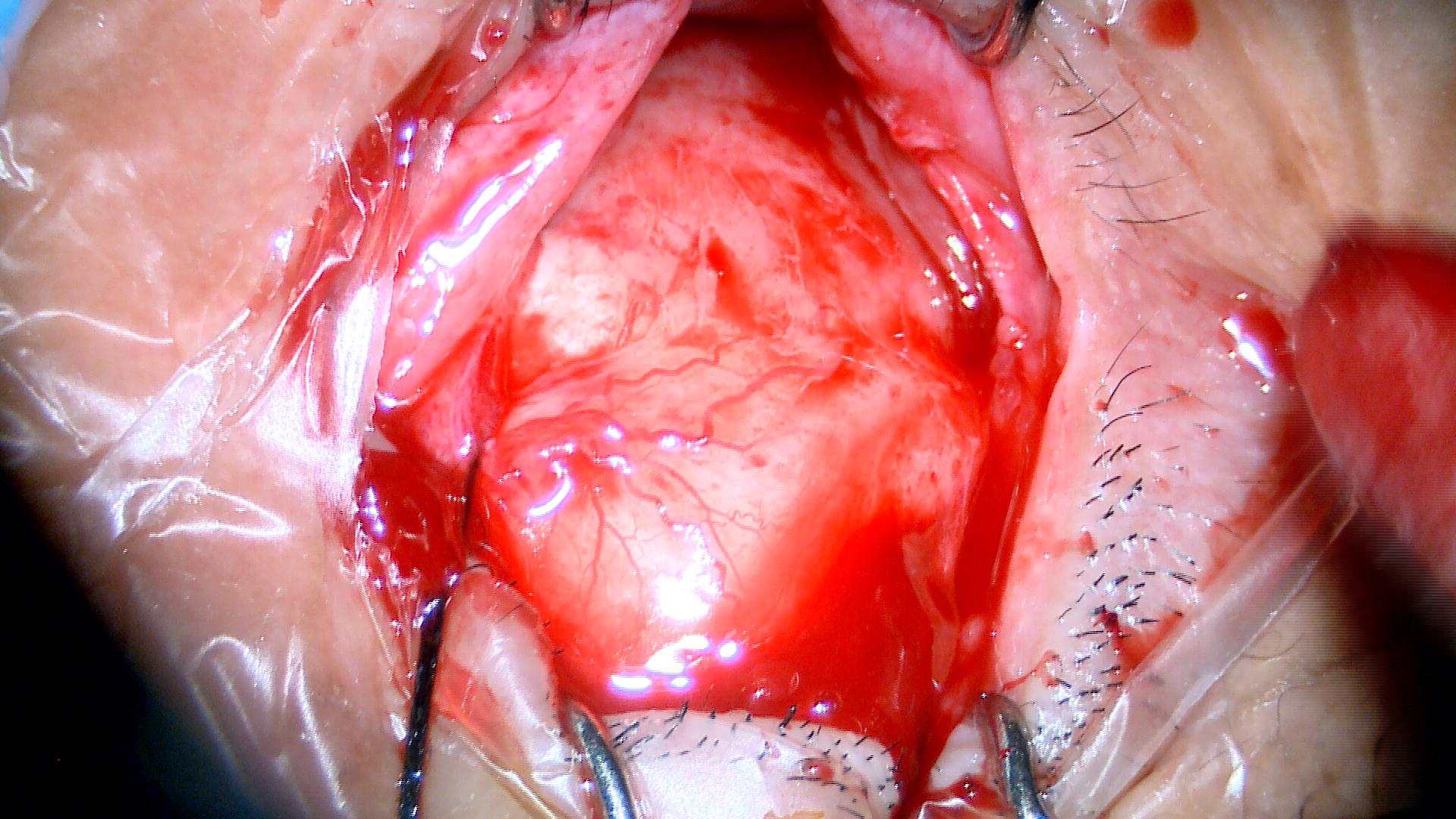

Supplement: Supplementary file 1 — Additional file 1: The raw data of this study. Table 1. The basic information of involved patients. [file 12886_2022_2598_MOESM1_ESM.zip › 3/μ£»Σ╕¡σñoΣ╜ôσâÅ/0123180234571.jpg]

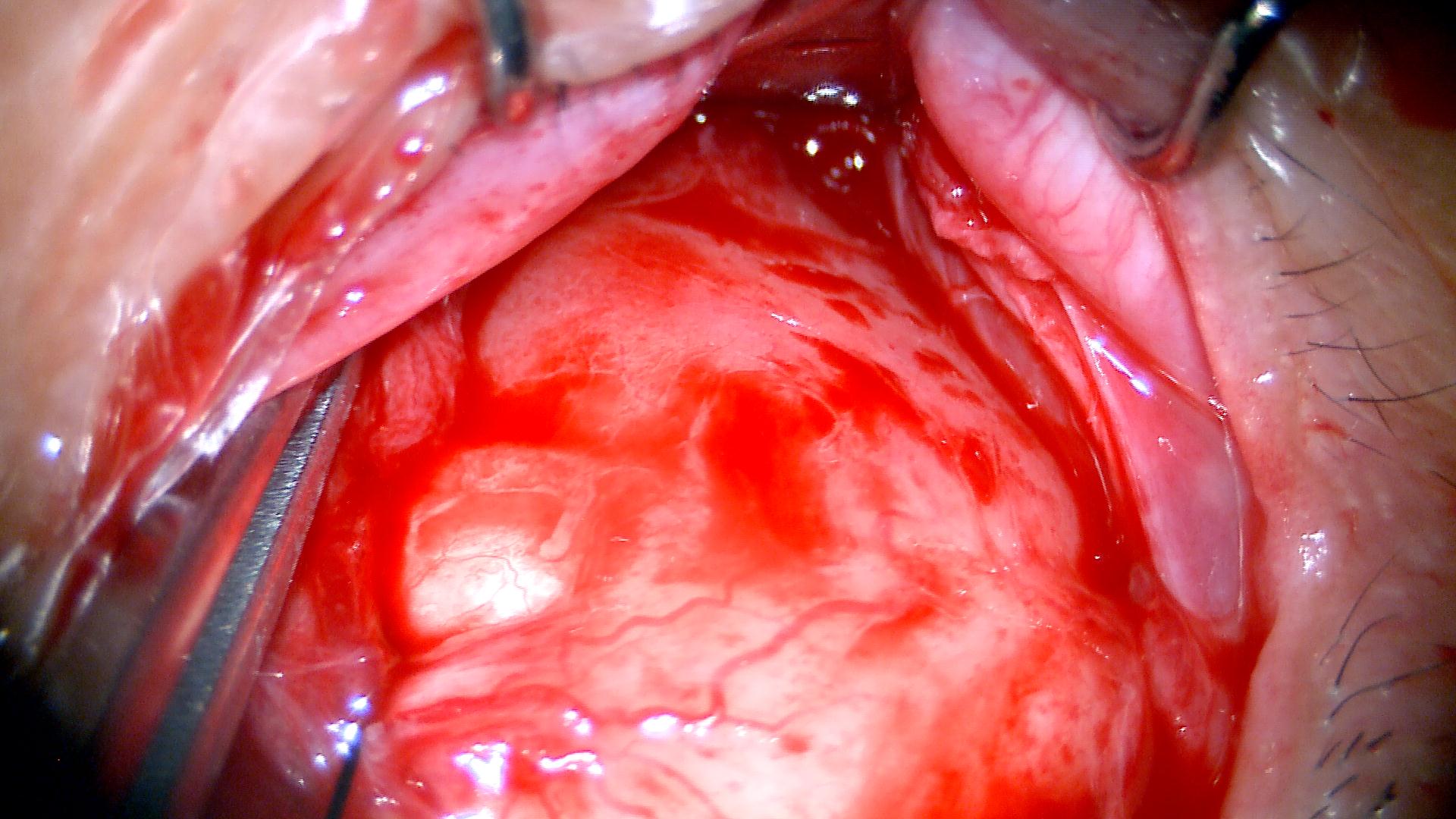

Supplement: Supplementary file 1 — Additional file 1: The raw data of this study. Table 1. The basic information of involved patients. [file 12886_2022_2598_MOESM1_ESM.zip › 3/μ£»Σ╕¡σñoΣ╜ôσâÅ/0123180344519.jpg]

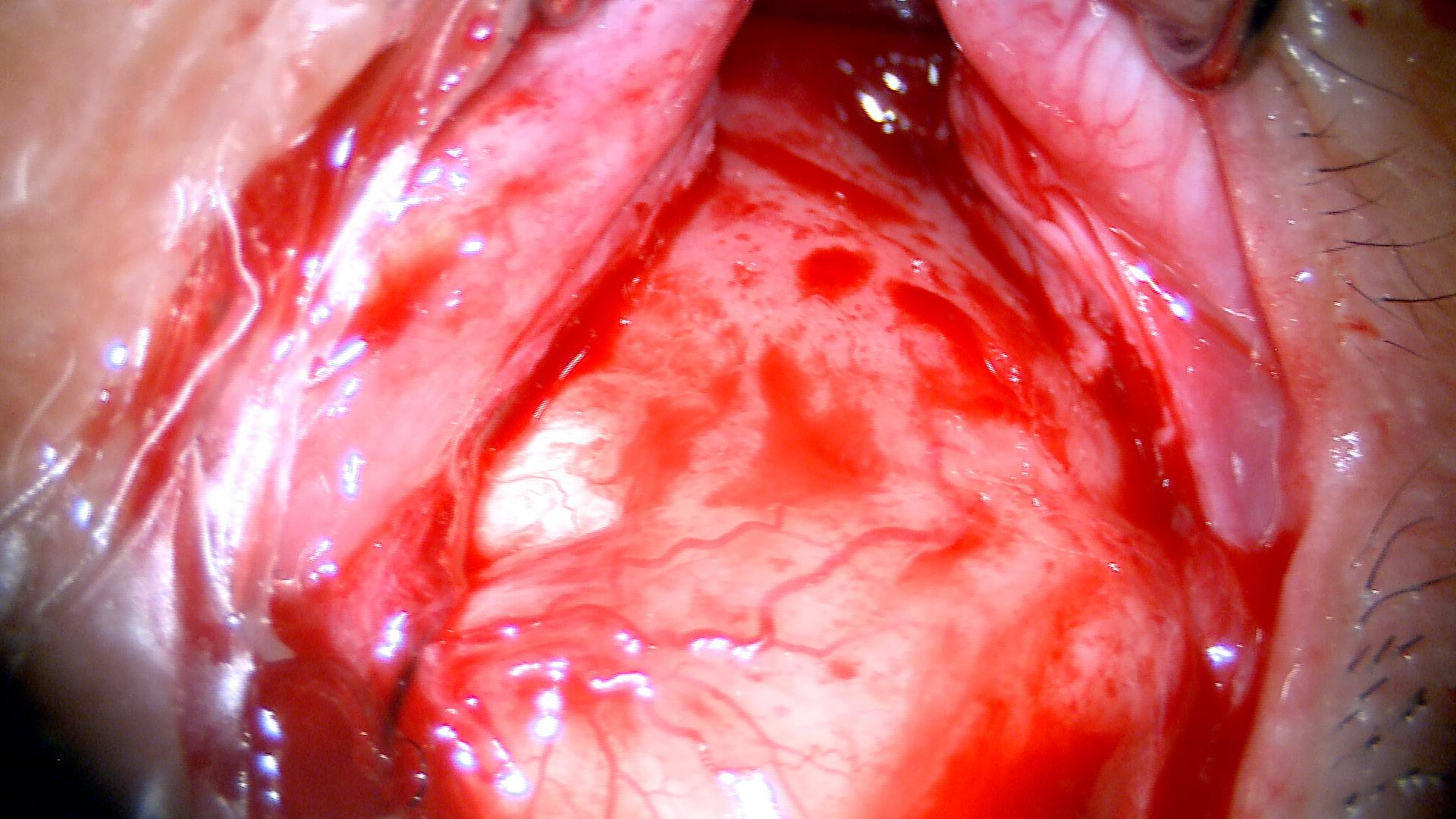

Supplement: Supplementary file 1 — Additional file 1: The raw data of this study. Table 1. The basic information of involved patients. [file 12886_2022_2598_MOESM1_ESM.zip › 3/μ£»Σ╕¡σñoΣ╜ôσâÅ/0123180329083.jpg]

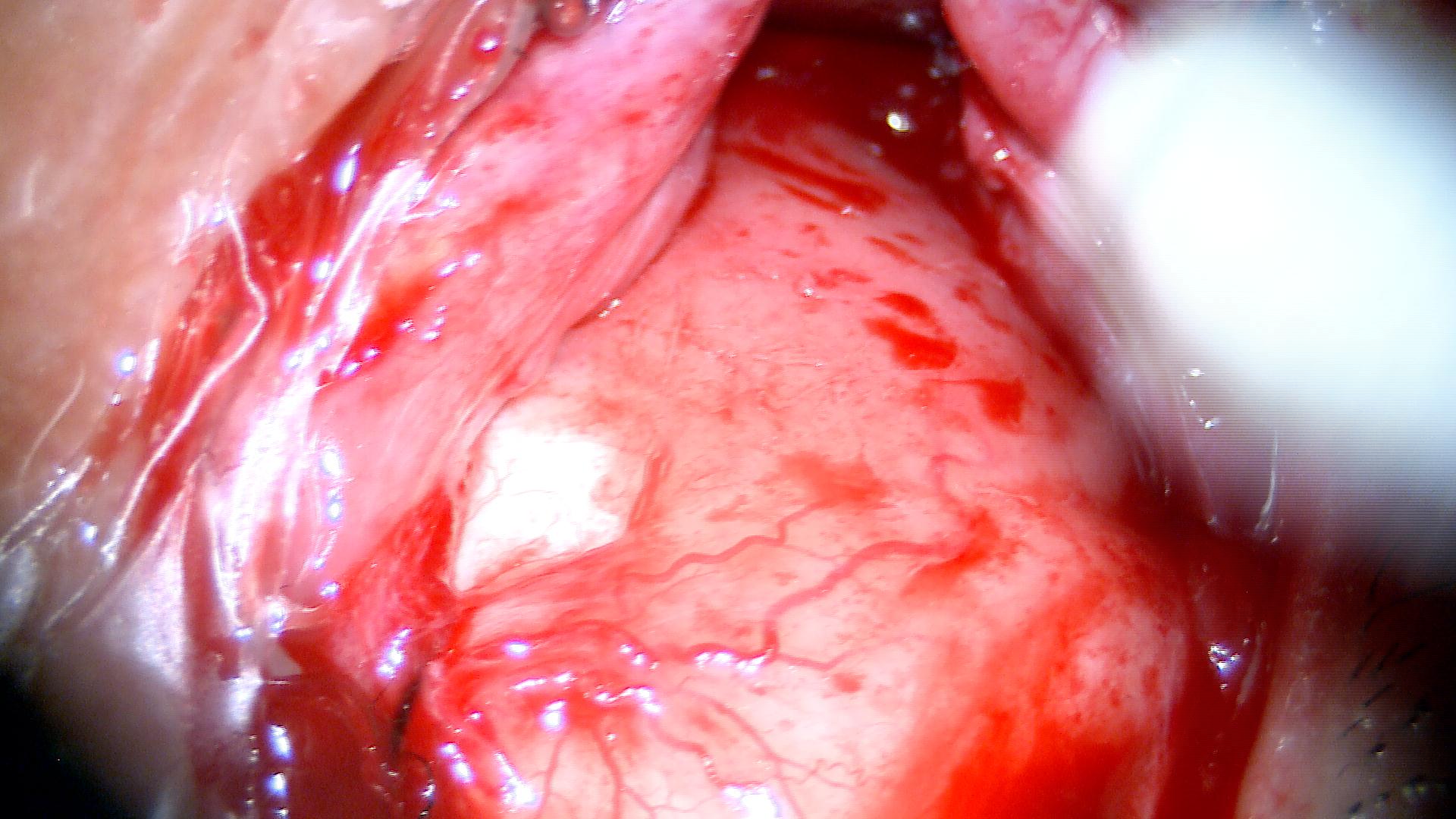

Supplement: Supplementary file 1 — Additional file 1: The raw data of this study. Table 1. The basic information of involved patients. [file 12886_2022_2598_MOESM1_ESM.zip › 3/μ£»Σ╕¡σñoΣ╜ôσâÅ/0123180323155.jpg]

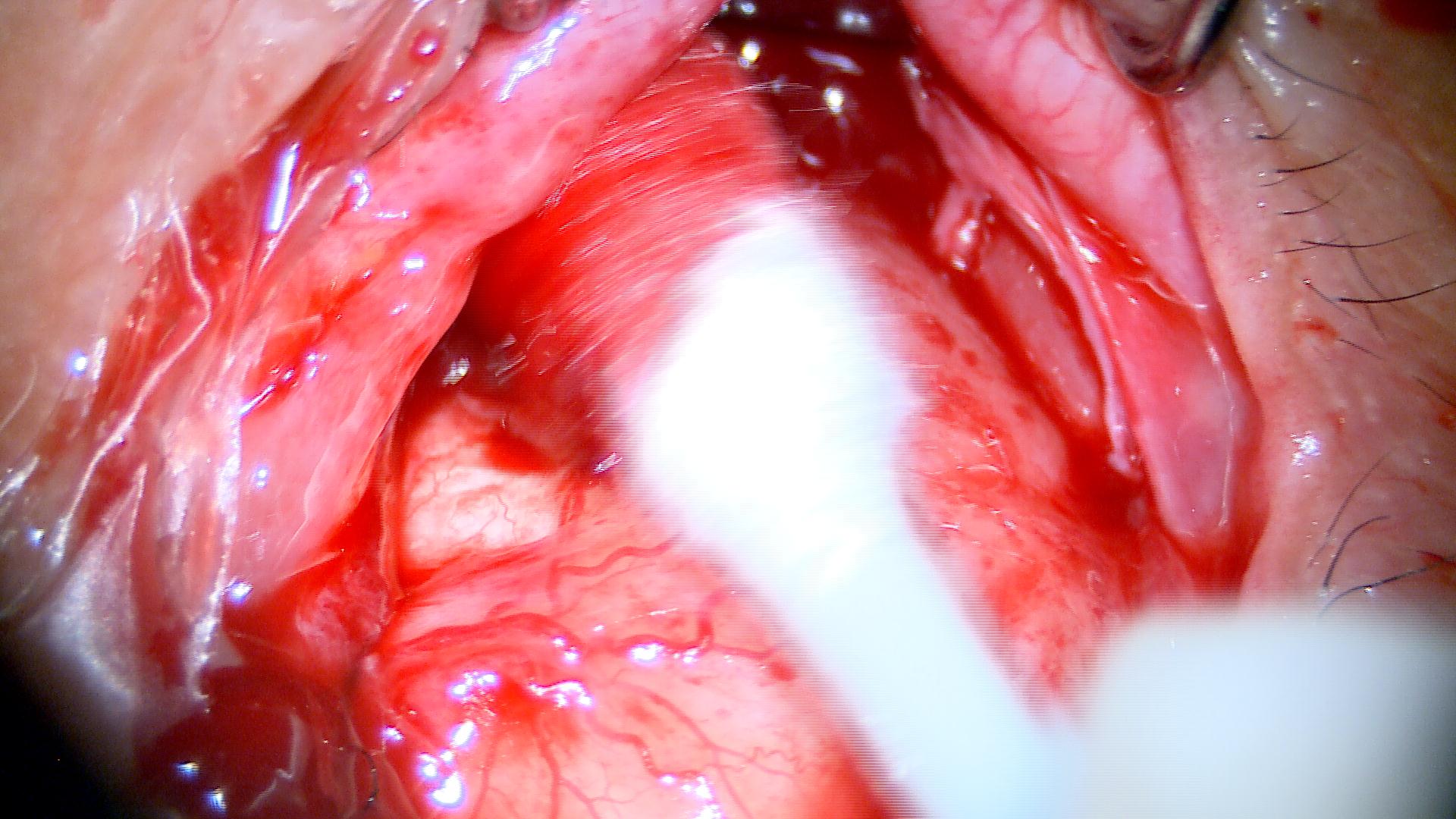

Supplement: Supplementary file 1 — Additional file 1: The raw data of this study. Table 1. The basic information of involved patients. [file 12886_2022_2598_MOESM1_ESM.zip › 3/μ£»Σ╕¡σñoΣ╜ôσâÅ/0123180318843.jpg]

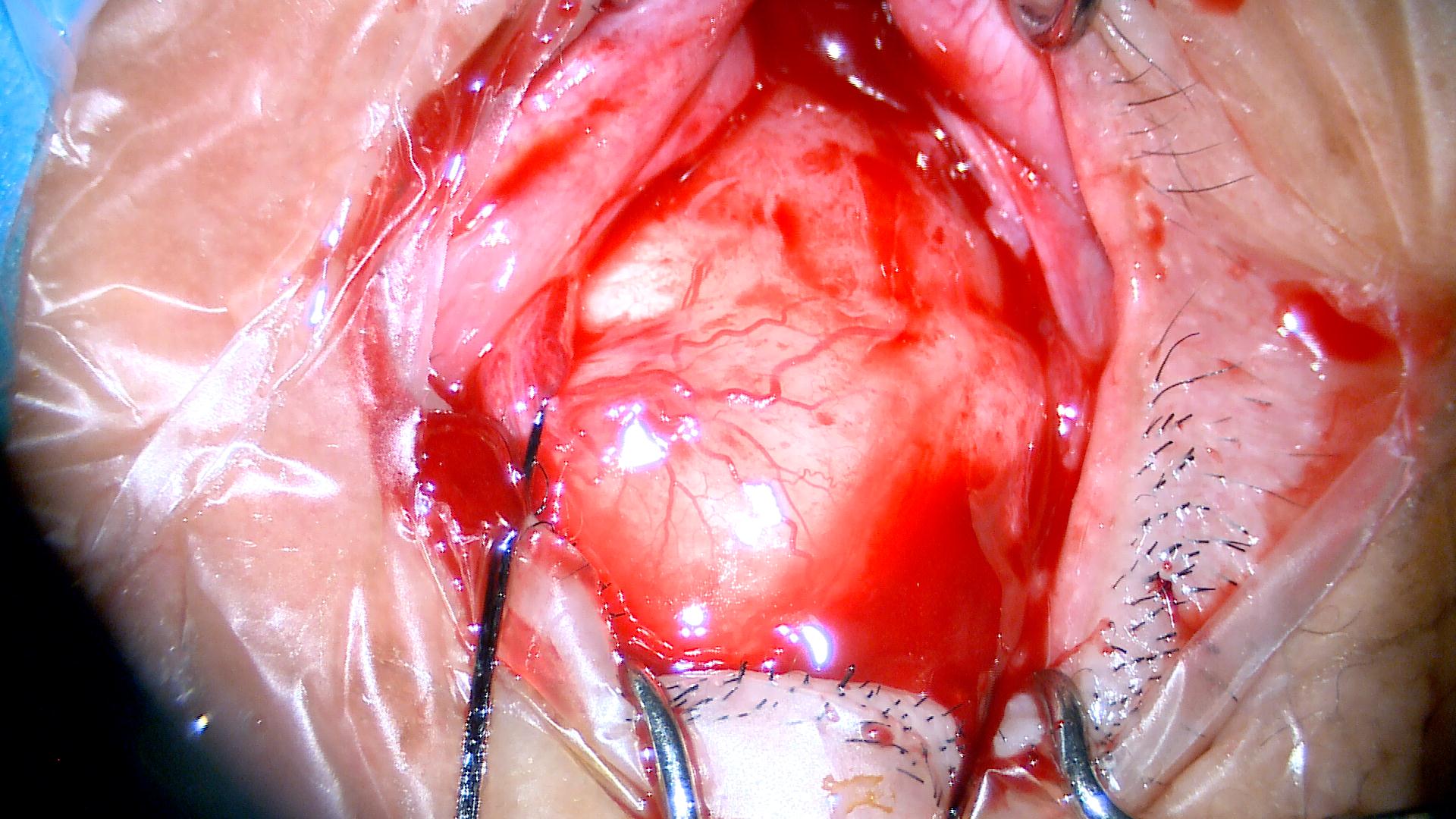

Supplement: Supplementary file 1 — Additional file 1: The raw data of this study. Table 1. The basic information of involved patients. [file 12886_2022_2598_MOESM1_ESM.zip › 3/μ£»Σ╕¡σñoΣ╜ôσâÅ/0123180248133.jpg]

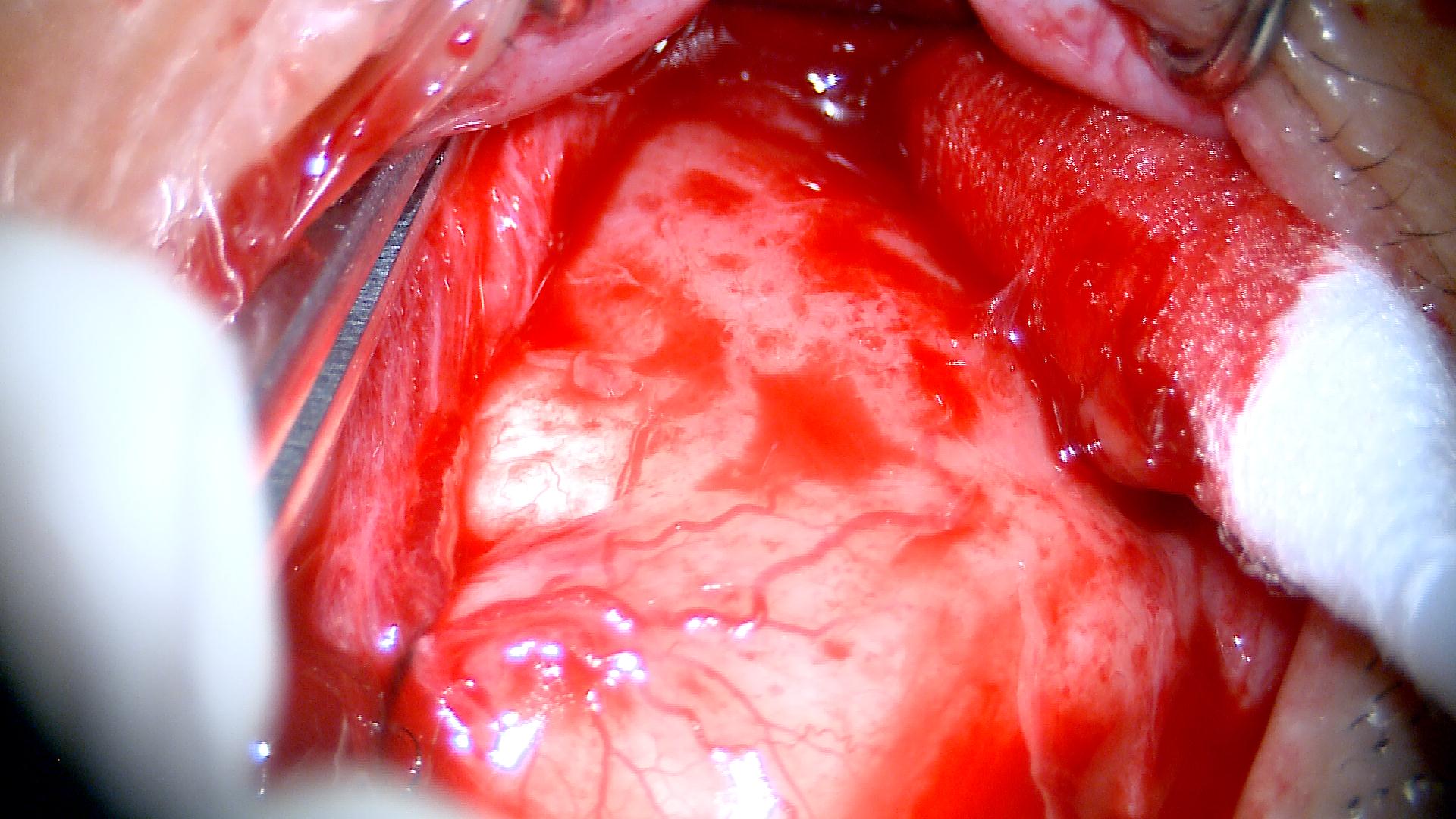

Supplement: Supplementary file 1 — Additional file 1: The raw data of this study. Table 1. The basic information of involved patients. [file 12886_2022_2598_MOESM1_ESM.zip › 3/μ£»Σ╕¡σñoΣ╜ôσâÅ/0123180316715.jpg]

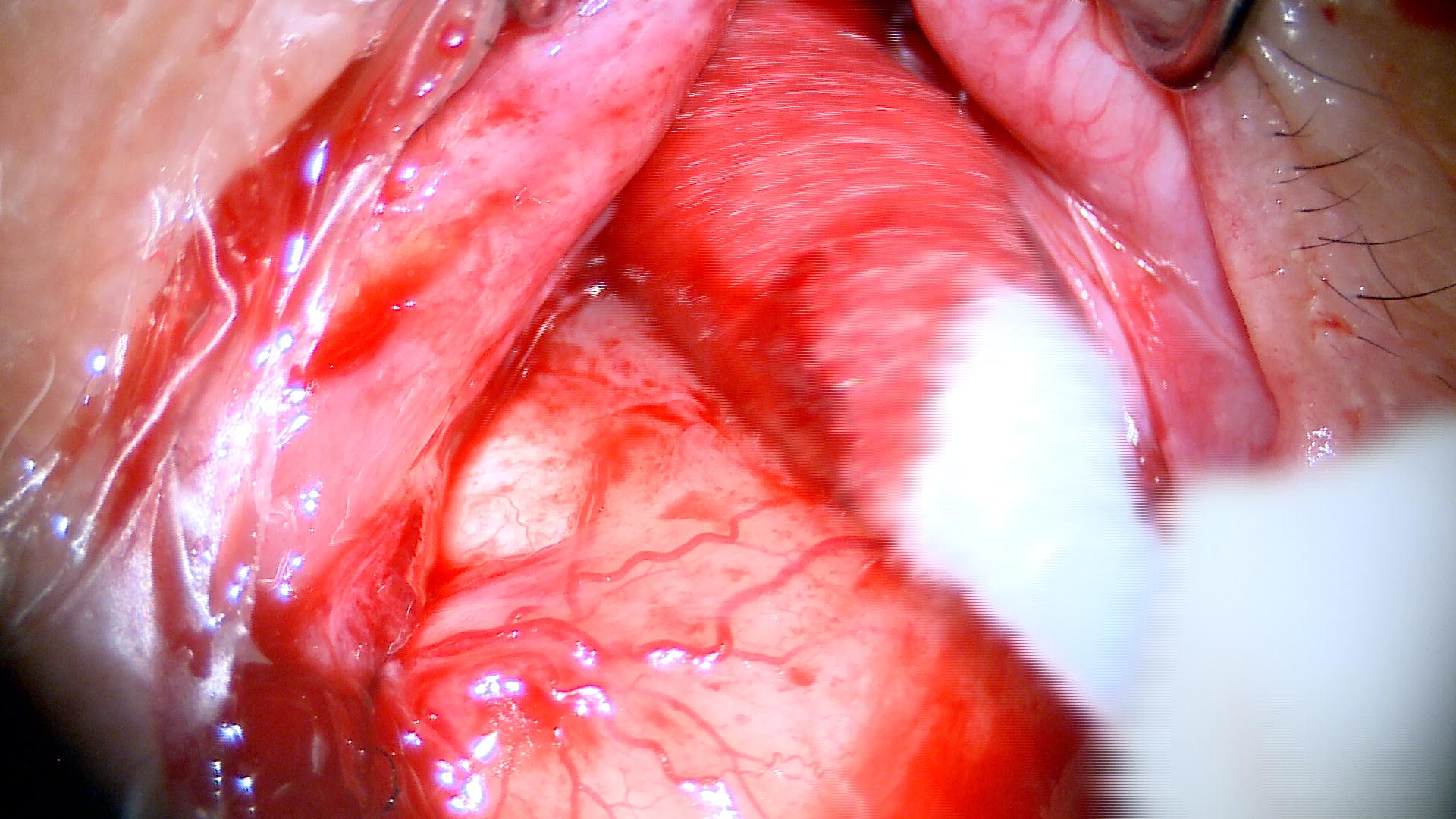

Supplement: Supplementary file 1 — Additional file 1: The raw data of this study. Table 1. The basic information of involved patients. [file 12886_2022_2598_MOESM1_ESM.zip › 3/μ£»Σ╕¡σñoΣ╜ôσâÅ/0123180321083.jpg]

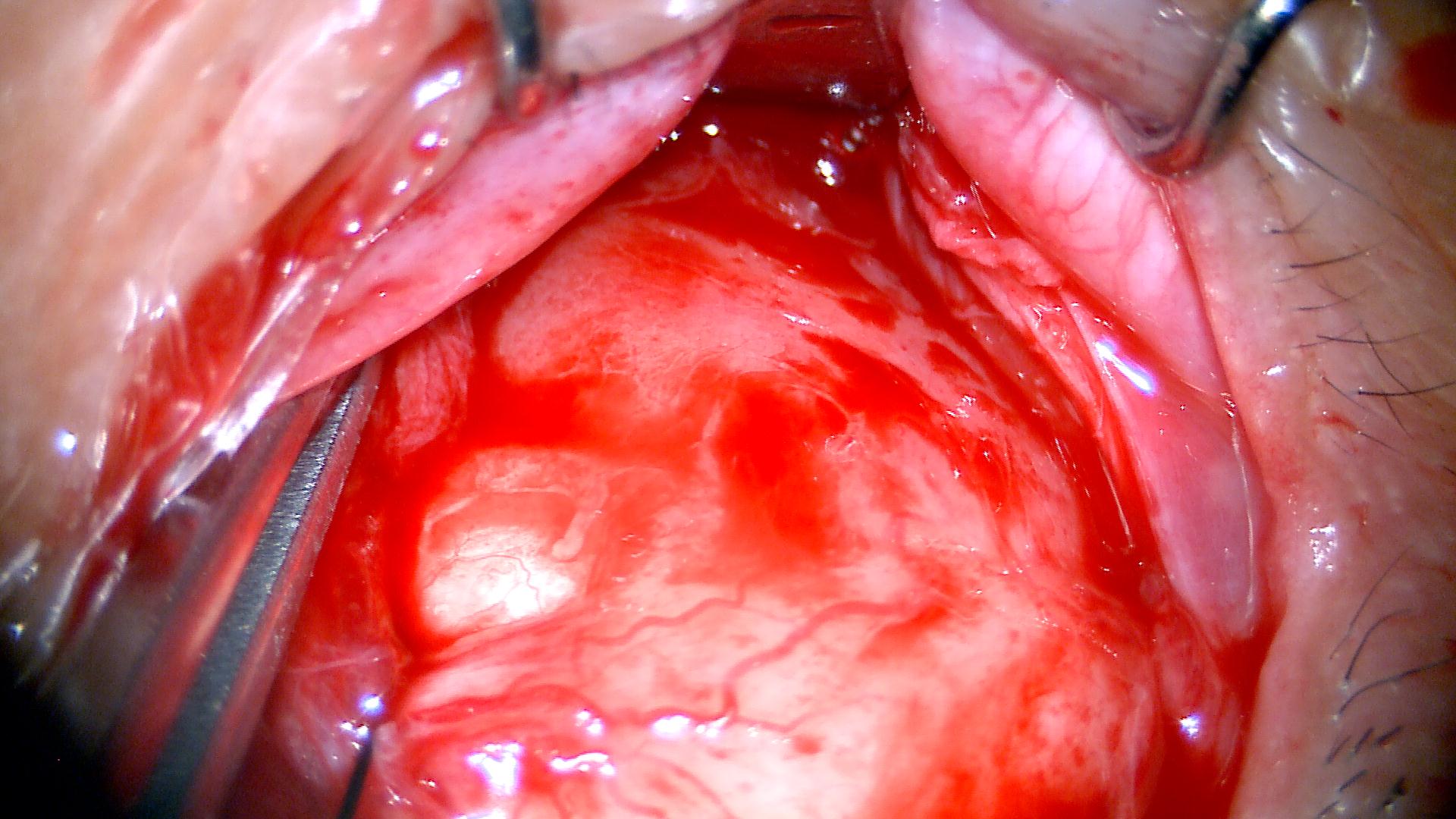

Supplement: Supplementary file 1 — Additional file 1: The raw data of this study. Table 1. The basic information of involved patients. [file 12886_2022_2598_MOESM1_ESM.zip › 3/μ£»Σ╕¡σñoΣ╜ôσâÅ/0123180345473.jpg]

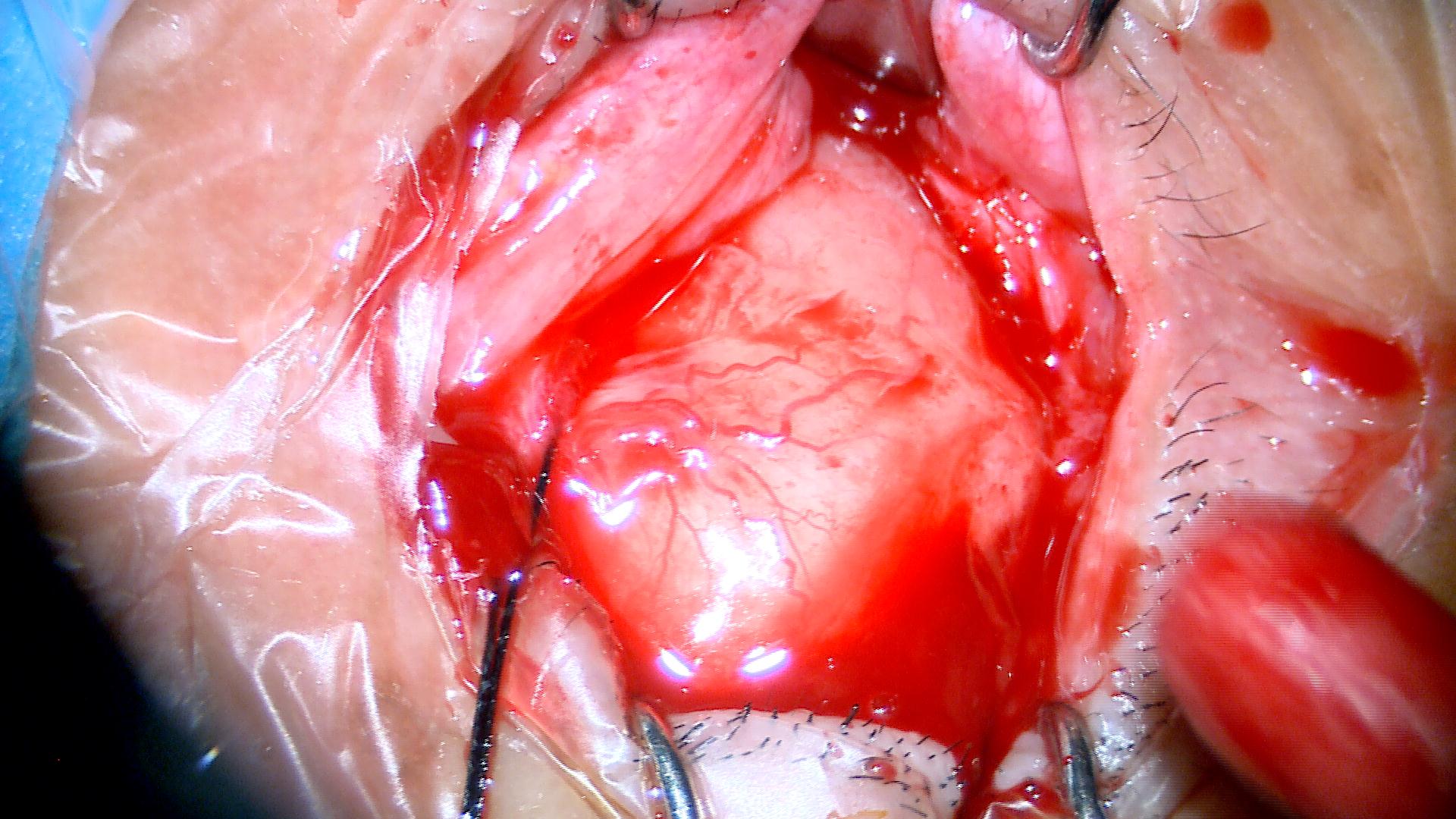

Supplement: Supplementary file 1 — Additional file 1: The raw data of this study. Table 1. The basic information of involved patients. [file 12886_2022_2598_MOESM1_ESM.zip › 3/μ£»Σ╕¡σñoΣ╜ôσâÅ/0123180227195.jpg]

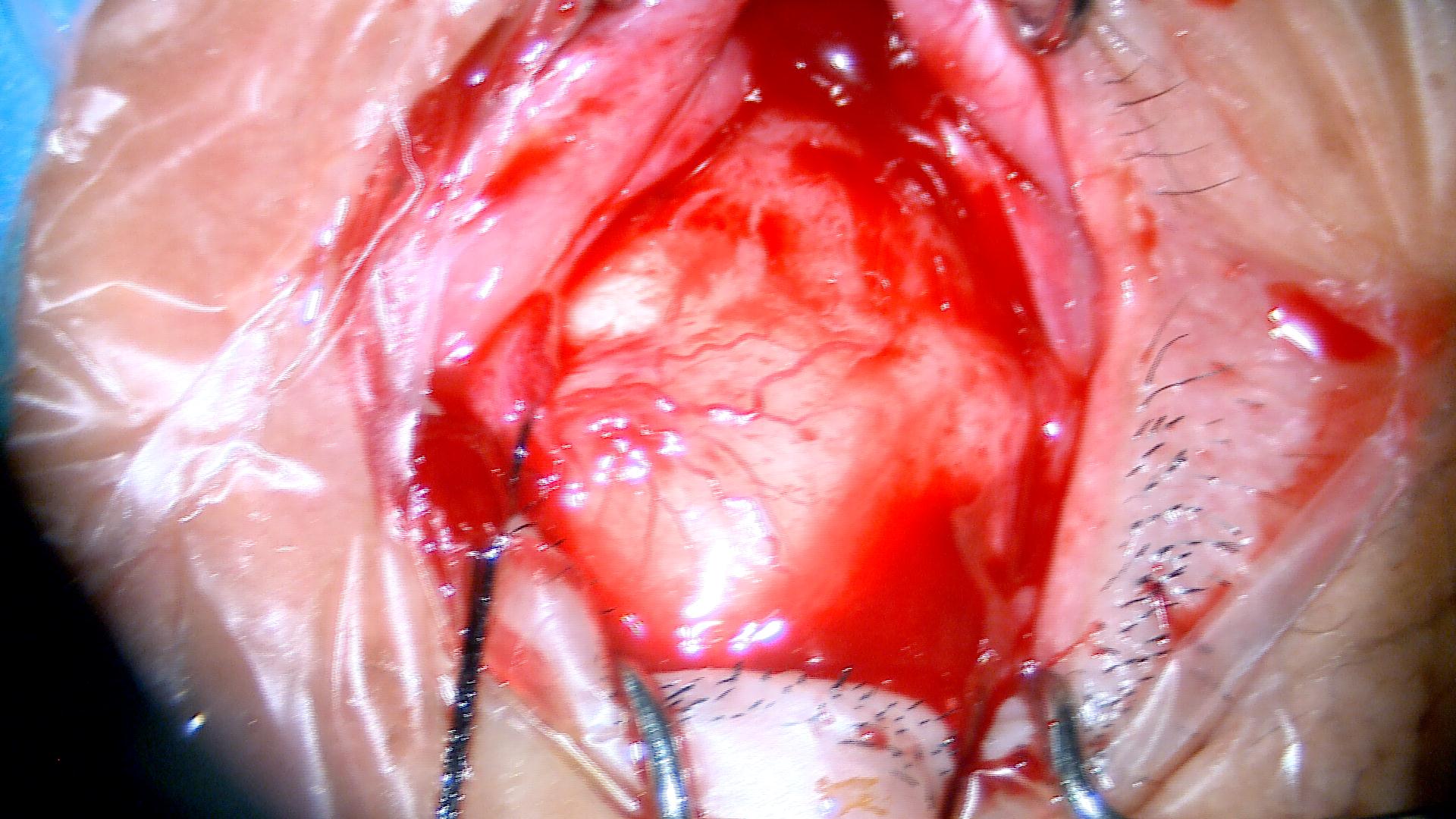

Supplement: Supplementary file 1 — Additional file 1: The raw data of this study. Table 1. The basic information of involved patients. [file 12886_2022_2598_MOESM1_ESM.zip › 3/μ£»Σ╕¡σñoΣ╜ôσâÅ/0123180249932.jpg]

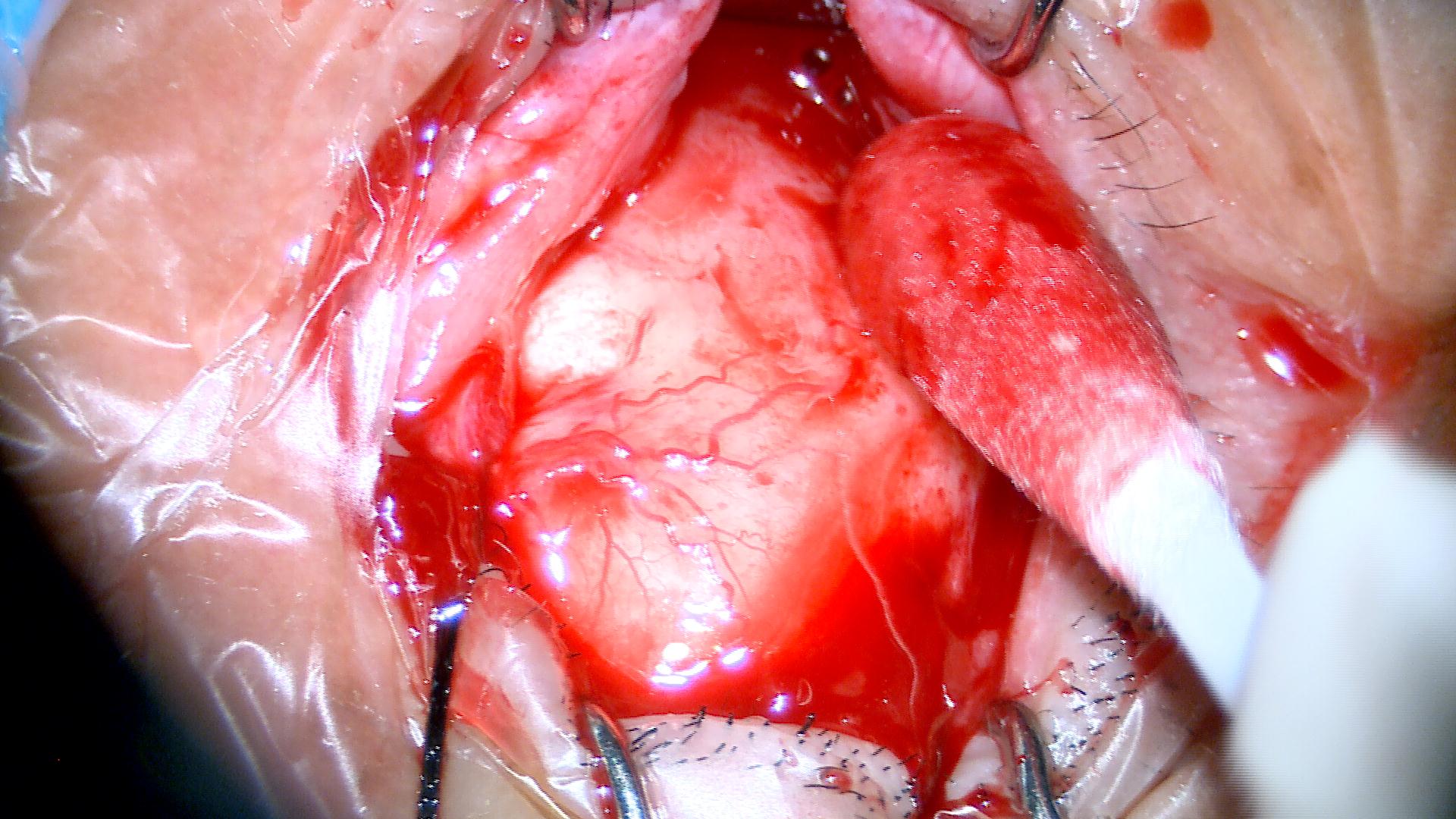

Supplement: Supplementary file 1 — Additional file 1: The raw data of this study. Table 1. The basic information of involved patients. [file 12886_2022_2598_MOESM1_ESM.zip › 3/μ£»Σ╕¡σñoΣ╜ôσâÅ/0123180238515.jpg]

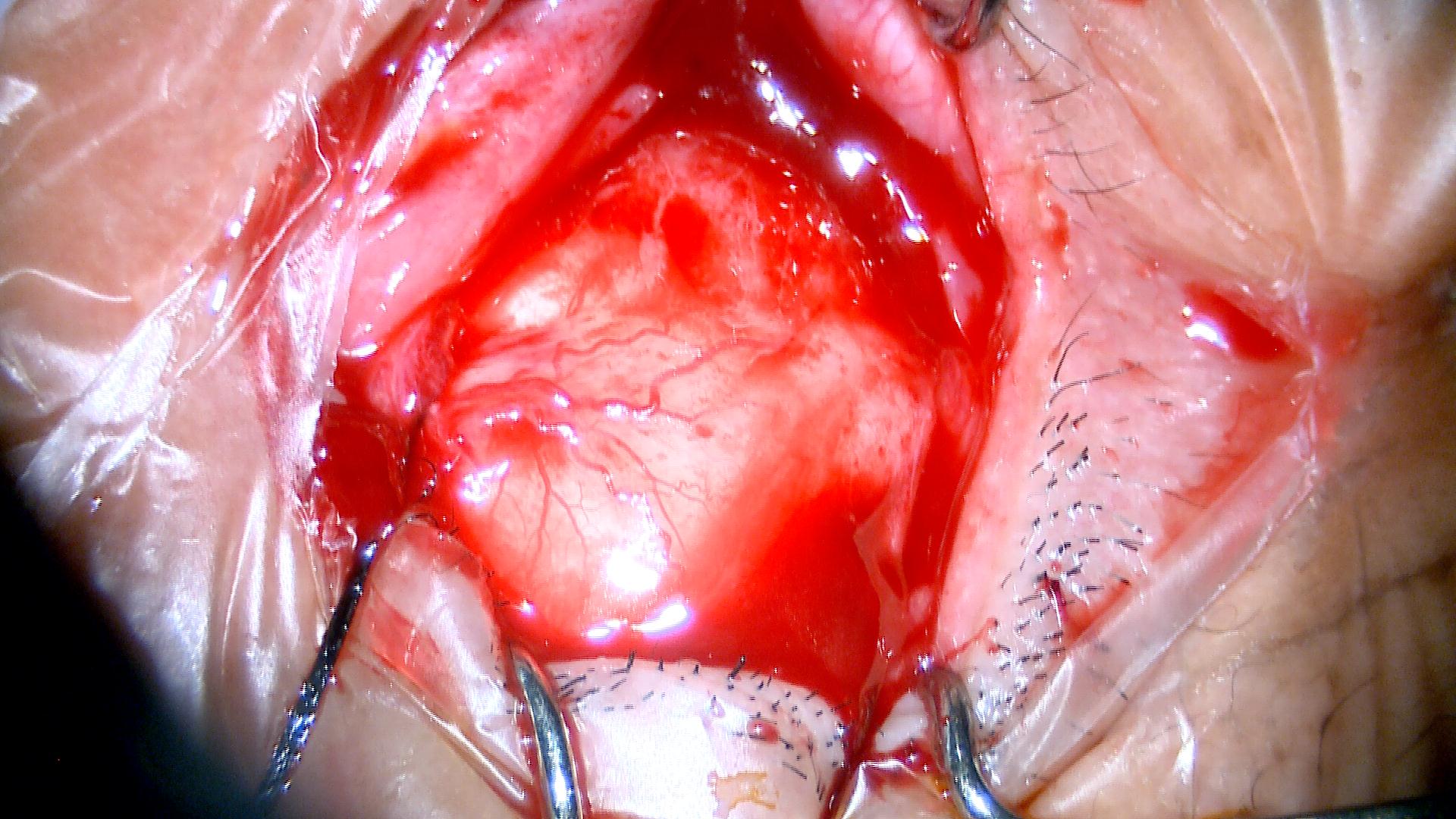

Supplement: Supplementary file 1 — Additional file 1: The raw data of this study. Table 1. The basic information of involved patients. [file 12886_2022_2598_MOESM1_ESM.zip › 3/μ£»Σ╕¡σñoΣ╜ôσâÅ/0123180254828.jpg]

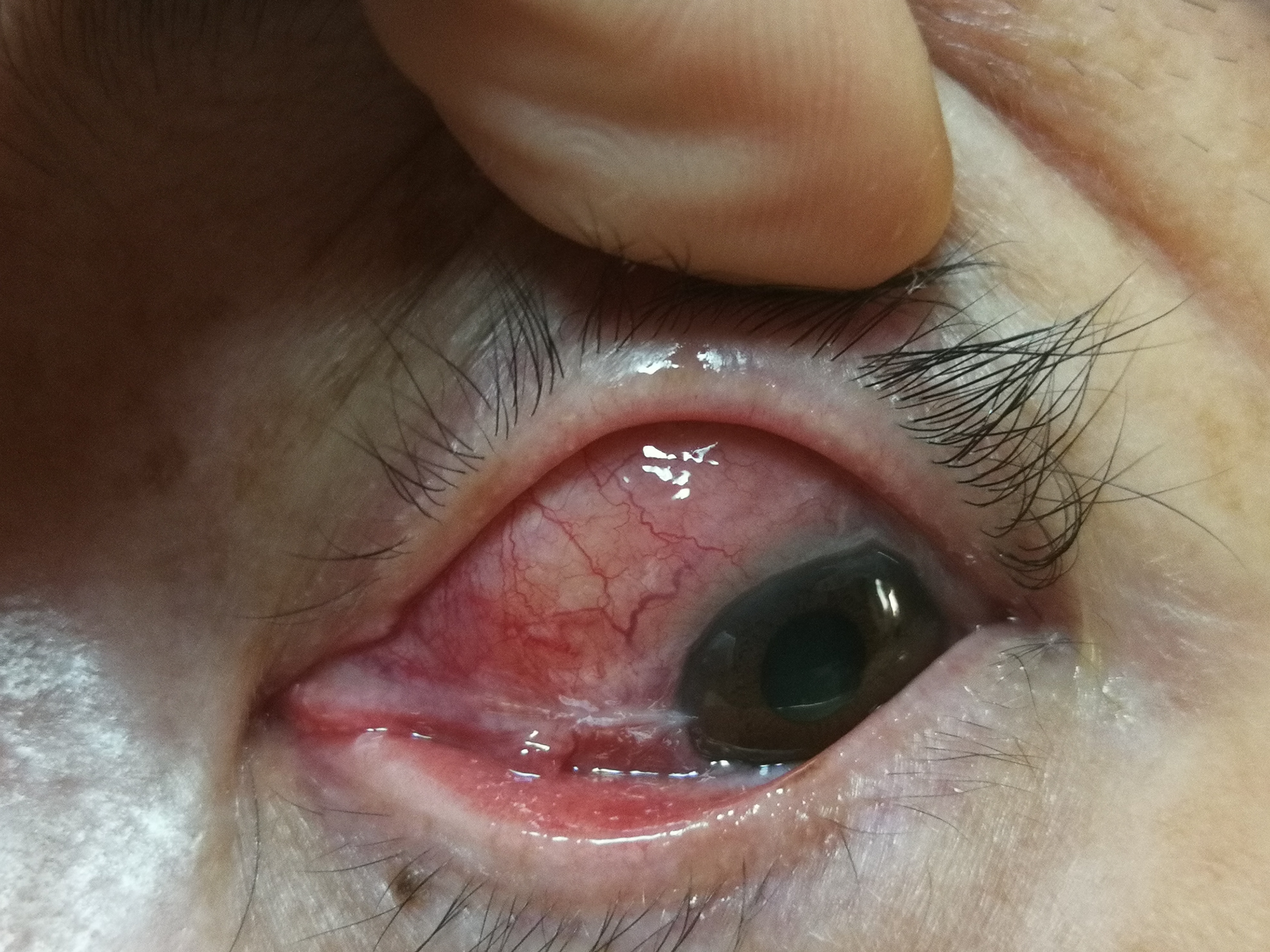

Supplement: Supplementary file 1 — Additional file 1: The raw data of this study. Table 1. The basic information of involved patients. [file 12886_2022_2598_MOESM1_ESM.zip › 1/σëìΦèéτàoτëç/681610003090_.pic_hd.jpg]

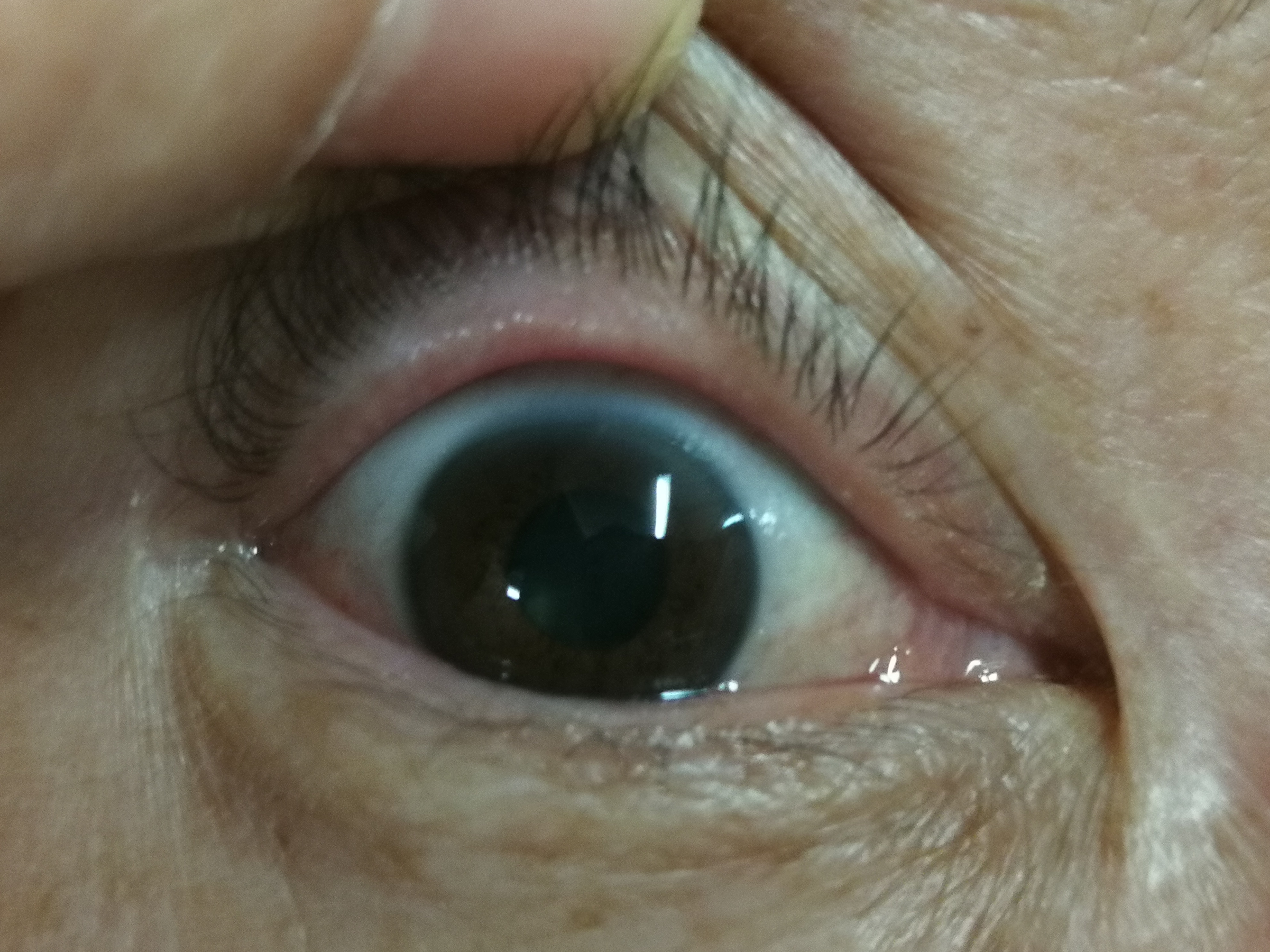

Supplement: Supplementary file 1 — Additional file 1: The raw data of this study. Table 1. The basic information of involved patients. [file 12886_2022_2598_MOESM1_ESM.zip › 1/σëìΦèéτàoτëç/691610003094_.pic_hd.jpg]

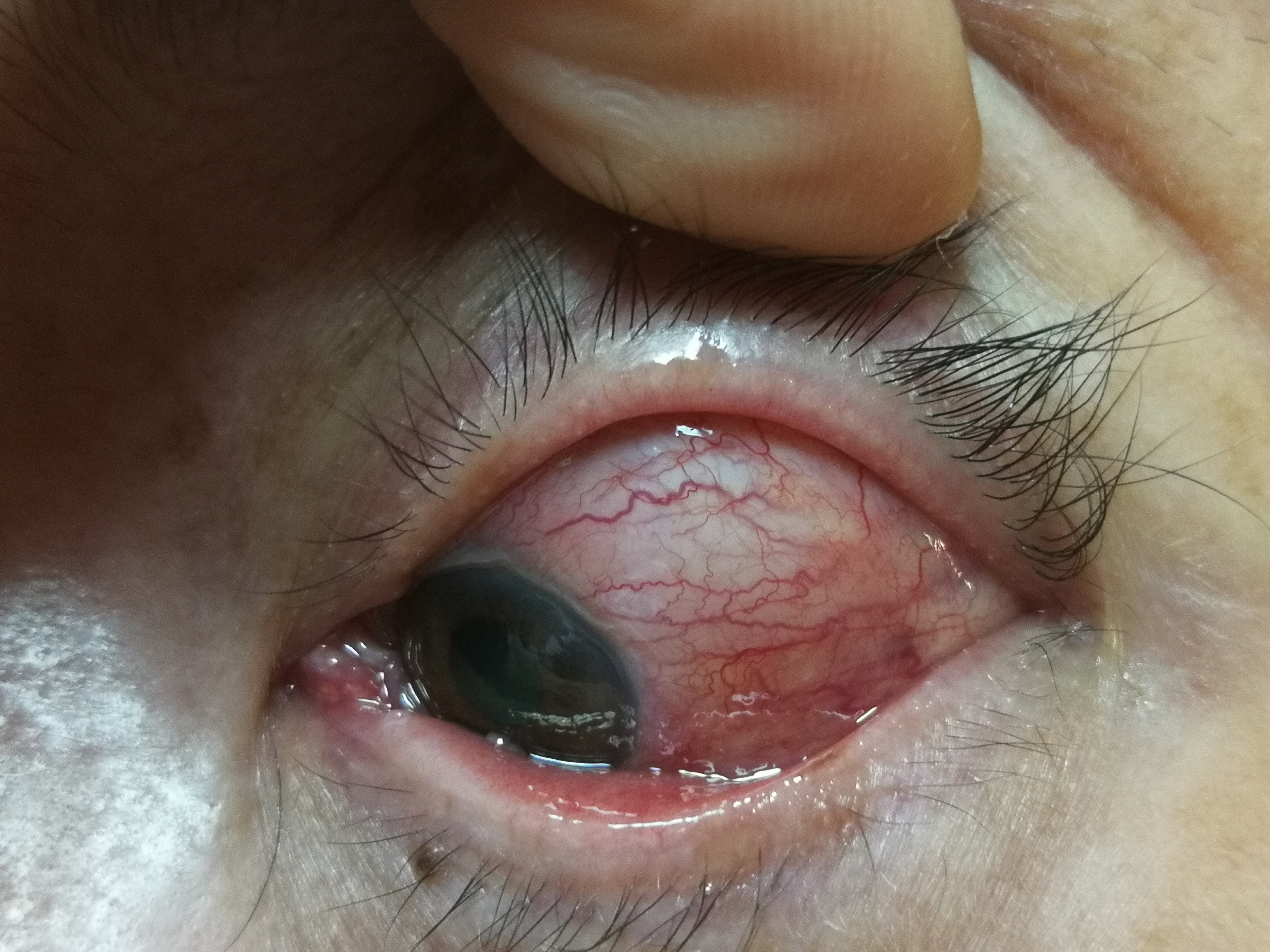

Supplement: Supplementary file 1 — Additional file 1: The raw data of this study. Table 1. The basic information of involved patients. [file 12886_2022_2598_MOESM1_ESM.zip › 1/σëìΦèéτàoτëç/661610003078_.pic_hd.jpg]

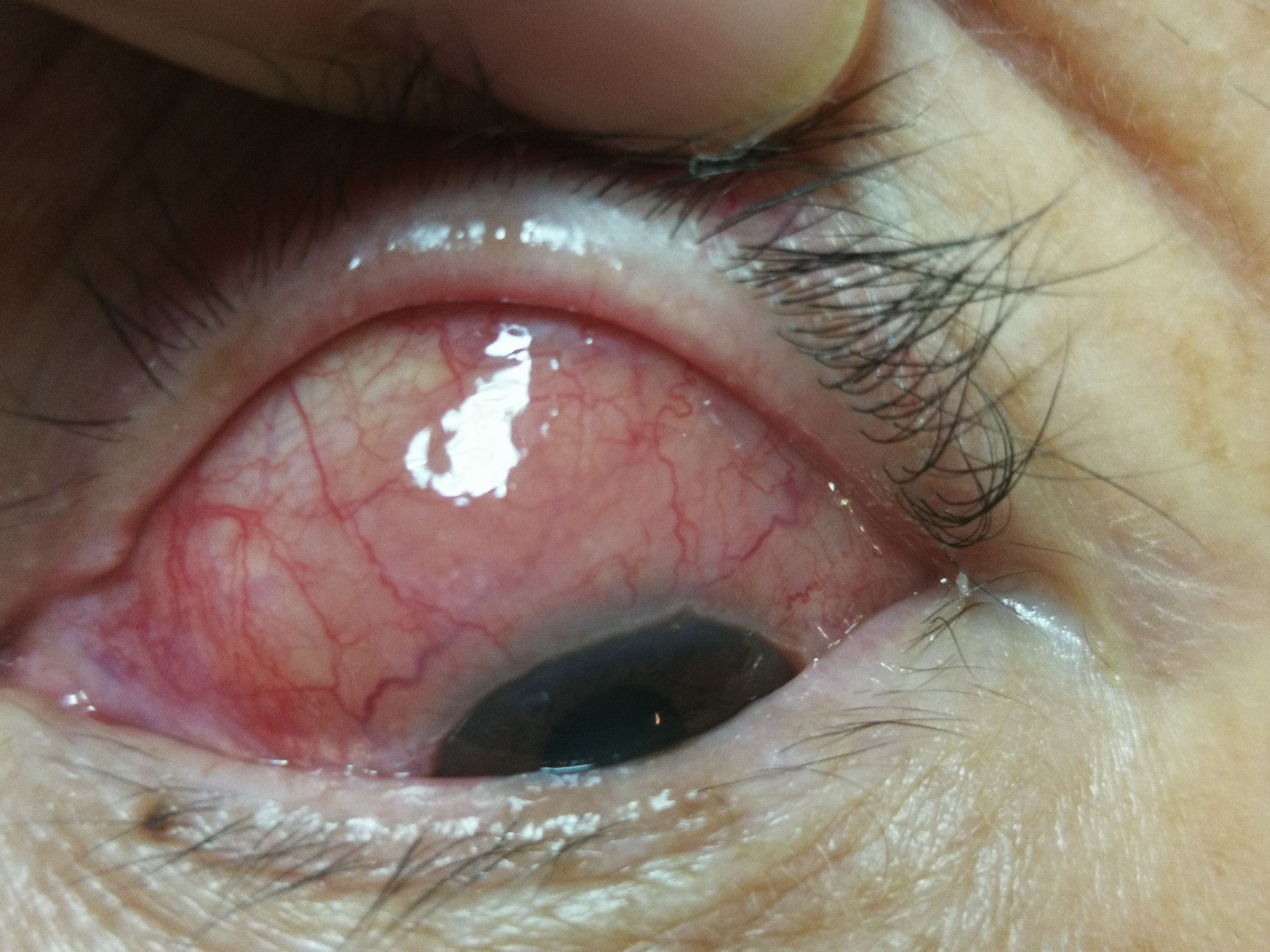

Supplement: Supplementary file 1 — Additional file 1: The raw data of this study. Table 1. The basic information of involved patients. [file 12886_2022_2598_MOESM1_ESM.zip › 1/σëìΦèéτàoτëç/671610003086_.pic_hd.jpg]

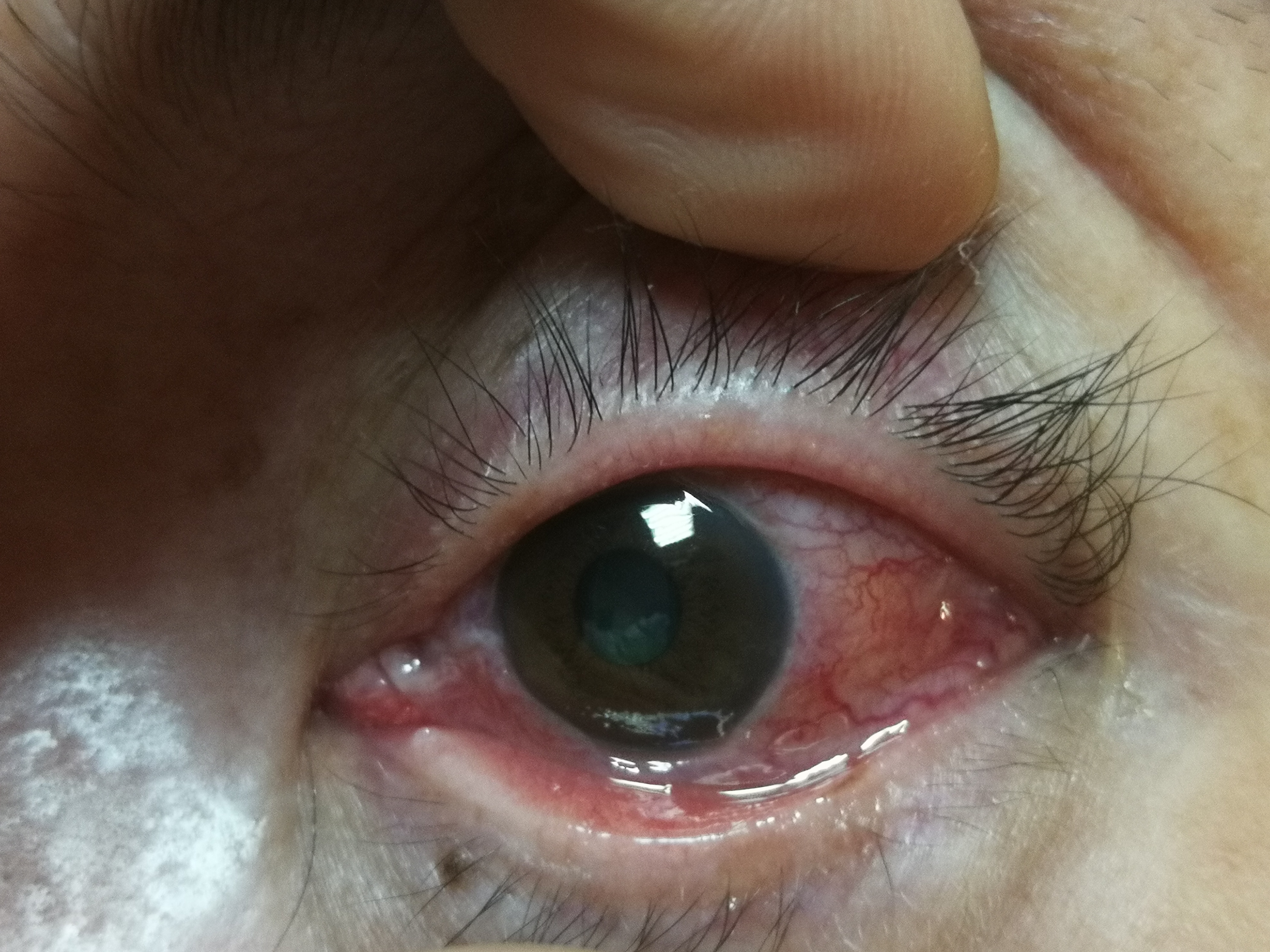

Supplement: Supplementary file 1 — Additional file 1: The raw data of this study. Table 1. The basic information of involved patients. [file 12886_2022_2598_MOESM1_ESM.zip › 1/σëìΦèéτàoτëç/651610003074_.pic_hd.jpg]

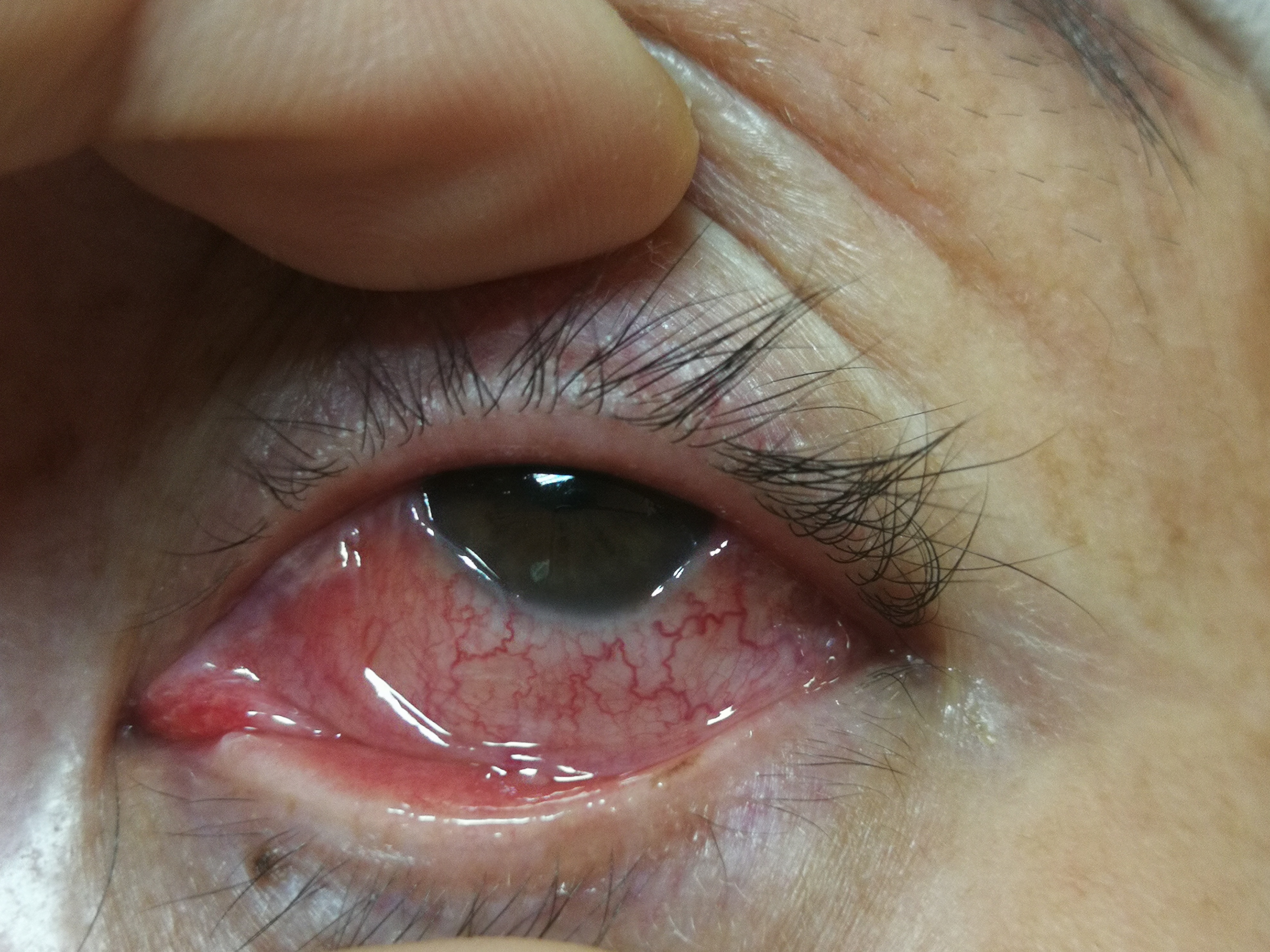

Supplement: Supplementary file 1 — Additional file 1: The raw data of this study. Table 1. The basic information of involved patients. [file 12886_2022_2598_MOESM1_ESM.zip › 1/σëìΦèéτàoτëç/641610003070_.pic_hd.jpg]

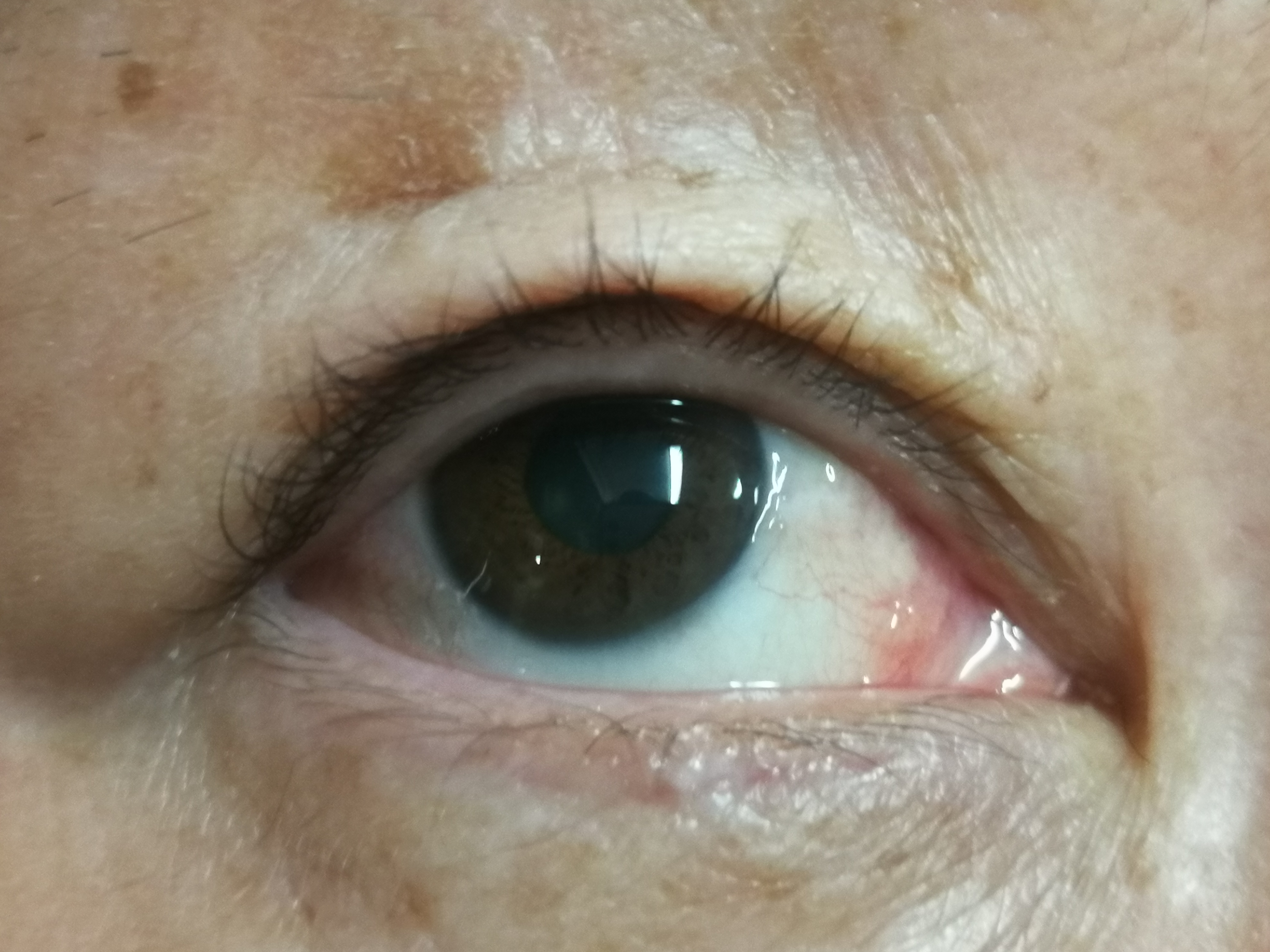

Supplement: Supplementary file 1 — Additional file 1: The raw data of this study. Table 1. The basic information of involved patients. [file 12886_2022_2598_MOESM1_ESM.zip › 1/σëìΦèéτàoτëç/701610003098_.pic_hd.jpg]

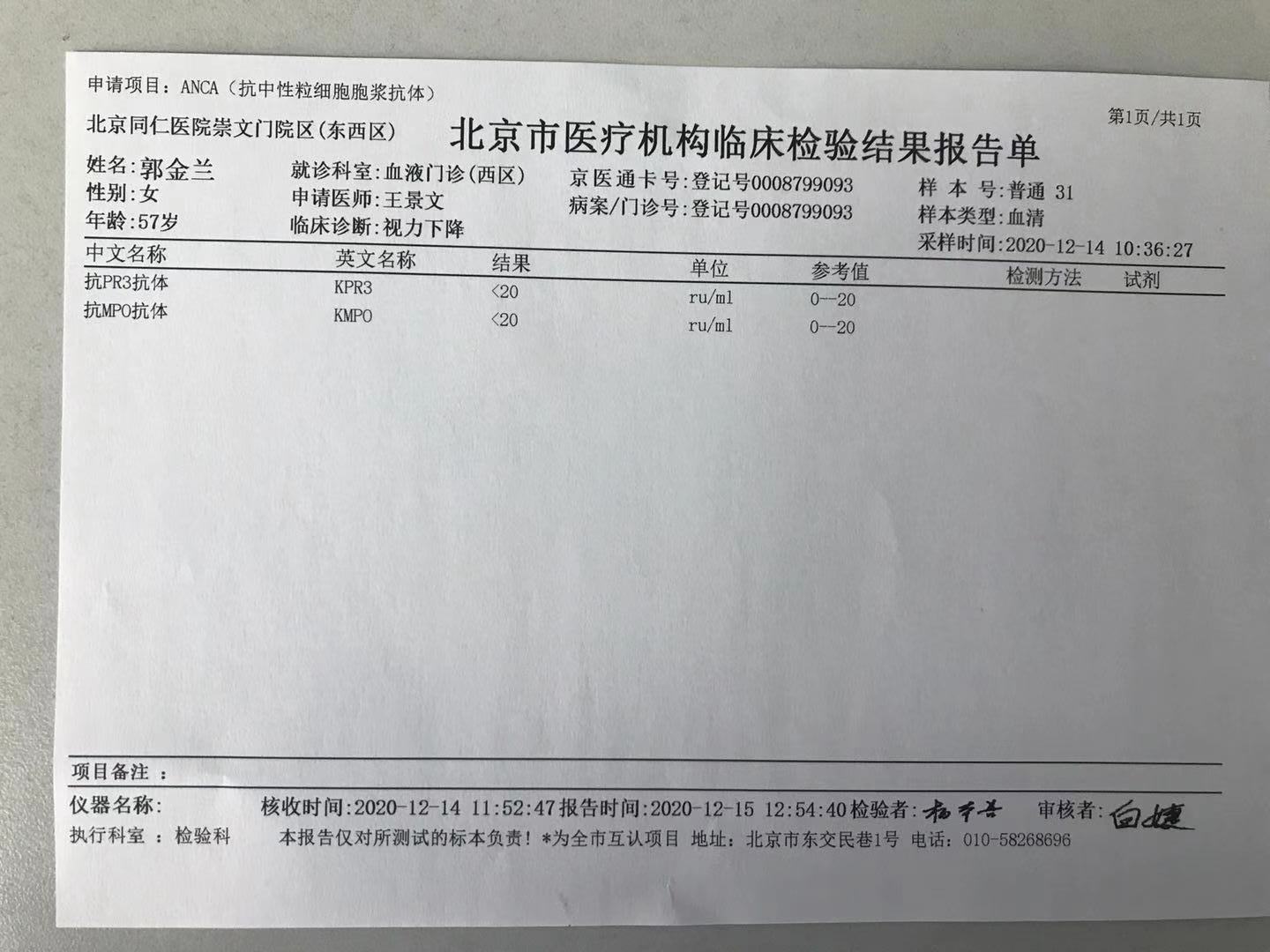

Supplement: Supplementary file 1 — Additional file 1: The raw data of this study. Table 1. The basic information of involved patients. [file 12886_2022_2598_MOESM1_ESM.zip › 1/σîûΘ¬î/20201215ANCA.jpg]

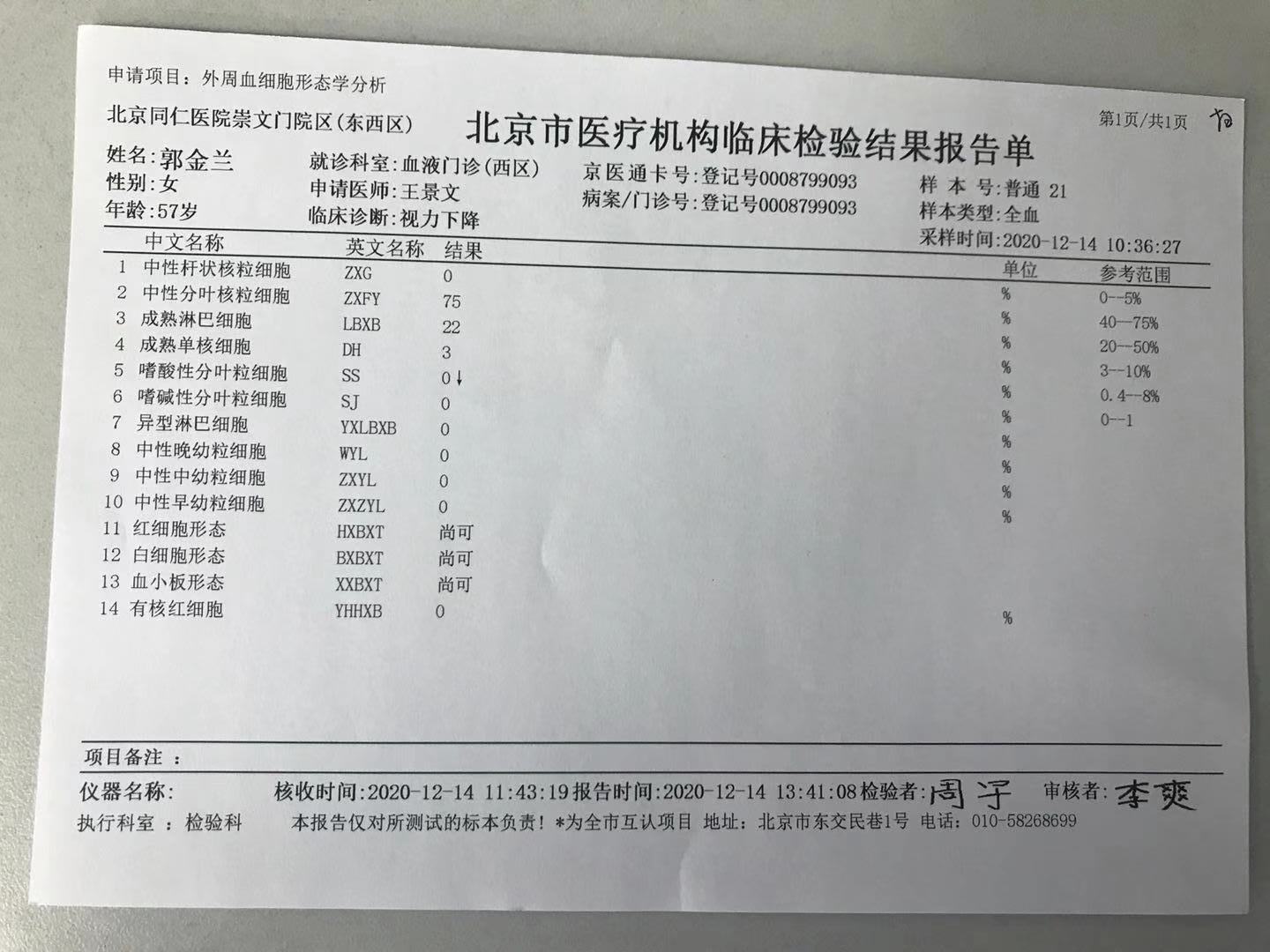

Supplement: Supplementary file 1 — Additional file 1: The raw data of this study. Table 1. The basic information of involved patients. [file 12886_2022_2598_MOESM1_ESM.zip › 1/σîûΘ¬î/20201214ΦíÇσ╕╕Φoä.jpg]

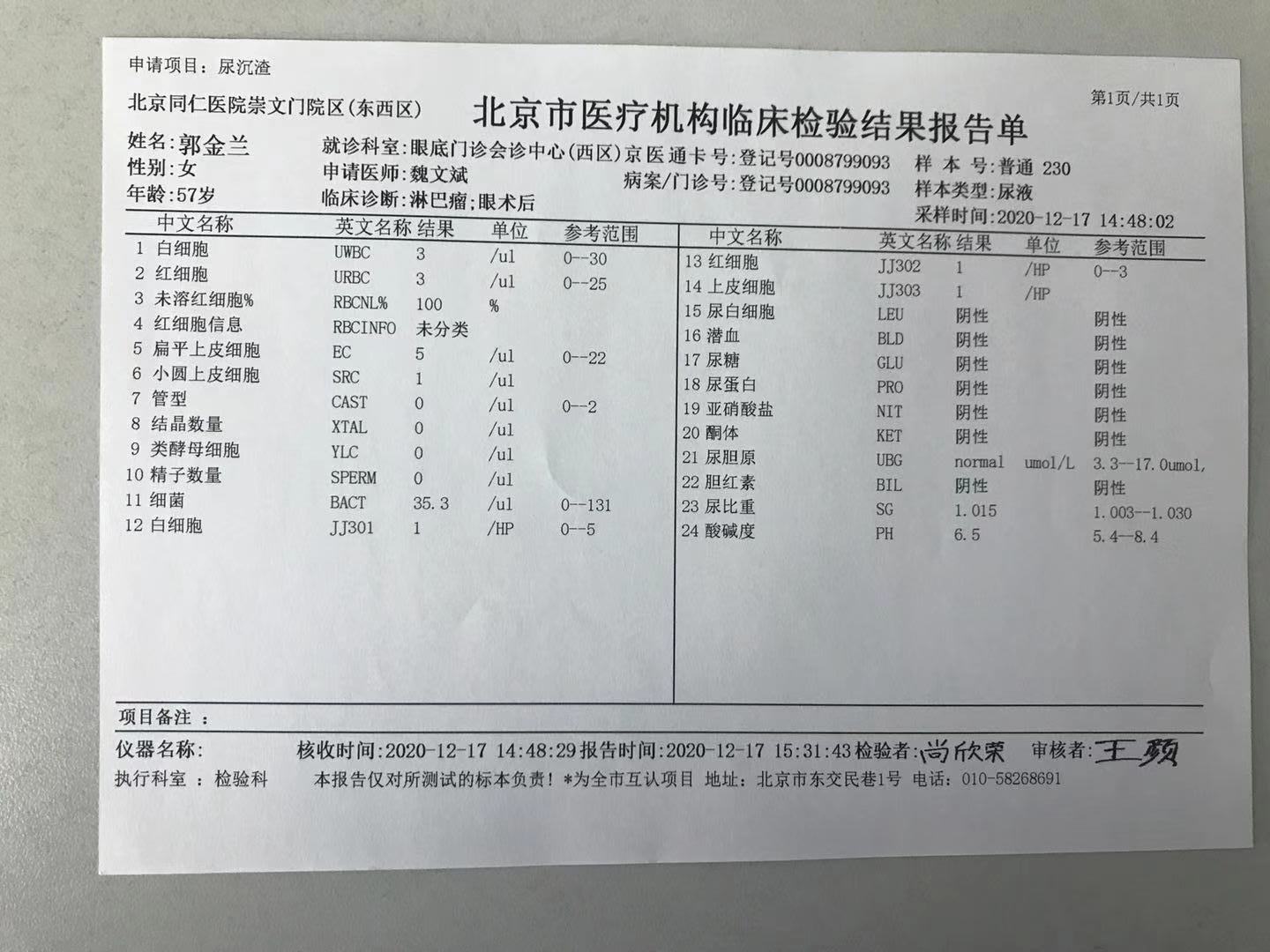

Supplement: Supplementary file 1 — Additional file 1: The raw data of this study. Table 1. The basic information of involved patients. [file 12886_2022_2598_MOESM1_ESM.zip › 1/σîûΘ¬î/20201217σ░┐σ╕╕Φoä.jpg]

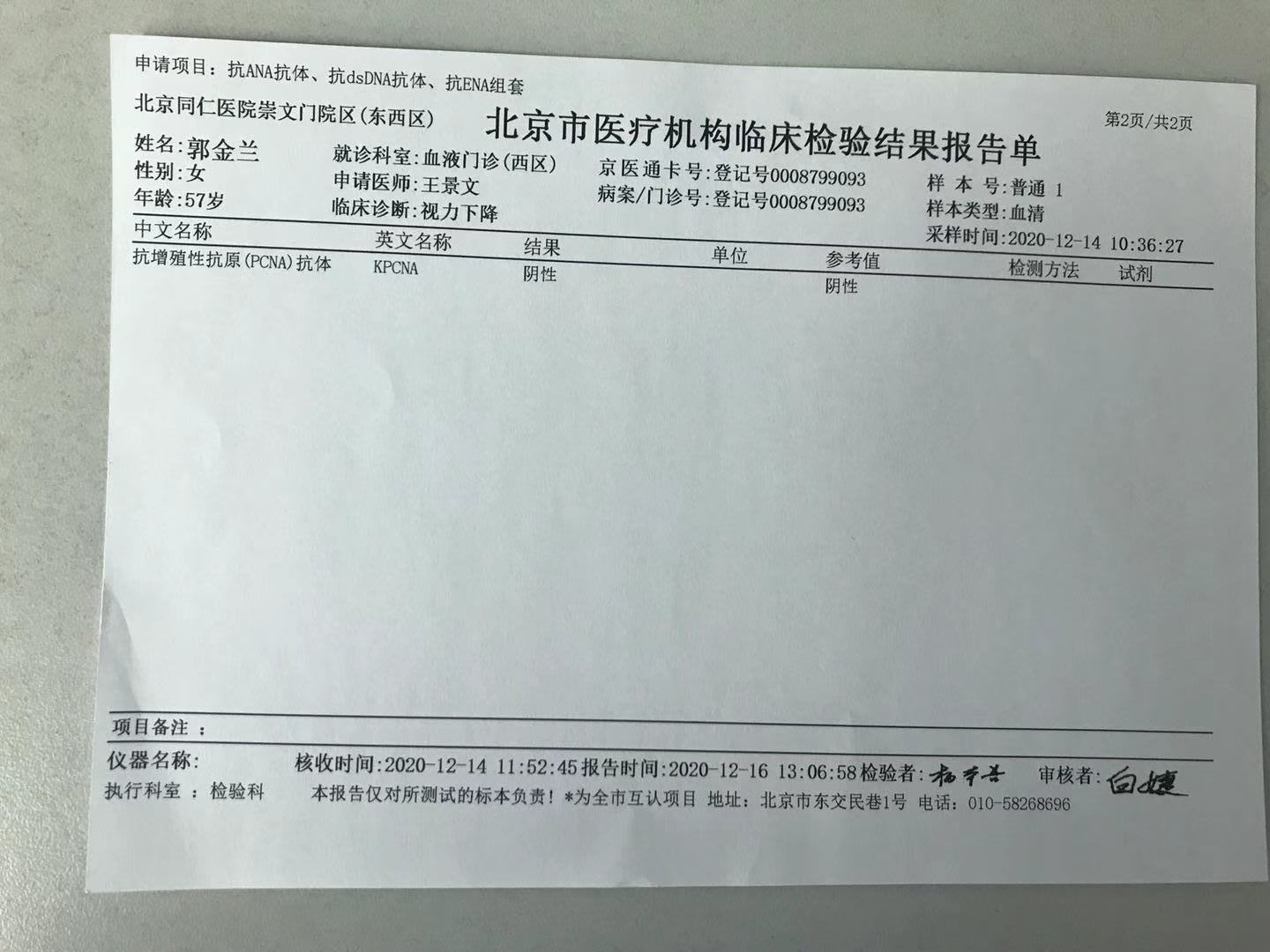

Supplement: Supplementary file 1 — Additional file 1: The raw data of this study. Table 1. The basic information of involved patients. [file 12886_2022_2598_MOESM1_ESM.zip › 1/σîûΘ¬î/20201216PCNA.jpg]

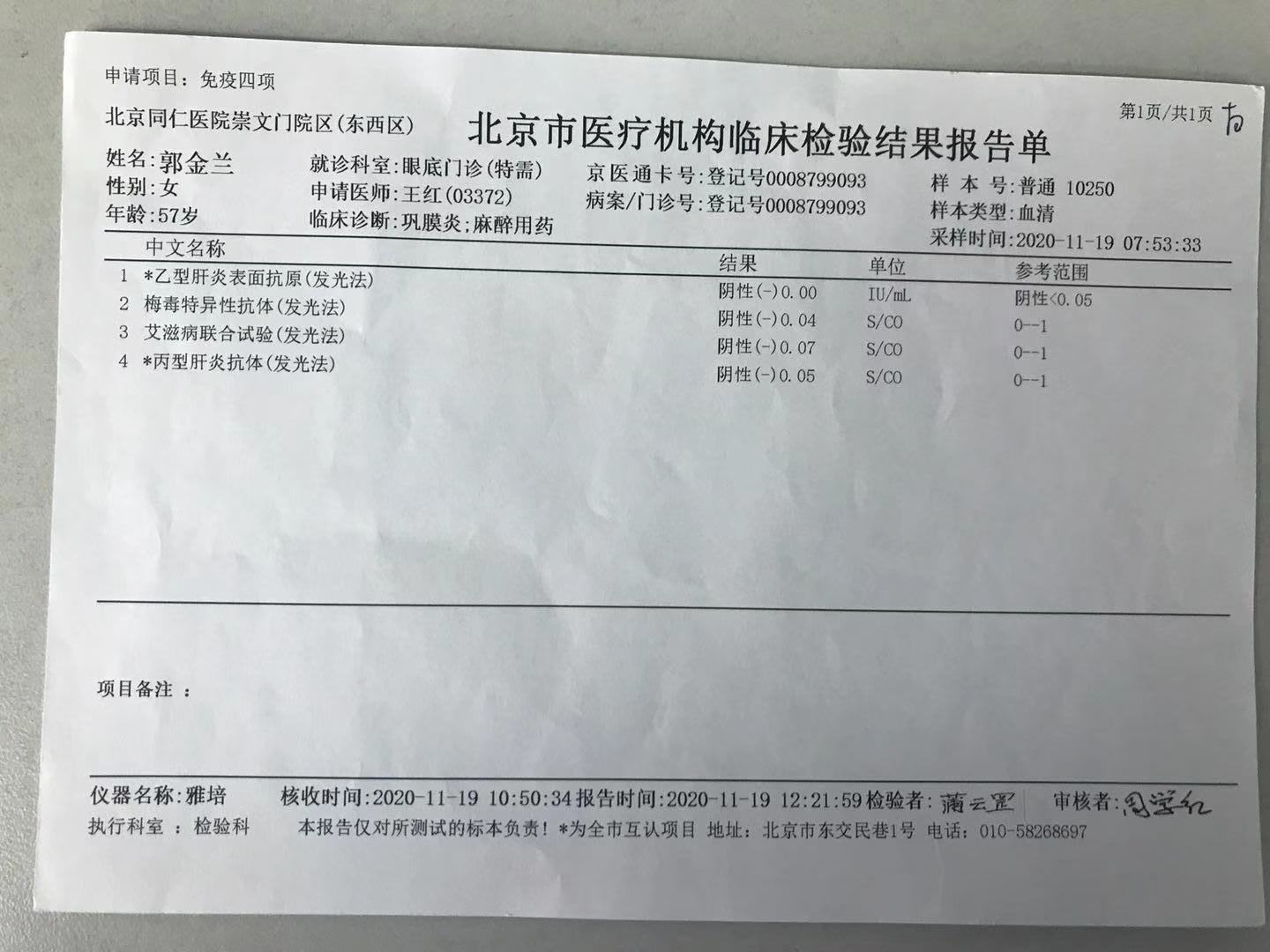

Supplement: Supplementary file 1 — Additional file 1: The raw data of this study. Table 1. The basic information of involved patients. [file 12886_2022_2598_MOESM1_ESM.zip › 1/σîûΘ¬î/20201119σàìτû1⁄2σ¢¢Θí╣.jpg]

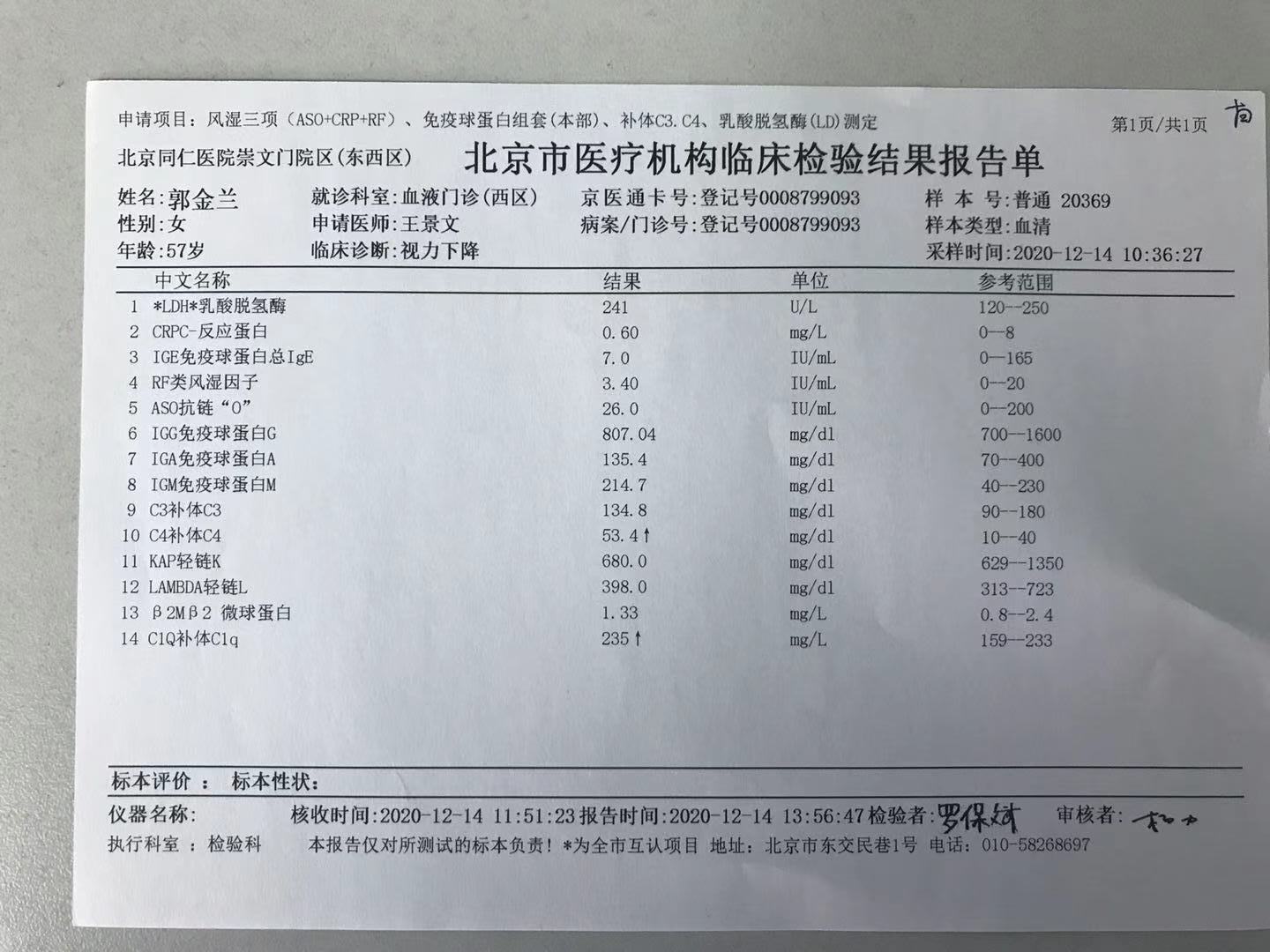

Supplement: Supplementary file 1 — Additional file 1: The raw data of this study. Table 1. The basic information of involved patients. [file 12886_2022_2598_MOESM1_ESM.zip › 1/σîûΘ¬î/20201214ΘúÄμ╣┐Σ╕ëΘí╣+σàìτû1⁄2τÉâΦ¢ïτÖ╜+Σ╣│Θà╕Φä▒μ░óΘà╢.jpg]

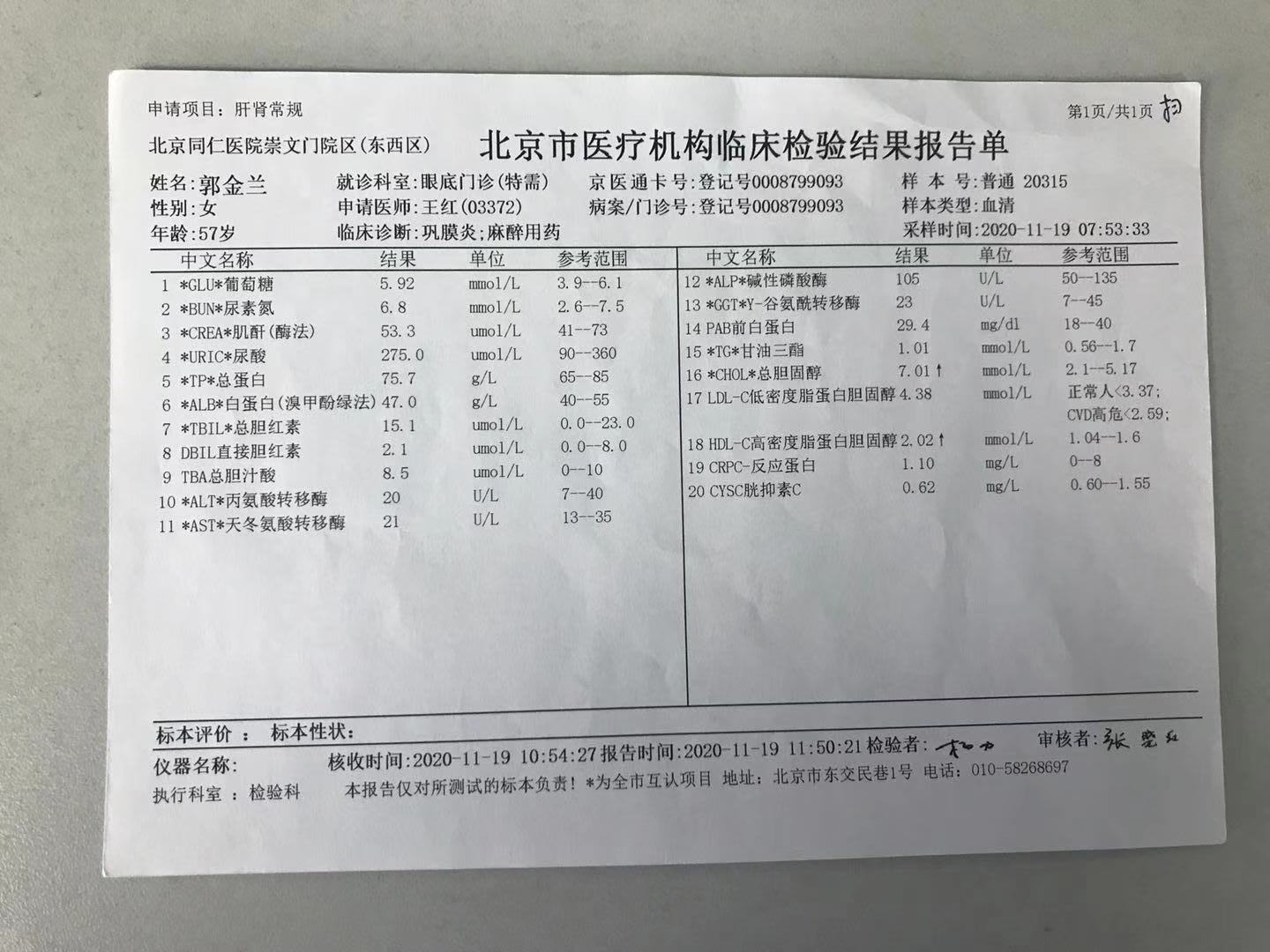

Supplement: Supplementary file 1 — Additional file 1: The raw data of this study. Table 1. The basic information of involved patients. [file 12886_2022_2598_MOESM1_ESM.zip › 1/σîûΘ¬î/20201119Φé¥Φé╛σ╕╕Φoä.jpg]

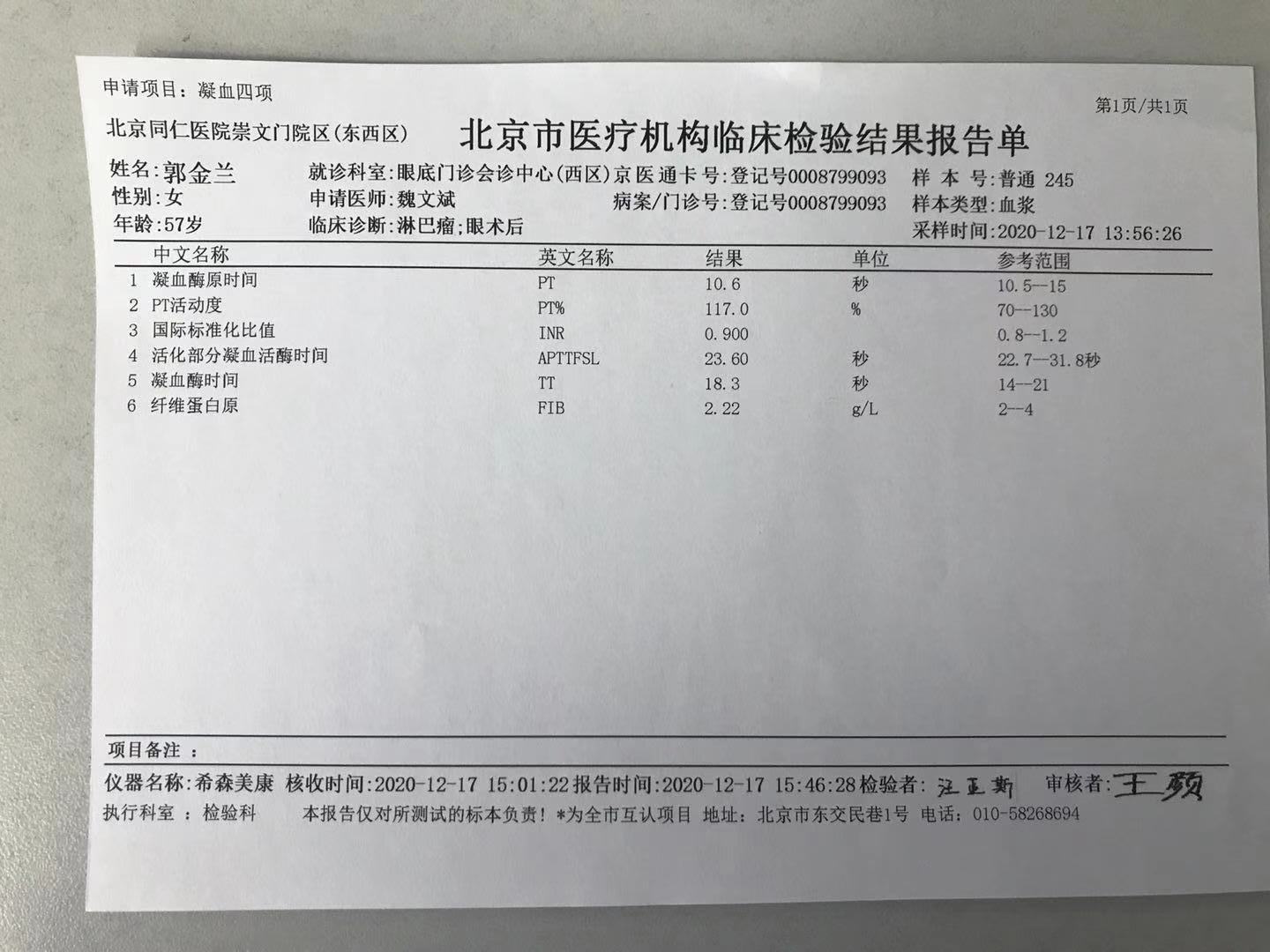

Supplement: Supplementary file 1 — Additional file 1: The raw data of this study. Table 1. The basic information of involved patients. [file 12886_2022_2598_MOESM1_ESM.zip › 1/σîûΘ¬î/20201217σç¥ΦíÇ.jpg]

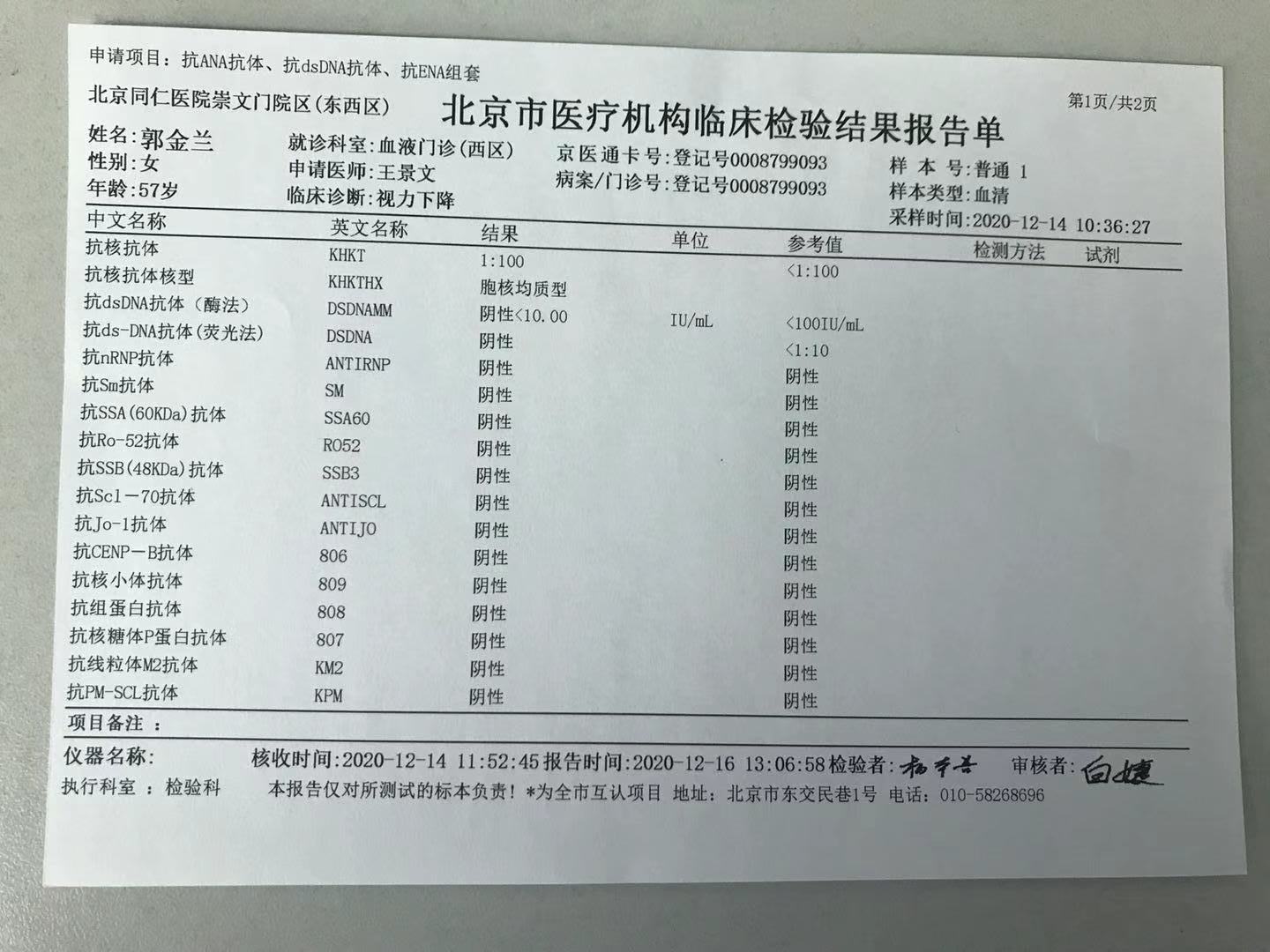

Supplement: Supplementary file 1 — Additional file 1: The raw data of this study. Table 1. The basic information of involved patients. [file 12886_2022_2598_MOESM1_ESM.zip › 1/σîûΘ¬î/20201216ANA+dsDNA+ENA.jpg]

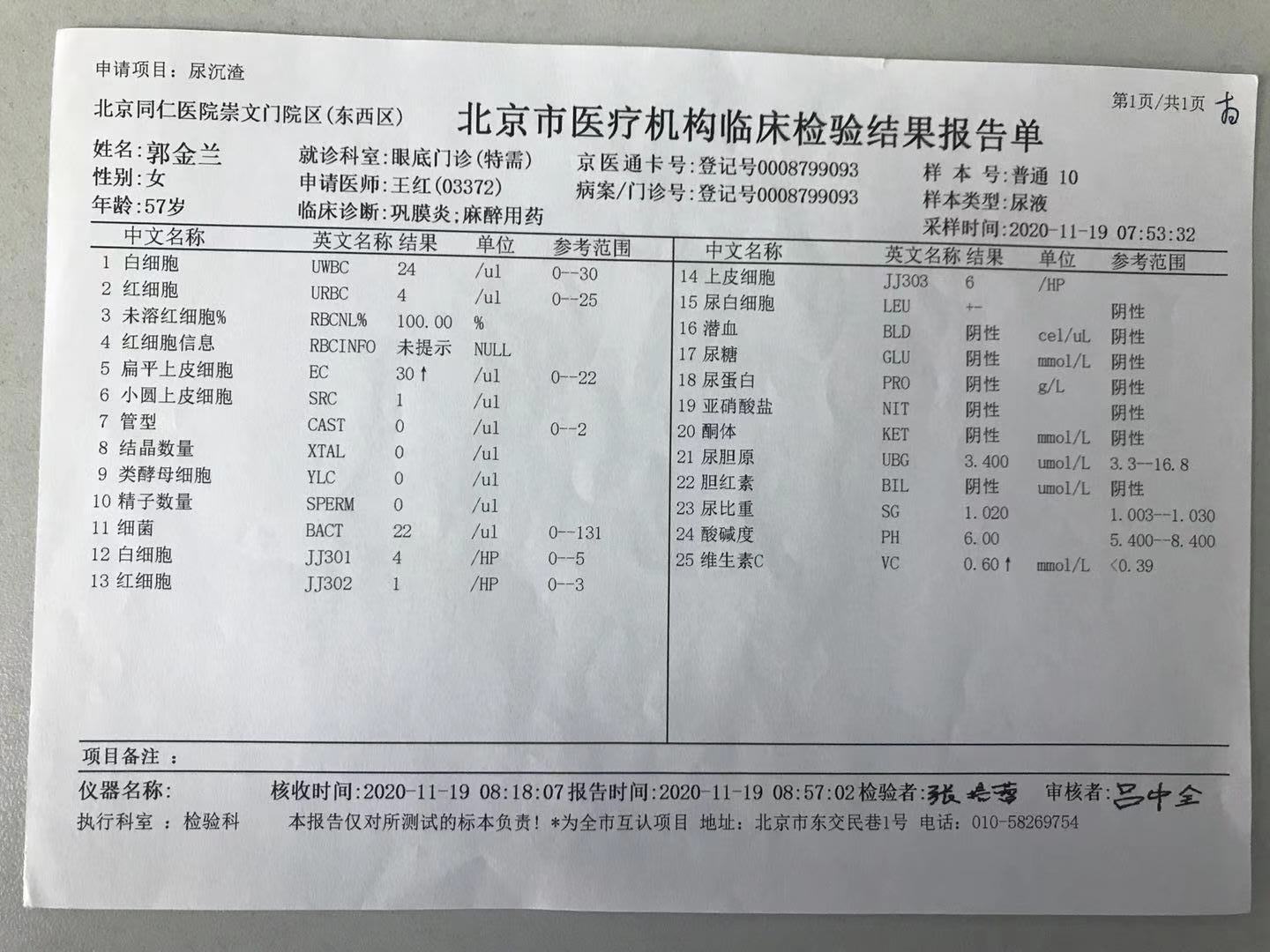

Supplement: Supplementary file 1 — Additional file 1: The raw data of this study. Table 1. The basic information of involved patients. [file 12886_2022_2598_MOESM1_ESM.zip › 1/σîûΘ¬î/20201119σ░┐σ╕╕Φoä.jpg]

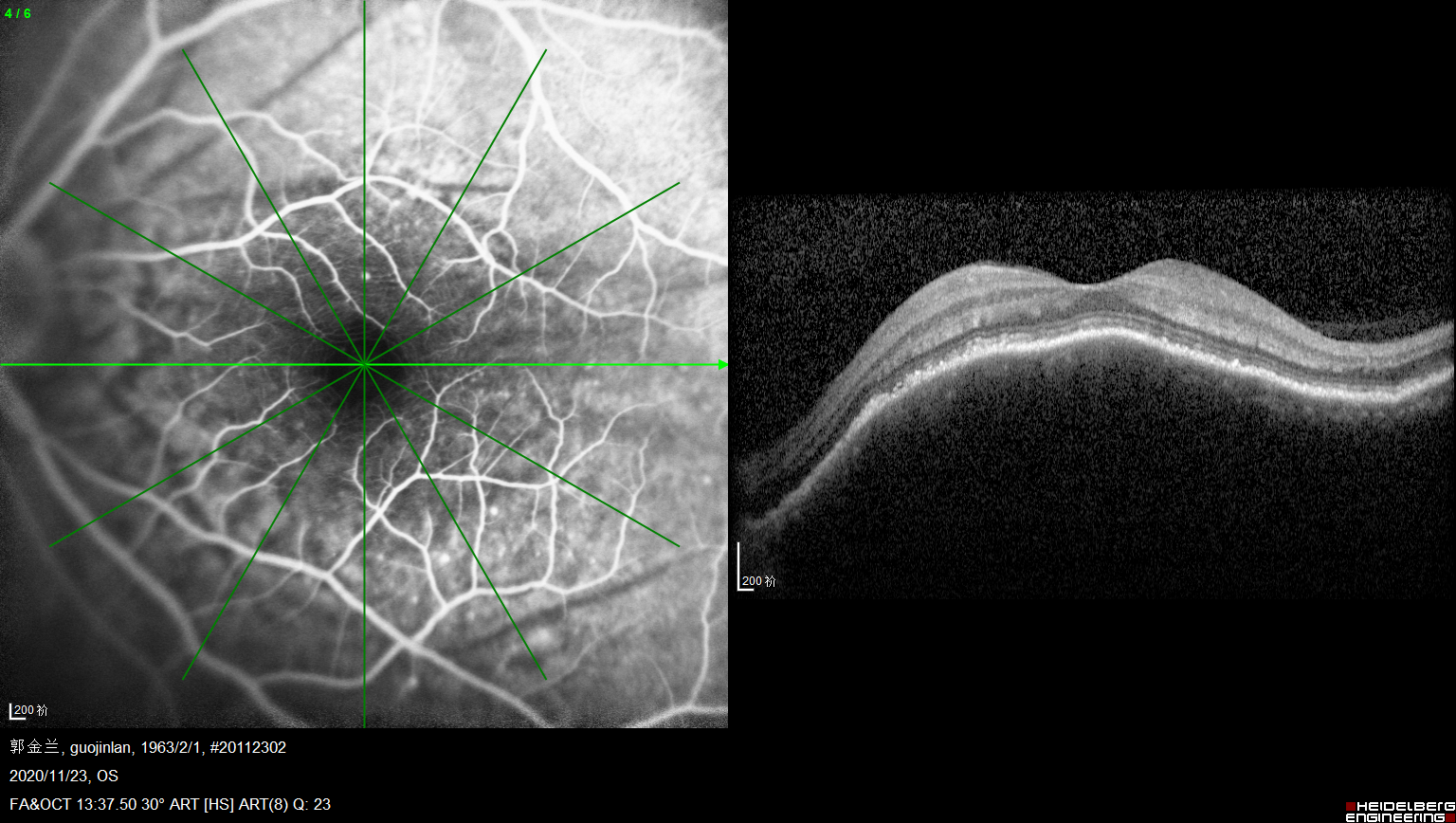

Supplement: Supplementary file 1 — Additional file 1: The raw data of this study. Table 1. The basic information of involved patients. [file 12886_2022_2598_MOESM1_ESM.zip › 1/Θâ¡Θçæσà░maltFFA/Θâ¡Θçæσà░g_012003.tif]

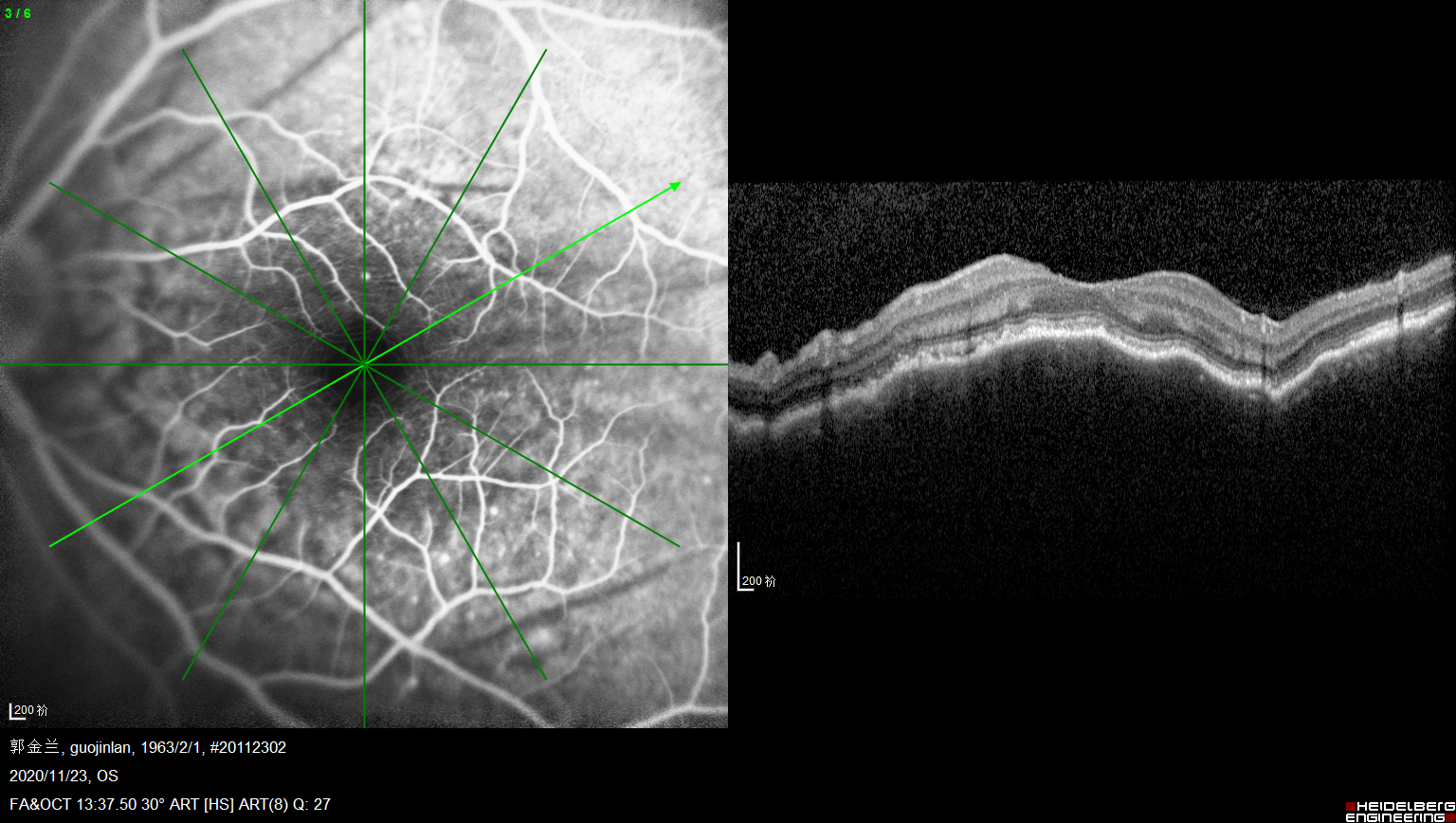

Supplement: Supplementary file 1 — Additional file 1: The raw data of this study. Table 1. The basic information of involved patients. [file 12886_2022_2598_MOESM1_ESM.zip › 1/Θâ¡Θçæσà░maltFFA/Θâ¡Θçæσà░g_012002.tif]

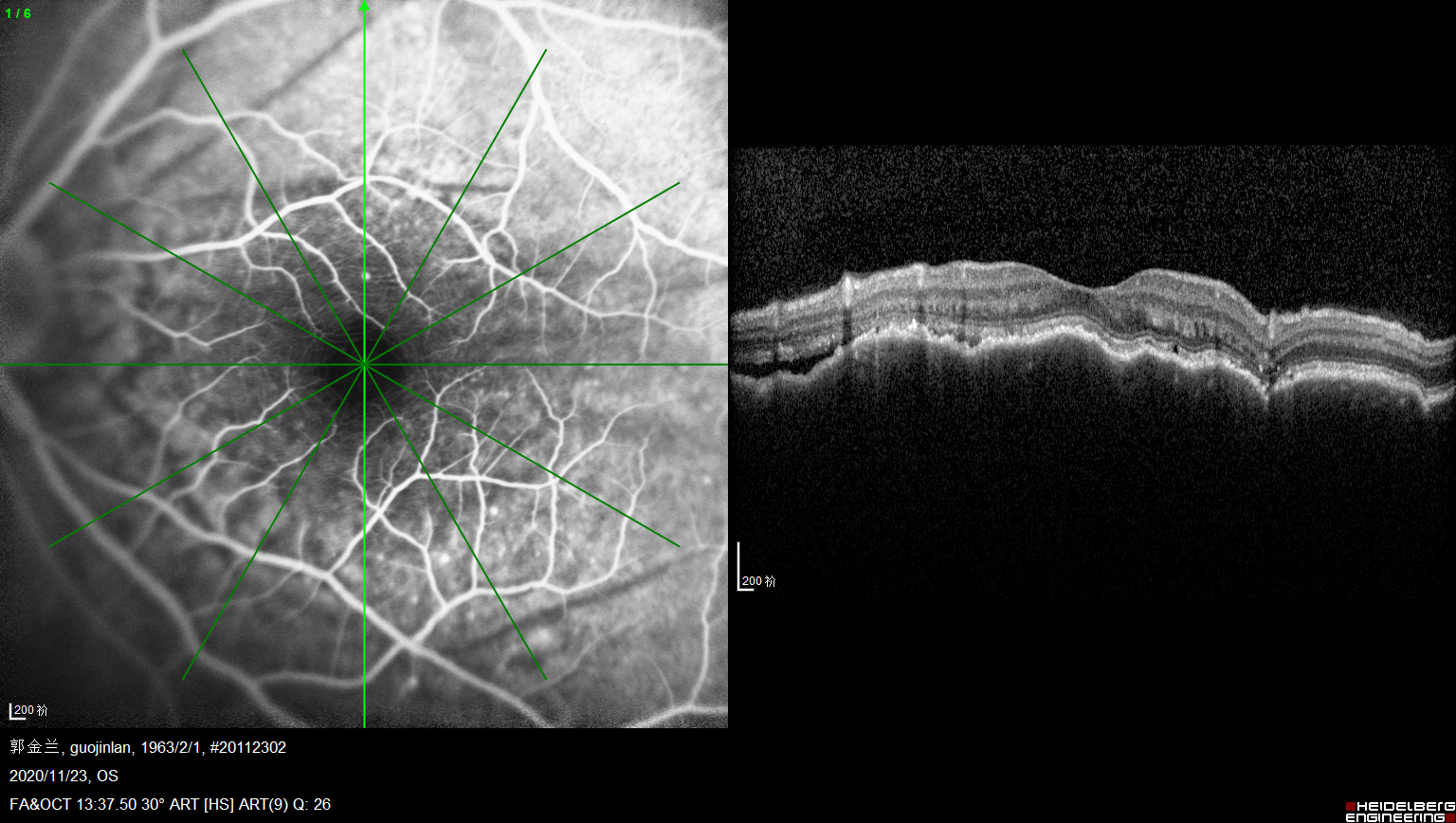

Supplement: Supplementary file 1 — Additional file 1: The raw data of this study. Table 1. The basic information of involved patients. [file 12886_2022_2598_MOESM1_ESM.zip › 1/Θâ¡Θçæσà░maltFFA/Θâ¡Θçæσà░g_012000.tif]

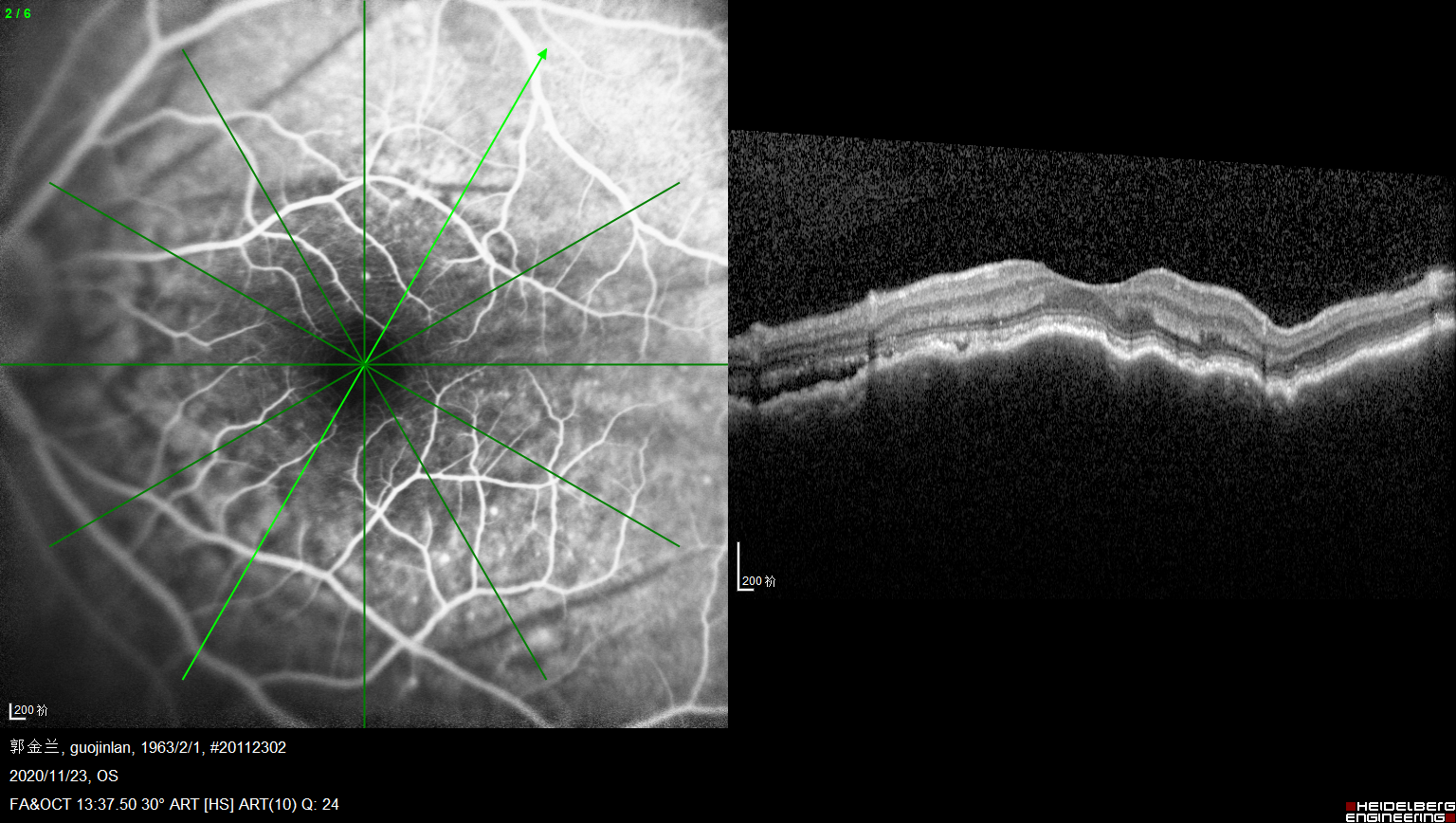

Supplement: Supplementary file 1 — Additional file 1: The raw data of this study. Table 1. The basic information of involved patients. [file 12886_2022_2598_MOESM1_ESM.zip › 1/Θâ¡Θçæσà░maltFFA/Θâ¡Θçæσà░g_012001.tif]

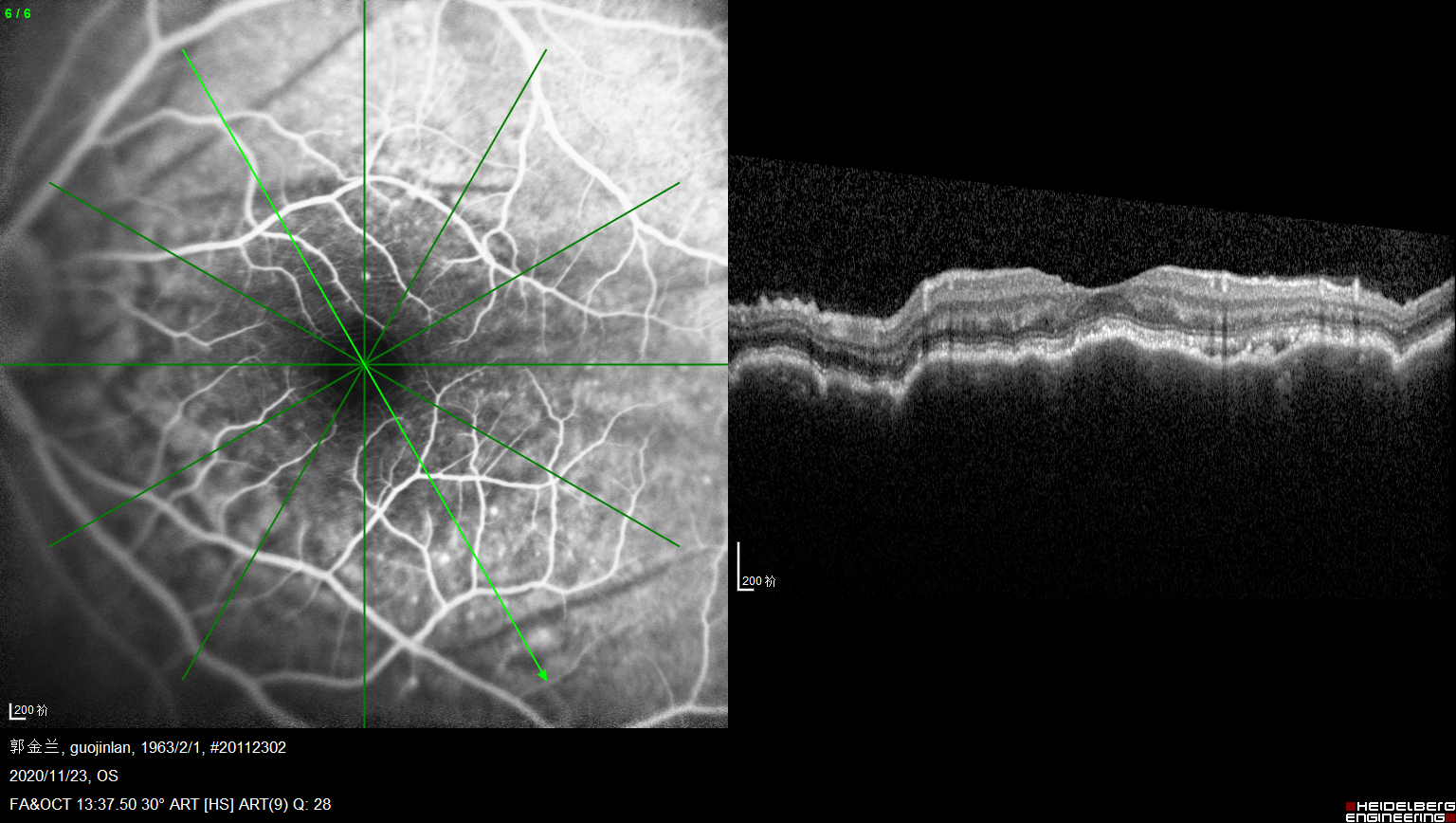

Supplement: Supplementary file 1 — Additional file 1: The raw data of this study. Table 1. The basic information of involved patients. [file 12886_2022_2598_MOESM1_ESM.zip › 1/Θâ¡Θçæσà░maltFFA/Θâ¡Θçæσà░g_012005.tif]

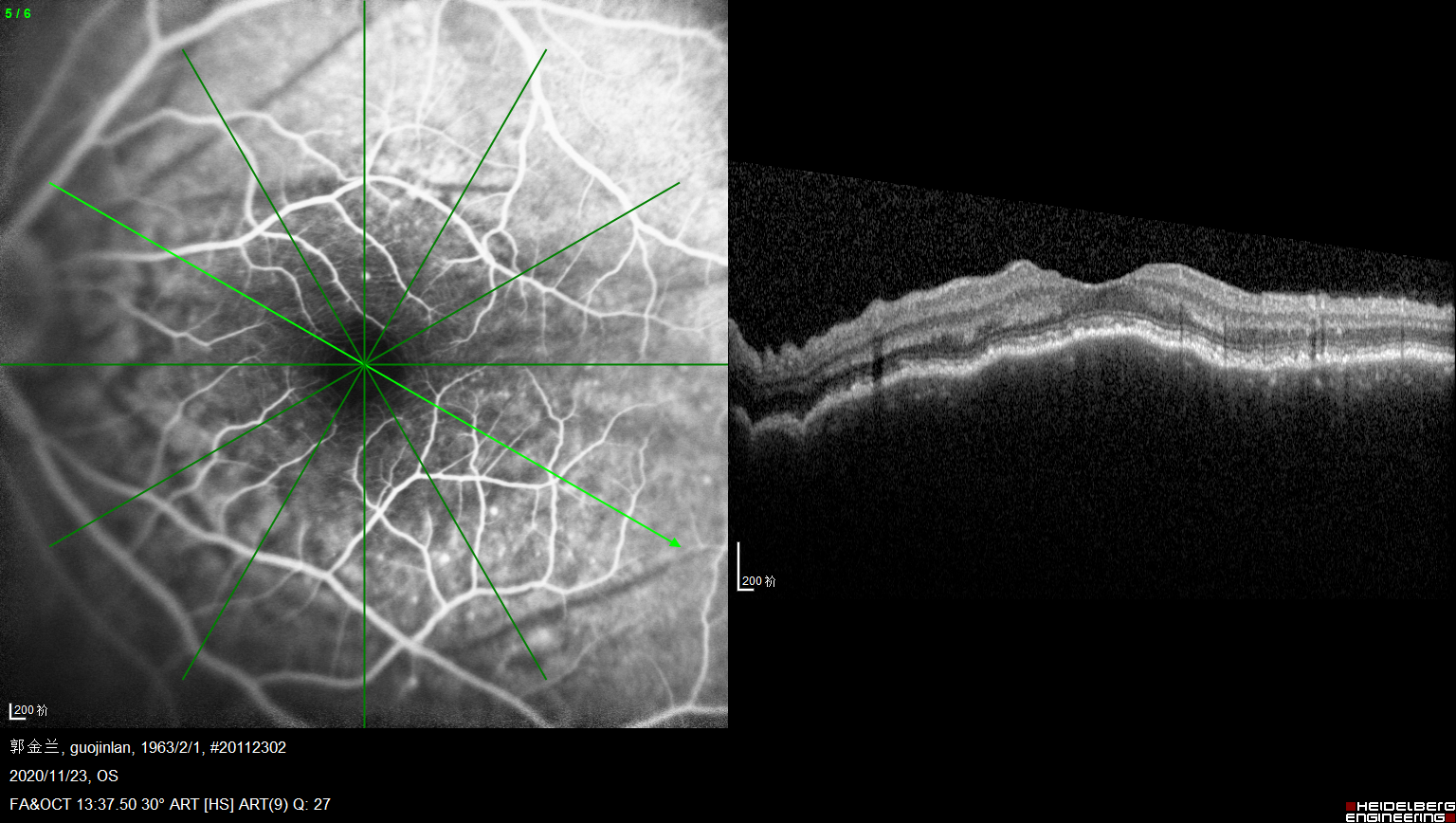

Supplement: Supplementary file 1 — Additional file 1: The raw data of this study. Table 1. The basic information of involved patients. [file 12886_2022_2598_MOESM1_ESM.zip › 1/Θâ¡Θçæσà░maltFFA/Θâ¡Θçæσà░g_012004.tif]

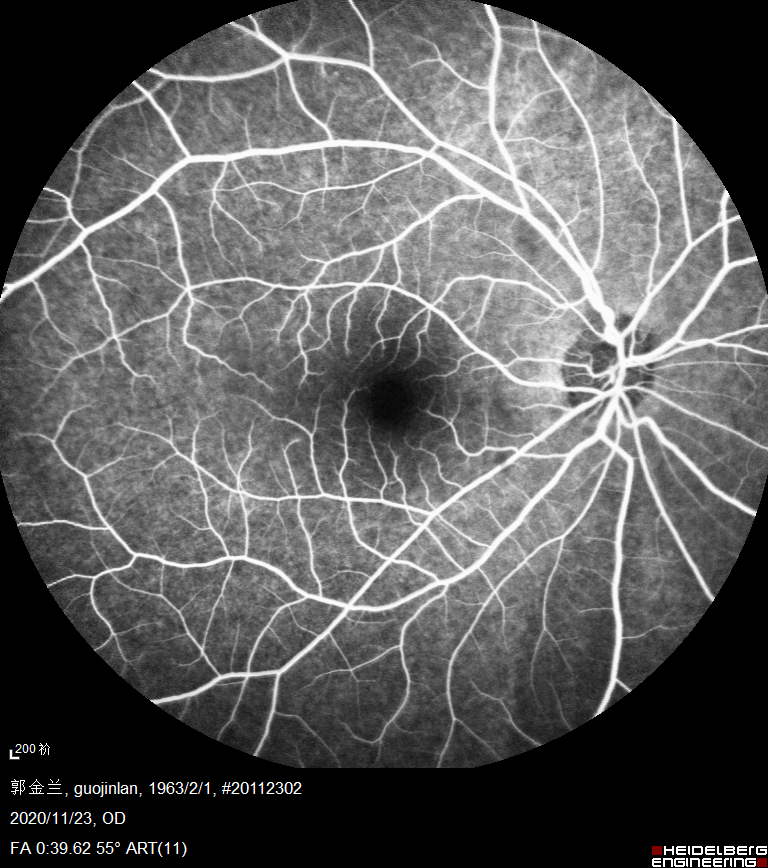

Supplement: Supplementary file 1 — Additional file 1: The raw data of this study. Table 1. The basic information of involved patients. [file 12886_2022_2598_MOESM1_ESM.zip › 1/Θâ¡Θçæσà░maltFFA/Θâ¡Θçæσà░g_009.tif]

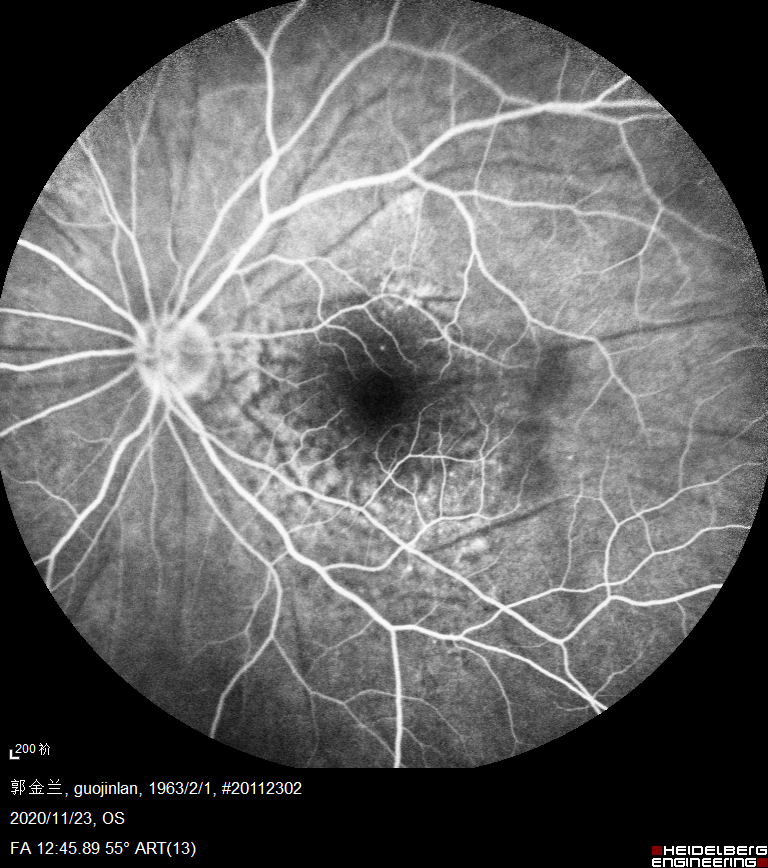

Supplement: Supplementary file 1 — Additional file 1: The raw data of this study. Table 1. The basic information of involved patients. [file 12886_2022_2598_MOESM1_ESM.zip › 1/Θâ¡Θçæσà░maltFFA/Θâ¡Θçæσà░g_008.tif]

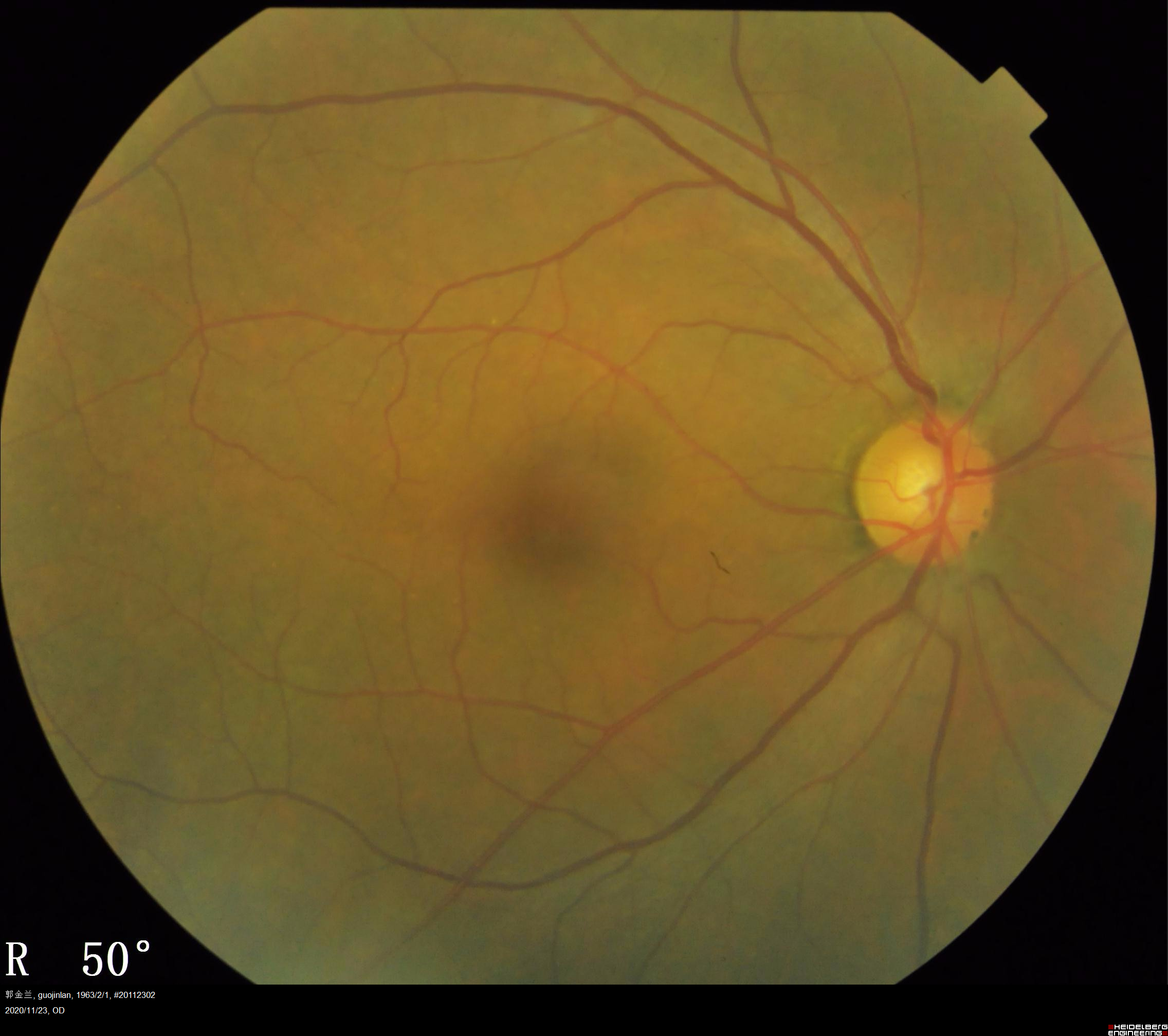

Supplement: Supplementary file 1 — Additional file 1: The raw data of this study. Table 1. The basic information of involved patients. [file 12886_2022_2598_MOESM1_ESM.zip › 1/Θâ¡Θçæσà░maltFFA/Θâ¡Θçæσà░g_000.tif]

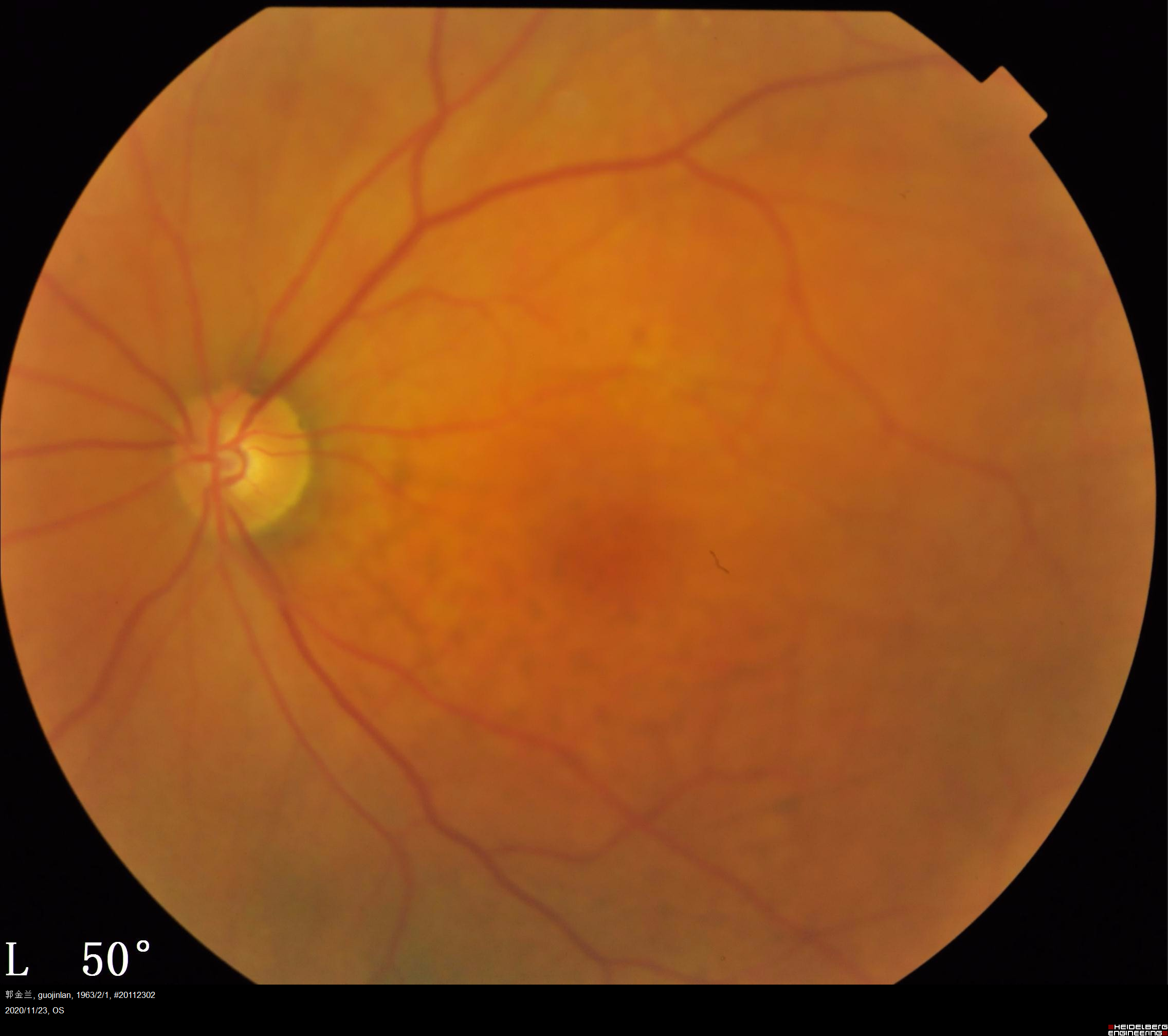

Supplement: Supplementary file 1 — Additional file 1: The raw data of this study. Table 1. The basic information of involved patients. [file 12886_2022_2598_MOESM1_ESM.zip › 1/Θâ¡Θçæσà░maltFFA/Θâ¡Θçæσà░g_001.tif]

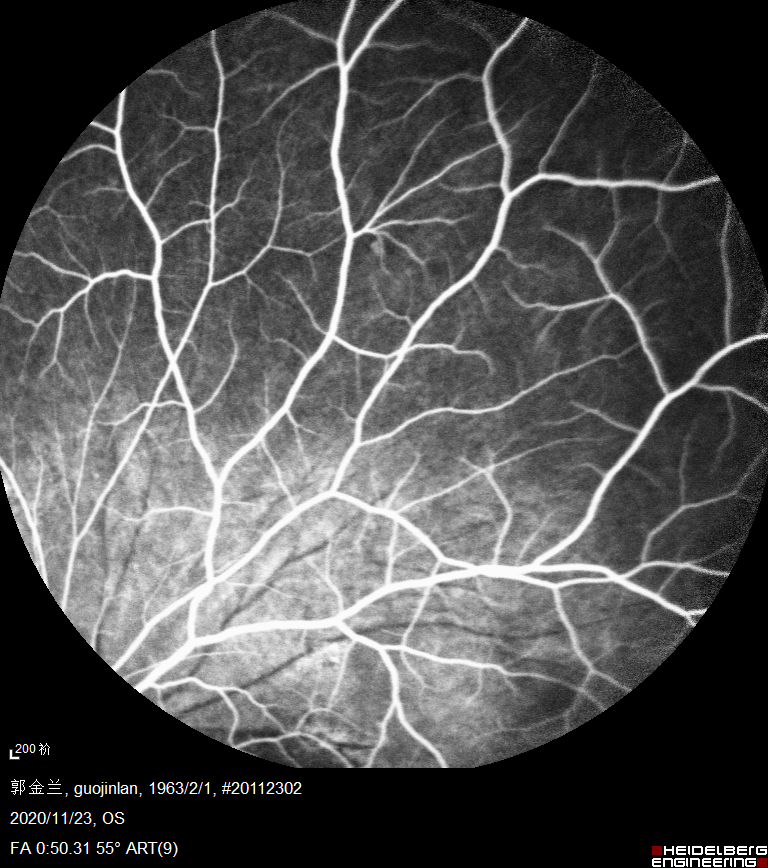

Supplement: Supplementary file 1 — Additional file 1: The raw data of this study. Table 1. The basic information of involved patients. [file 12886_2022_2598_MOESM1_ESM.zip › 1/Θâ¡Θçæσà░maltFFA/Θâ¡Θçæσà░g_003.tif]

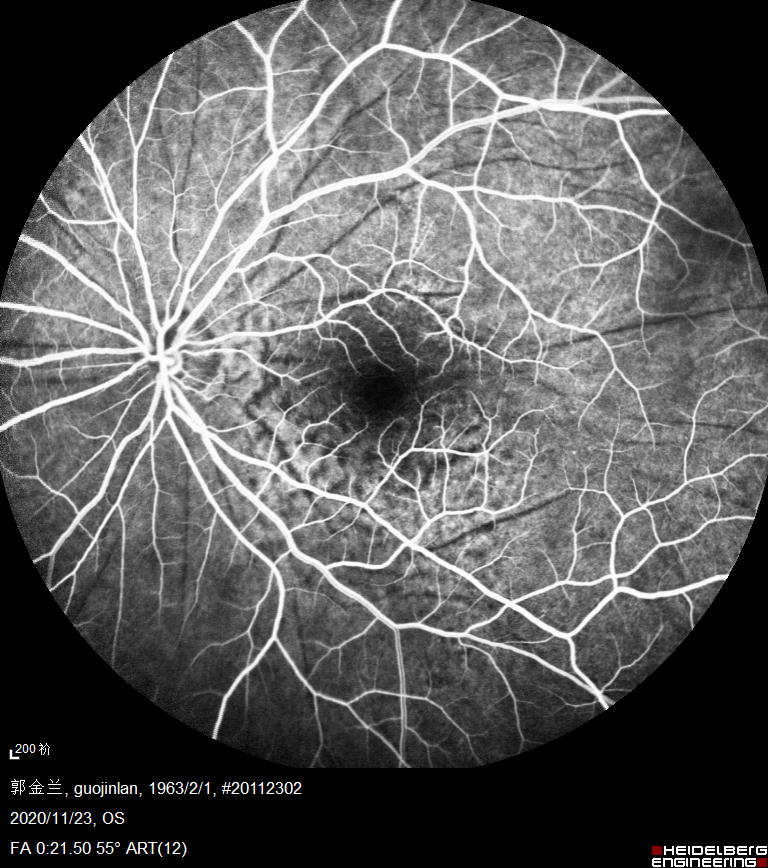

Supplement: Supplementary file 1 — Additional file 1: The raw data of this study. Table 1. The basic information of involved patients. [file 12886_2022_2598_MOESM1_ESM.zip › 1/Θâ¡Θçæσà░maltFFA/Θâ¡Θçæσà░g_002.tif]

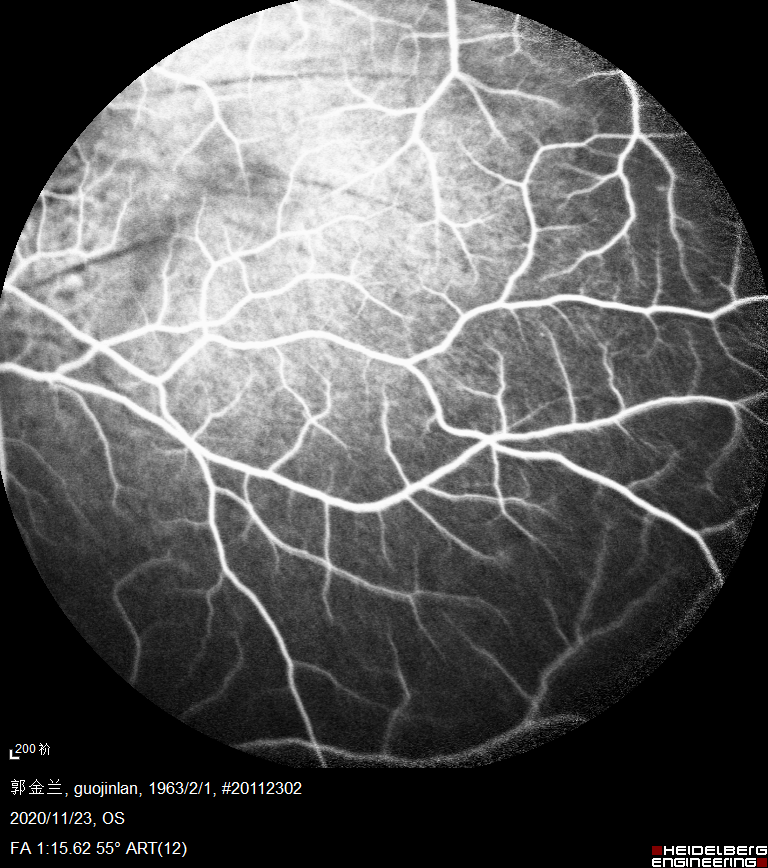

Supplement: Supplementary file 1 — Additional file 1: The raw data of this study. Table 1. The basic information of involved patients. [file 12886_2022_2598_MOESM1_ESM.zip › 1/Θâ¡Θçæσà░maltFFA/Θâ¡Θçæσà░g_006.tif]

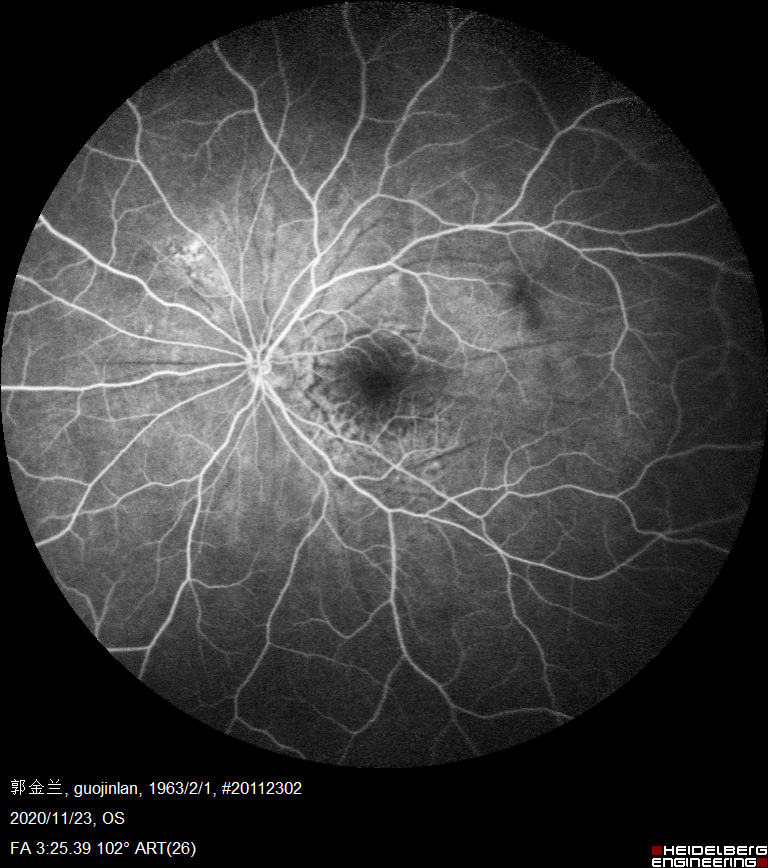

Supplement: Supplementary file 1 — Additional file 1: The raw data of this study. Table 1. The basic information of involved patients. [file 12886_2022_2598_MOESM1_ESM.zip › 1/Θâ¡Θçæσà░maltFFA/Θâ¡Θçæσà░g_007.tif]

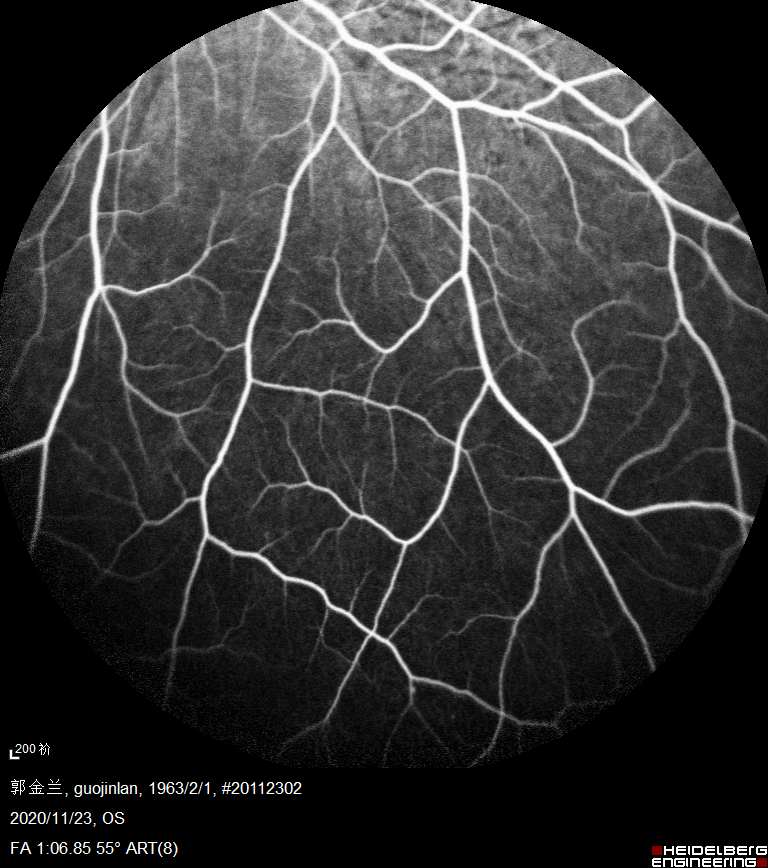

Supplement: Supplementary file 1 — Additional file 1: The raw data of this study. Table 1. The basic information of involved patients. [file 12886_2022_2598_MOESM1_ESM.zip › 1/Θâ¡Θçæσà░maltFFA/Θâ¡Θçæσà░g_005.tif]

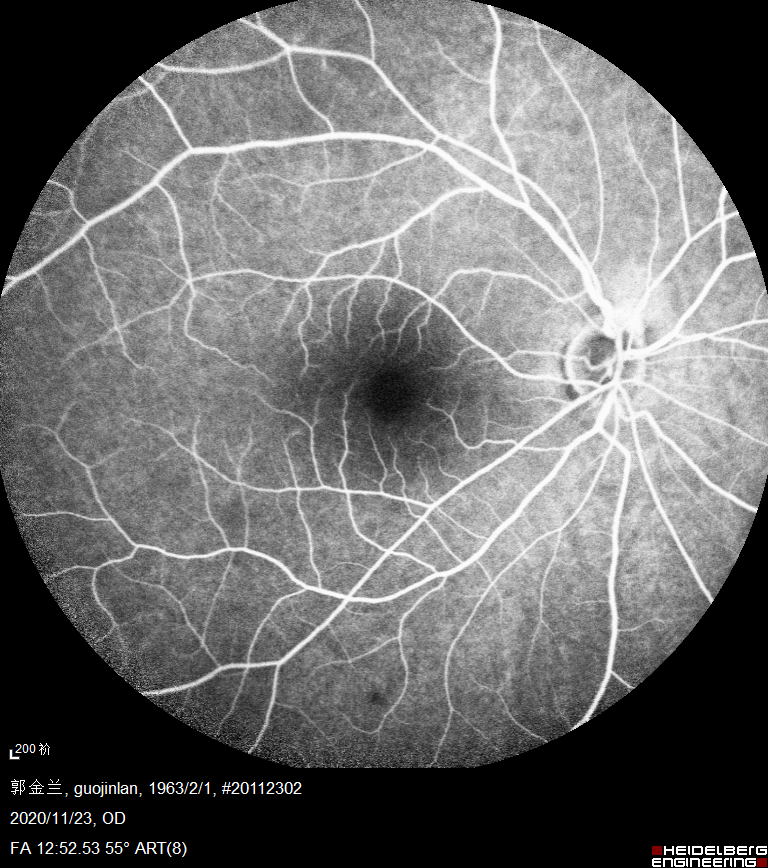

Supplement: Supplementary file 1 — Additional file 1: The raw data of this study. Table 1. The basic information of involved patients. [file 12886_2022_2598_MOESM1_ESM.zip › 1/Θâ¡Θçæσà░maltFFA/Θâ¡Θçæσà░g_011.tif]

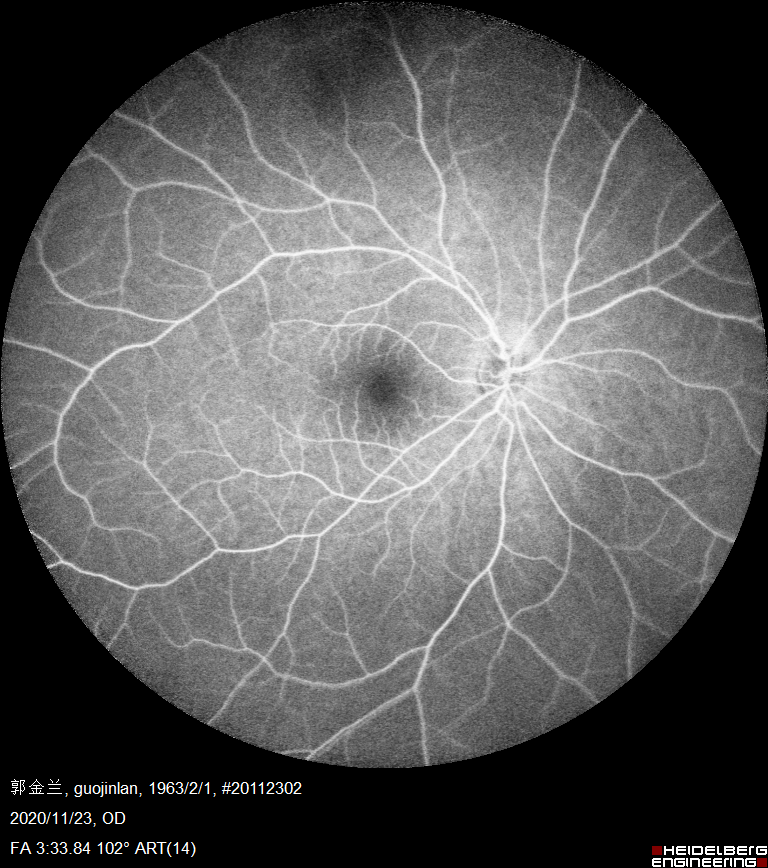

Supplement: Supplementary file 1 — Additional file 1: The raw data of this study. Table 1. The basic information of involved patients. [file 12886_2022_2598_MOESM1_ESM.zip › 1/Θâ¡Θçæσà░maltFFA/Θâ¡Θçæσà░g_010.tif]

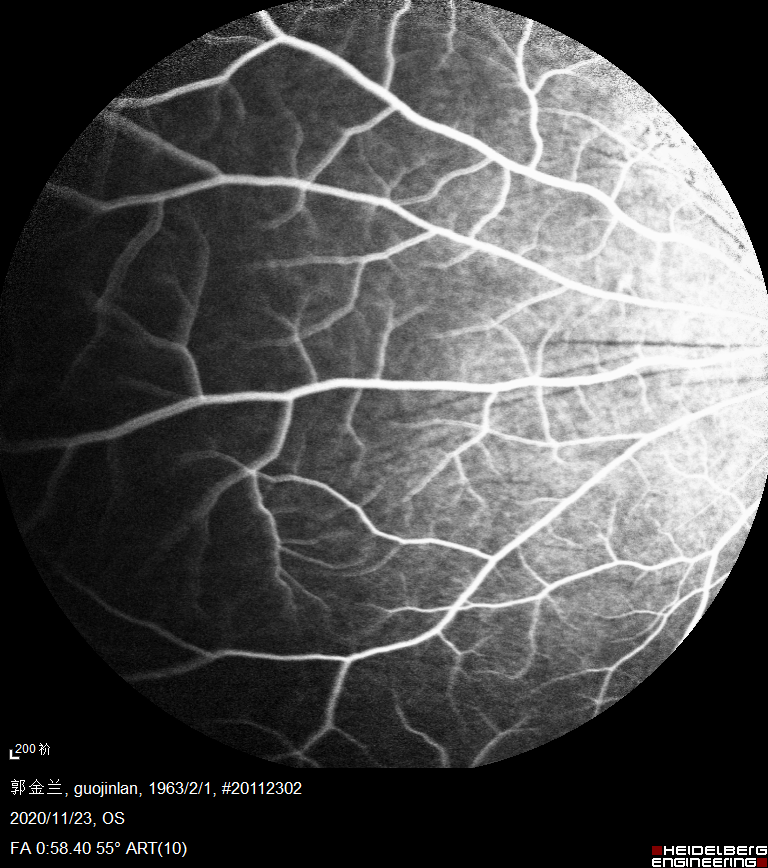

Supplement: Supplementary file 1 — Additional file 1: The raw data of this study. Table 1. The basic information of involved patients. [file 12886_2022_2598_MOESM1_ESM.zip › 1/Θâ¡Θçæσà░maltFFA/Θâ¡Θçæσà░g_004.tif]

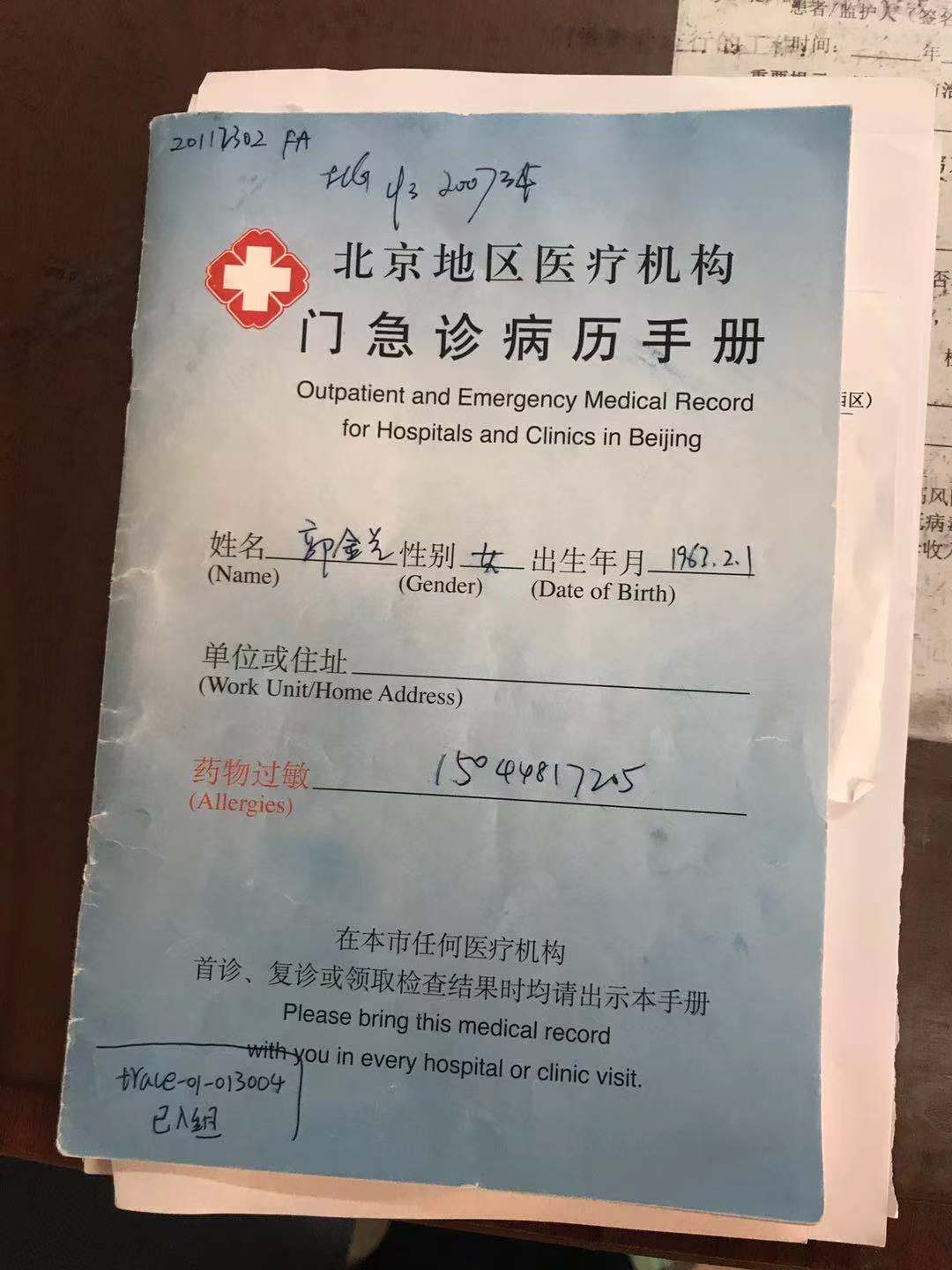

Supplement: Supplementary file 1 — Additional file 1: The raw data of this study. Table 1. The basic information of involved patients. [file 12886_2022_2598_MOESM1_ESM.zip › 1/τùàσÄåμò┤τÉå/21609732338_.pic.jpg]

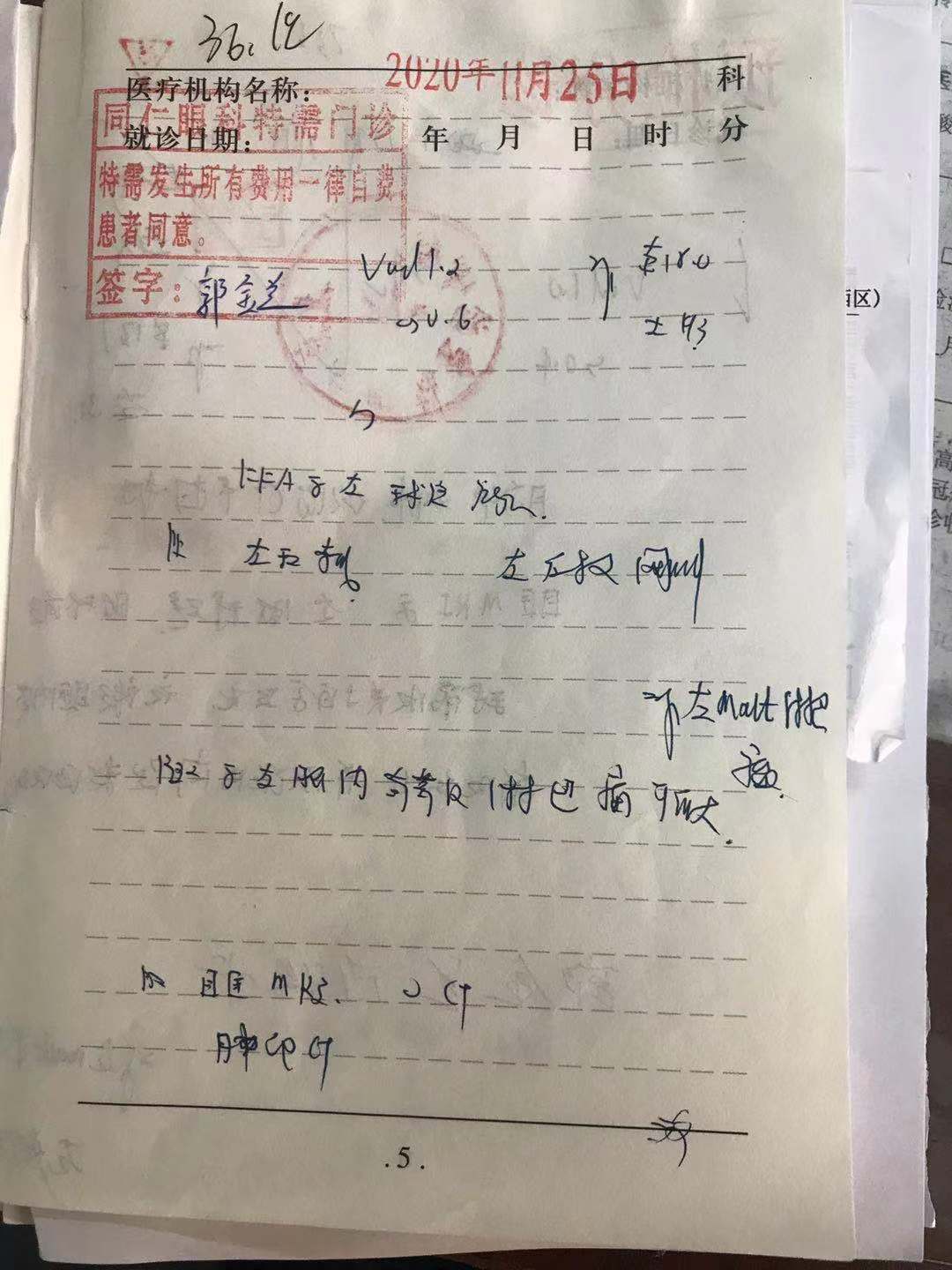

Supplement: Supplementary file 1 — Additional file 1: The raw data of this study. Table 1. The basic information of involved patients. [file 12886_2022_2598_MOESM1_ESM.zip › 1/τùàσÄåμò┤τÉå/71609732343_.pic.jpg]

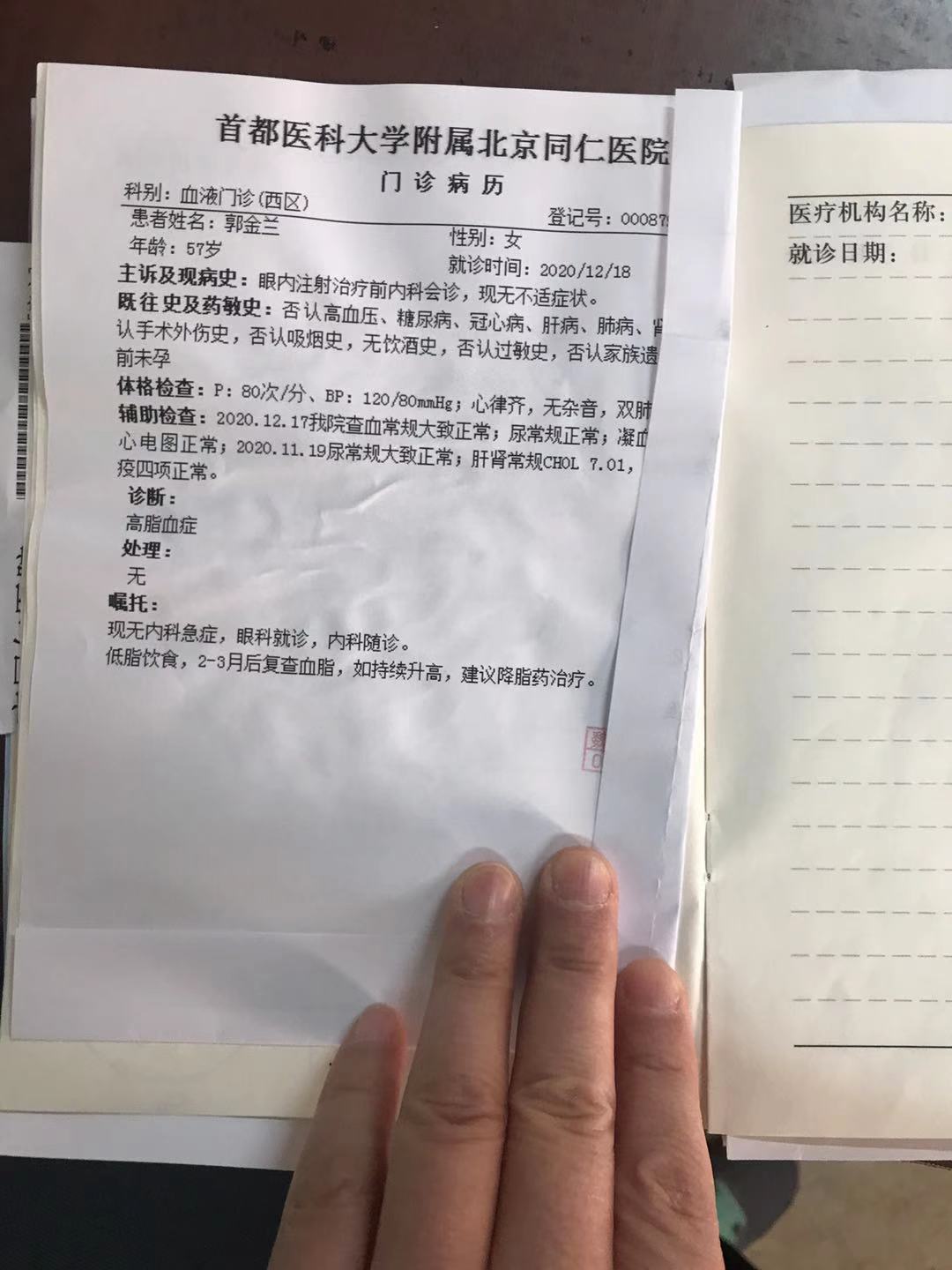

Supplement: Supplementary file 1 — Additional file 1: The raw data of this study. Table 1. The basic information of involved patients. [file 12886_2022_2598_MOESM1_ESM.zip › 1/τùàσÄåμò┤τÉå/151609732387_.pic.jpg]

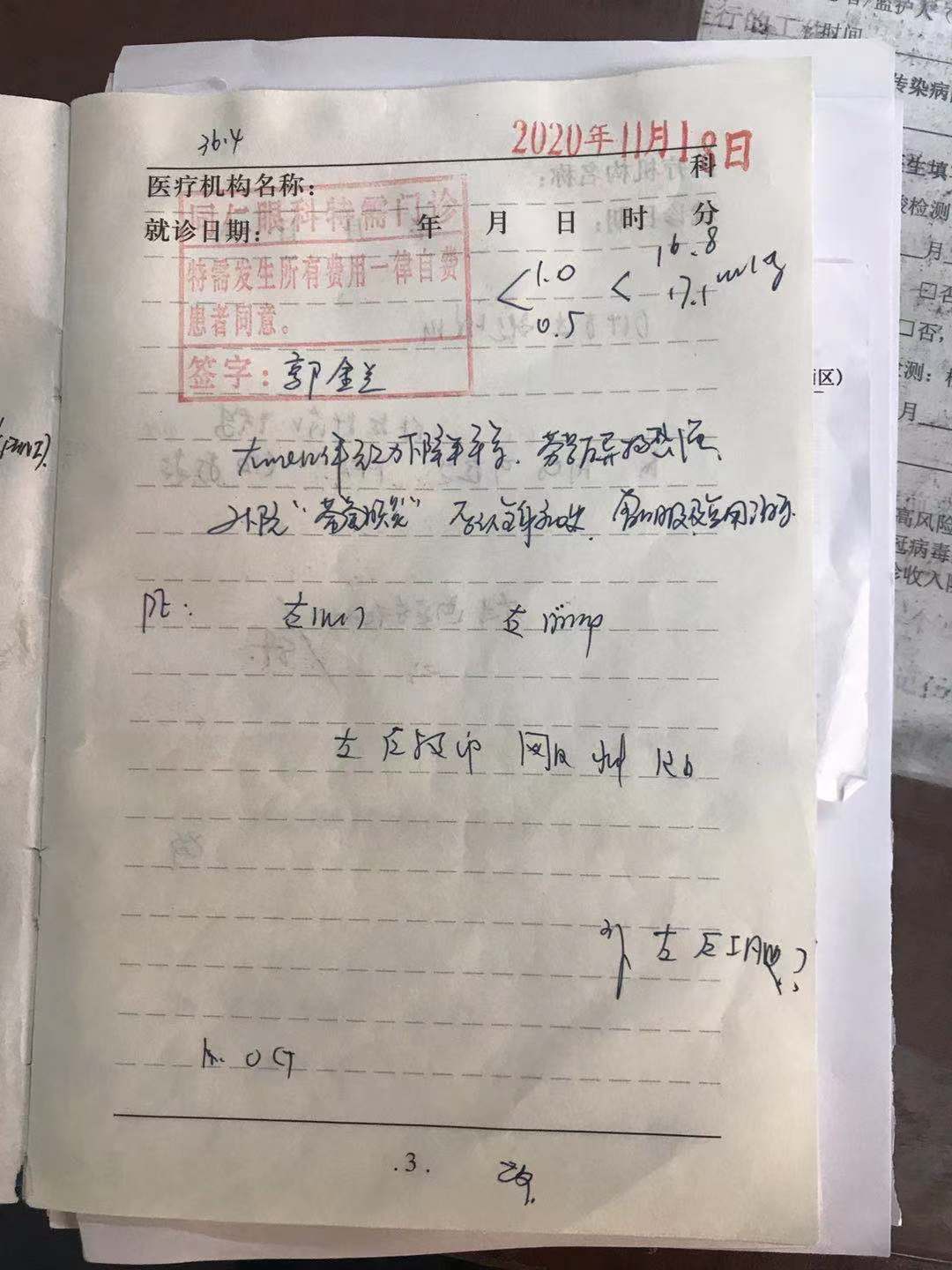

Supplement: Supplementary file 1 — Additional file 1: The raw data of this study. Table 1. The basic information of involved patients. [file 12886_2022_2598_MOESM1_ESM.zip › 1/τùàσÄåμò┤τÉå/51609732341_.pic.jpg]

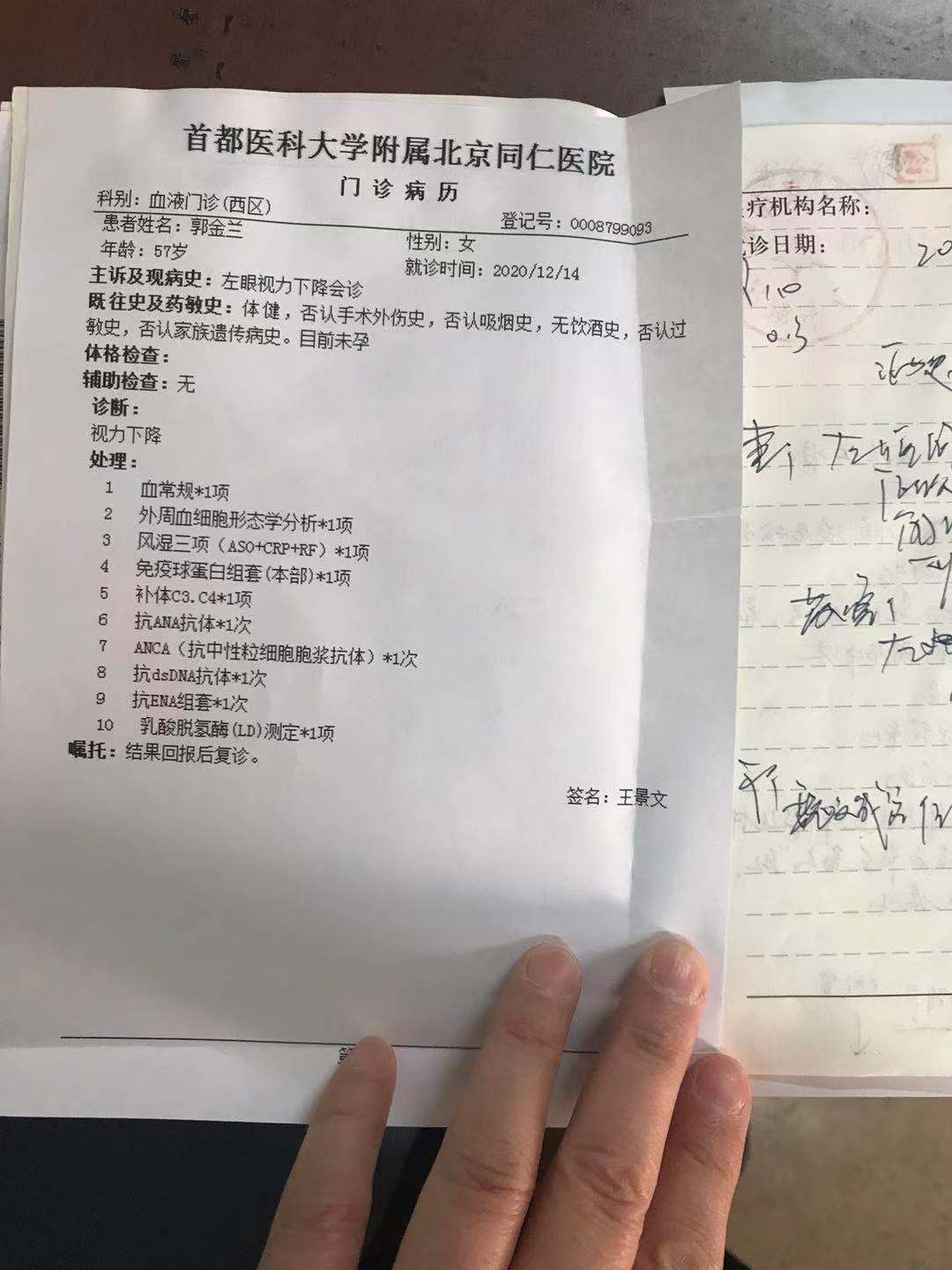

Supplement: Supplementary file 1 — Additional file 1: The raw data of this study. Table 1. The basic information of involved patients. [file 12886_2022_2598_MOESM1_ESM.zip › 1/τùàσÄåμò┤τÉå/101609732346_.pic.jpg]

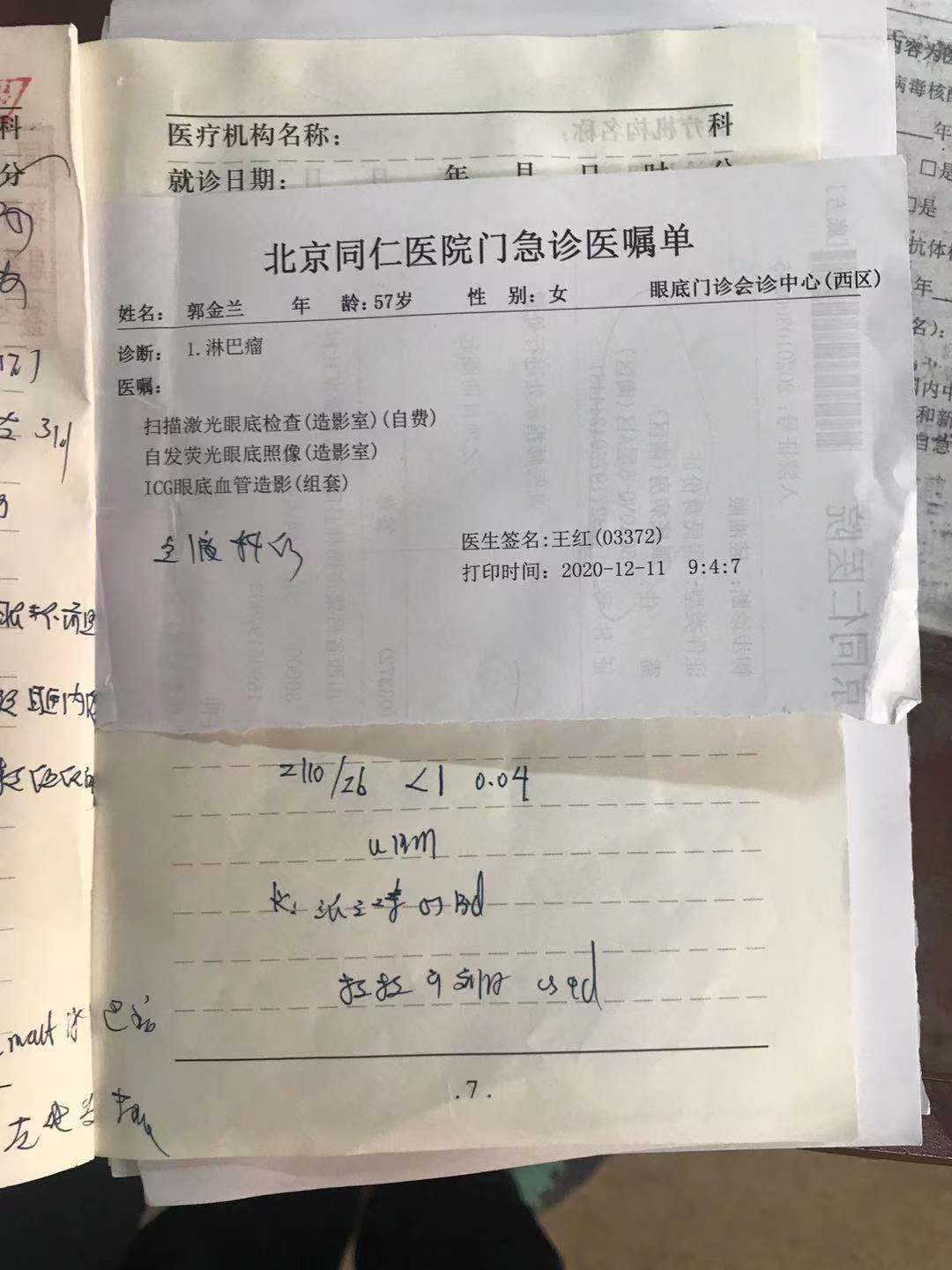

Supplement: Supplementary file 1 — Additional file 1: The raw data of this study. Table 1. The basic information of involved patients. [file 12886_2022_2598_MOESM1_ESM.zip › 1/τùàσÄåμò┤τÉå/91609732345_.pic.jpg]

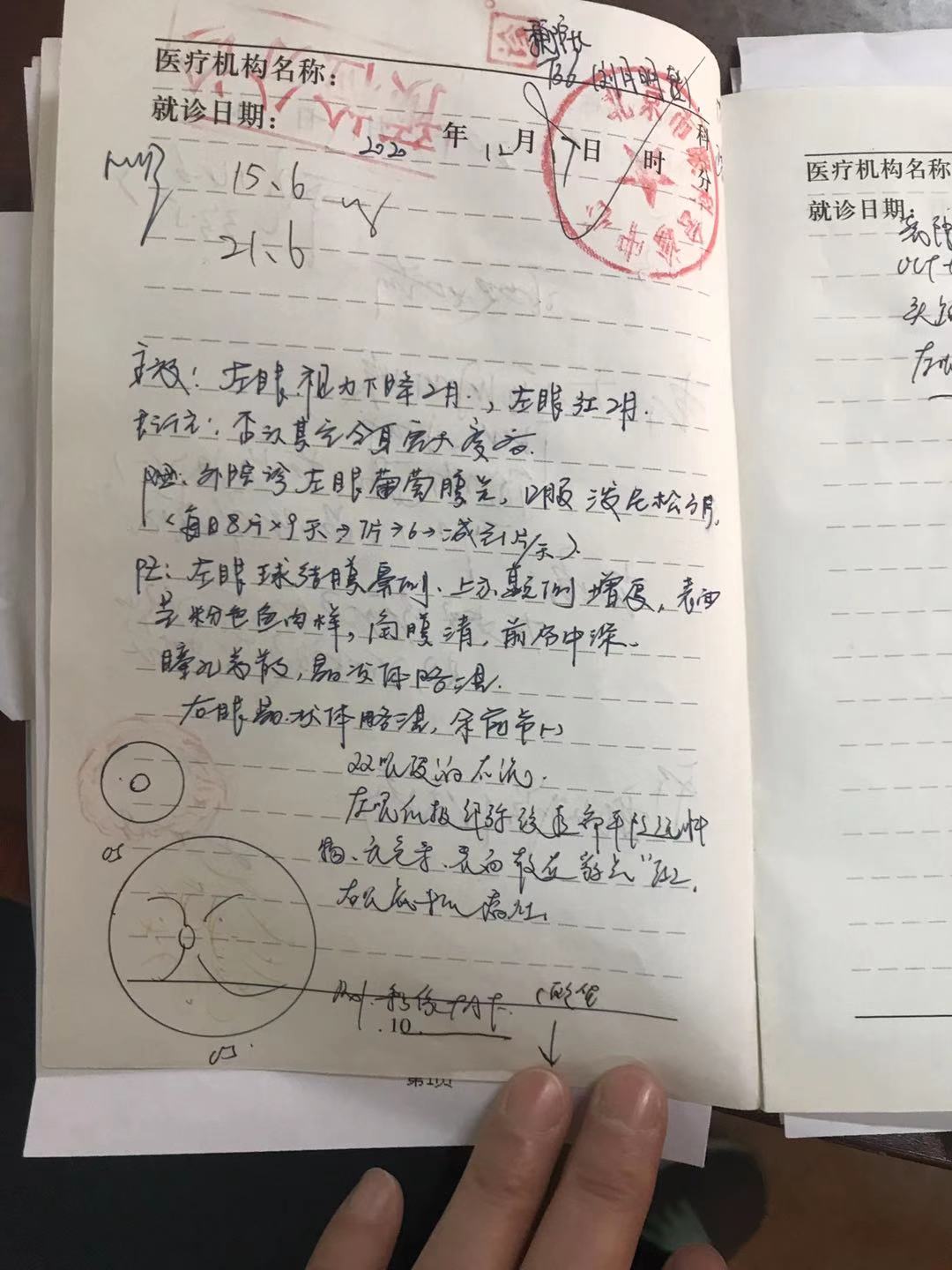

Supplement: Supplementary file 1 — Additional file 1: The raw data of this study. Table 1. The basic information of involved patients. [file 12886_2022_2598_MOESM1_ESM.zip › 1/τùàσÄåμò┤τÉå/131609732385_.pic.jpg]

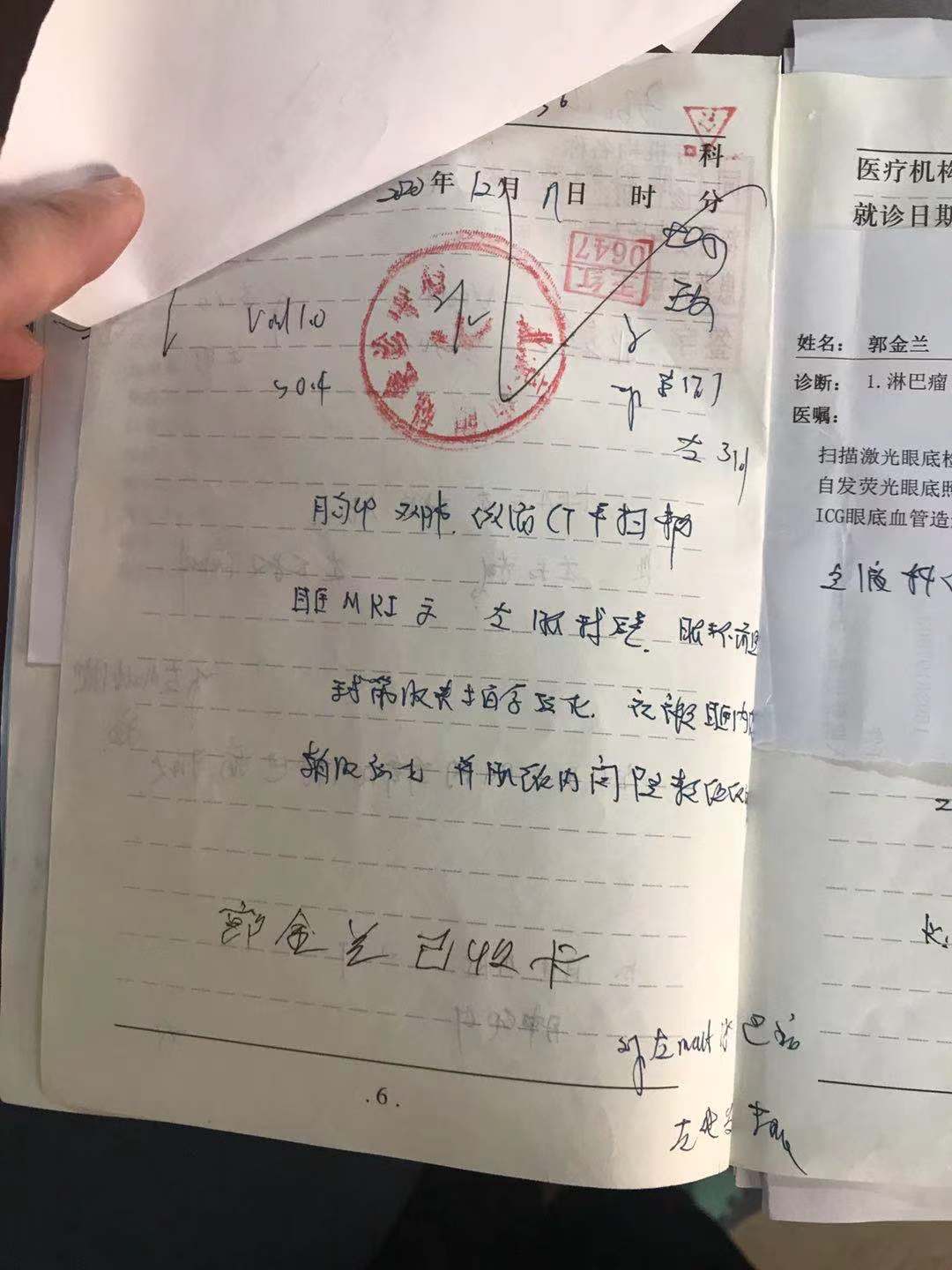

Supplement: Supplementary file 1 — Additional file 1: The raw data of this study. Table 1. The basic information of involved patients. [file 12886_2022_2598_MOESM1_ESM.zip › 1/τùàσÄåμò┤τÉå/81609732344_.pic.jpg]

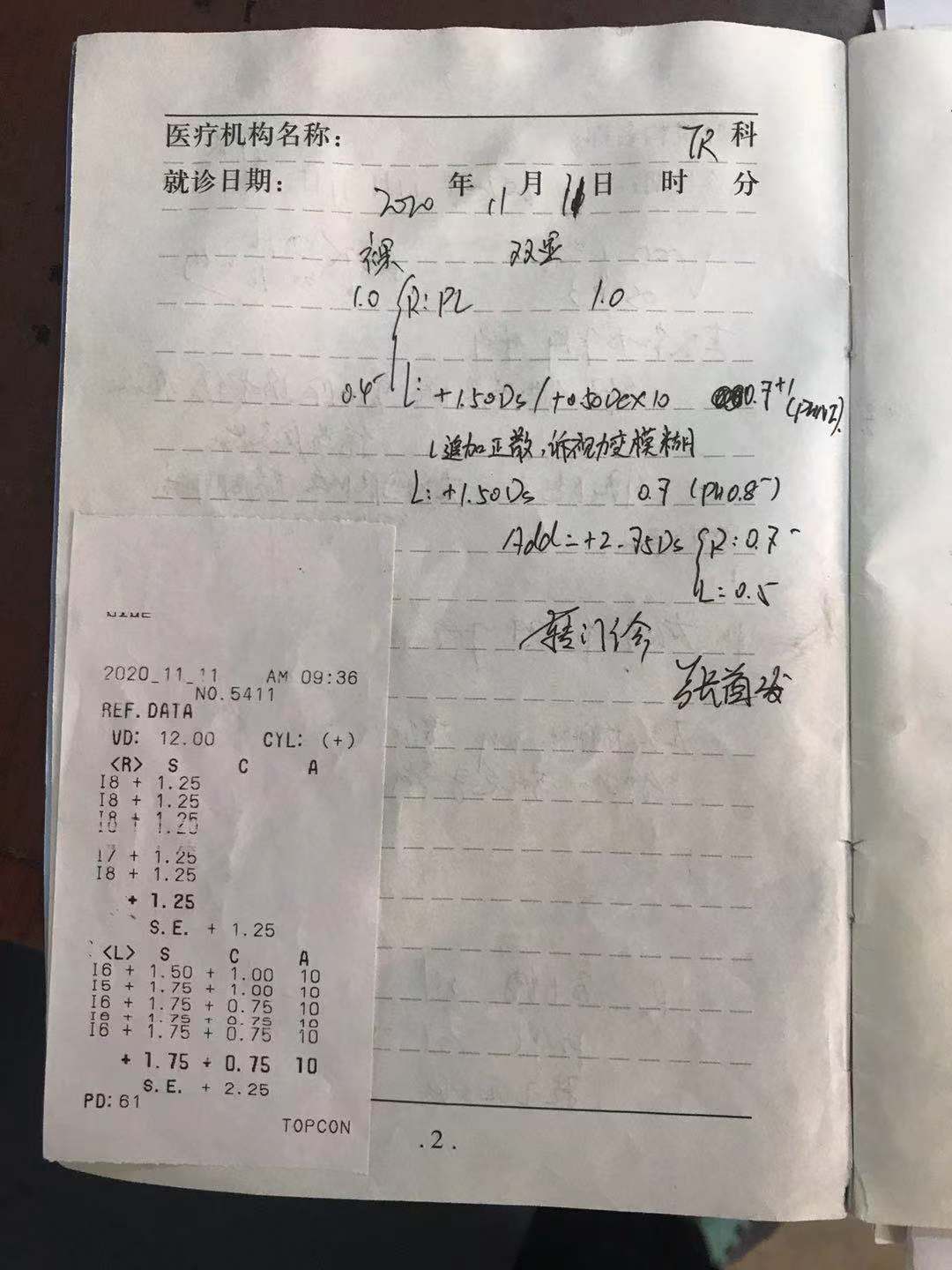

Supplement: Supplementary file 1 — Additional file 1: The raw data of this study. Table 1. The basic information of involved patients. [file 12886_2022_2598_MOESM1_ESM.zip › 1/τùàσÄåμò┤τÉå/41609732340_.pic.jpg]

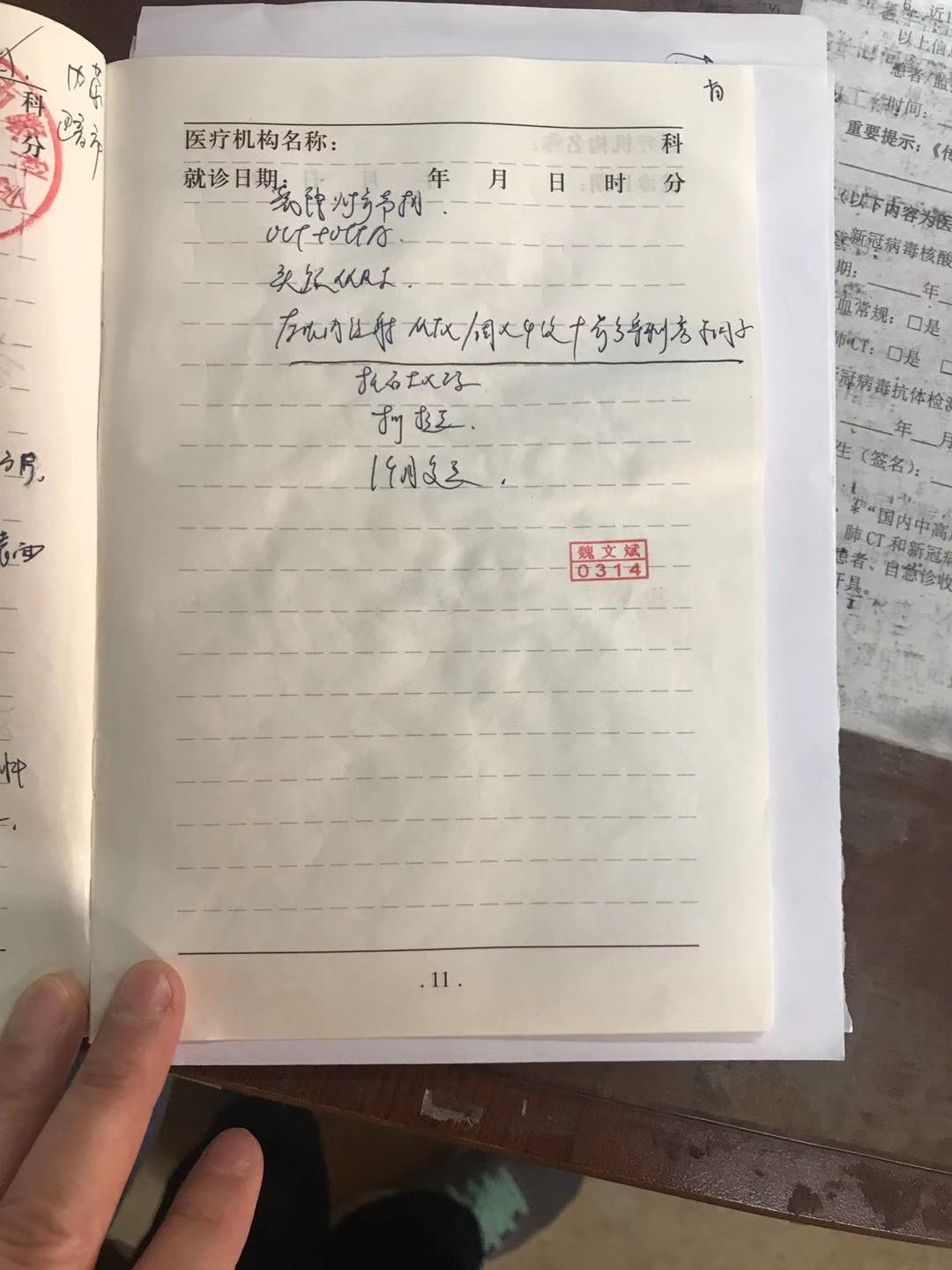

Supplement: Supplementary file 1 — Additional file 1: The raw data of this study. Table 1. The basic information of involved patients. [file 12886_2022_2598_MOESM1_ESM.zip › 1/τùàσÄåμò┤τÉå/141609732386_.pic.jpg]

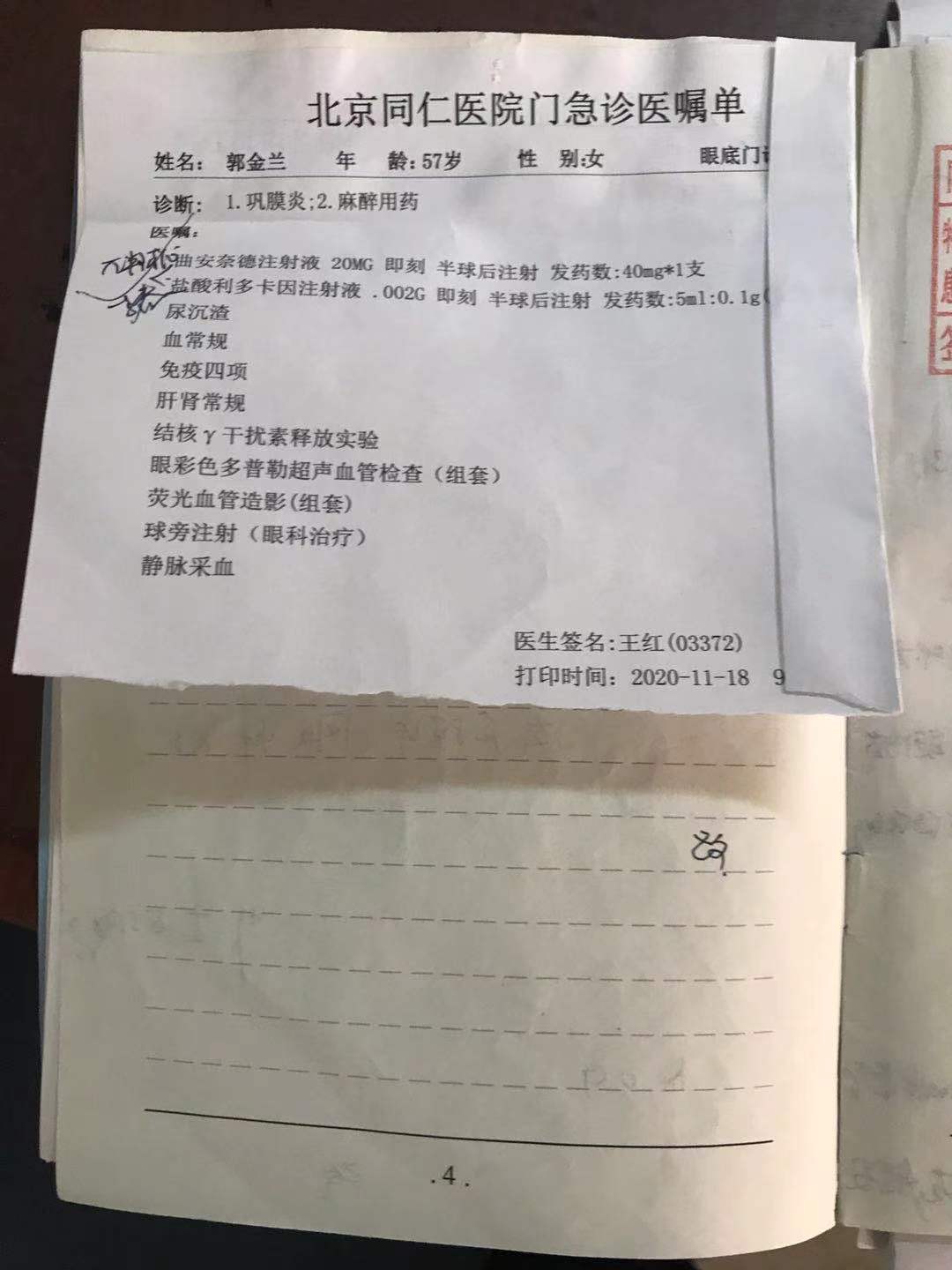

Supplement: Supplementary file 1 — Additional file 1: The raw data of this study. Table 1. The basic information of involved patients. [file 12886_2022_2598_MOESM1_ESM.zip › 1/τùàσÄåμò┤τÉå/61609732342_.pic.jpg]

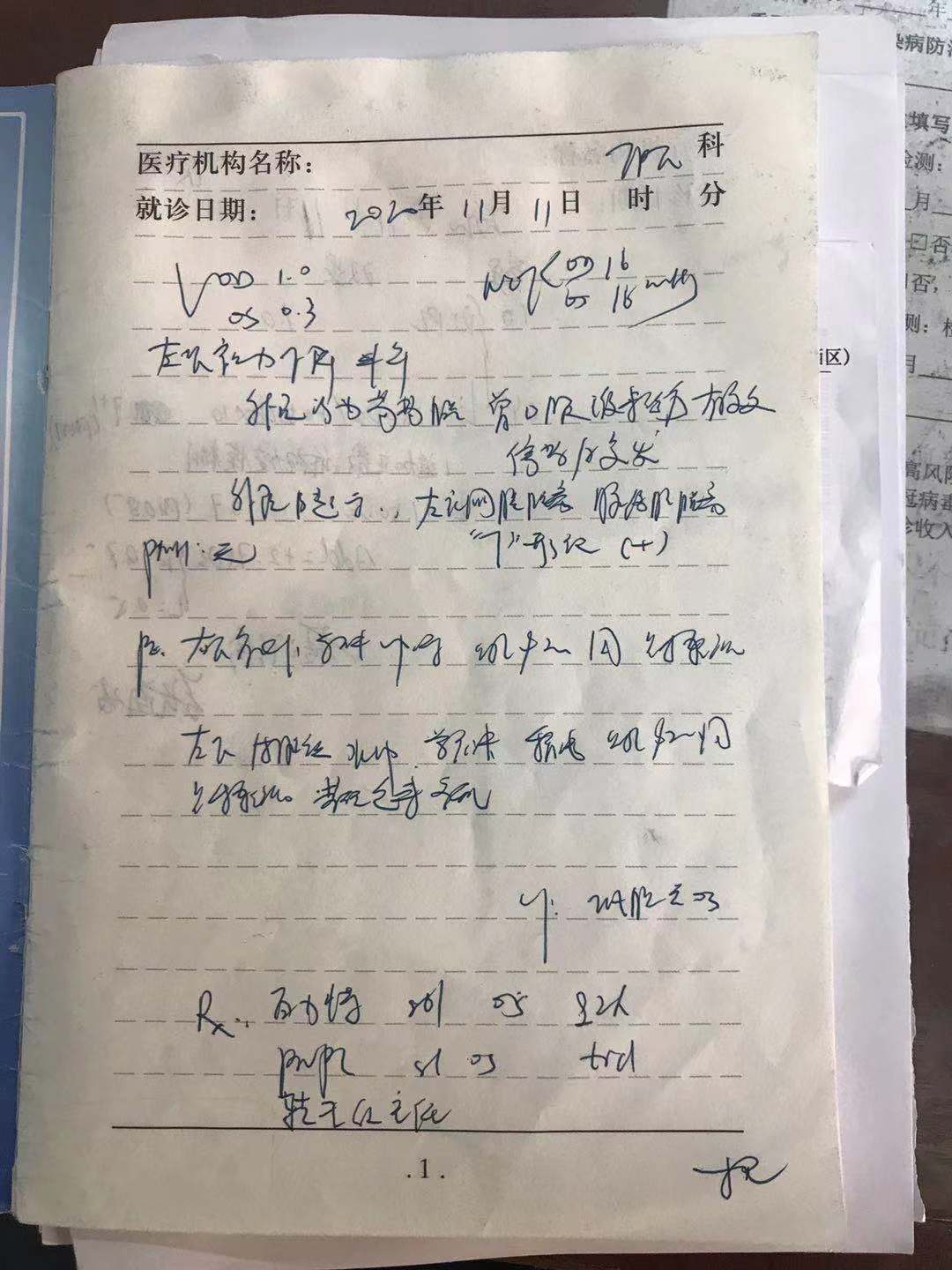

Supplement: Supplementary file 1 — Additional file 1: The raw data of this study. Table 1. The basic information of involved patients. [file 12886_2022_2598_MOESM1_ESM.zip › 1/τùàσÄåμò┤τÉå/31609732339_.pic.jpg]

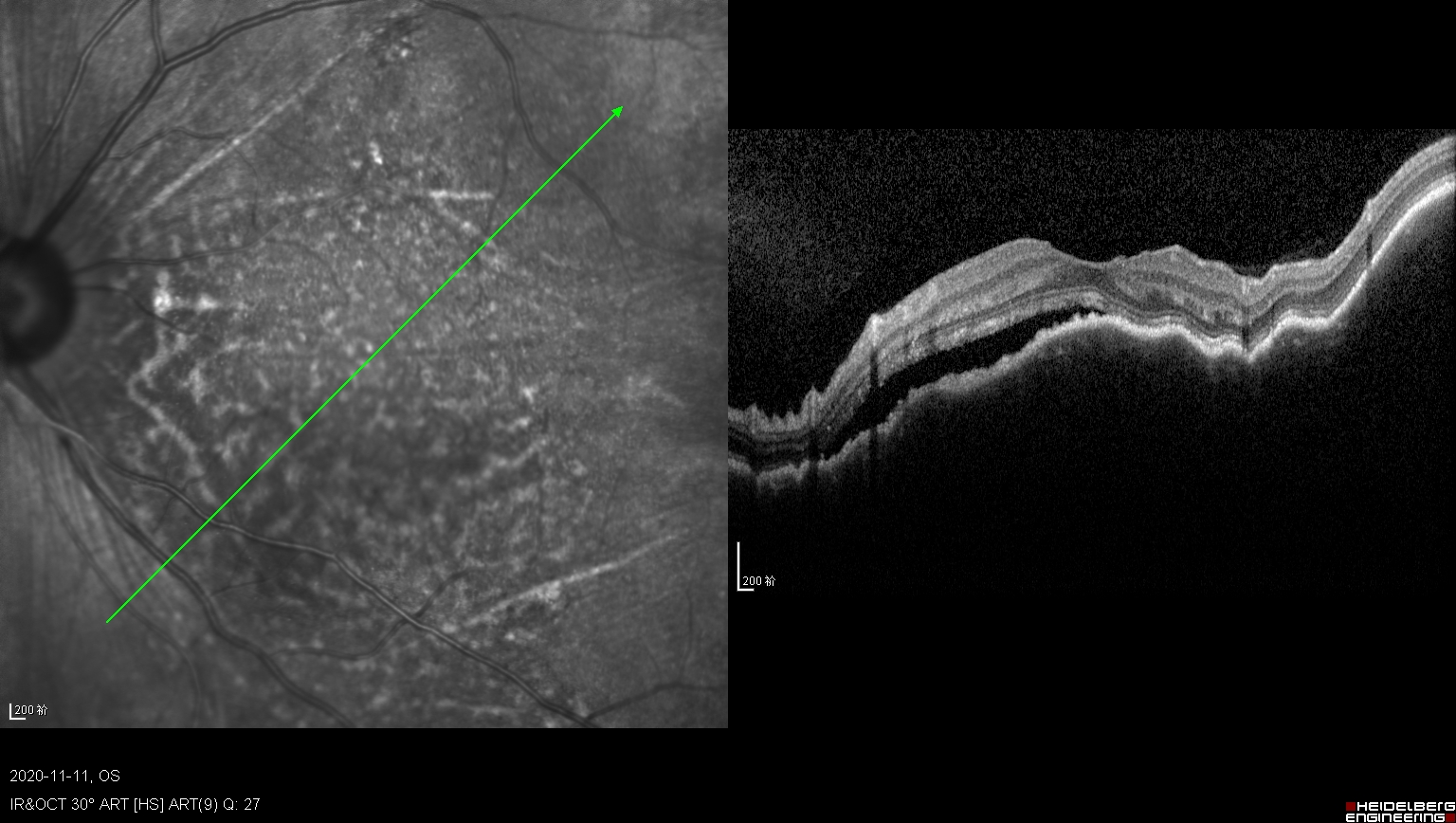

Supplement: Supplementary file 1 — Additional file 1: The raw data of this study. Table 1. The basic information of involved patients. [file 12886_2022_2598_MOESM1_ESM.zip › 1/OCTτ£╝σ║òτàoτ¢╕/20201111110049250_100002_801486_1489.jpg]

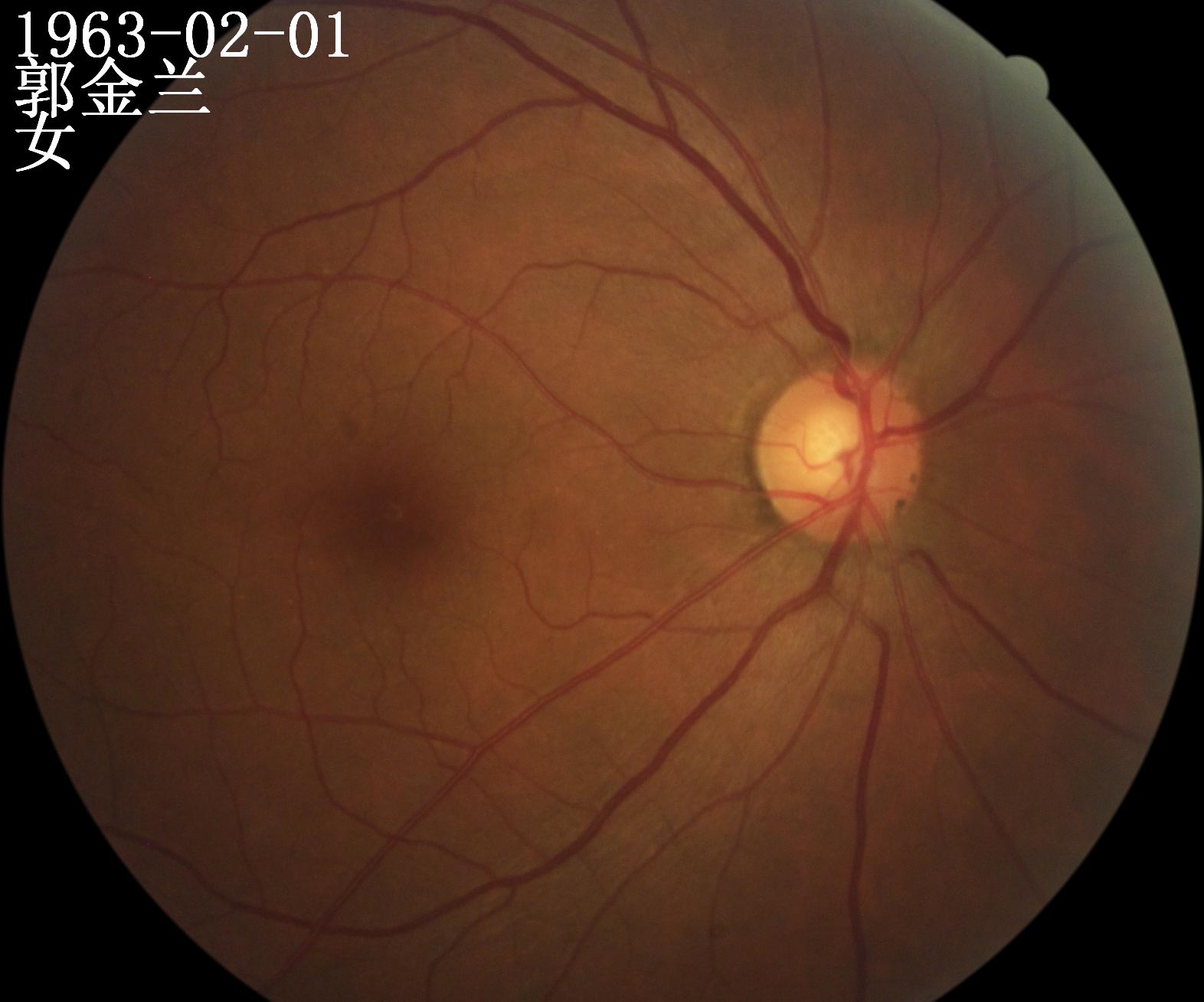

Supplement: Supplementary file 1 — Additional file 1: The raw data of this study. Table 1. The basic information of involved patients. [file 12886_2022_2598_MOESM1_ESM.zip › 1/OCTτ£╝σ║òτàoτ¢╕/20201111110709437_100002_800143_1897.jpg]

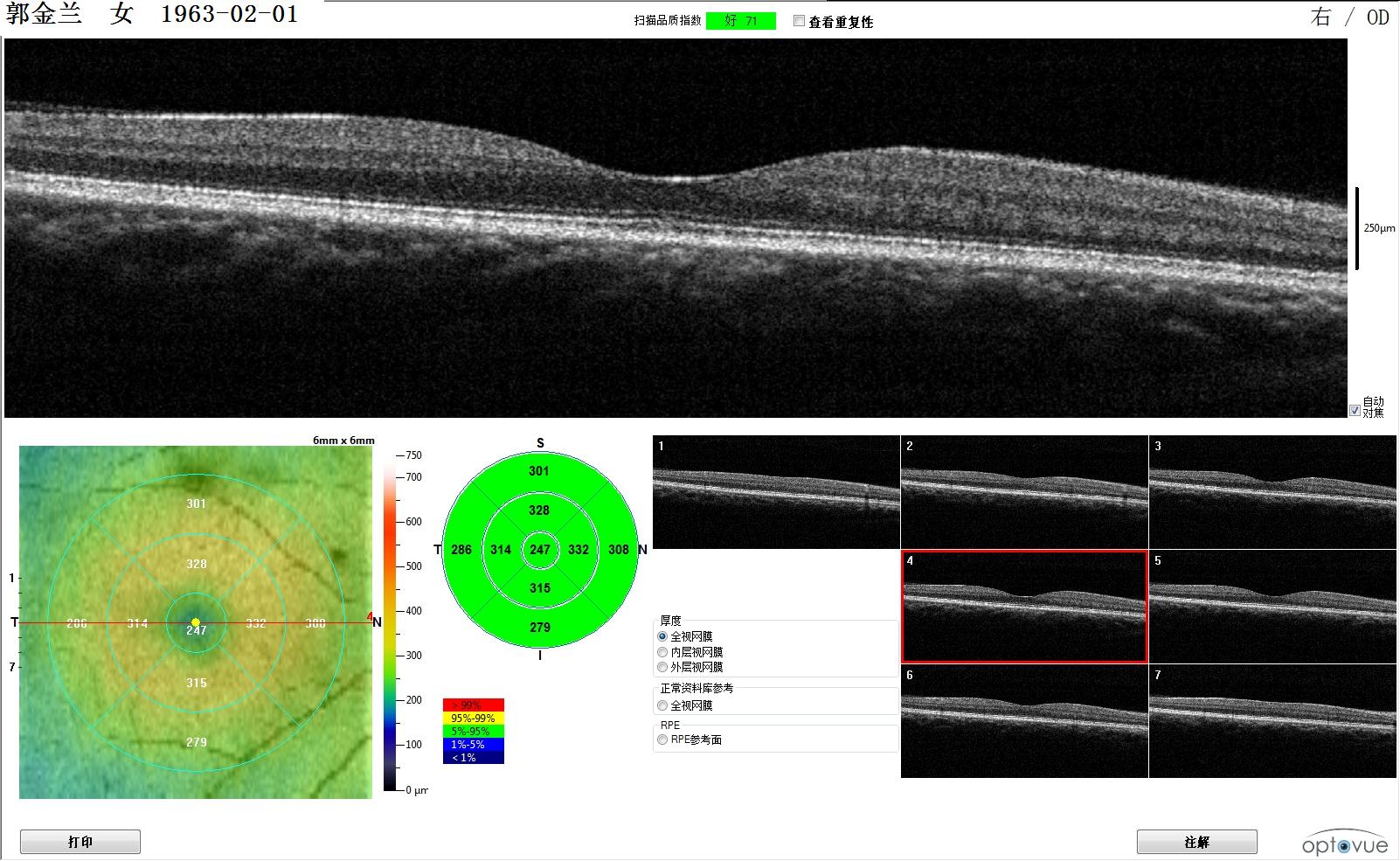

Supplement: Supplementary file 1 — Additional file 1: The raw data of this study. Table 1. The basic information of involved patients. [file 12886_2022_2598_MOESM1_ESM.zip › 1/OCTτ£╝σ║òτàoτ¢╕/20201125114949144_100002_800159_3454.jpg]

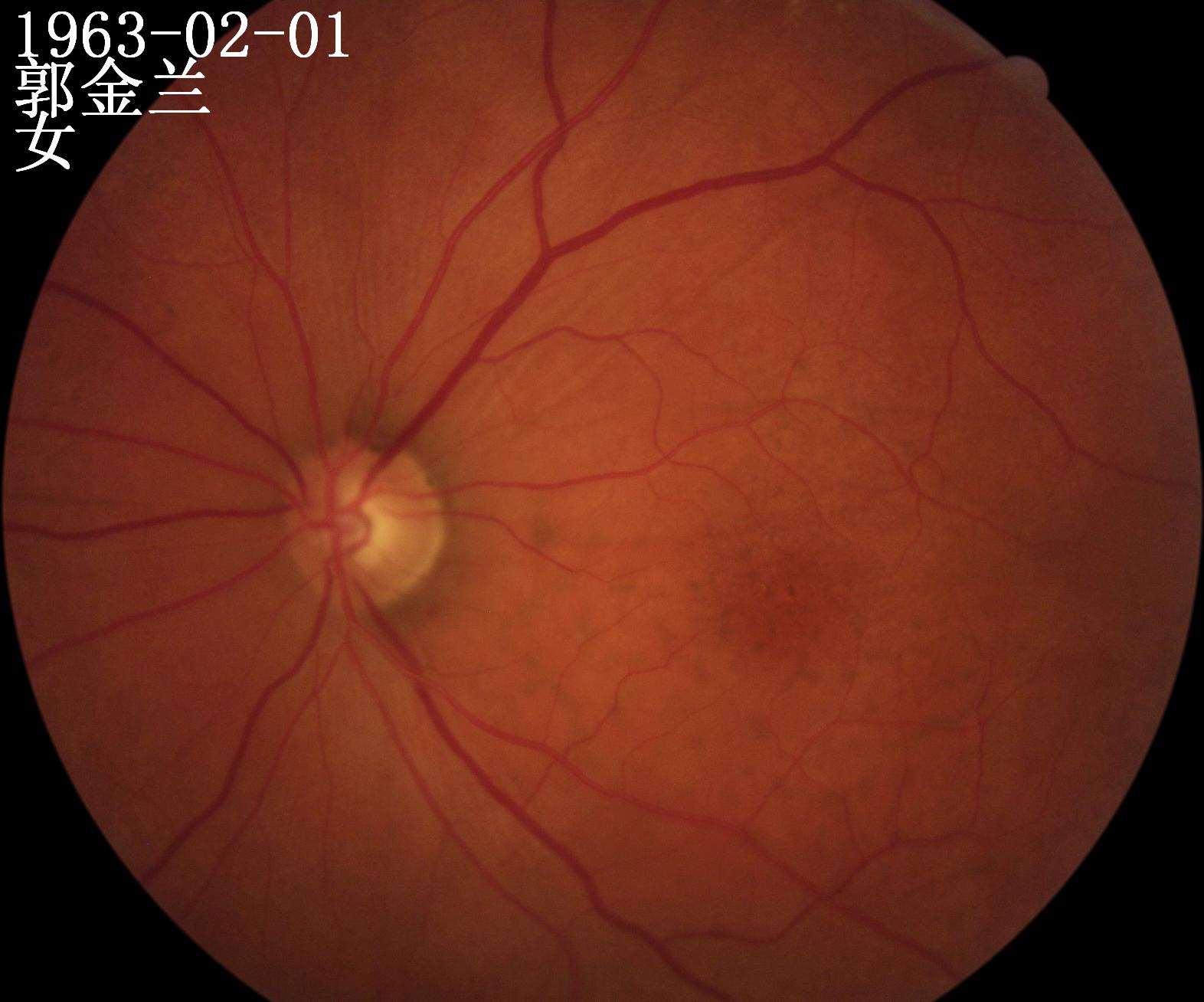

Supplement: Supplementary file 1 — Additional file 1: The raw data of this study. Table 1. The basic information of involved patients. [file 12886_2022_2598_MOESM1_ESM.zip › 1/OCTτ£╝σ║òτàoτ¢╕/20201111110710375_100002_800143_1305.jpg]

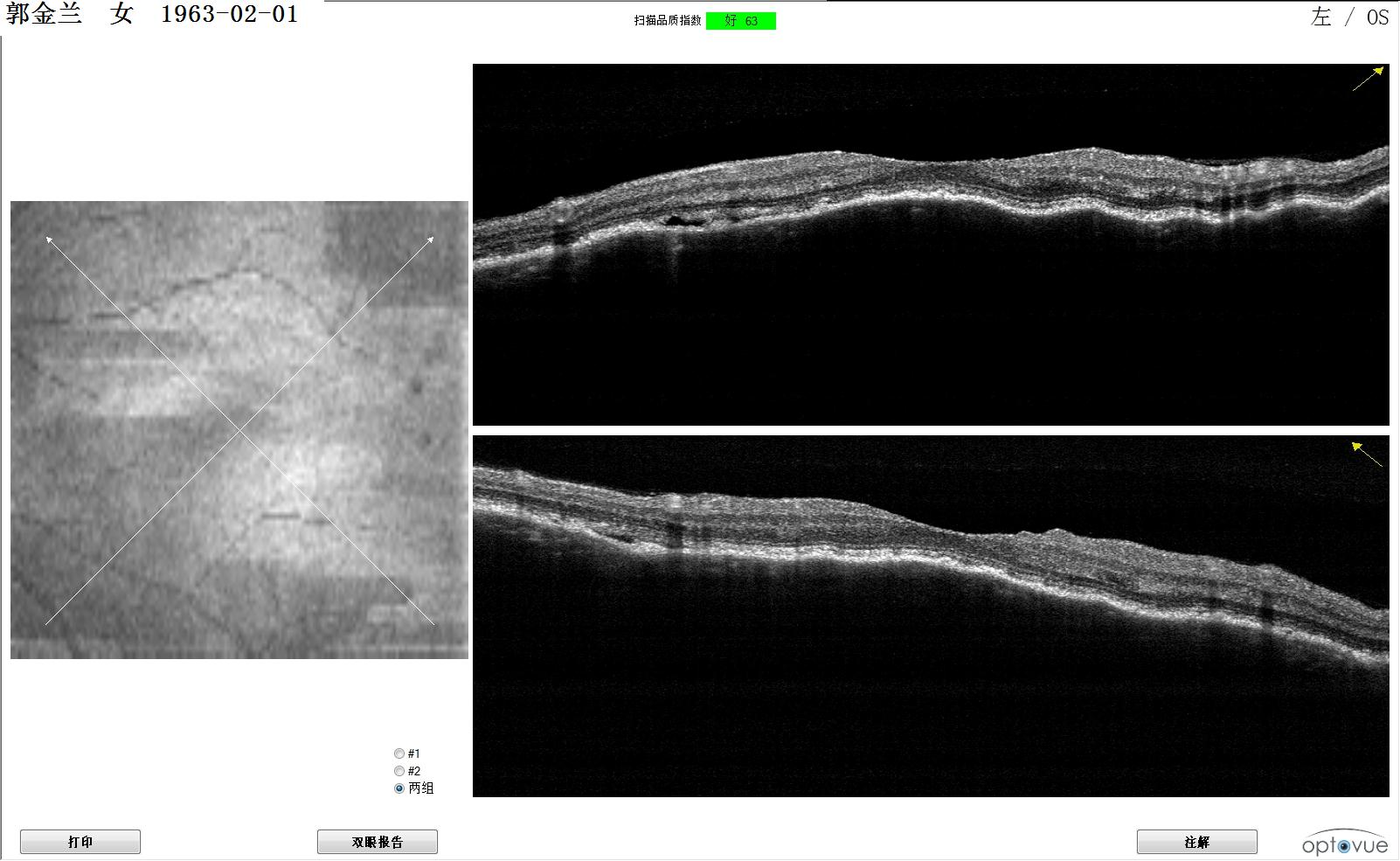

Supplement: Supplementary file 1 — Additional file 1: The raw data of this study. Table 1. The basic information of involved patients. [file 12886_2022_2598_MOESM1_ESM.zip › 1/OCTτ£╝σ║òτàoτ¢╕/20201125114951110_100002_800159_4917.jpg]

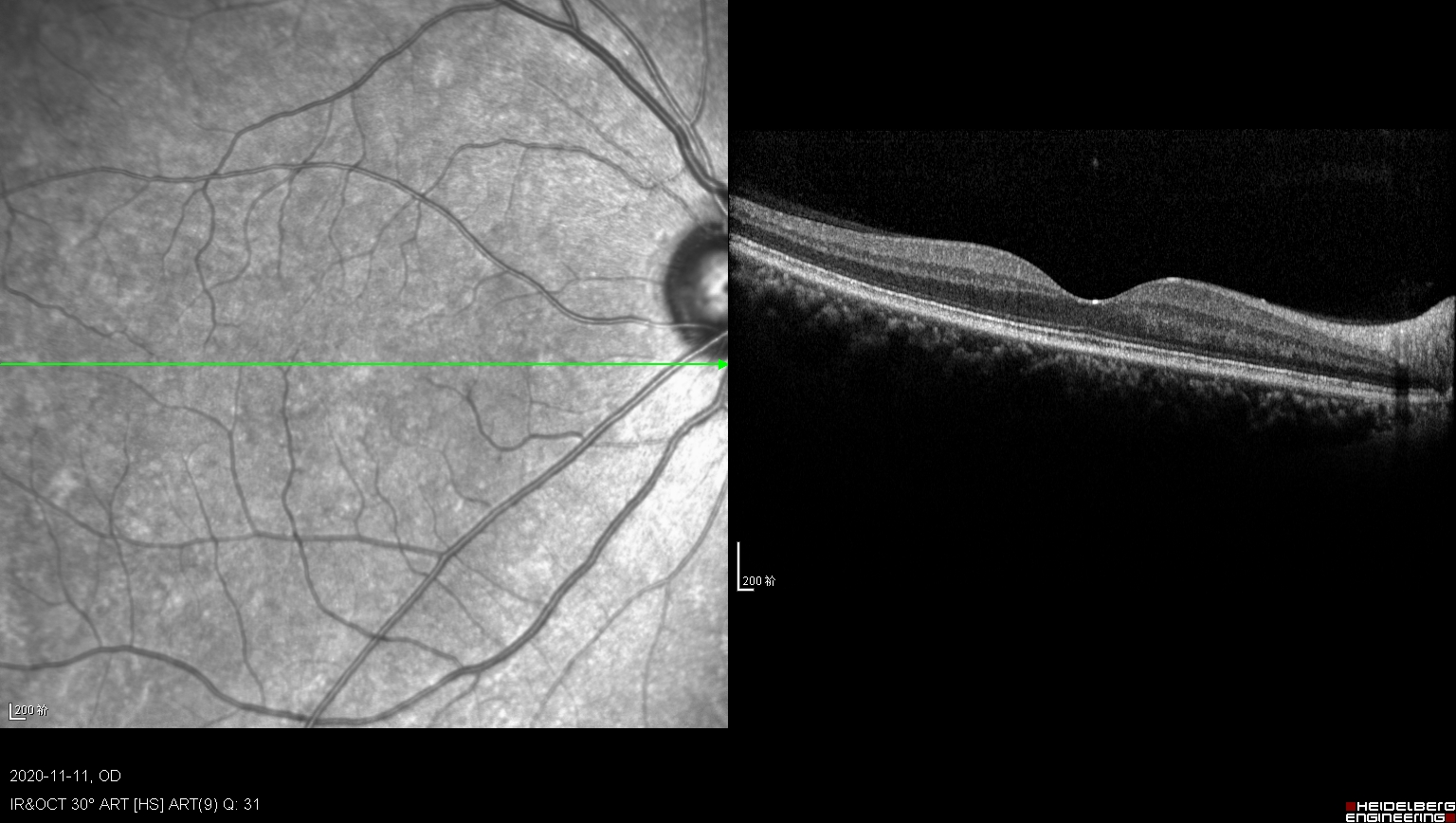

Supplement: Supplementary file 1 — Additional file 1: The raw data of this study. Table 1. The basic information of involved patients. [file 12886_2022_2598_MOESM1_ESM.zip › 1/OCTτ£╝σ║òτàoτ¢╕/20201111110046578_100002_801486_4835.jpg]

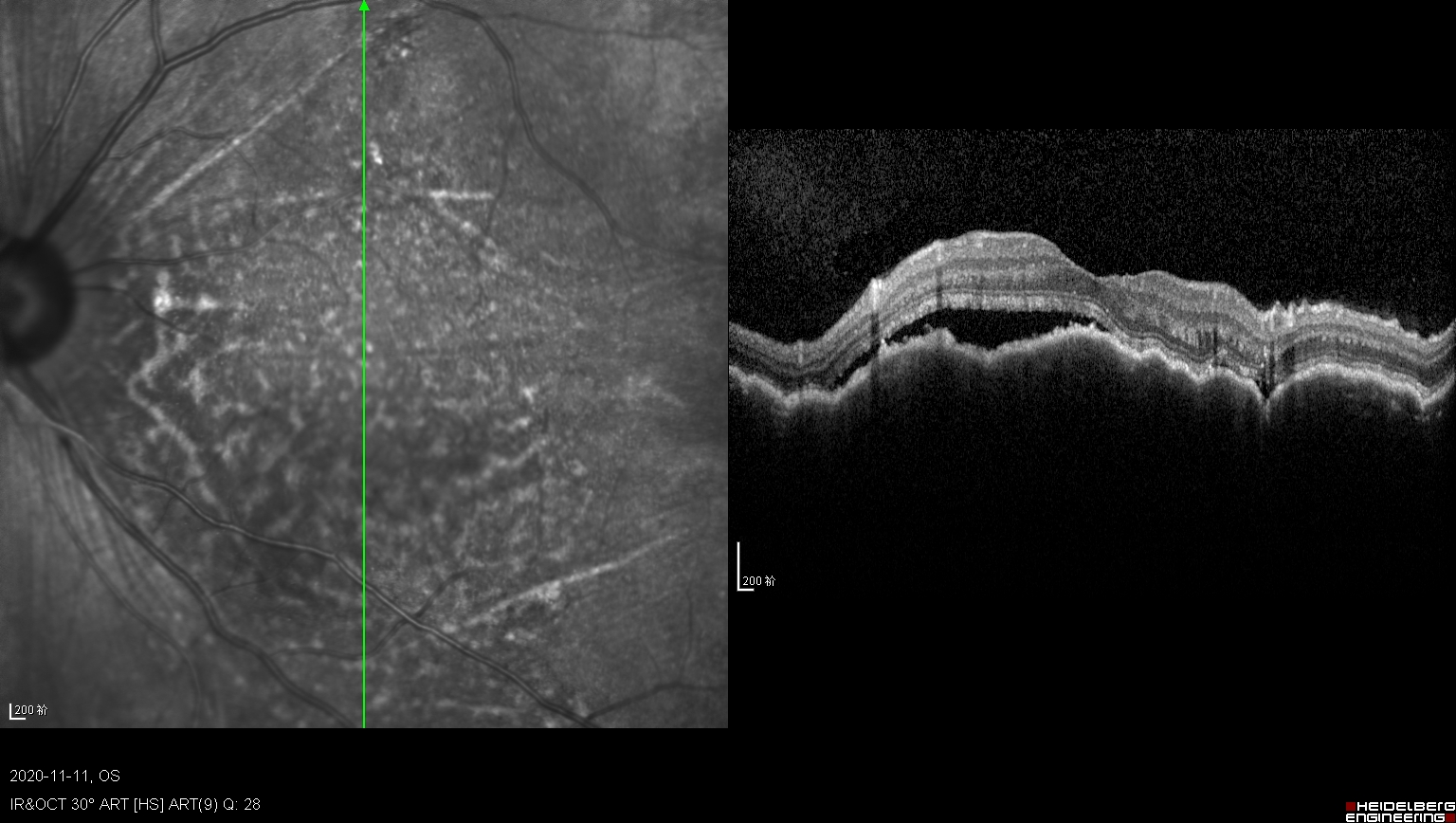

Supplement: Supplementary file 1 — Additional file 1: The raw data of this study. Table 1. The basic information of involved patients. [file 12886_2022_2598_MOESM1_ESM.zip › 1/OCTτ£╝σ║òτàoτ¢╕/20201111110048750_100002_801486_174.jpg]

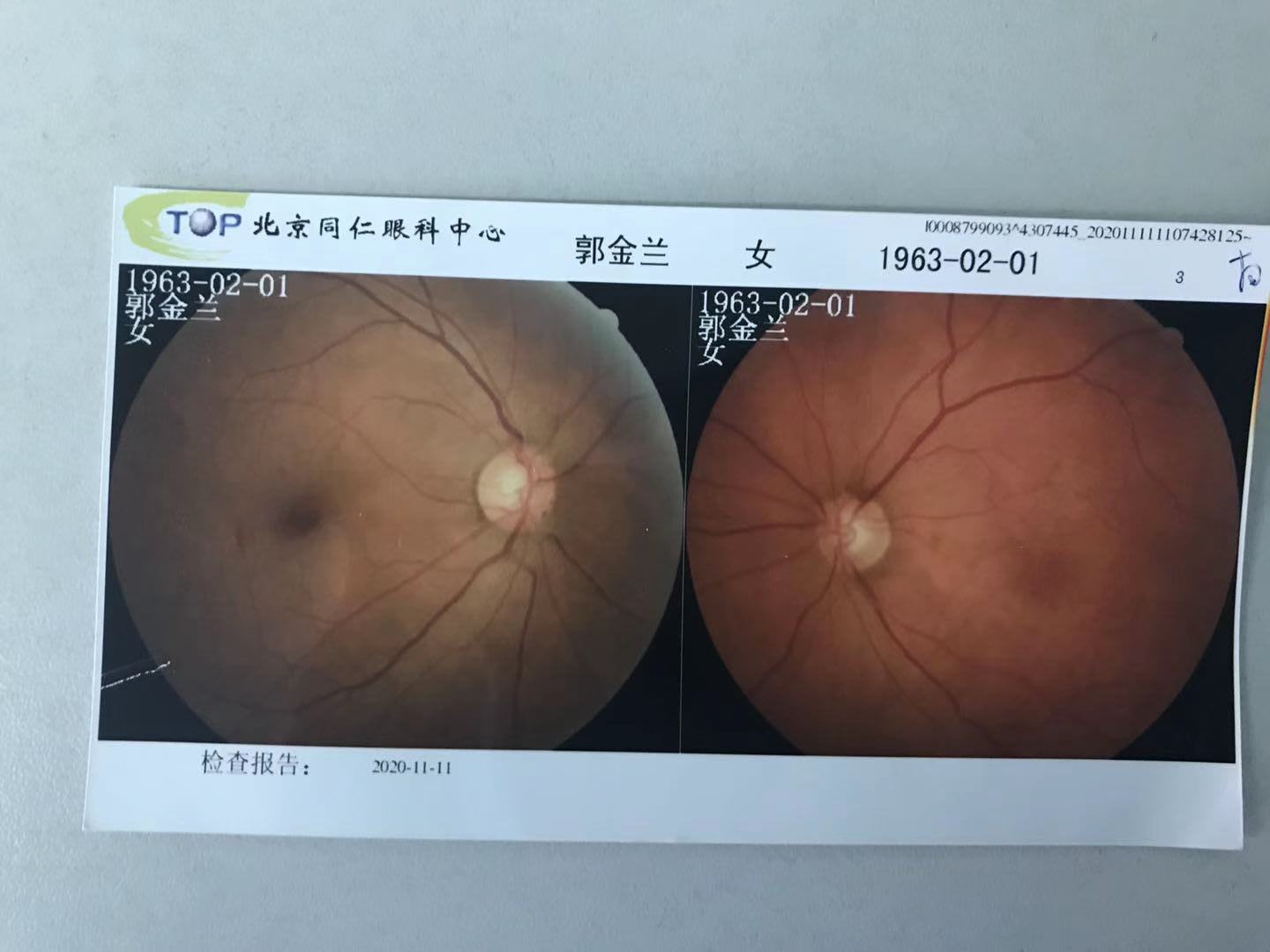

Supplement: Supplementary file 1 — Additional file 1: The raw data of this study. Table 1. The basic information of involved patients. [file 12886_2022_2598_MOESM1_ESM.zip › 1/τ£╝τoæτë╣μúÇ/20201111τ£╝σ║òτ¢╕1.jpg]

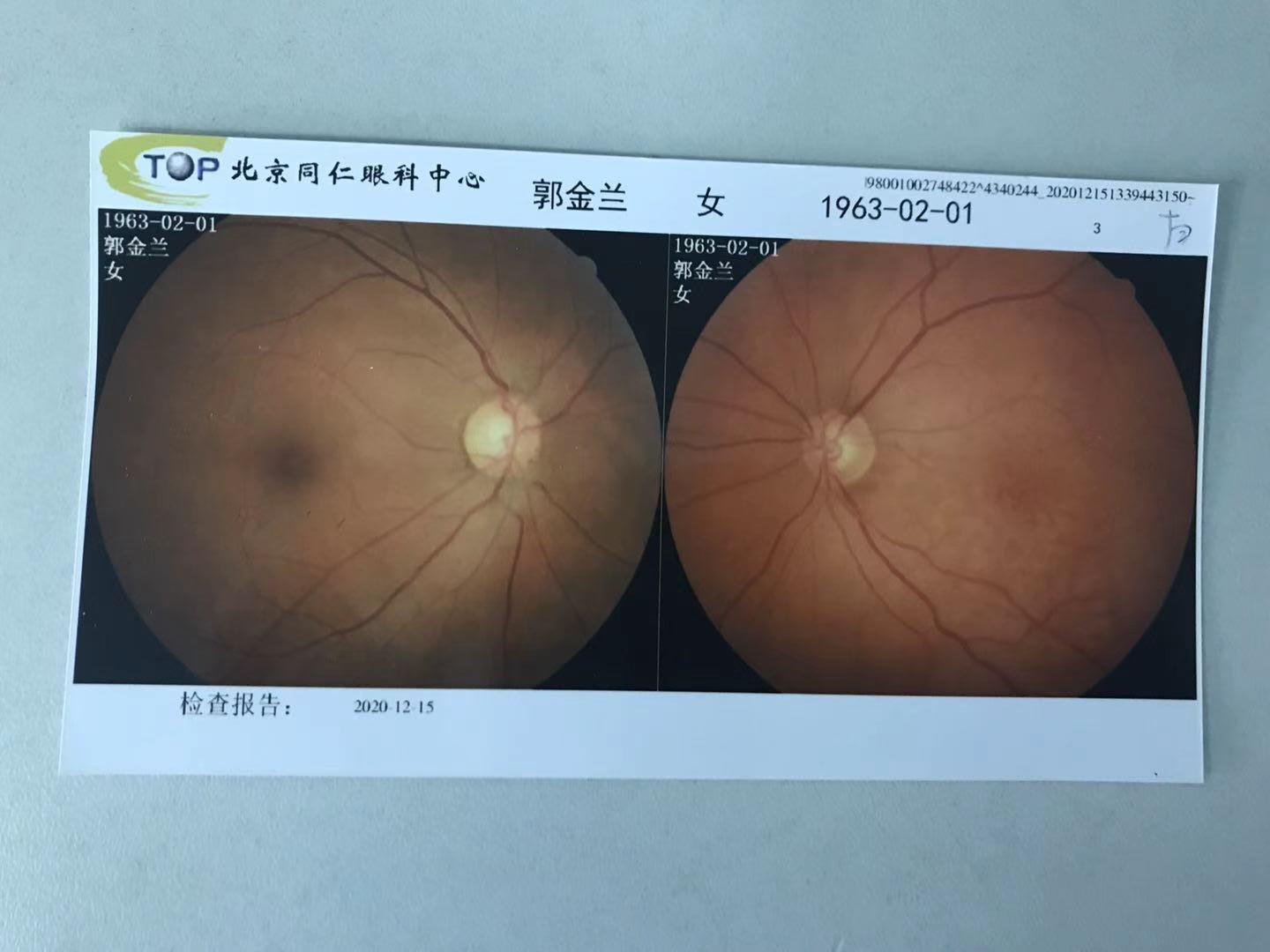

Supplement: Supplementary file 1 — Additional file 1: The raw data of this study. Table 1. The basic information of involved patients. [file 12886_2022_2598_MOESM1_ESM.zip › 1/τ£╝τoæτë╣μúÇ/20201215τ£╝σ║òτ¢╕1.jpg]

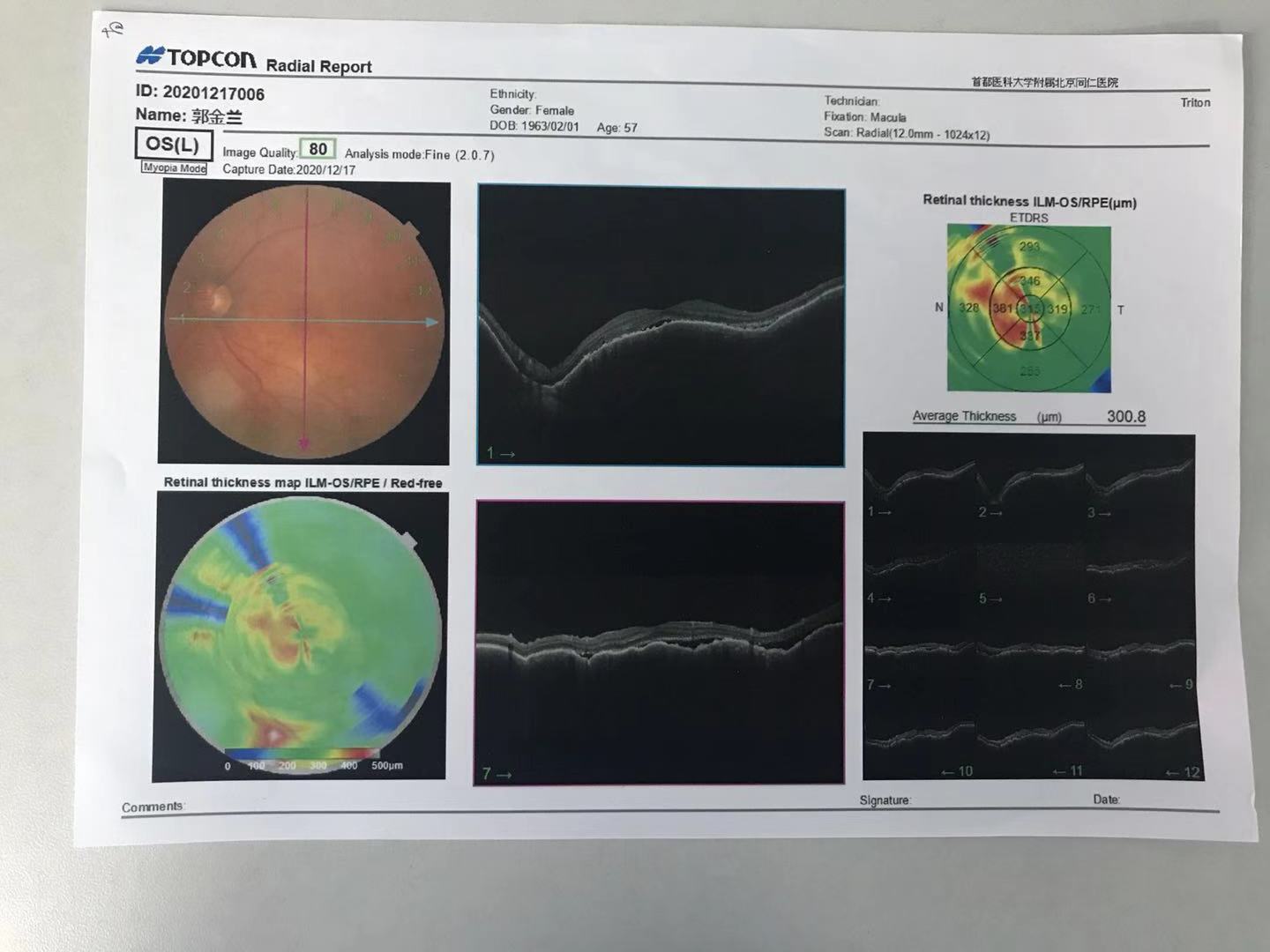

Supplement: Supplementary file 1 — Additional file 1: The raw data of this study. Table 1. The basic information of involved patients. [file 12886_2022_2598_MOESM1_ESM.zip › 1/τ£╝τoæτë╣μúÇ/20201217OCT2.jpg]

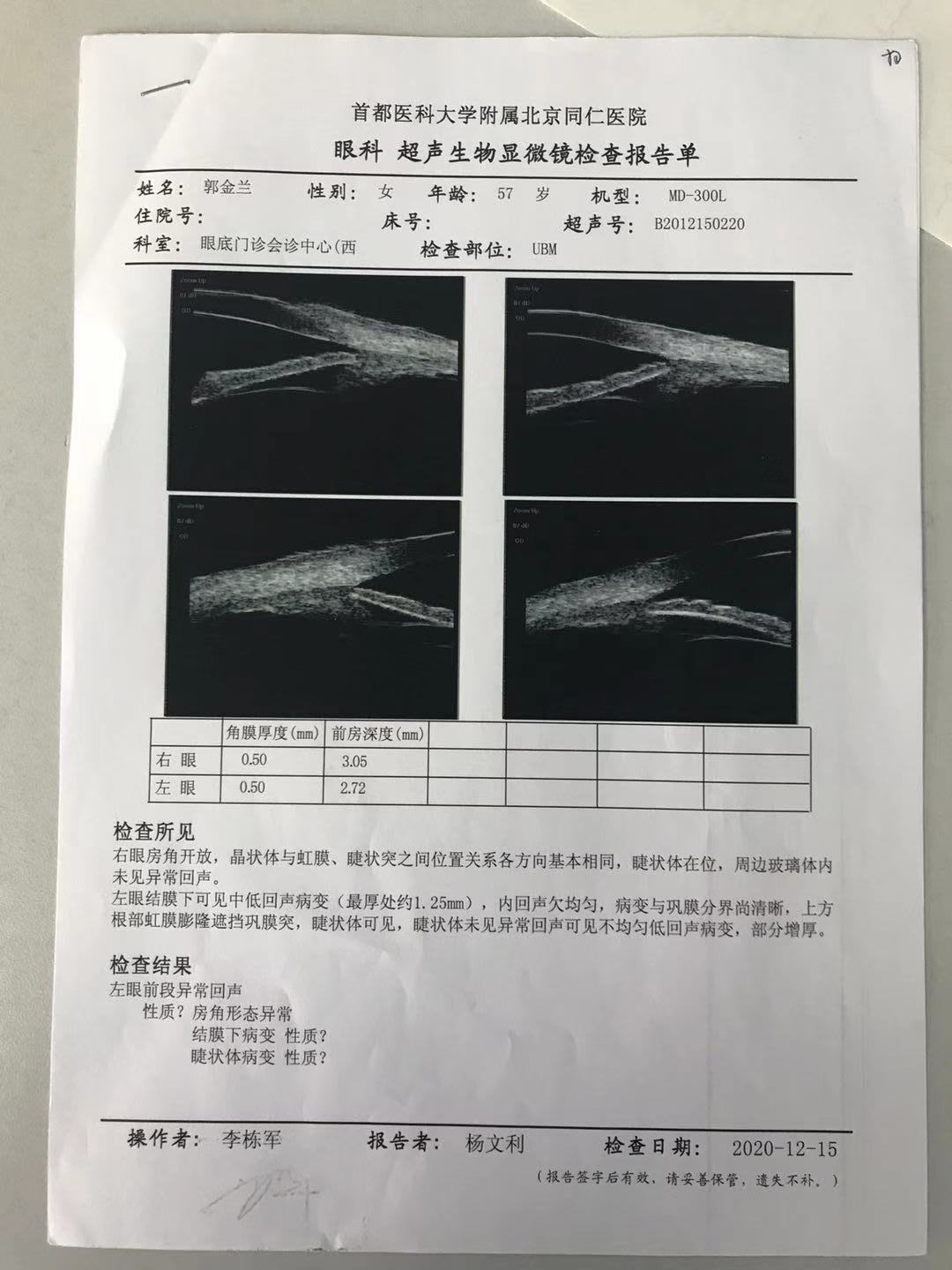

Supplement: Supplementary file 1 — Additional file 1: The raw data of this study. Table 1. The basic information of involved patients. [file 12886_2022_2598_MOESM1_ESM.zip › 1/τ£╝τoæτë╣μúÇ/20201215UBM1.jpg]

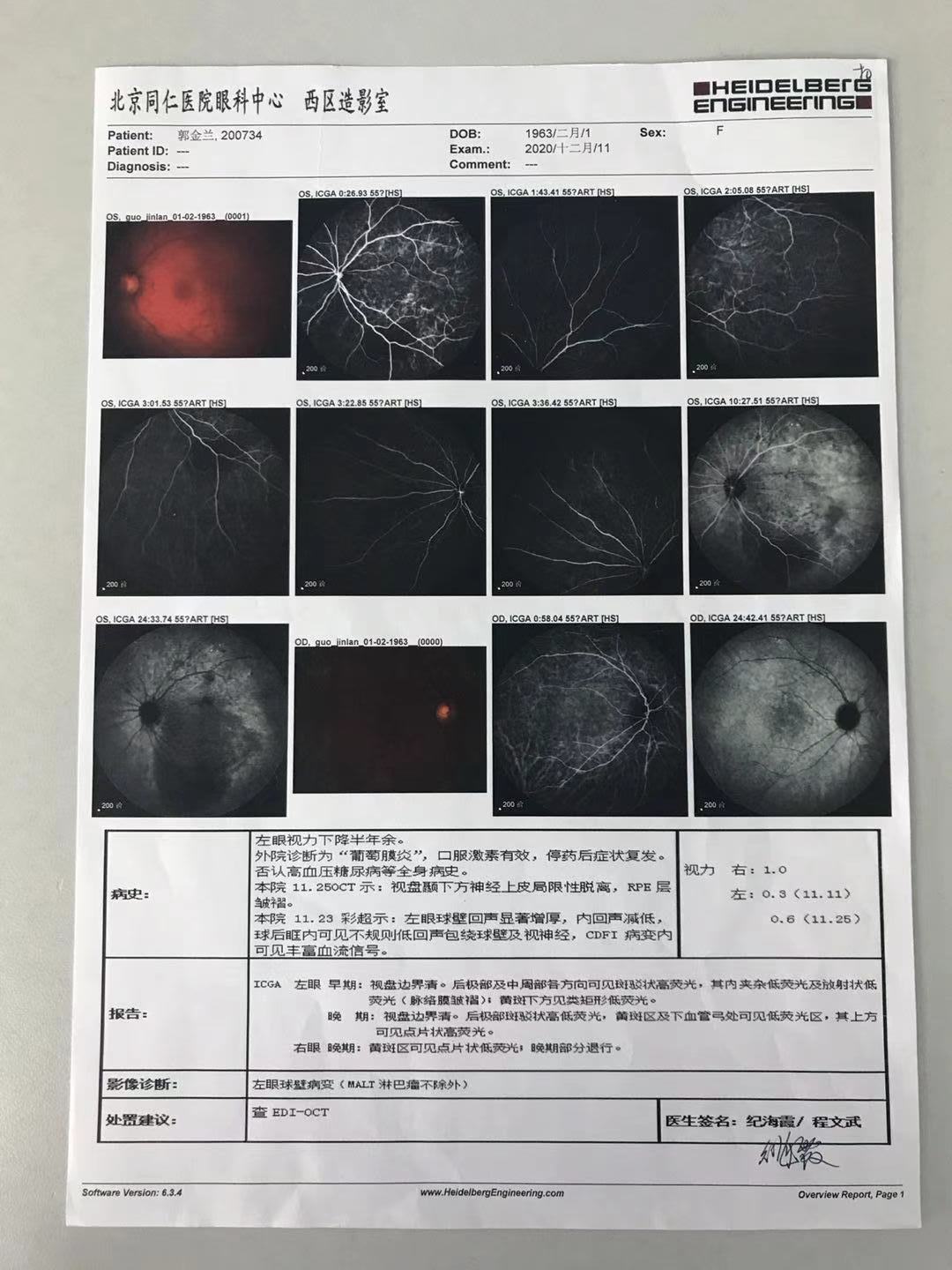

Supplement: Supplementary file 1 — Additional file 1: The raw data of this study. Table 1. The basic information of involved patients. [file 12886_2022_2598_MOESM1_ESM.zip › 1/τ£╝τoæτë╣μúÇ/20201211ICGA2.jpg]

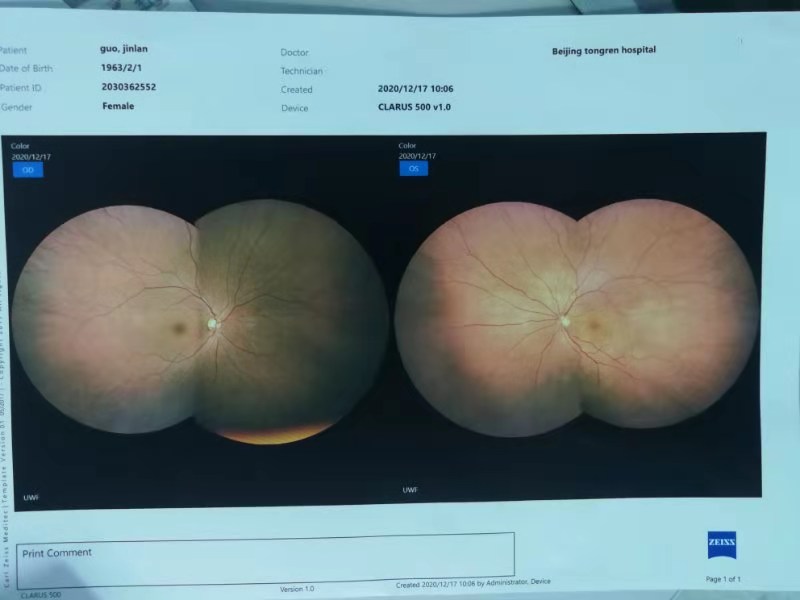

Supplement: Supplementary file 1 — Additional file 1: The raw data of this study. Table 1. The basic information of involved patients. [file 12886_2022_2598_MOESM1_ESM.zip › 1/τ£╝τoæτë╣μúÇ/20201217τ£╝σ║òτ¢╕.jpg]

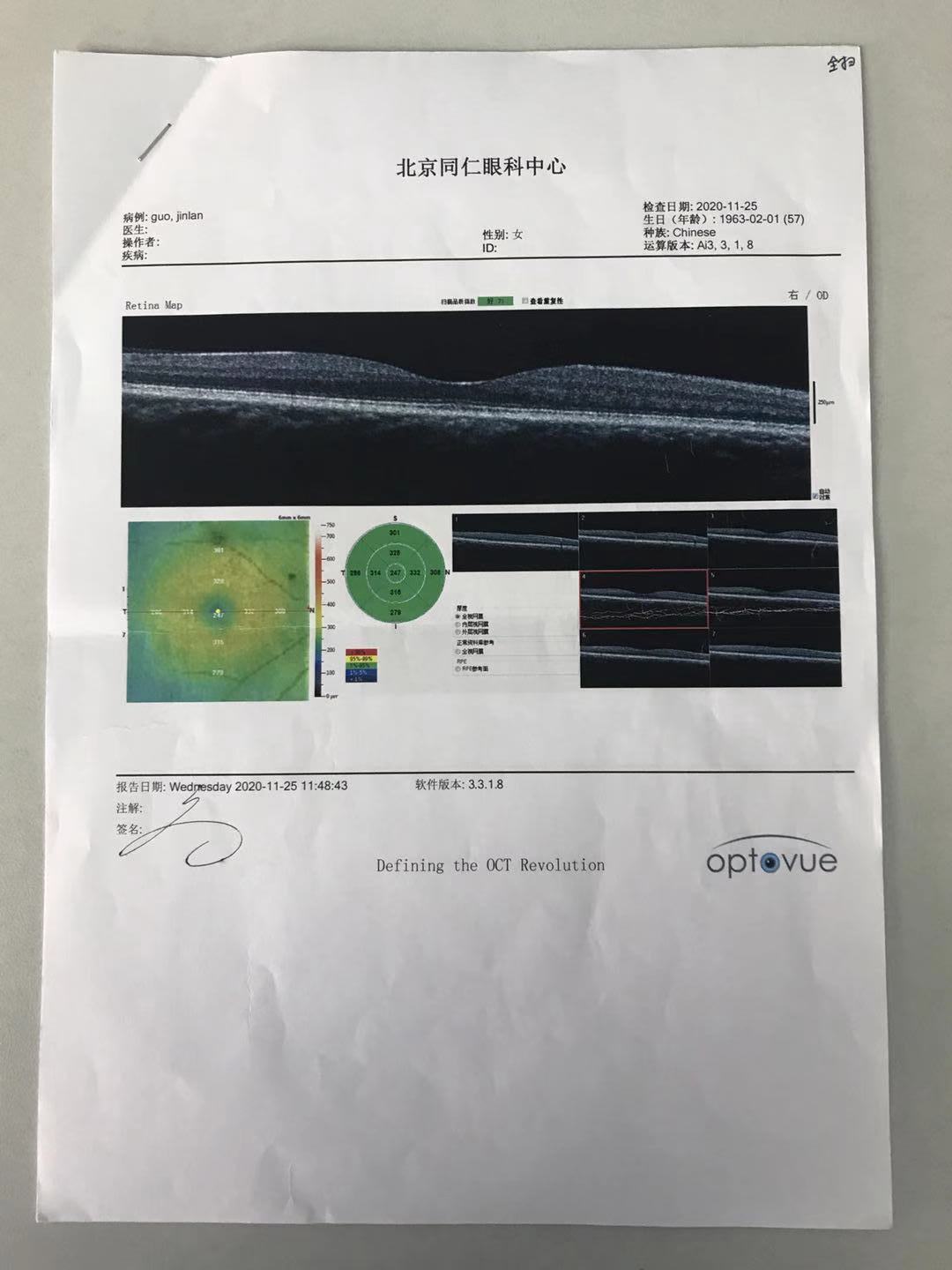

Supplement: Supplementary file 1 — Additional file 1: The raw data of this study. Table 1. The basic information of involved patients. [file 12886_2022_2598_MOESM1_ESM.zip › 1/τ£╝τoæτë╣μúÇ/20201125OCT2.jpg]

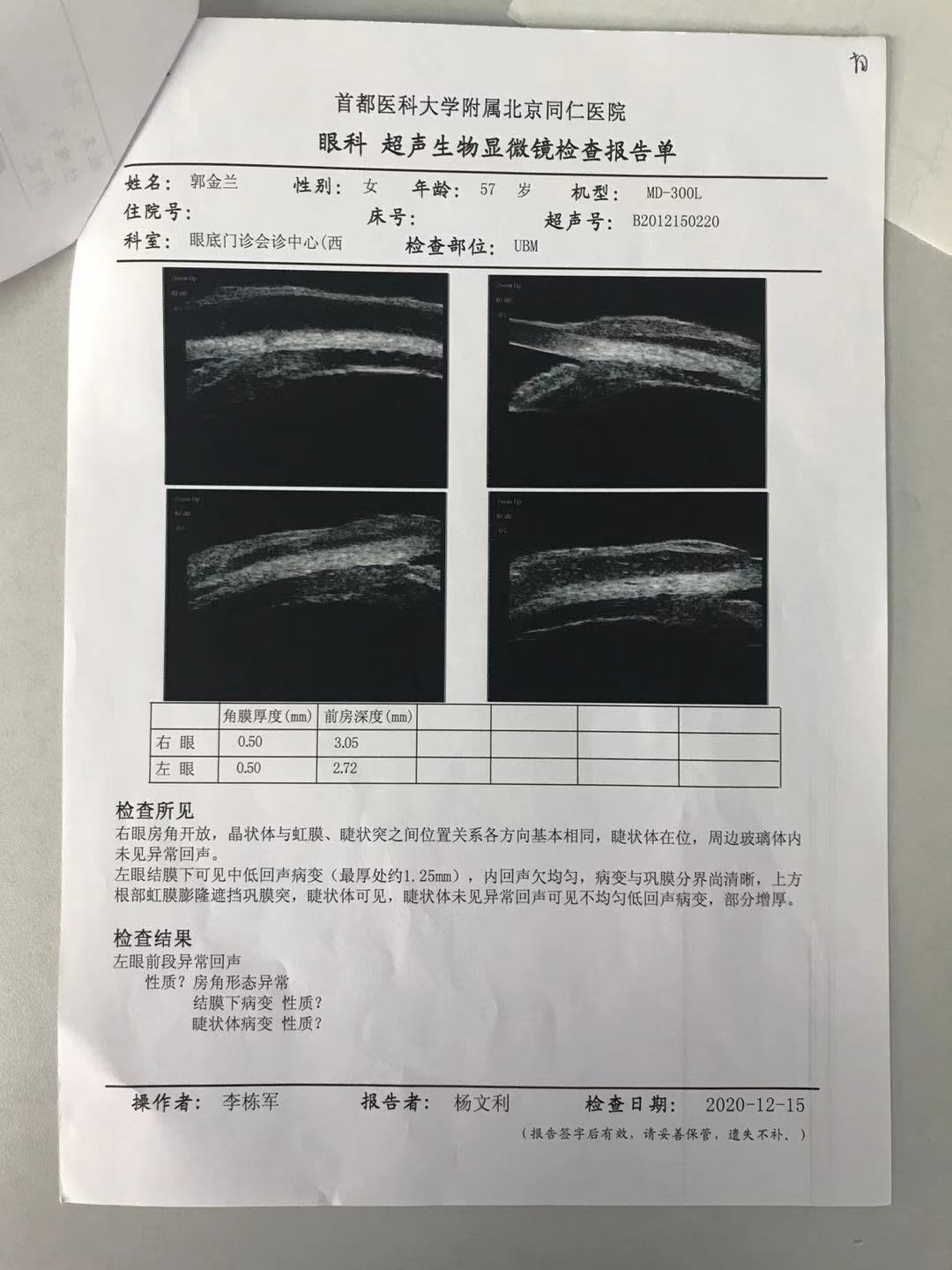

Supplement: Supplementary file 1 — Additional file 1: The raw data of this study. Table 1. The basic information of involved patients. [file 12886_2022_2598_MOESM1_ESM.zip › 1/τ£╝τoæτë╣μúÇ/20201215UBM2.jpg]

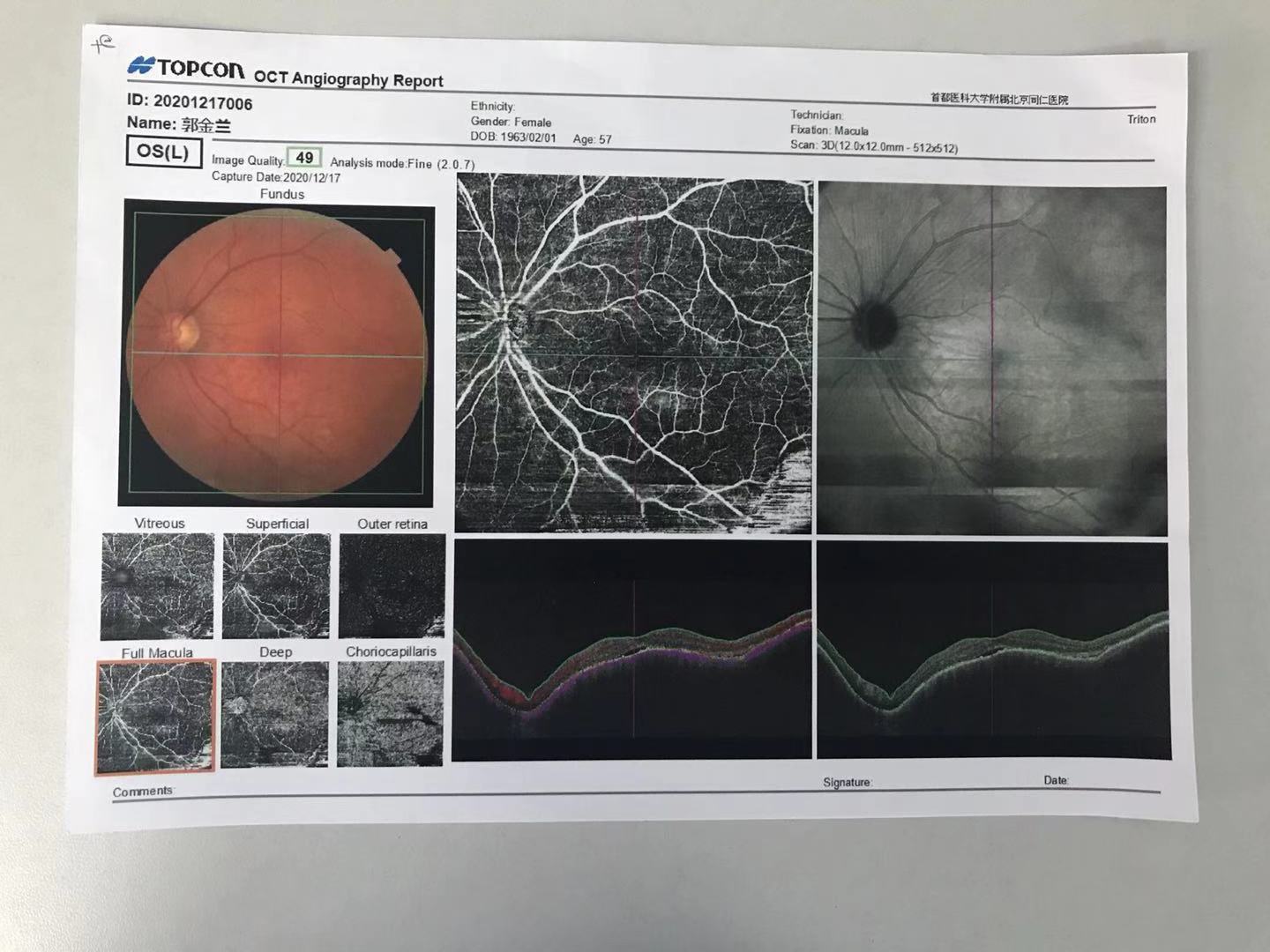

Supplement: Supplementary file 1 — Additional file 1: The raw data of this study. Table 1. The basic information of involved patients. [file 12886_2022_2598_MOESM1_ESM.zip › 1/τ£╝τoæτë╣μúÇ/20201217OCTA.jpg]

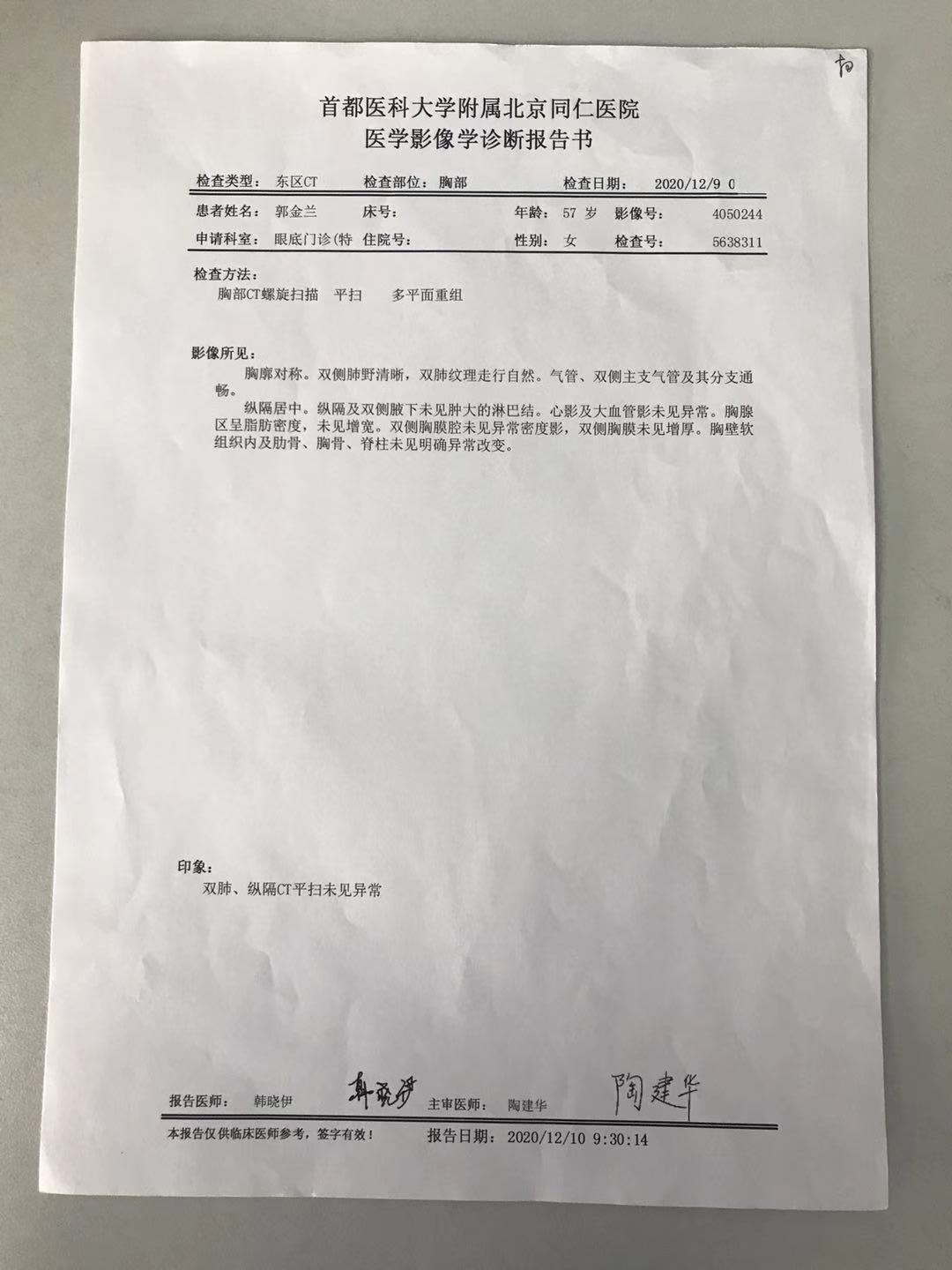

Supplement: Supplementary file 1 — Additional file 1: The raw data of this study. Table 1. The basic information of involved patients. [file 12886_2022_2598_MOESM1_ESM.zip › 1/τ£╝τoæτë╣μúÇ/20201209Φâ╕Θâ¿CT.jpg]

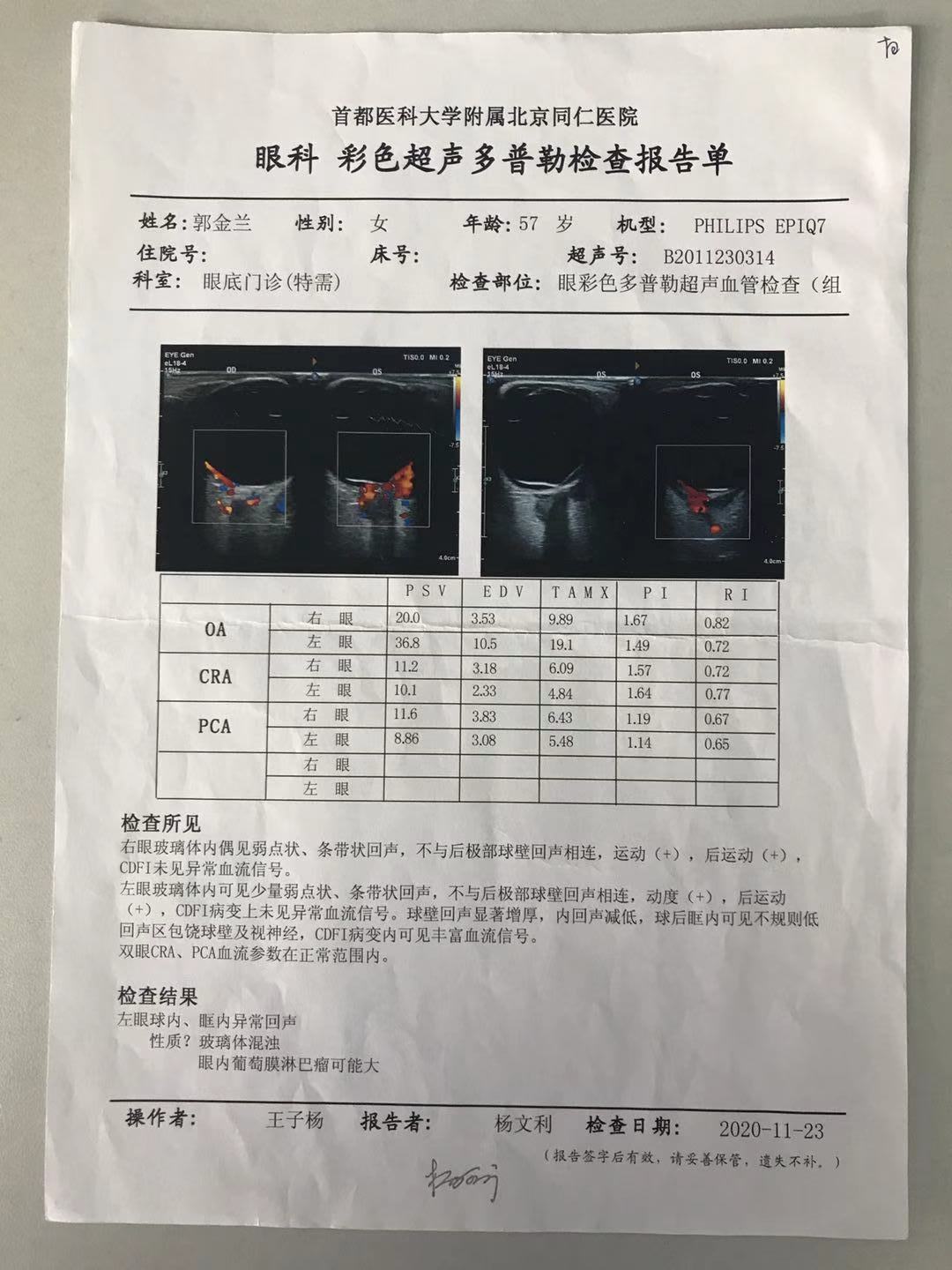

Supplement: Supplementary file 1 — Additional file 1: The raw data of this study. Table 1. The basic information of involved patients. [file 12886_2022_2598_MOESM1_ESM.zip › 1/τ£╝τoæτë╣μúÇ/20201123BΦ╢à.jpg]

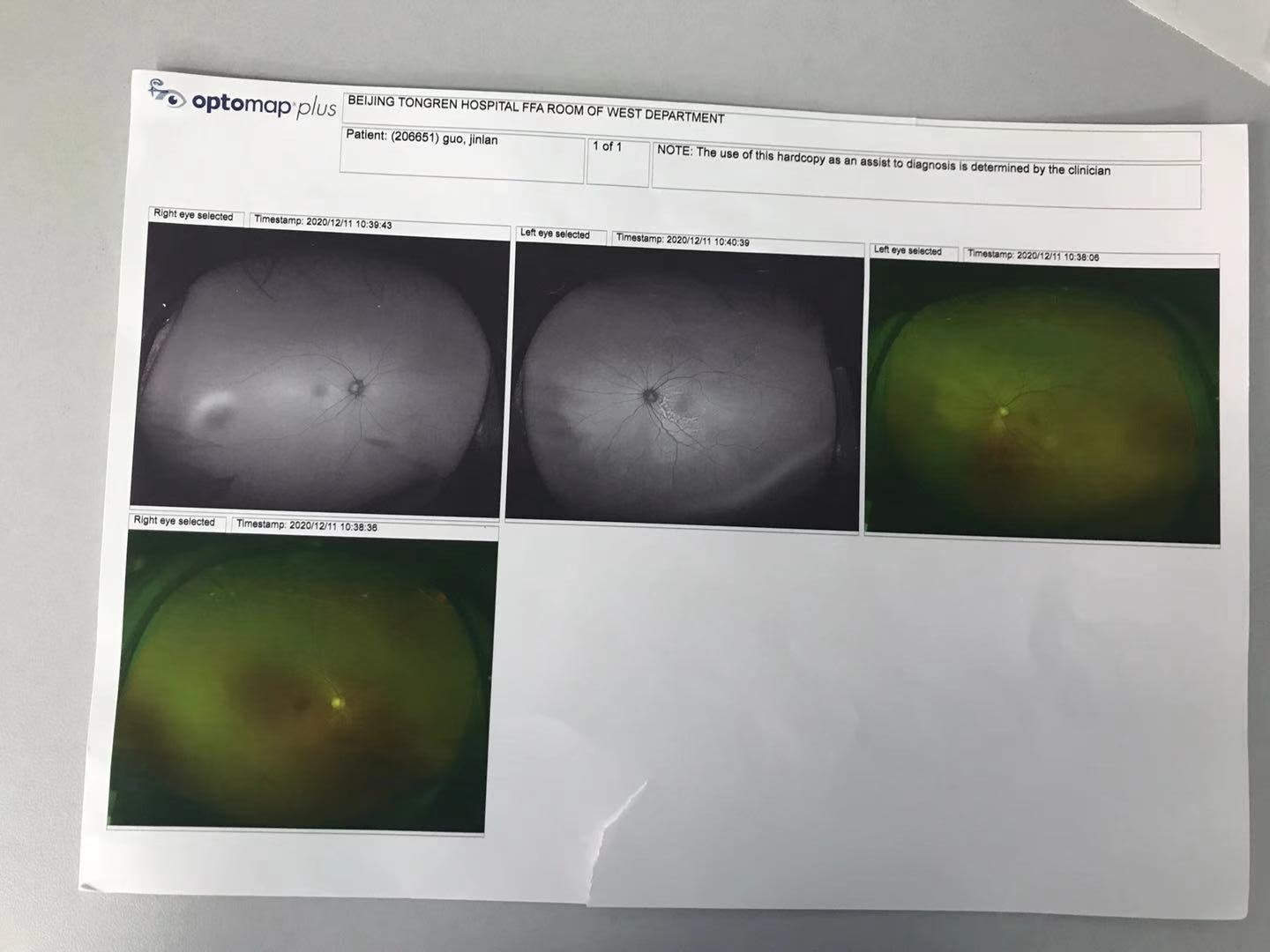

Supplement: Supplementary file 1 — Additional file 1: The raw data of this study. Table 1. The basic information of involved patients. [file 12886_2022_2598_MOESM1_ESM.zip › 1/τ£╝τoæτë╣μúÇ/20201211μ1⁄4oσáí.jpg]

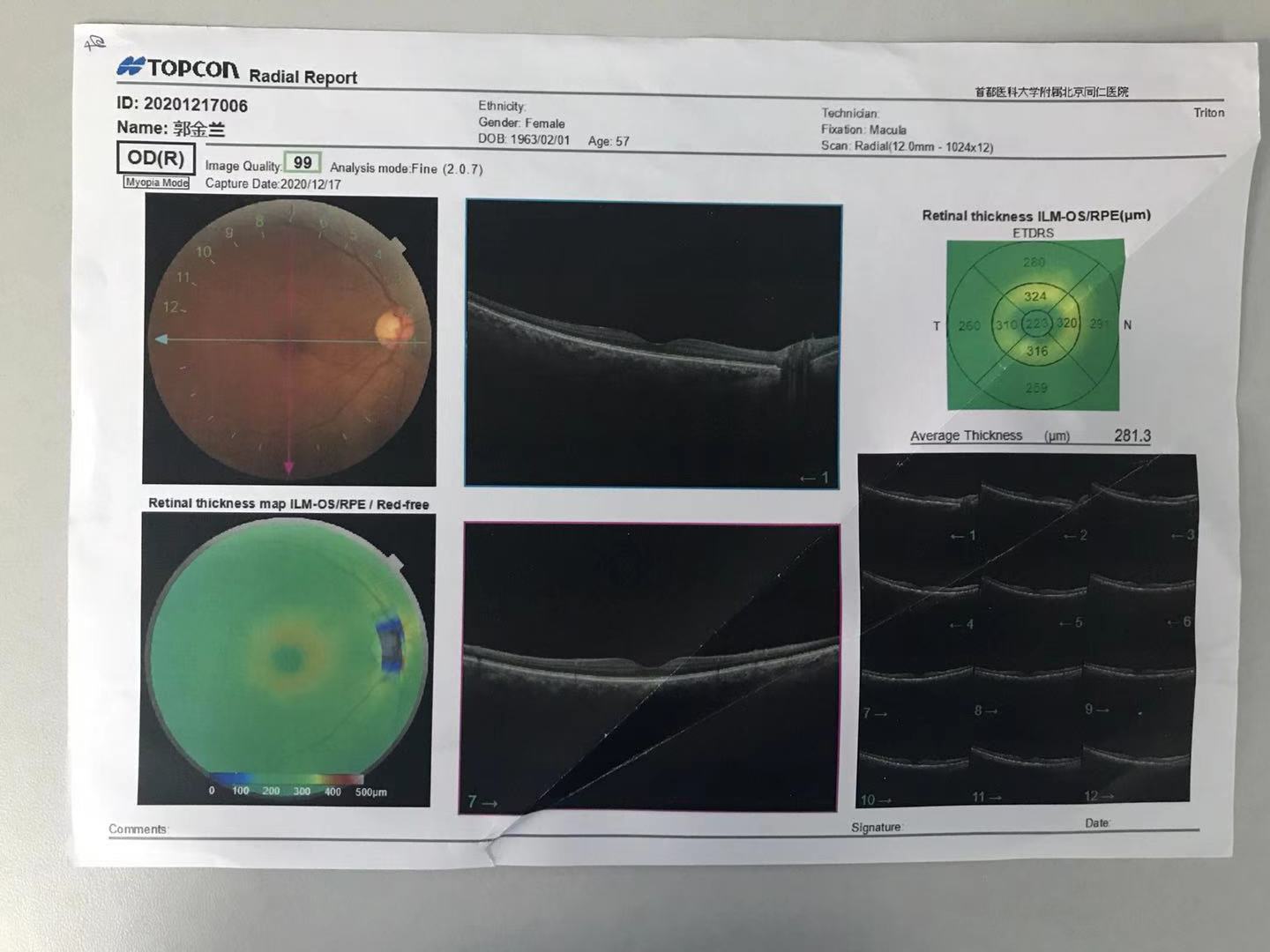

Supplement: Supplementary file 1 — Additional file 1: The raw data of this study. Table 1. The basic information of involved patients. [file 12886_2022_2598_MOESM1_ESM.zip › 1/τ£╝τoæτë╣μúÇ/20201217OCT.jpg]

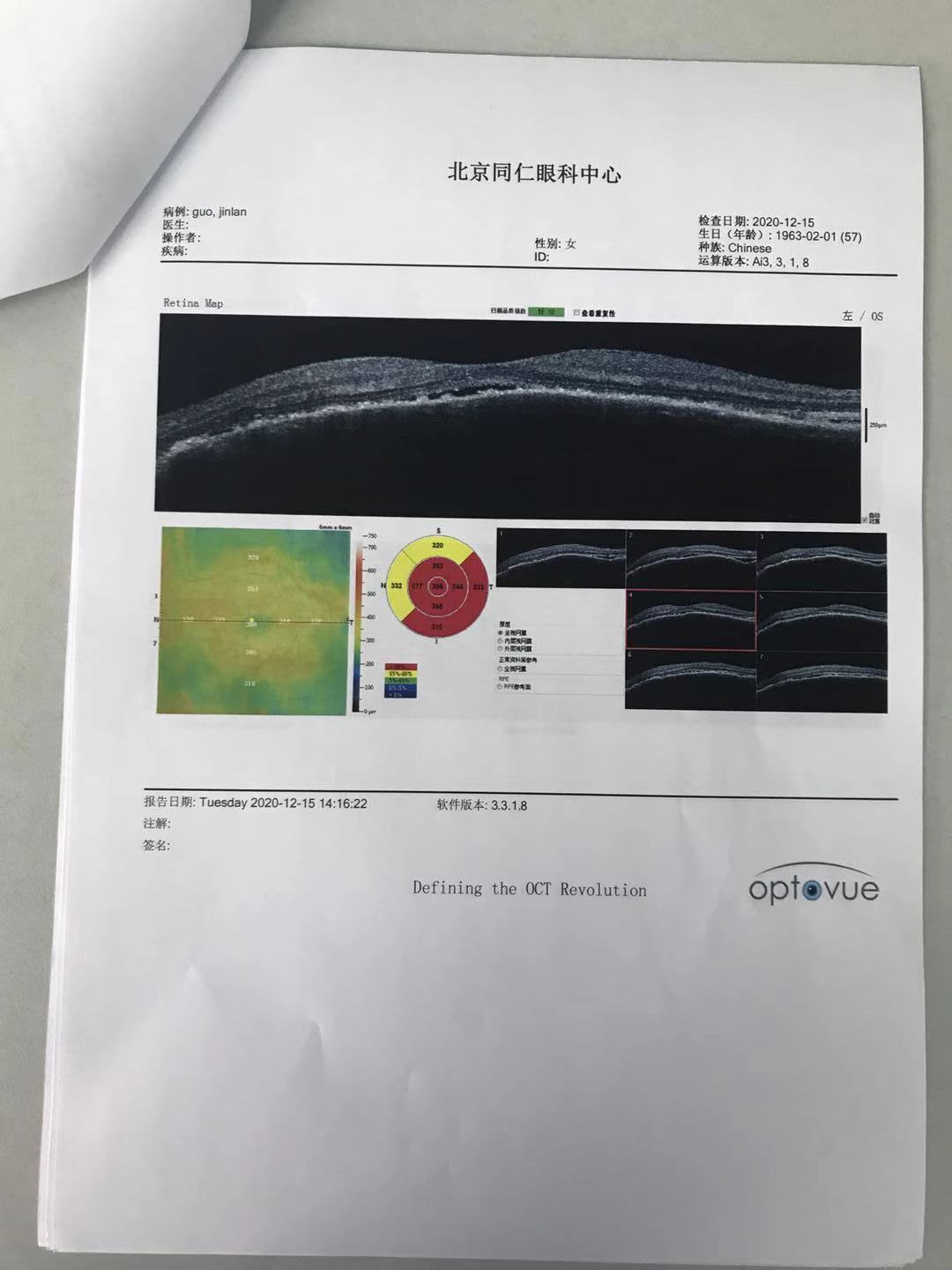

Supplement: Supplementary file 1 — Additional file 1: The raw data of this study. Table 1. The basic information of involved patients. [file 12886_2022_2598_MOESM1_ESM.zip › 1/τ£╝τoæτë╣μúÇ/20201215OCT.jpg]

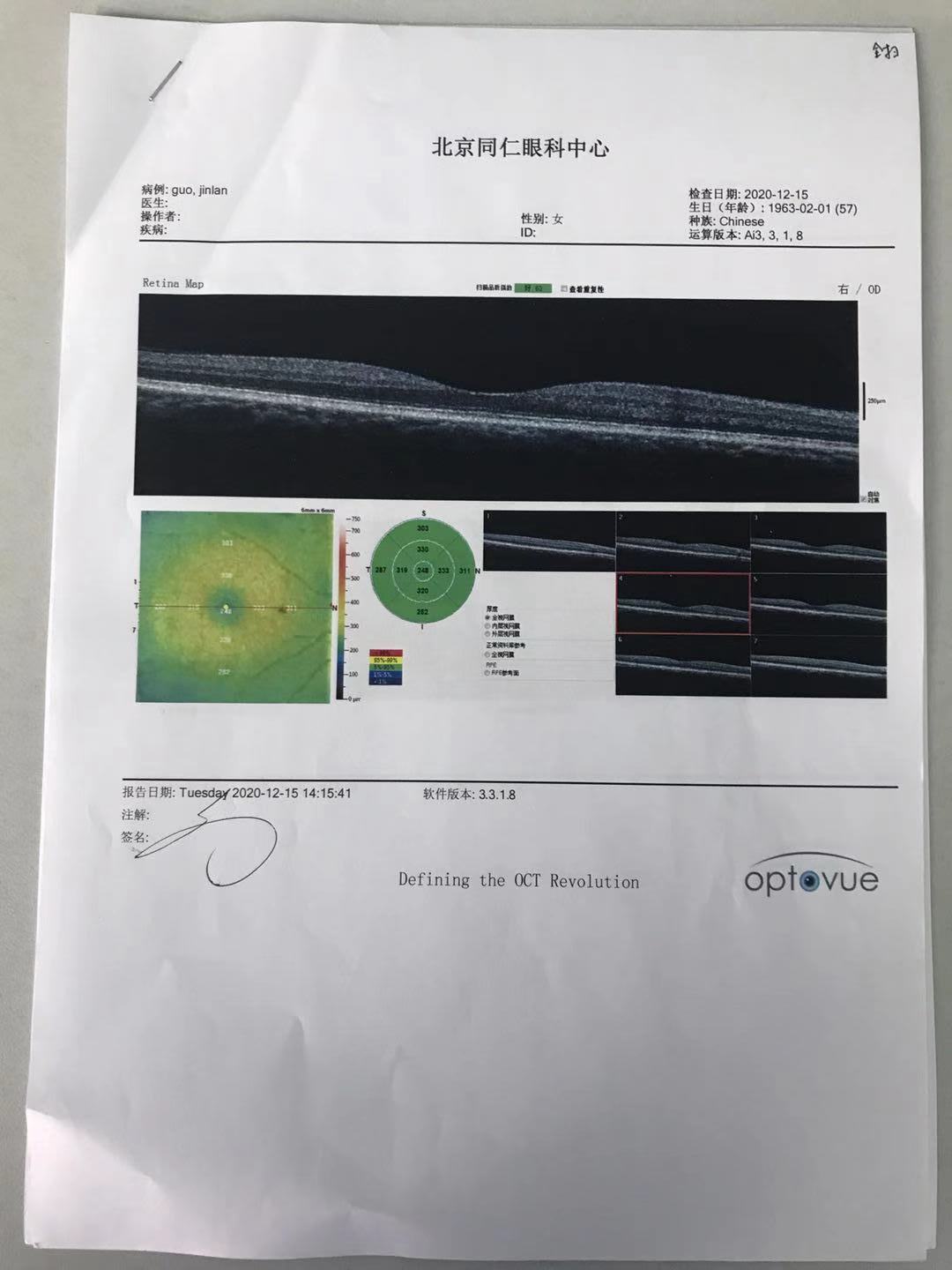

Supplement: Supplementary file 1 — Additional file 1: The raw data of this study. Table 1. The basic information of involved patients. [file 12886_2022_2598_MOESM1_ESM.zip › 1/τ£╝τoæτë╣μúÇ/20201215OCT2.jpg]

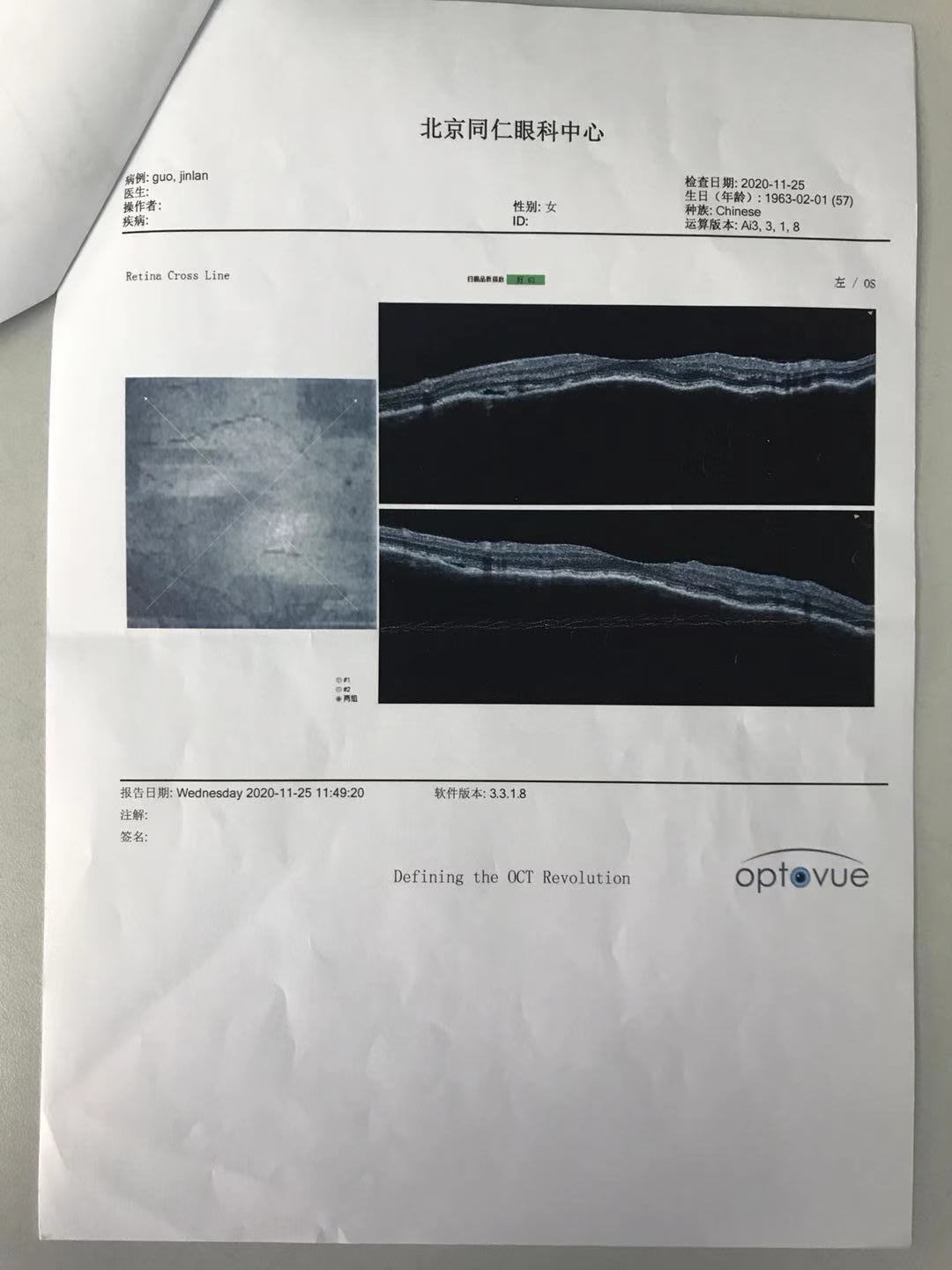

Supplement: Supplementary file 1 — Additional file 1: The raw data of this study. Table 1. The basic information of involved patients. [file 12886_2022_2598_MOESM1_ESM.zip › 1/τ£╝τoæτë╣μúÇ/20201125OCT.jpg]

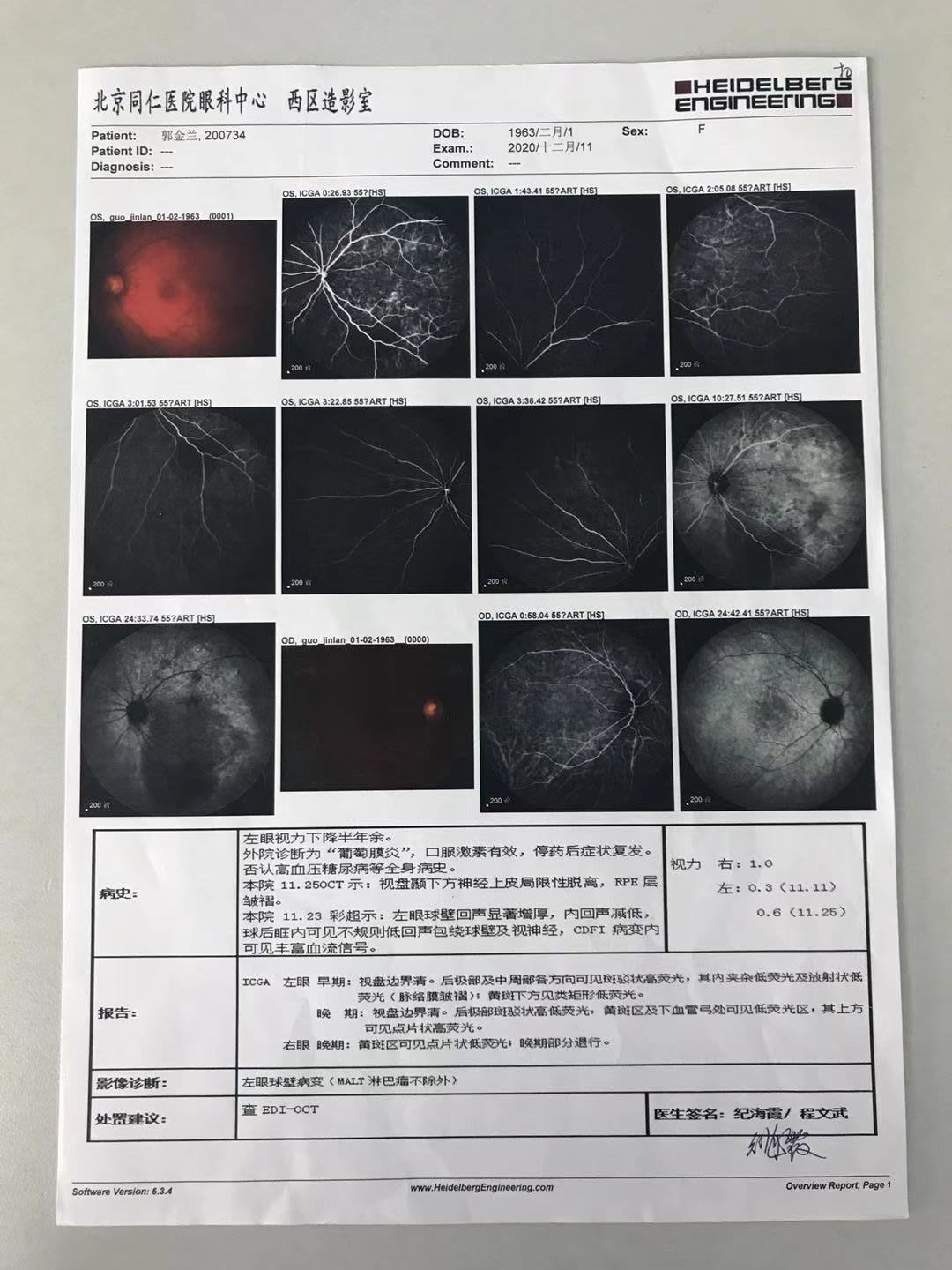

Supplement: Supplementary file 1 — Additional file 1: The raw data of this study. Table 1. The basic information of involved patients. [file 12886_2022_2598_MOESM1_ESM.zip › 1/τ£╝τoæτë╣μúÇ/20201211ICGA.jpg]

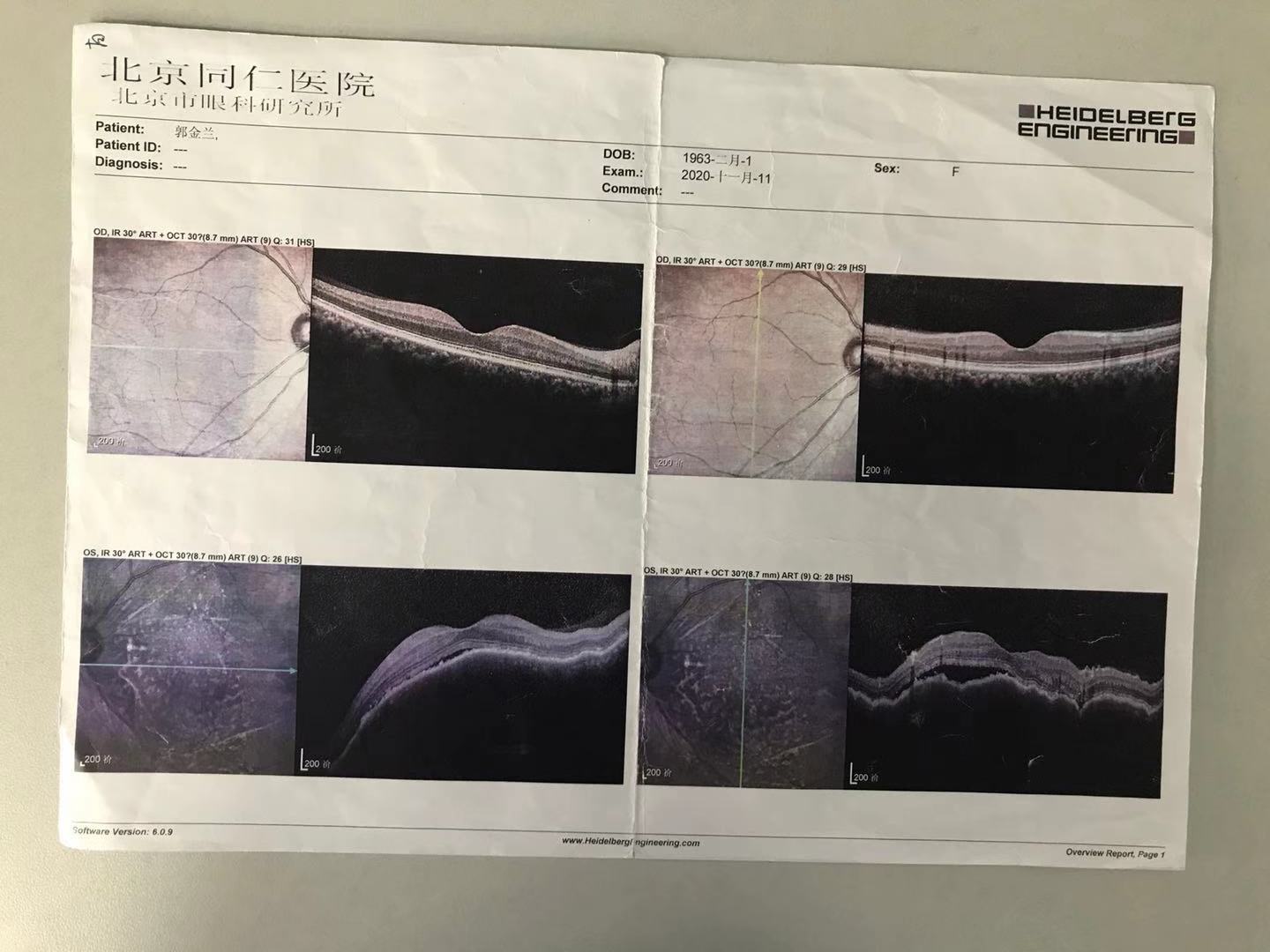

Supplement: Supplementary file 1 — Additional file 1: The raw data of this study. Table 1. The basic information of involved patients. [file 12886_2022_2598_MOESM1_ESM.zip › 1/τ£╝τoæτë╣μúÇ/20201111OCT.jpg]

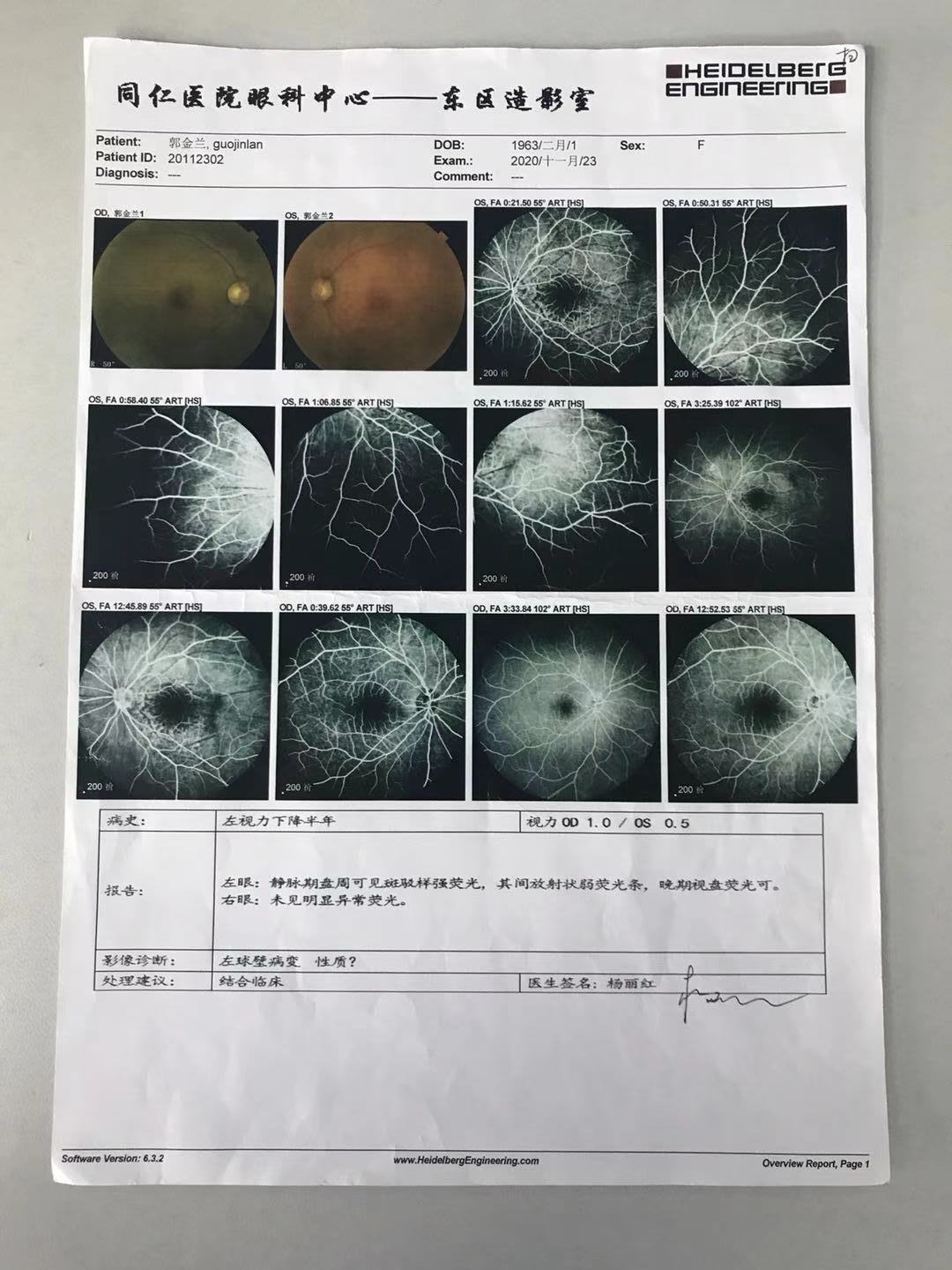

Supplement: Supplementary file 1 — Additional file 1: The raw data of this study. Table 1. The basic information of involved patients. [file 12886_2022_2598_MOESM1_ESM.zip › 1/τ£╝τoæτë╣μúÇ/20201123FFA.jpg]

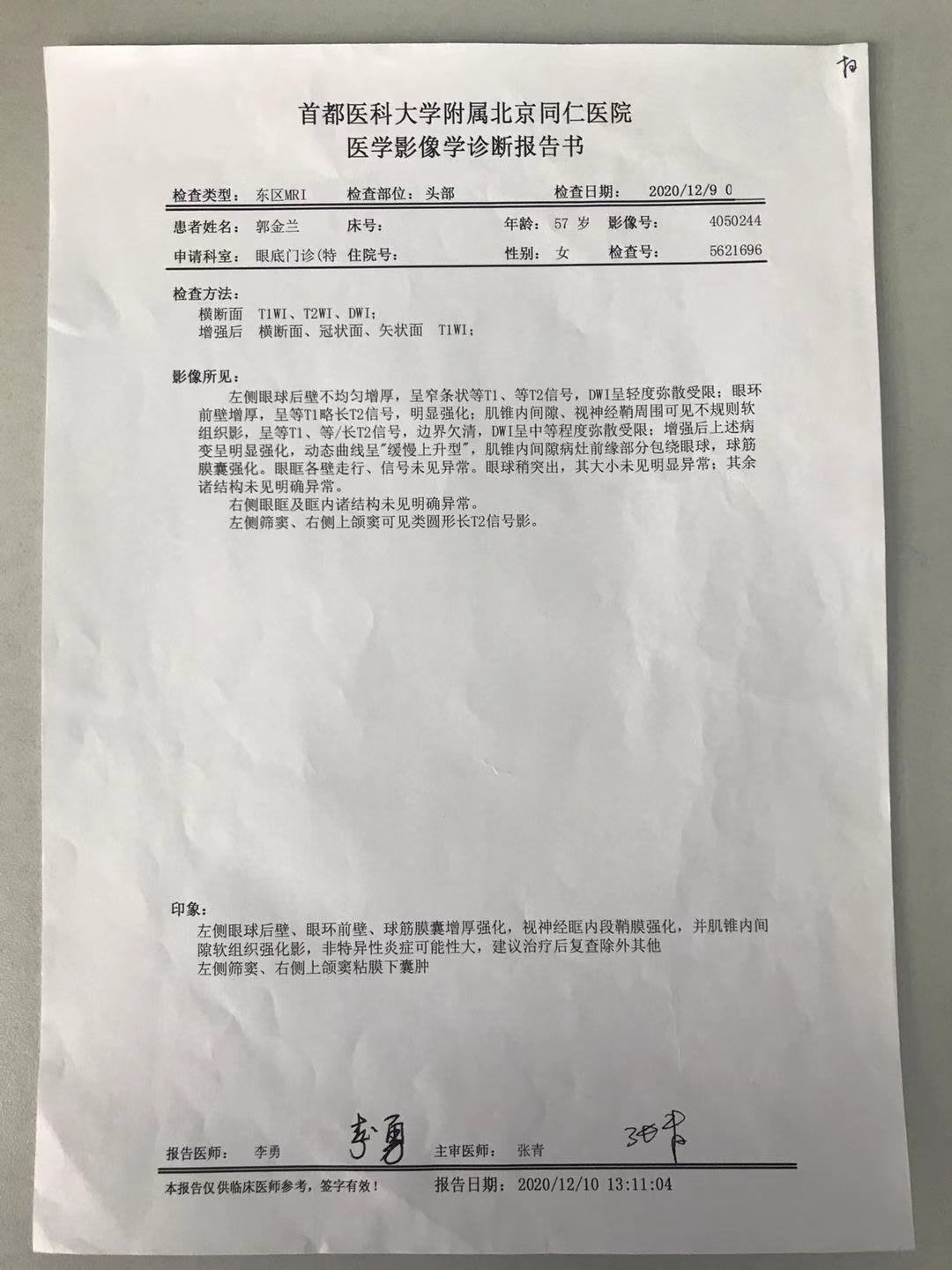

Supplement: Supplementary file 1 — Additional file 1: The raw data of this study. Table 1. The basic information of involved patients. [file 12886_2022_2598_MOESM1_ESM.zip › 1/τ£╝τoæτë╣μúÇ/20201209MRI1.jpg]

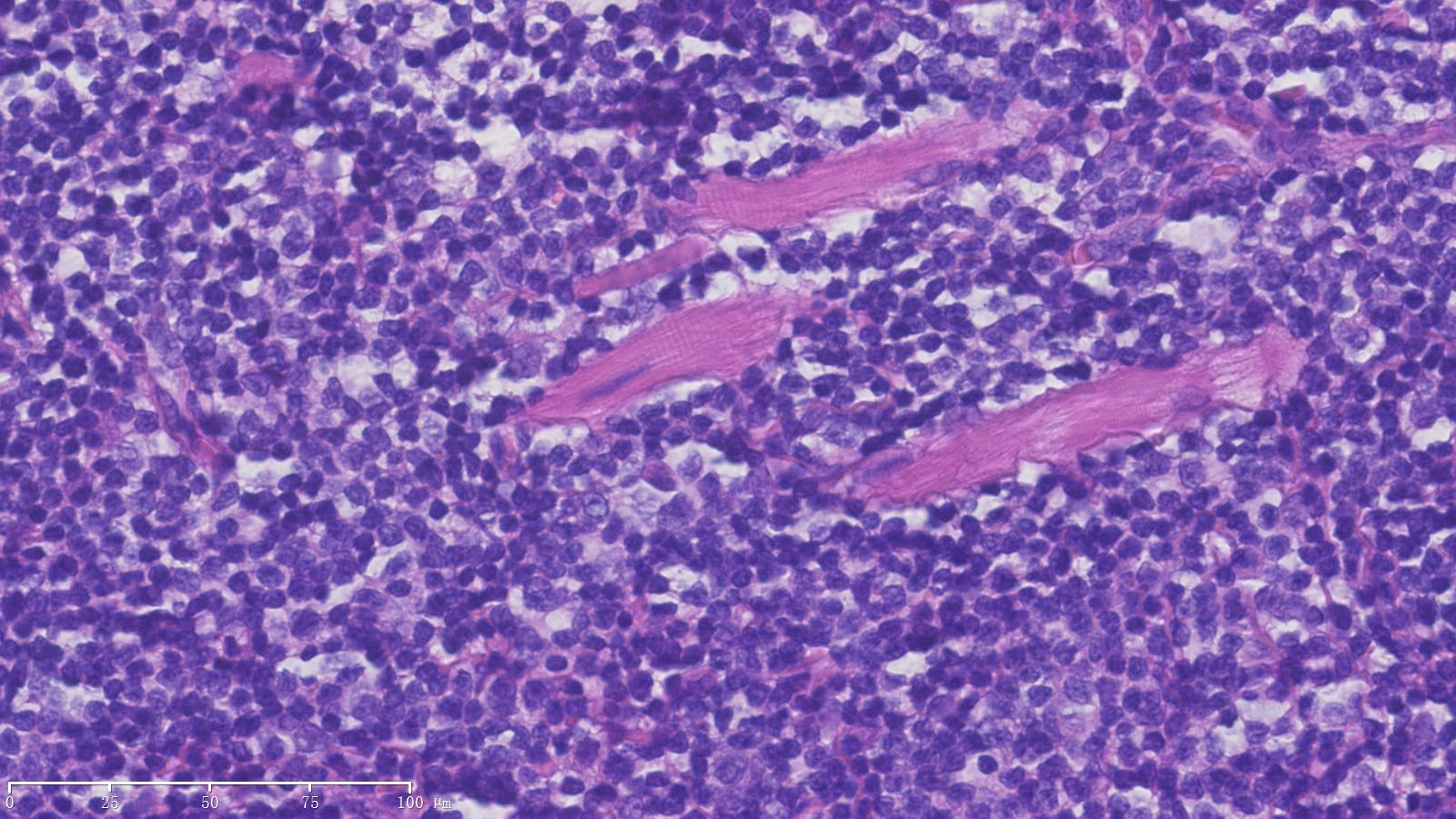

Supplement: Supplementary file 1 — Additional file 1: The raw data of this study. Table 1. The basic information of involved patients. [file 12886_2022_2598_MOESM1_ESM.zip › 1/Θâ¡Θçæσà░τùàτÉå/WechatIMG164.jpeg]

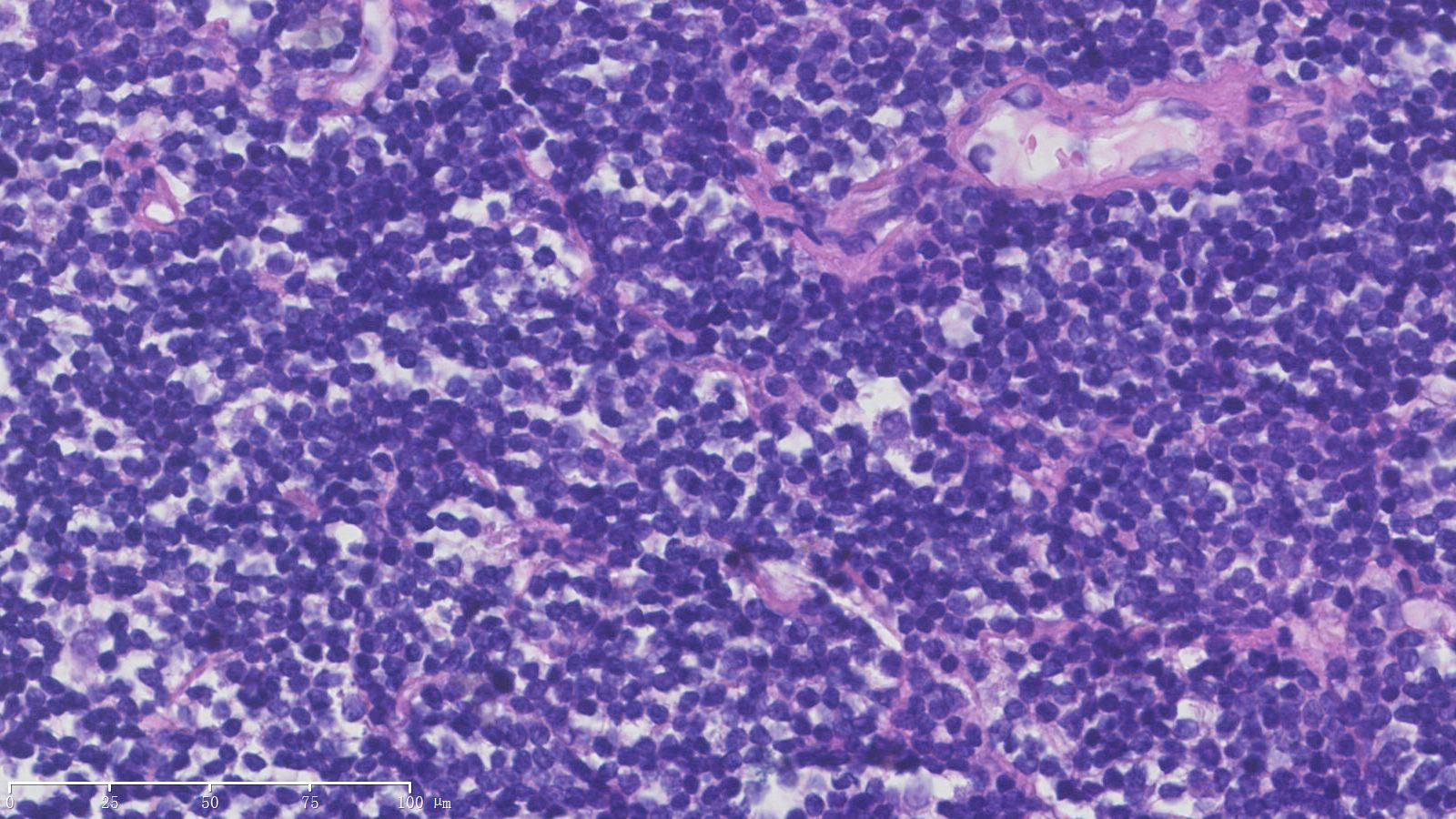

Supplement: Supplementary file 1 — Additional file 1: The raw data of this study. Table 1. The basic information of involved patients. [file 12886_2022_2598_MOESM1_ESM.zip › 1/Θâ¡Θçæσà░τùàτÉå/WechatIMG163.jpeg]

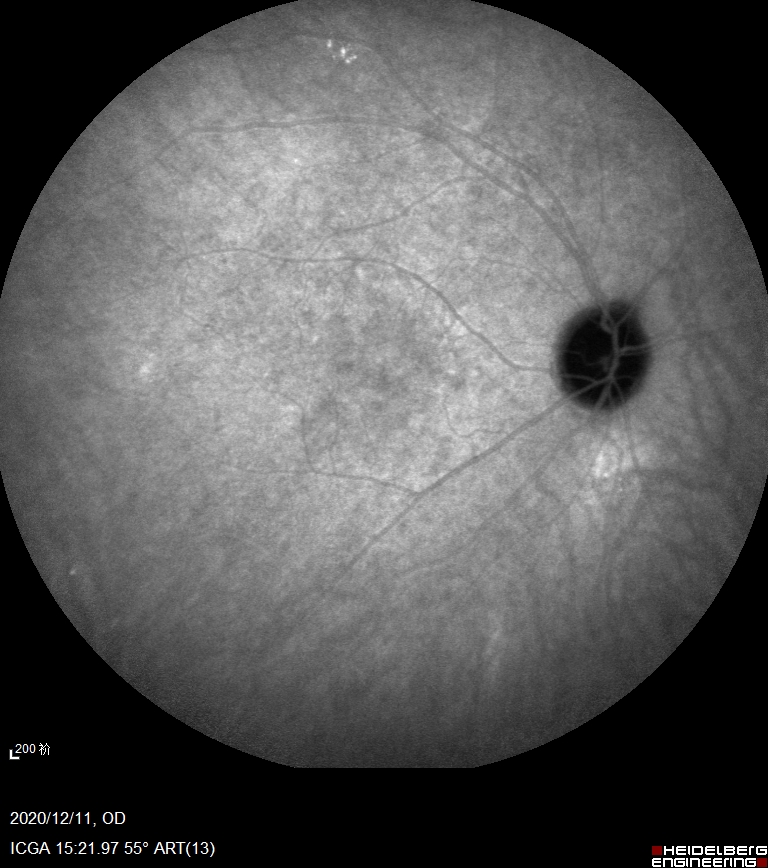

Supplement: Supplementary file 1 — Additional file 1: The raw data of this study. Table 1. The basic information of involved patients. [file 12886_2022_2598_MOESM1_ESM.zip › 1/ICGAΘâ¡Θçæσà░2/Θâ¡Θçæσà░2_016.jpg]

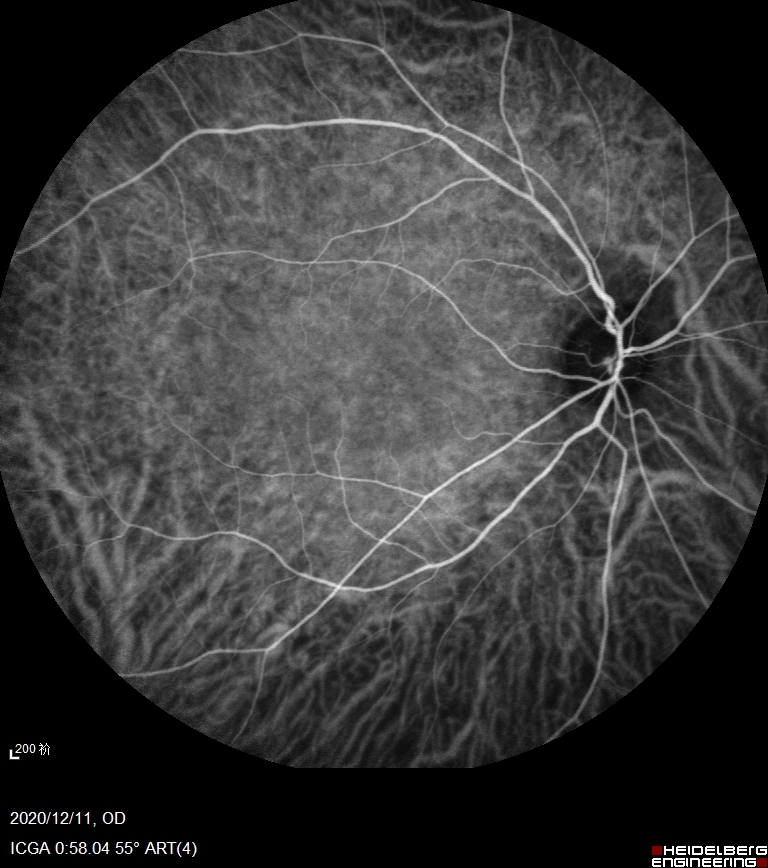

Supplement: Supplementary file 1 — Additional file 1: The raw data of this study. Table 1. The basic information of involved patients. [file 12886_2022_2598_MOESM1_ESM.zip › 1/ICGAΘâ¡Θçæσà░2/Θâ¡Θçæσà░2_002.jpg]

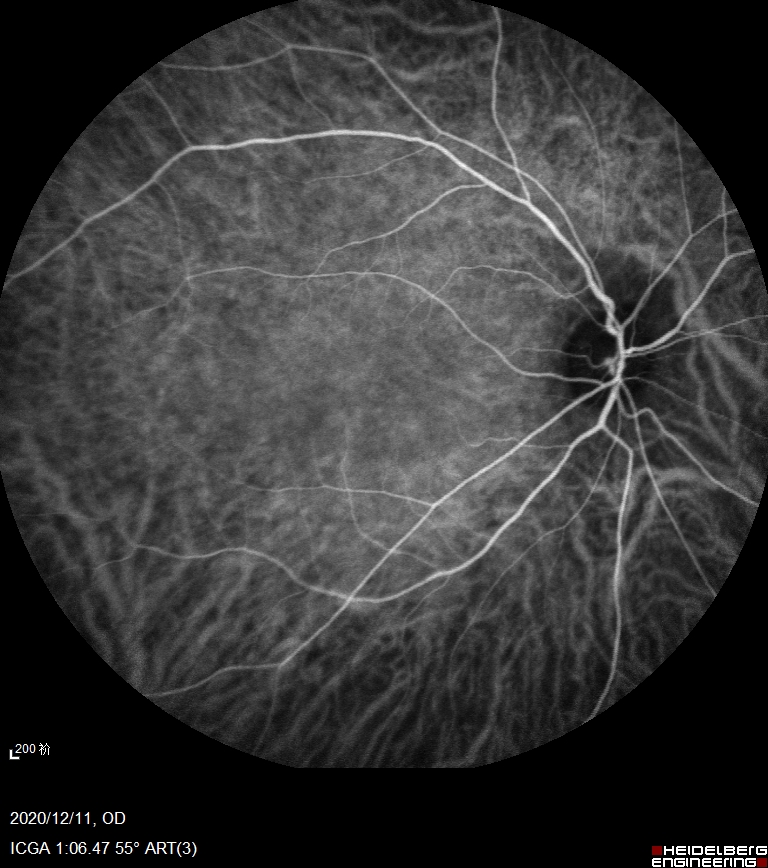

Supplement: Supplementary file 1 — Additional file 1: The raw data of this study. Table 1. The basic information of involved patients. [file 12886_2022_2598_MOESM1_ESM.zip › 1/ICGAΘâ¡Θçæσà░2/Θâ¡Θçæσà░2_003.jpg]
