# Supplementary figures and images for: Mucosal associated Lymphoid Tissue Lymphoma of the uvea: an analysis of 3 cases (part 1 of 2)
Source: BMC Ophthalmol. 2022 Sep 19;22:371. doi: 10.1186/s12886-022-02598-2 (PMC9484074; doi:10.1186/s12886-022-02598-2)

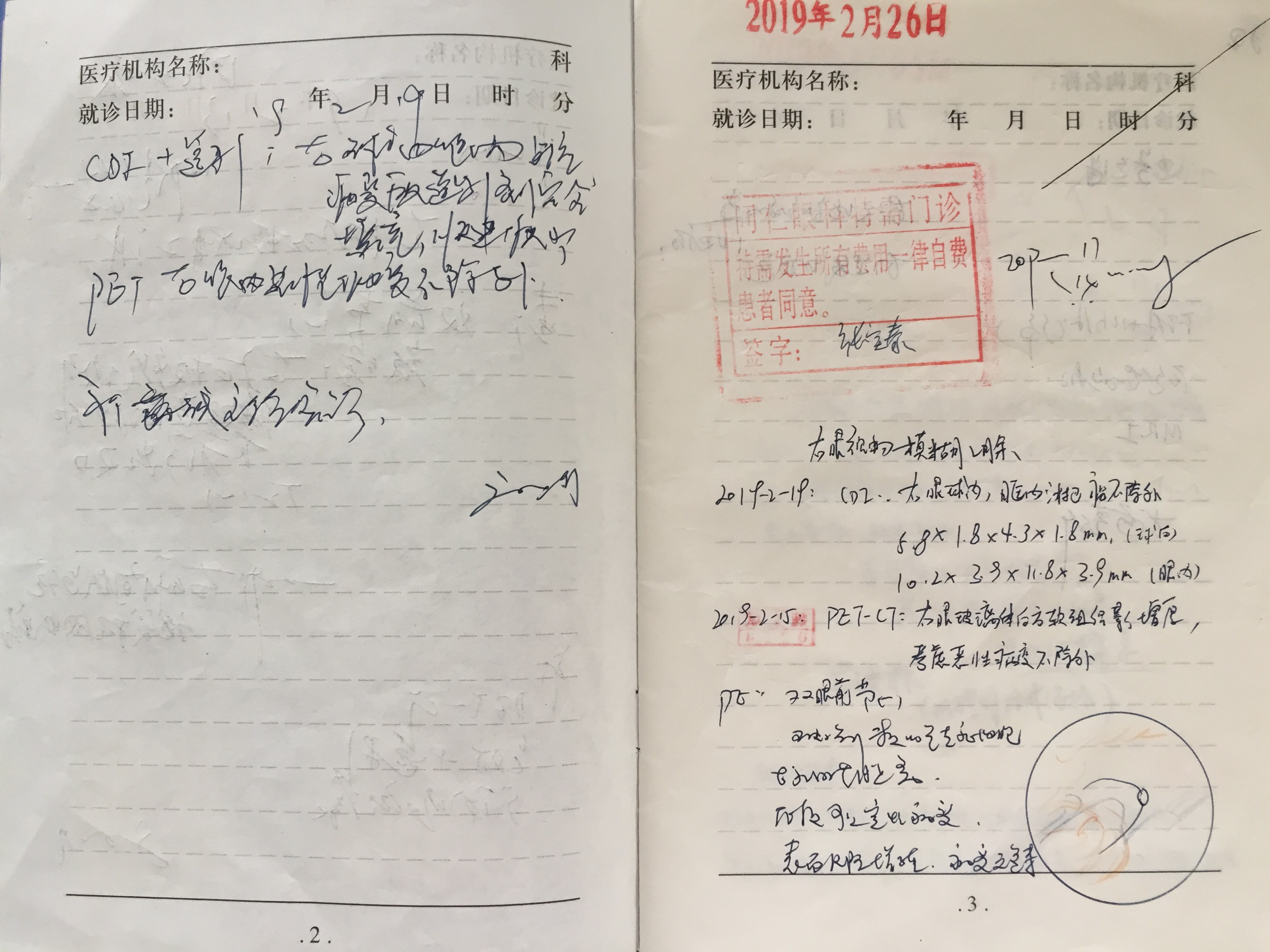

Supplement: Supplementary file 1 — Additional file 1: The raw data of this study. Table 1. The basic information of involved patients. [file 12886_2022_2598_MOESM1_ESM.zip › 2/IMG_8058.JPG]

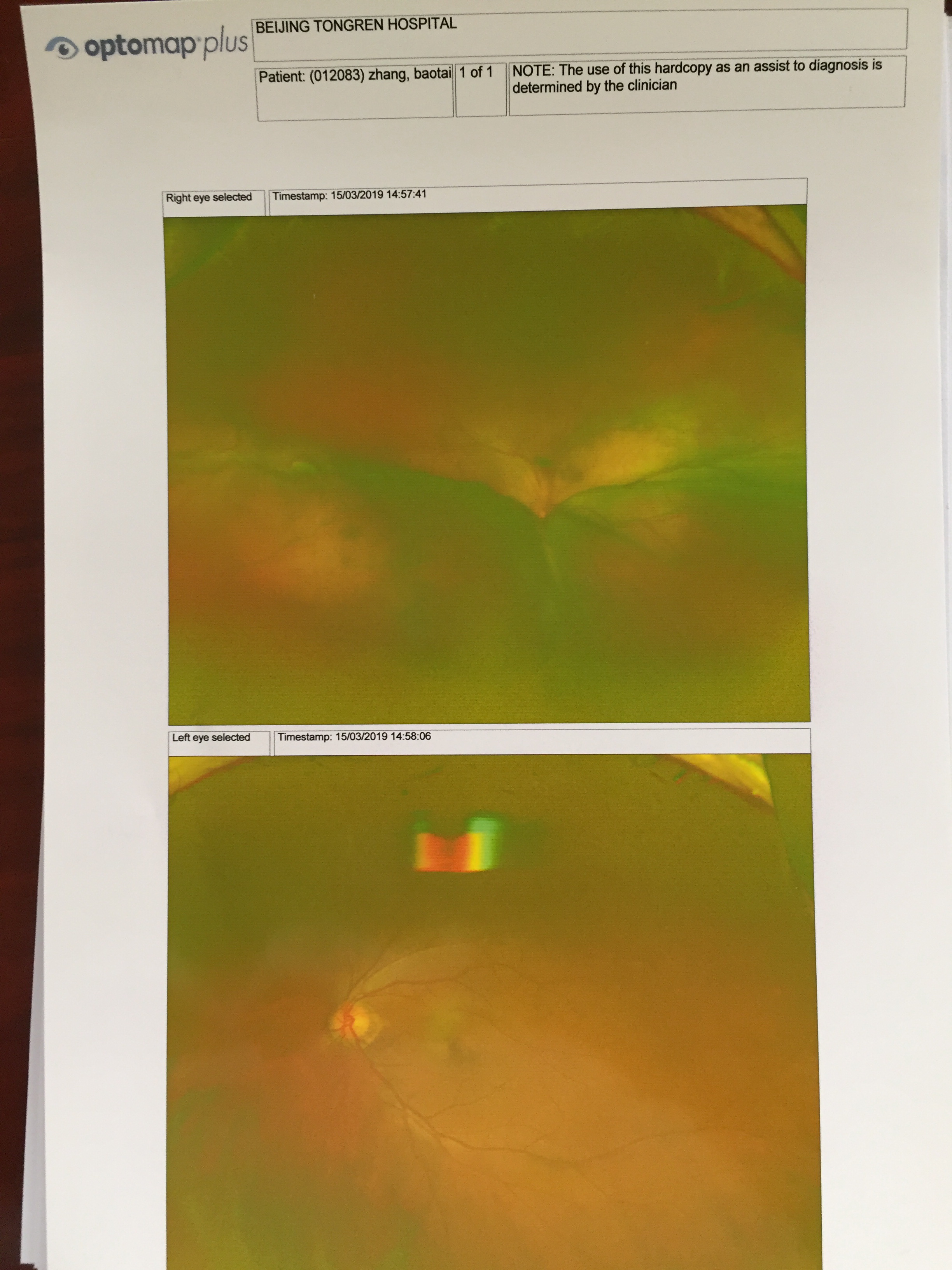

Supplement: Supplementary file 1 — Additional file 1: The raw data of this study. Table 1. The basic information of involved patients. [file 12886_2022_2598_MOESM1_ESM.zip › 2/IMG_8064.JPG]

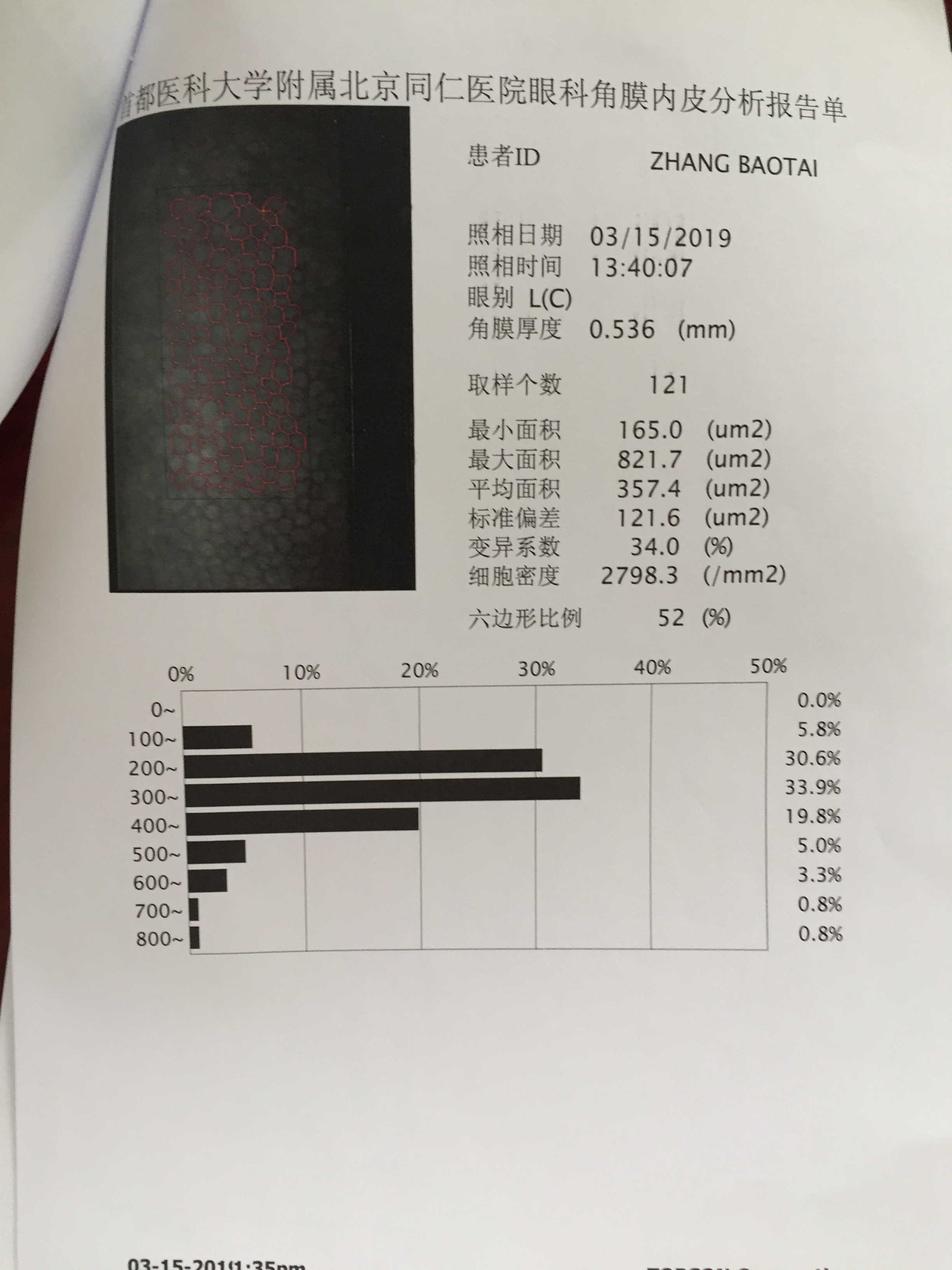

Supplement: Supplementary file 1 — Additional file 1: The raw data of this study. Table 1. The basic information of involved patients. [file 12886_2022_2598_MOESM1_ESM.zip › 2/IMG_8070.JPG]

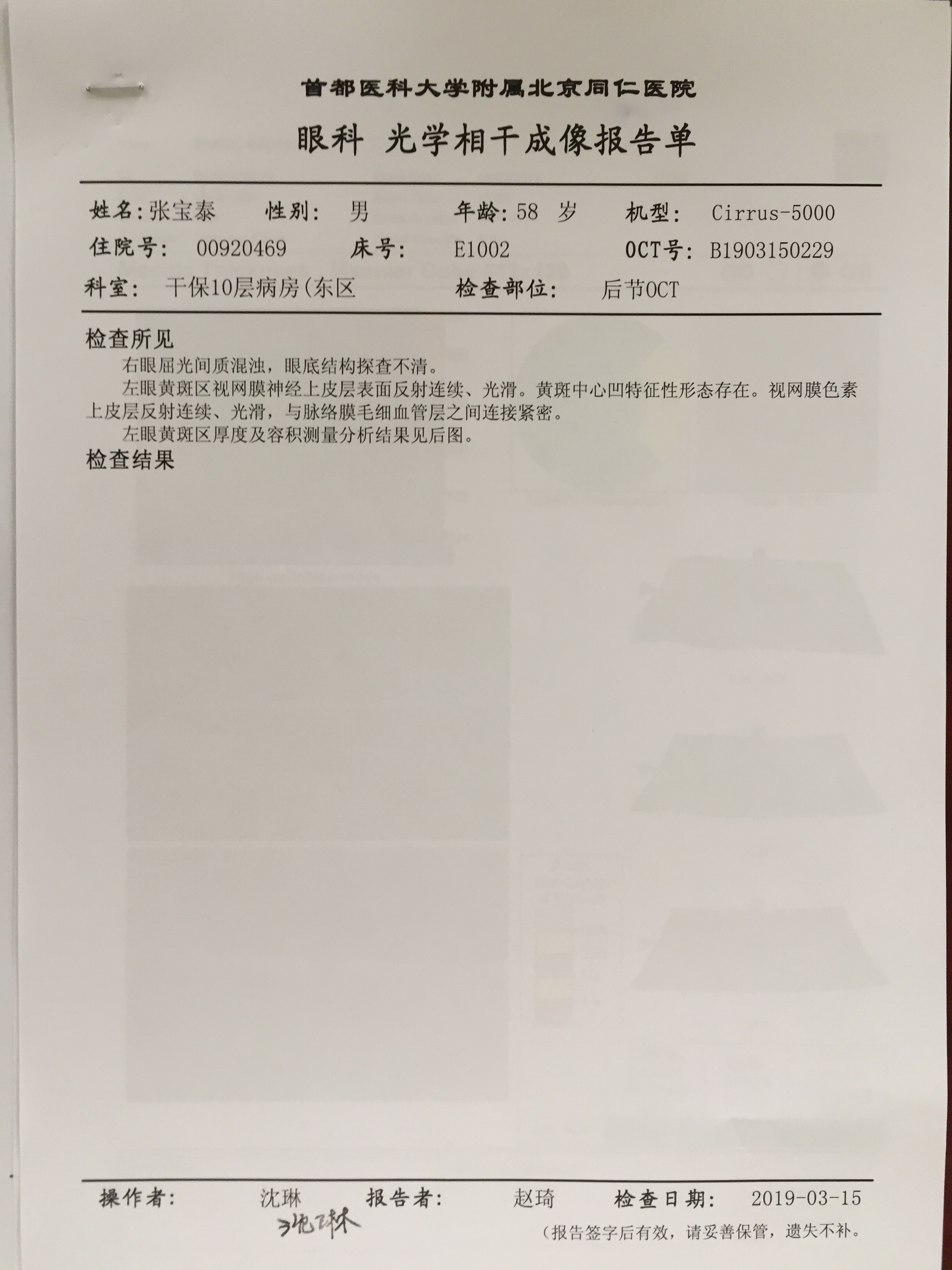

Supplement: Supplementary file 1 — Additional file 1: The raw data of this study. Table 1. The basic information of involved patients. [file 12886_2022_2598_MOESM1_ESM.zip › 2/IMG_8071.JPG]

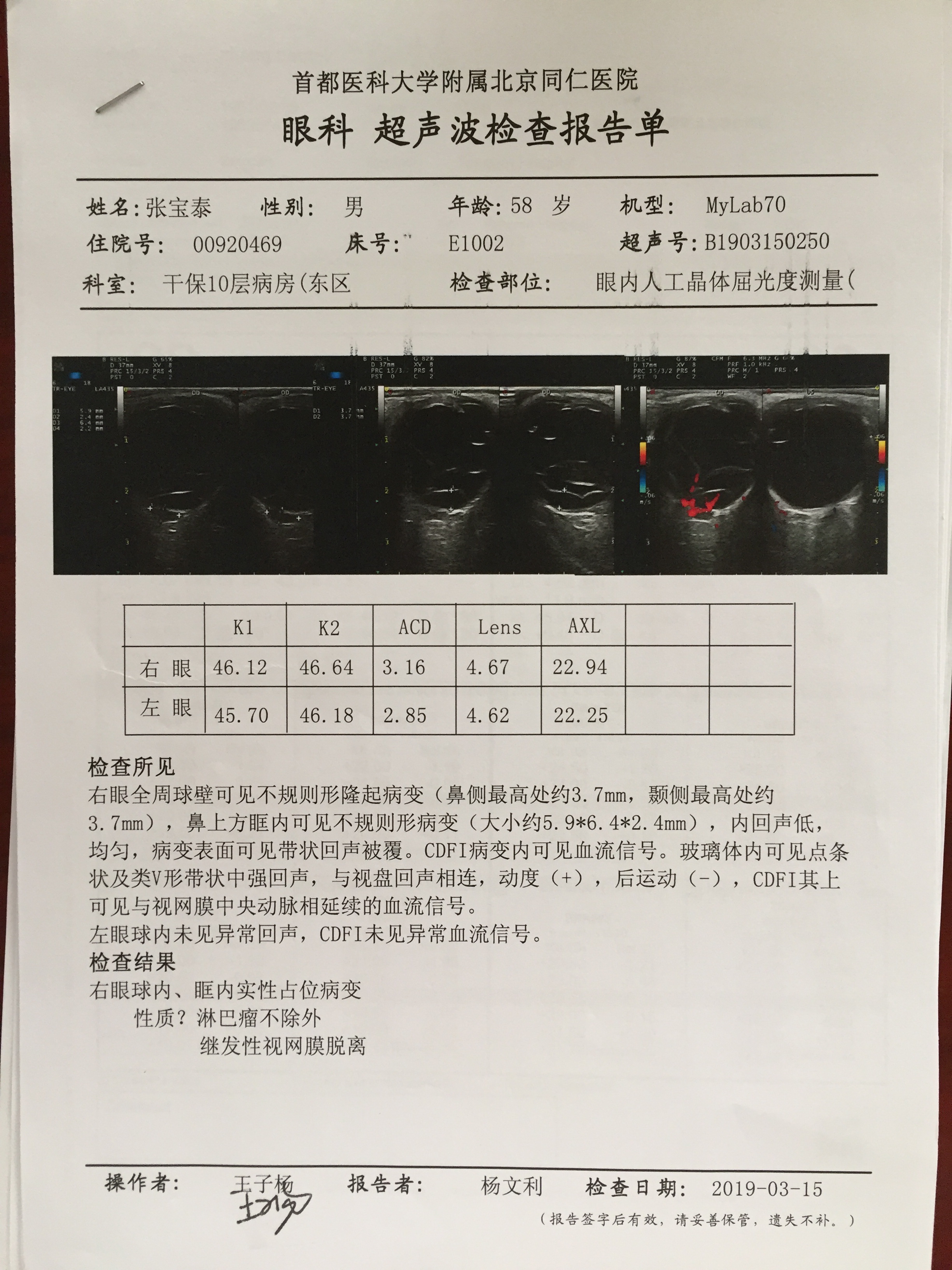

Supplement: Supplementary file 1 — Additional file 1: The raw data of this study. Table 1. The basic information of involved patients. [file 12886_2022_2598_MOESM1_ESM.zip › 2/IMG_8065.JPG]

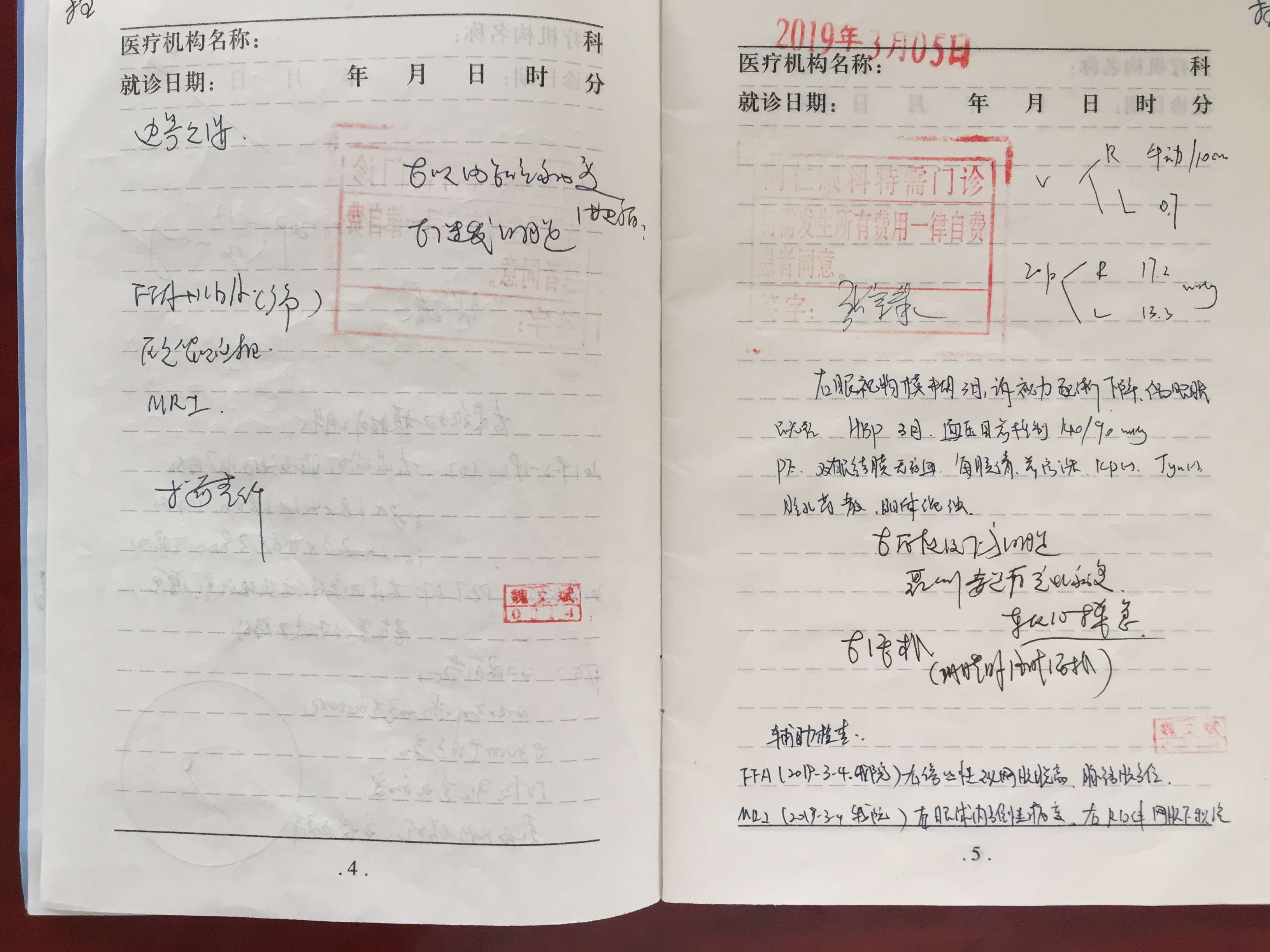

Supplement: Supplementary file 1 — Additional file 1: The raw data of this study. Table 1. The basic information of involved patients. [file 12886_2022_2598_MOESM1_ESM.zip › 2/IMG_8059.JPG]

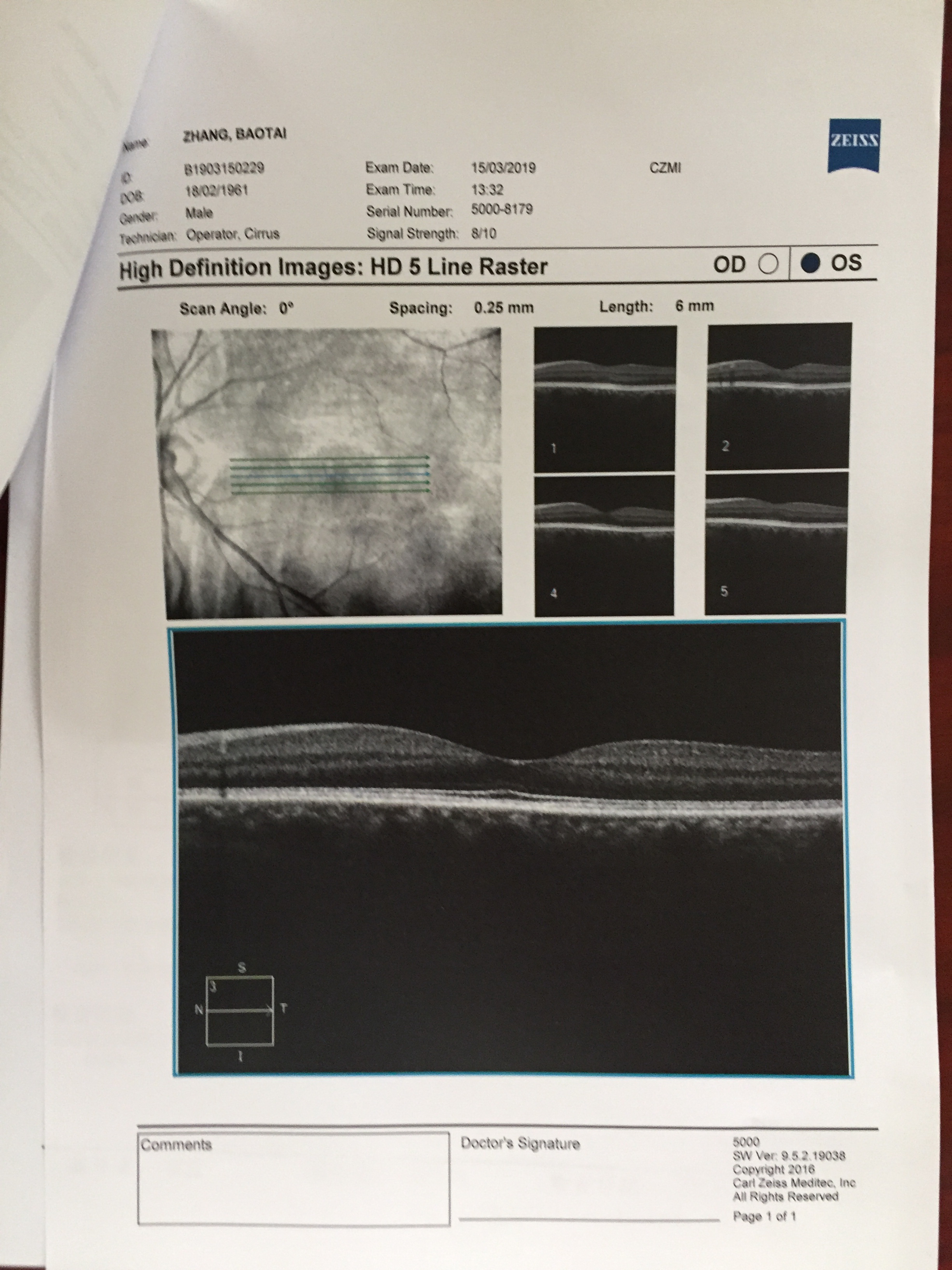

Supplement: Supplementary file 1 — Additional file 1: The raw data of this study. Table 1. The basic information of involved patients. [file 12886_2022_2598_MOESM1_ESM.zip › 2/IMG_8073.JPG]

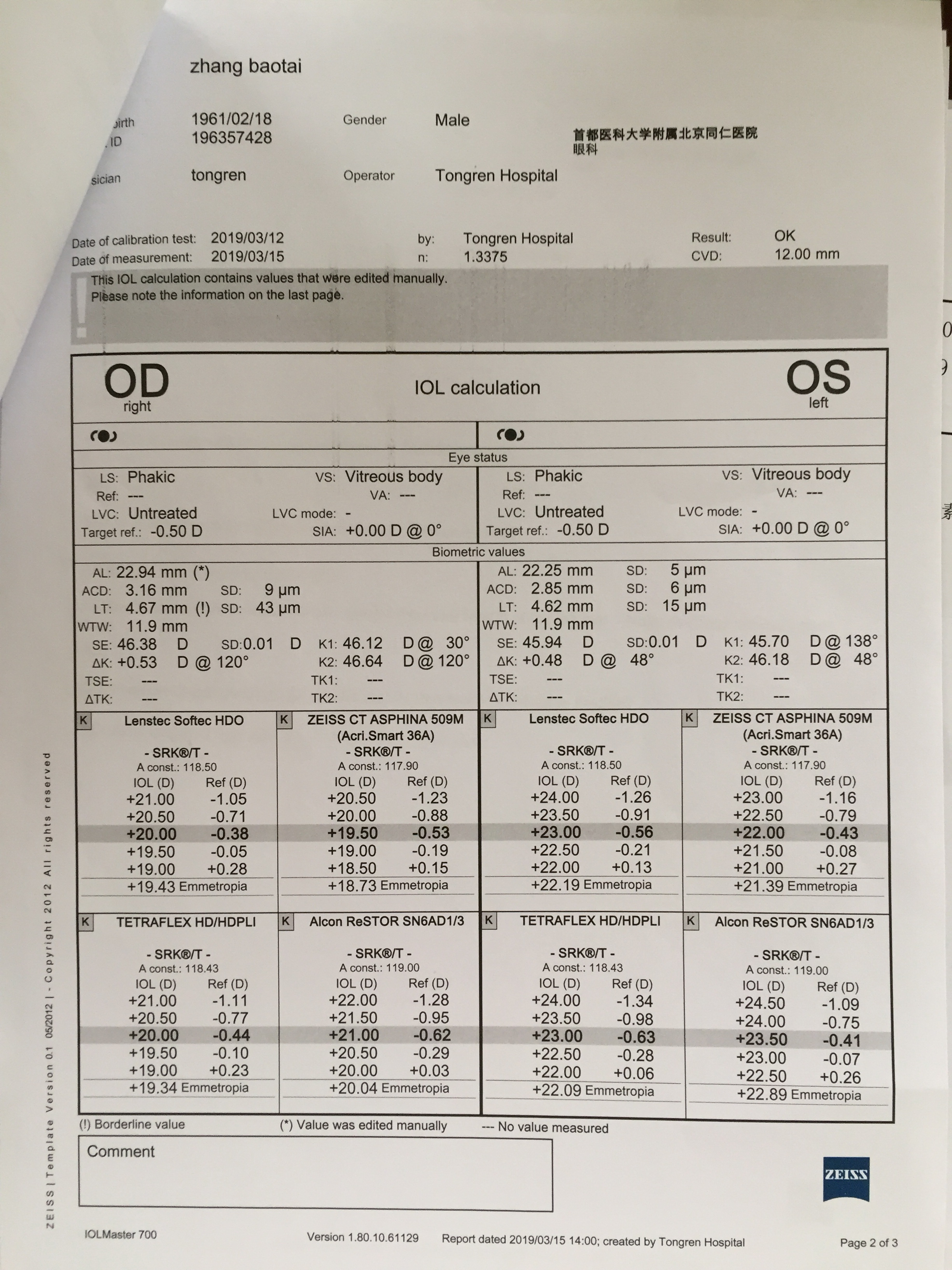

Supplement: Supplementary file 1 — Additional file 1: The raw data of this study. Table 1. The basic information of involved patients. [file 12886_2022_2598_MOESM1_ESM.zip › 2/IMG_8067.JPG]

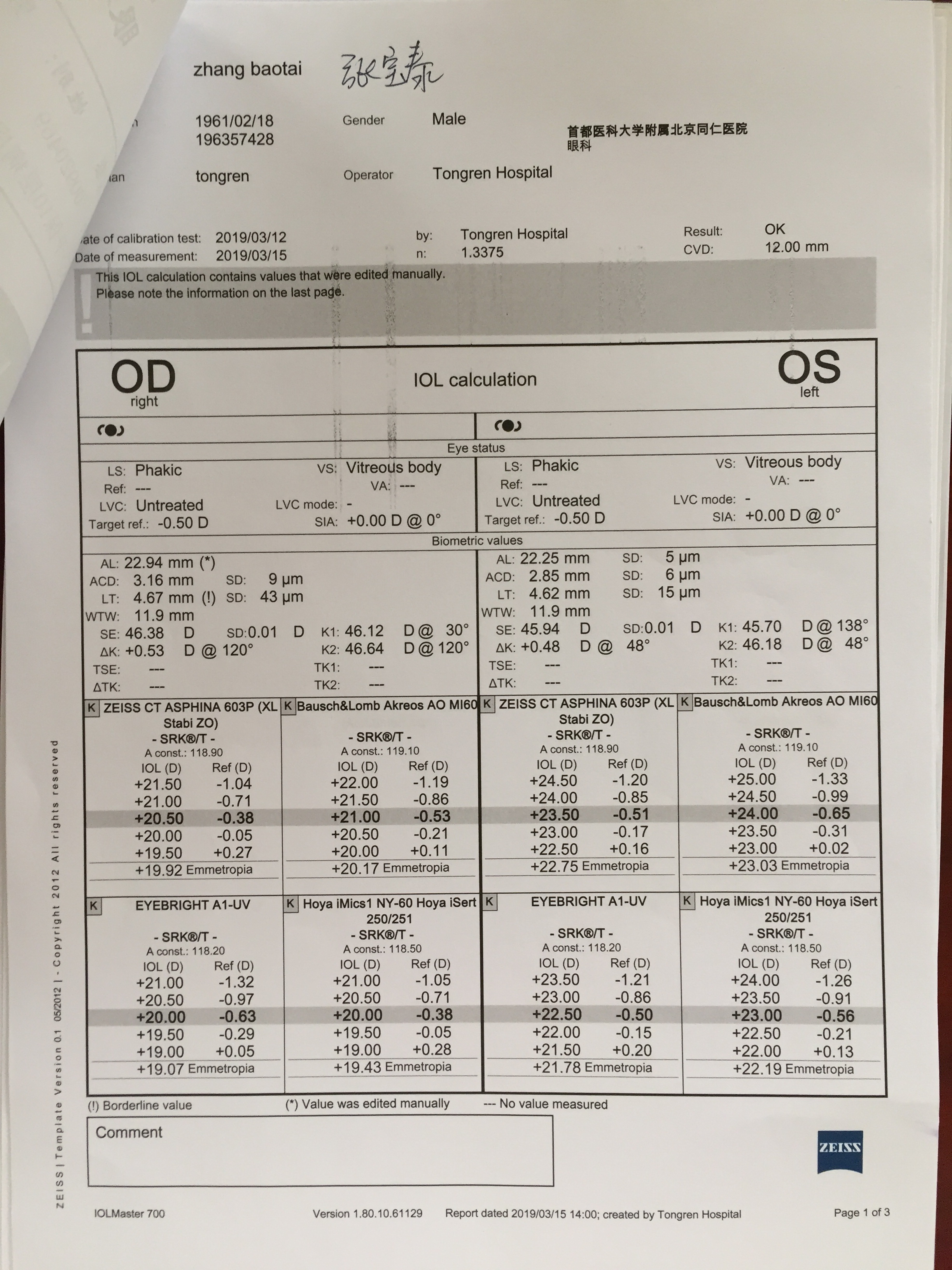

Supplement: Supplementary file 1 — Additional file 1: The raw data of this study. Table 1. The basic information of involved patients. [file 12886_2022_2598_MOESM1_ESM.zip › 2/IMG_8066.JPG]

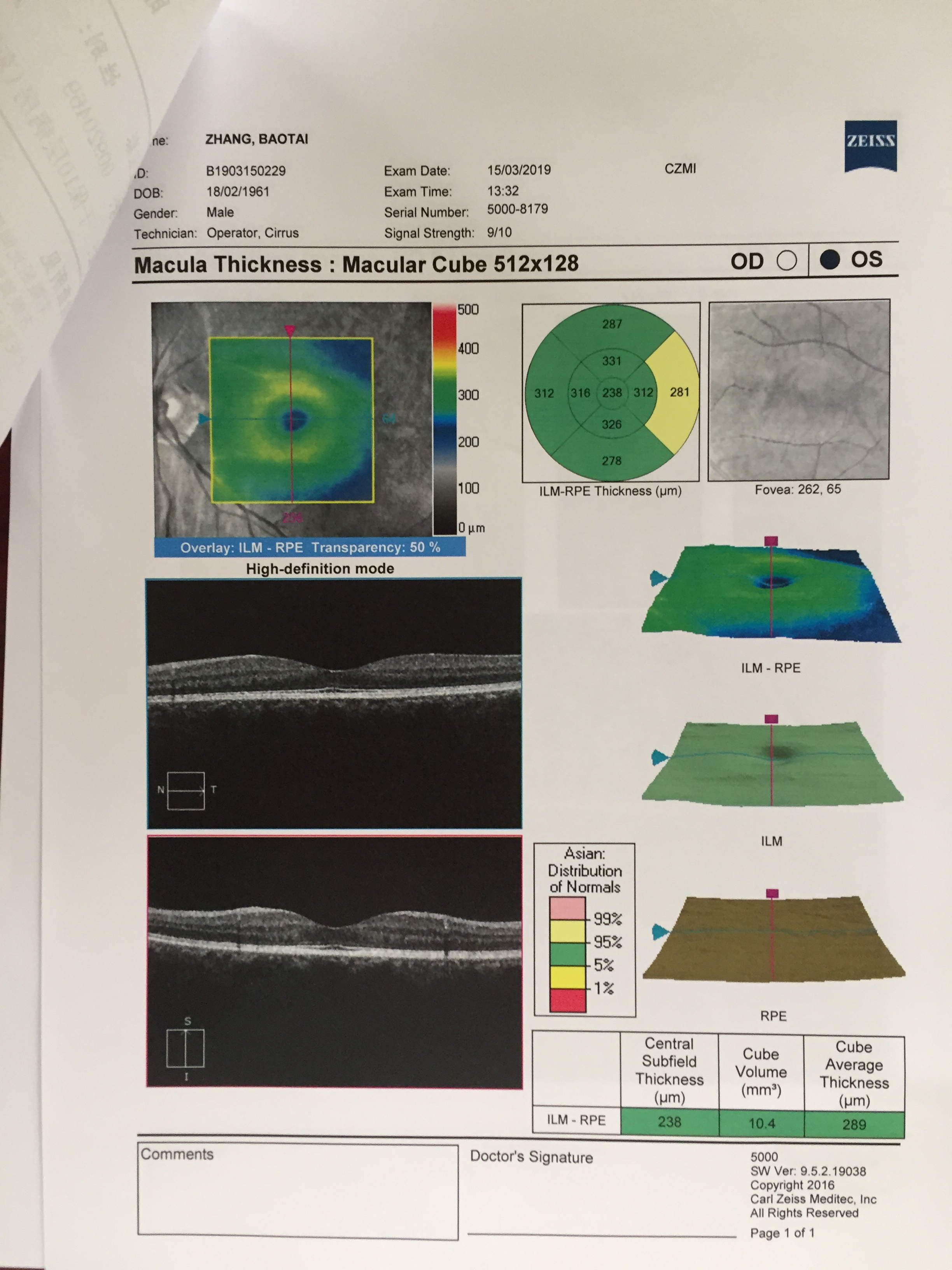

Supplement: Supplementary file 1 — Additional file 1: The raw data of this study. Table 1. The basic information of involved patients. [file 12886_2022_2598_MOESM1_ESM.zip › 2/IMG_8072.JPG]

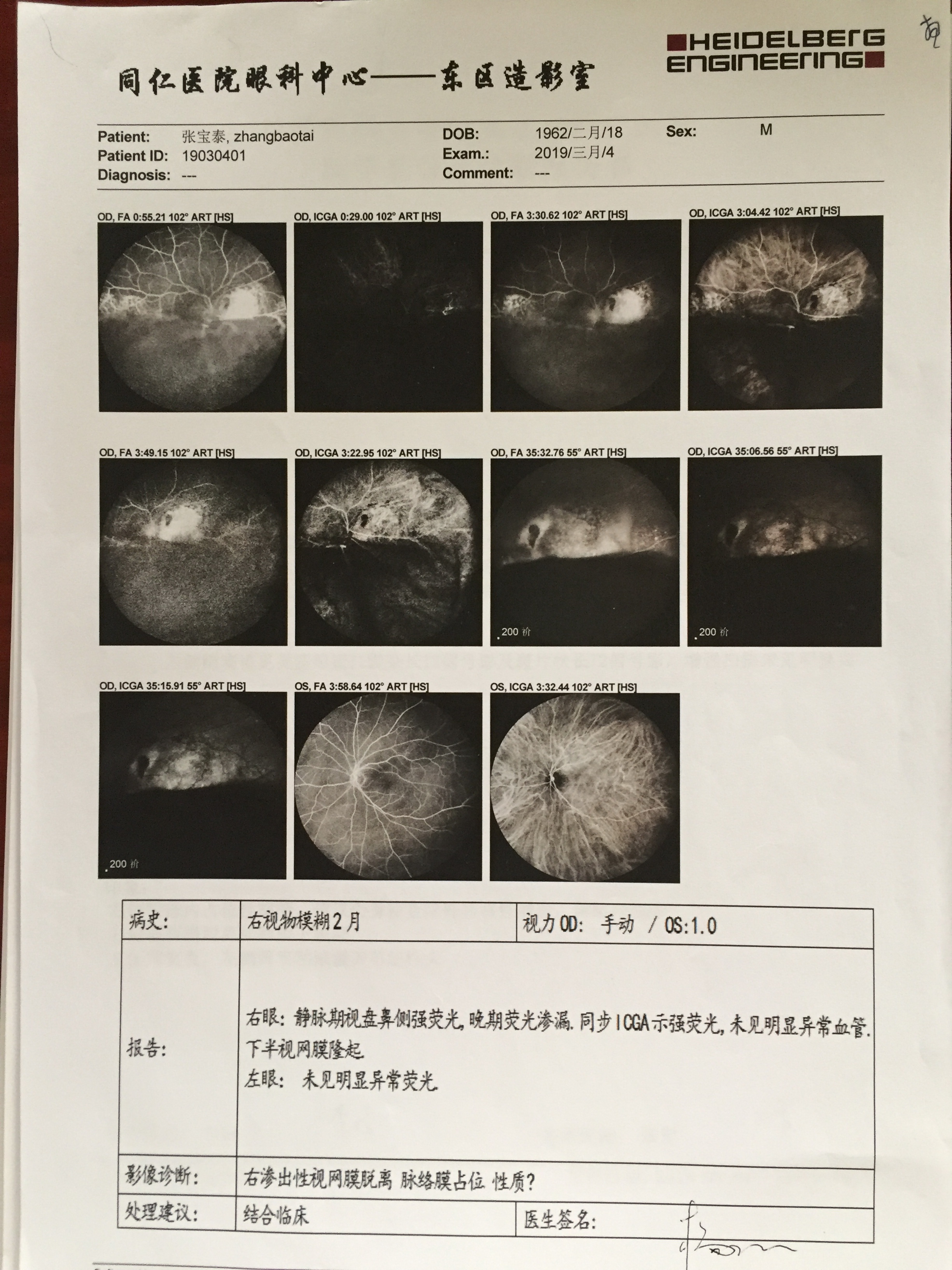

Supplement: Supplementary file 1 — Additional file 1: The raw data of this study. Table 1. The basic information of involved patients. [file 12886_2022_2598_MOESM1_ESM.zip › 2/IMG_8089.JPG]

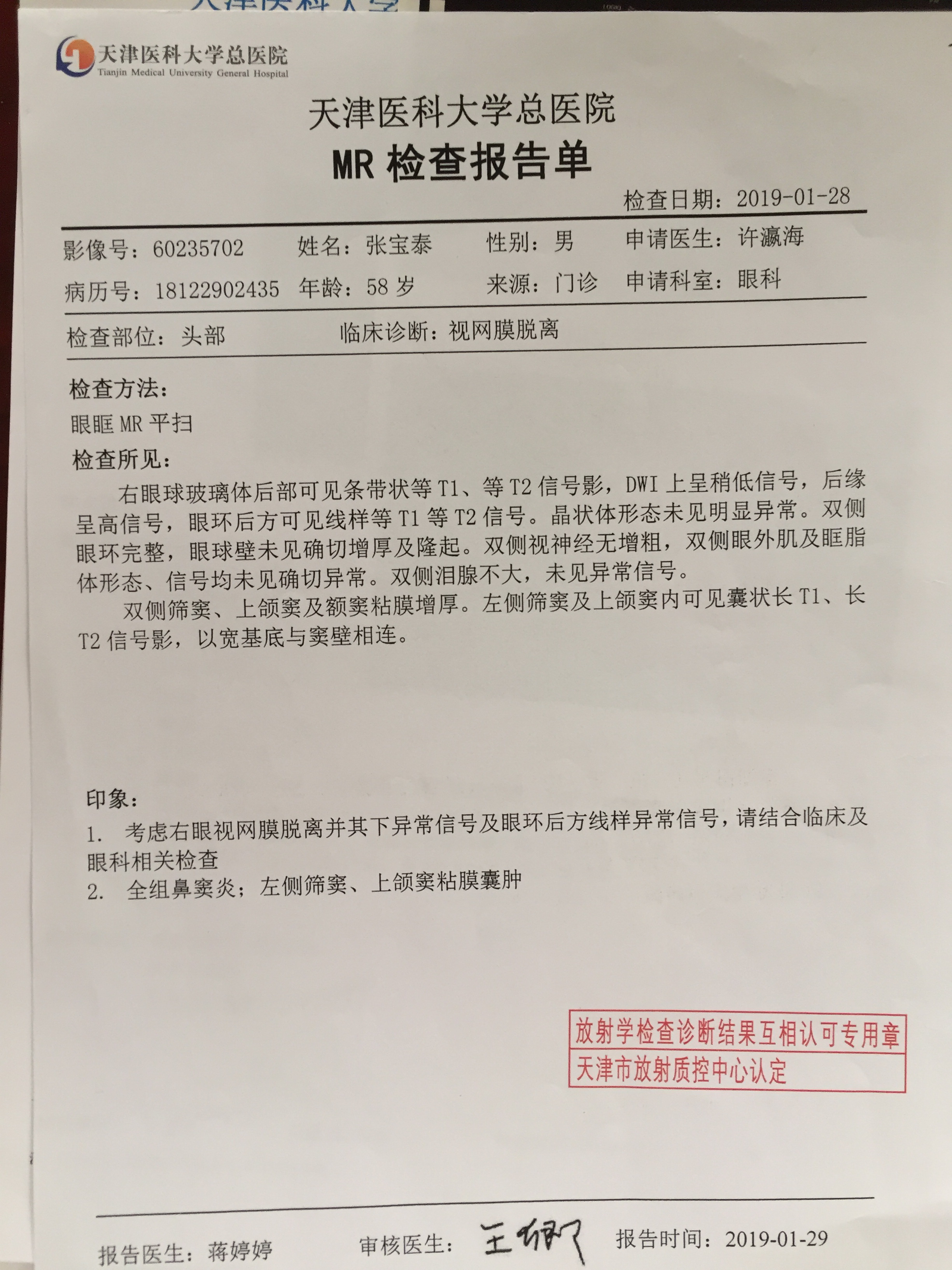

Supplement: Supplementary file 1 — Additional file 1: The raw data of this study. Table 1. The basic information of involved patients. [file 12886_2022_2598_MOESM1_ESM.zip › 2/IMG_8076.JPG]

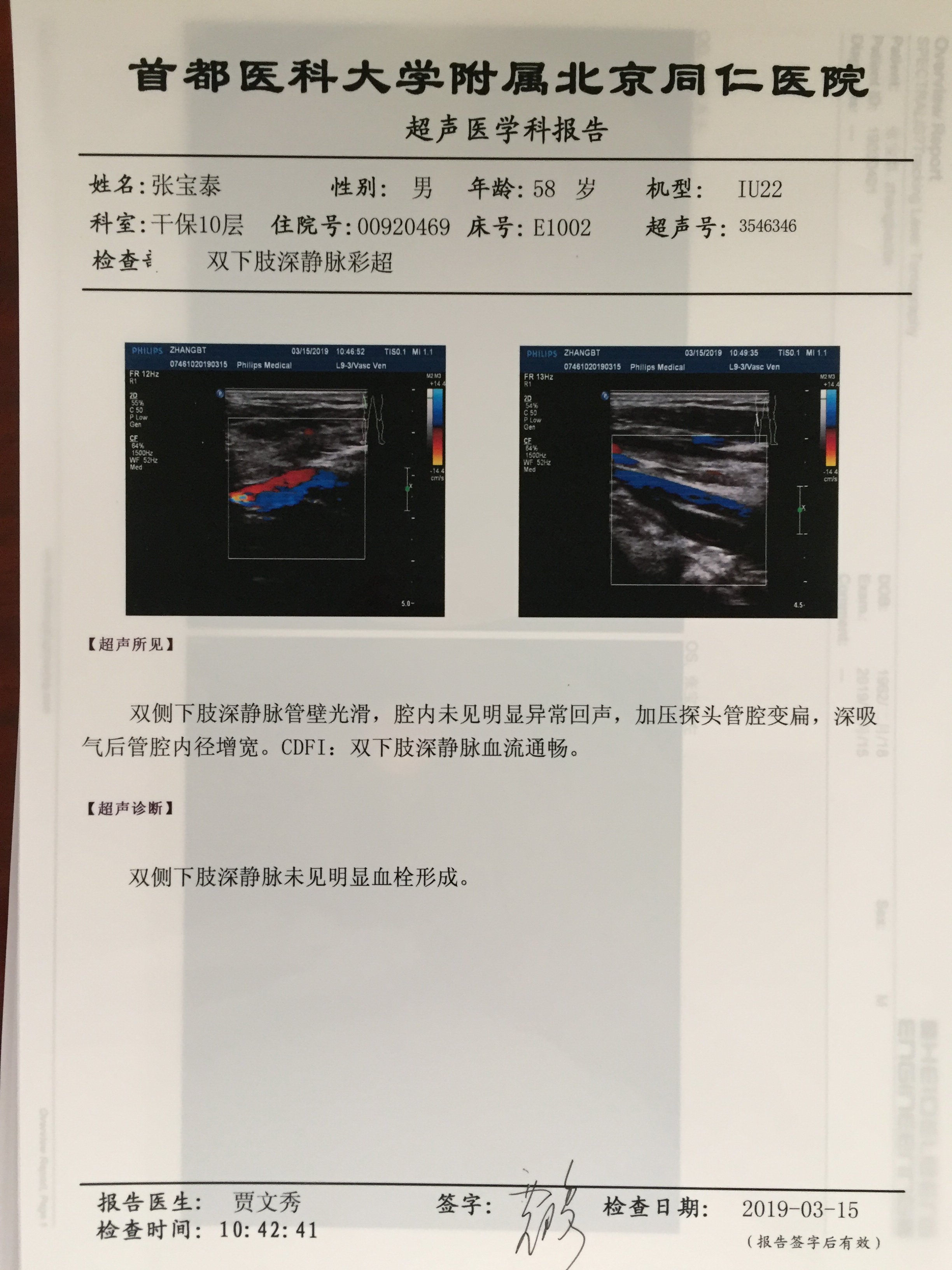

Supplement: Supplementary file 1 — Additional file 1: The raw data of this study. Table 1. The basic information of involved patients. [file 12886_2022_2598_MOESM1_ESM.zip › 2/IMG_8062.JPG]

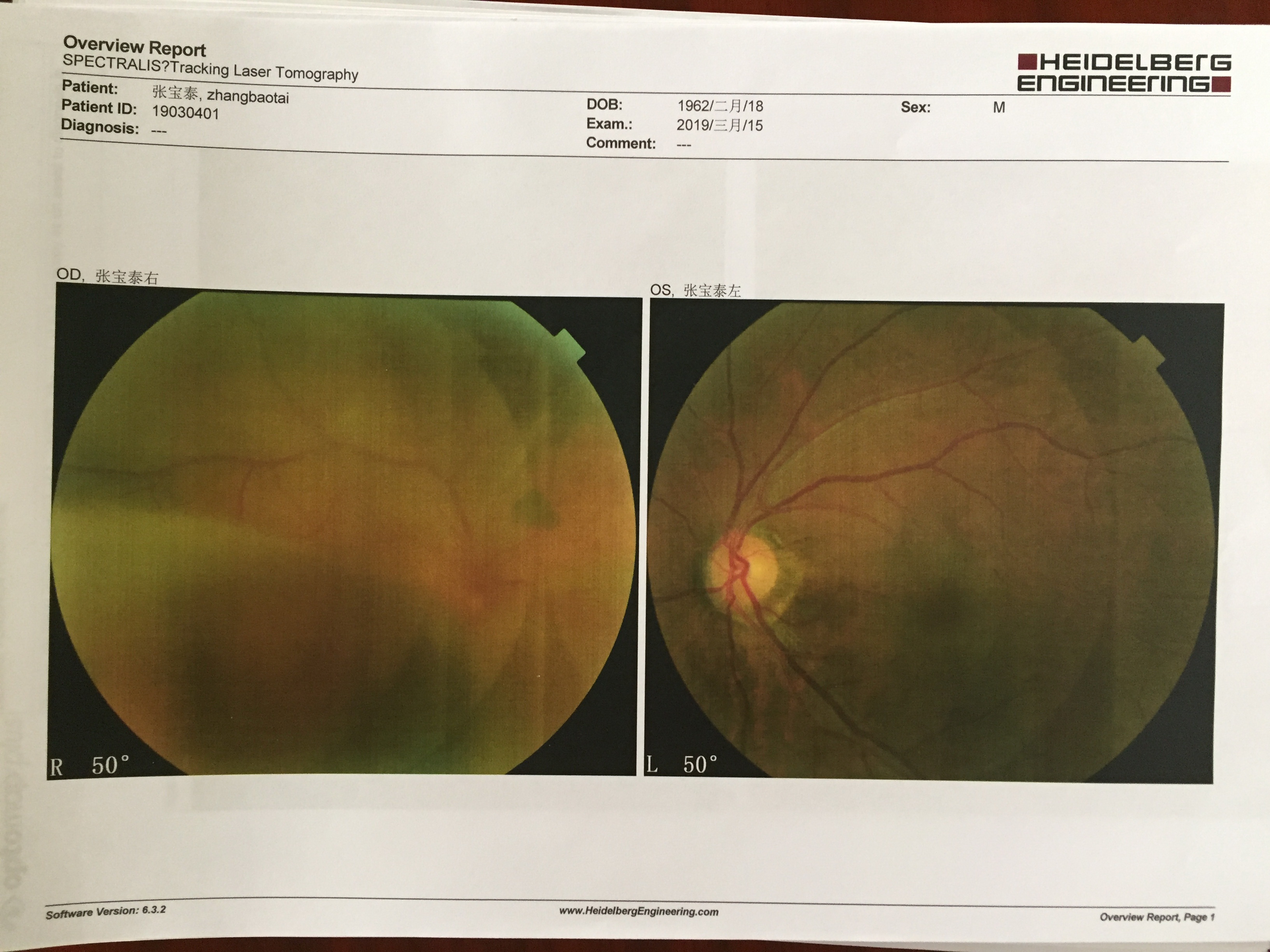

Supplement: Supplementary file 1 — Additional file 1: The raw data of this study. Table 1. The basic information of involved patients. [file 12886_2022_2598_MOESM1_ESM.zip › 2/IMG_8063.JPG]

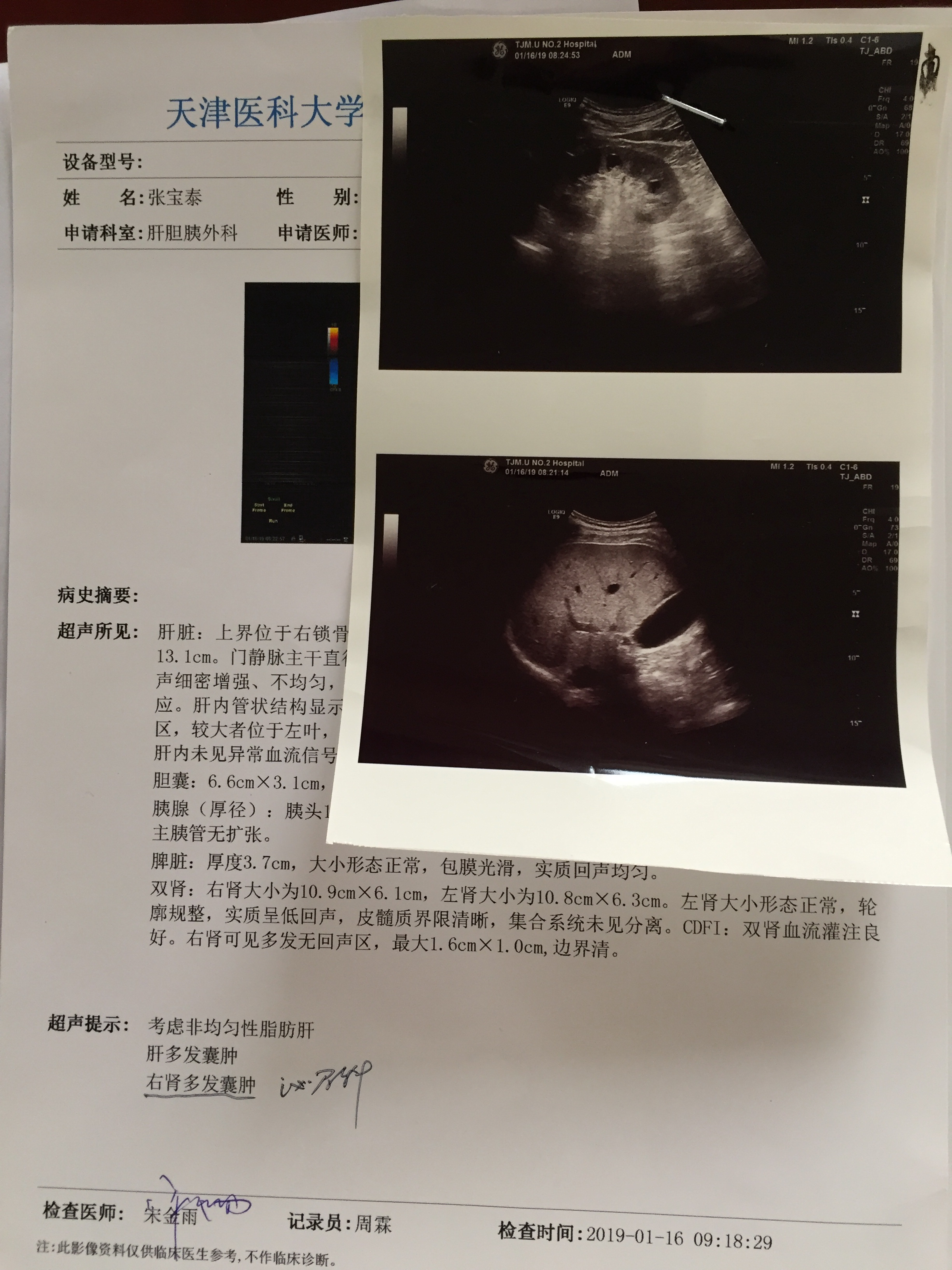

Supplement: Supplementary file 1 — Additional file 1: The raw data of this study. Table 1. The basic information of involved patients. [file 12886_2022_2598_MOESM1_ESM.zip › 2/IMG_8077.JPG]

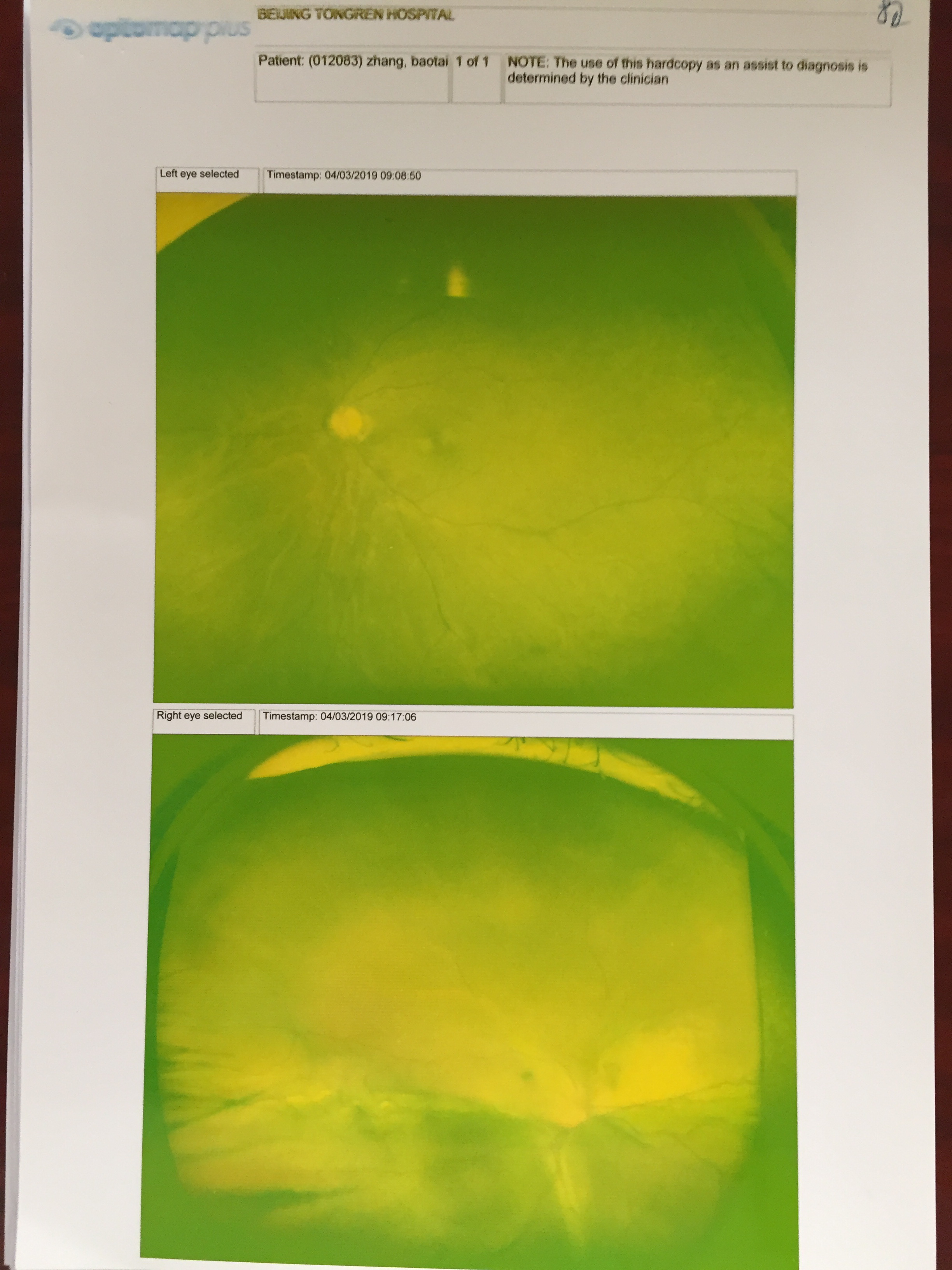

Supplement: Supplementary file 1 — Additional file 1: The raw data of this study. Table 1. The basic information of involved patients. [file 12886_2022_2598_MOESM1_ESM.zip › 2/IMG_8088.JPG]

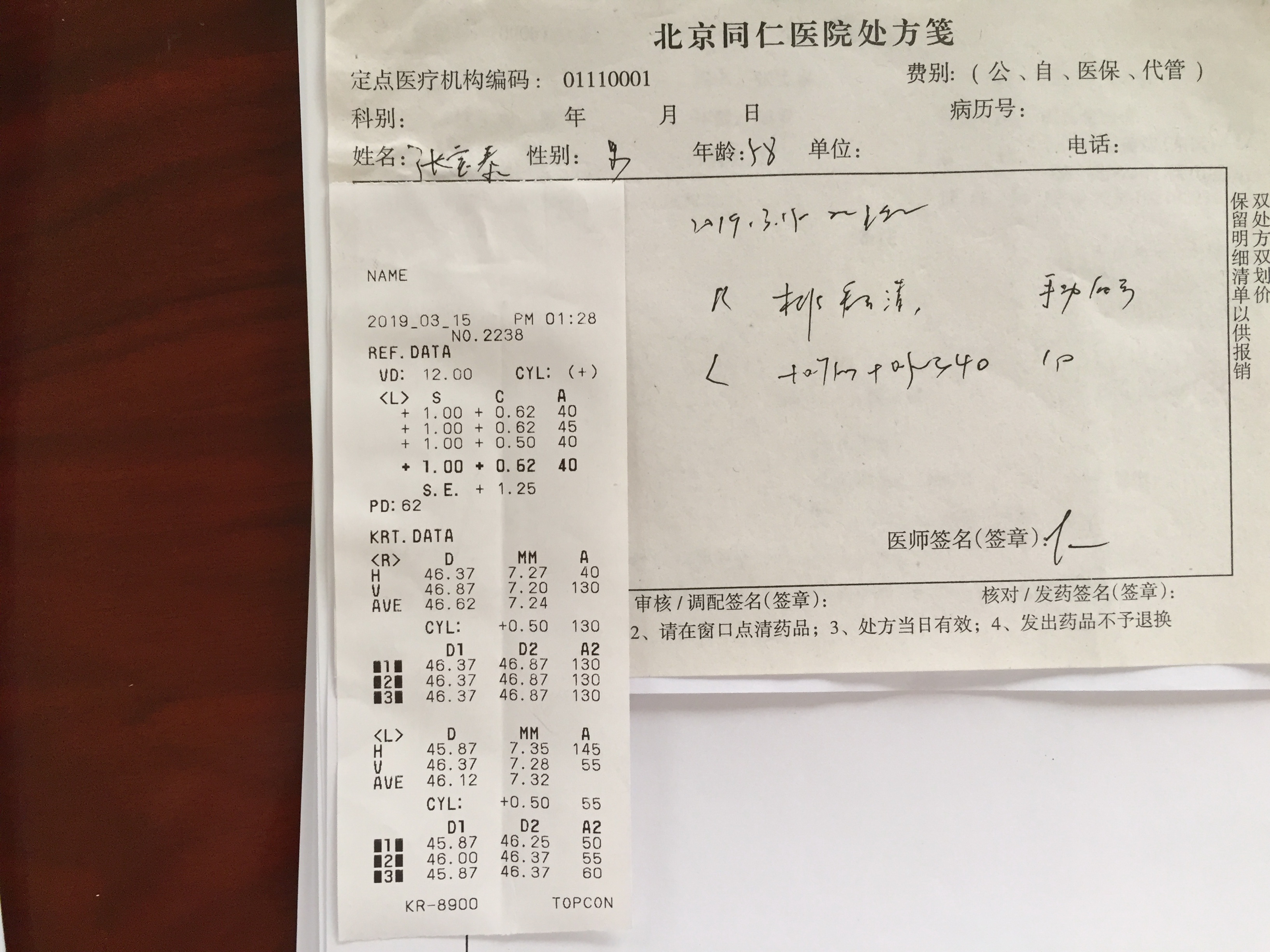

Supplement: Supplementary file 1 — Additional file 1: The raw data of this study. Table 1. The basic information of involved patients. [file 12886_2022_2598_MOESM1_ESM.zip › 2/IMG_8061.JPG]

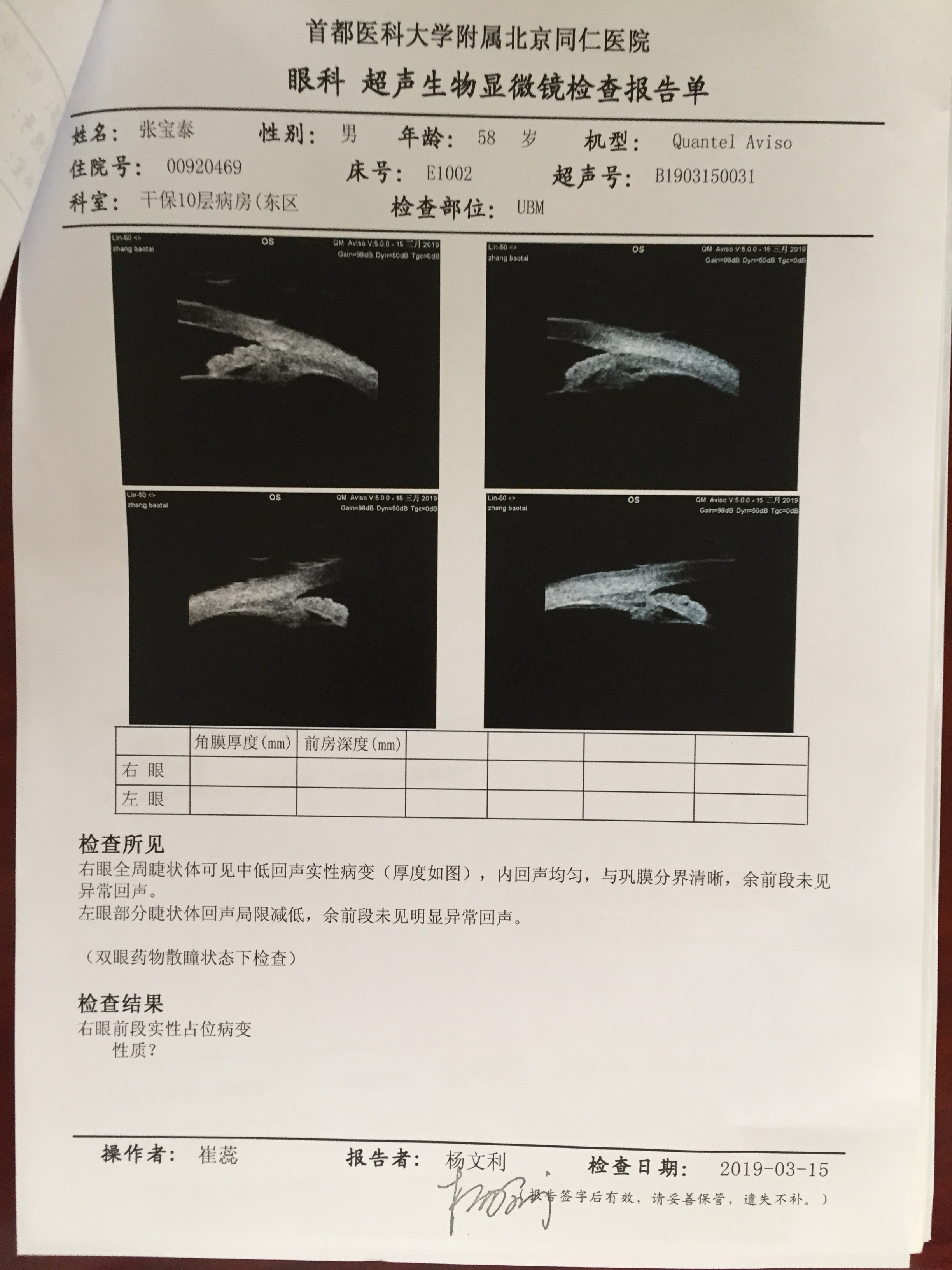

Supplement: Supplementary file 1 — Additional file 1: The raw data of this study. Table 1. The basic information of involved patients. [file 12886_2022_2598_MOESM1_ESM.zip › 2/IMG_8075.JPG]

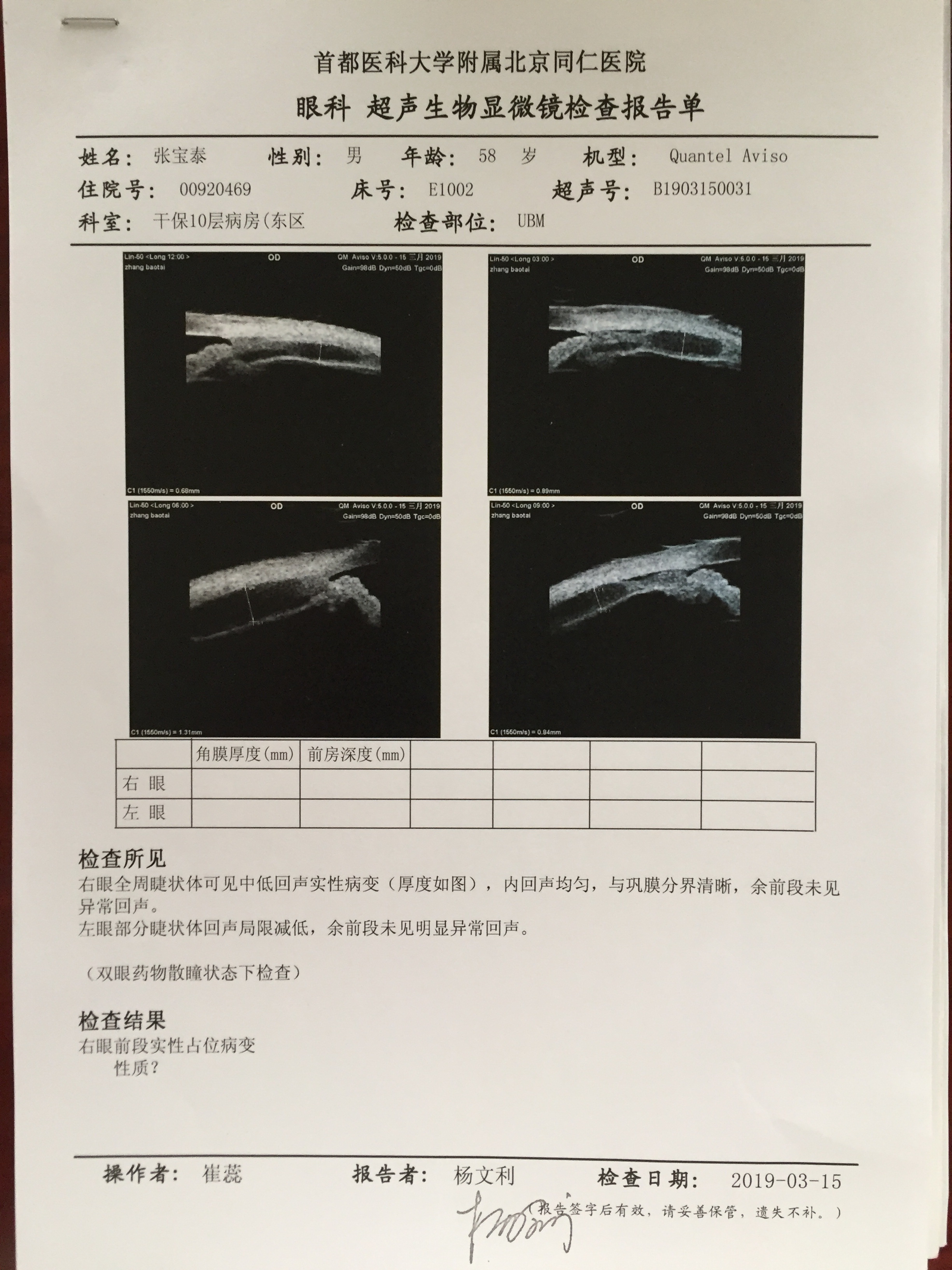

Supplement: Supplementary file 1 — Additional file 1: The raw data of this study. Table 1. The basic information of involved patients. [file 12886_2022_2598_MOESM1_ESM.zip › 2/IMG_8074.JPG]

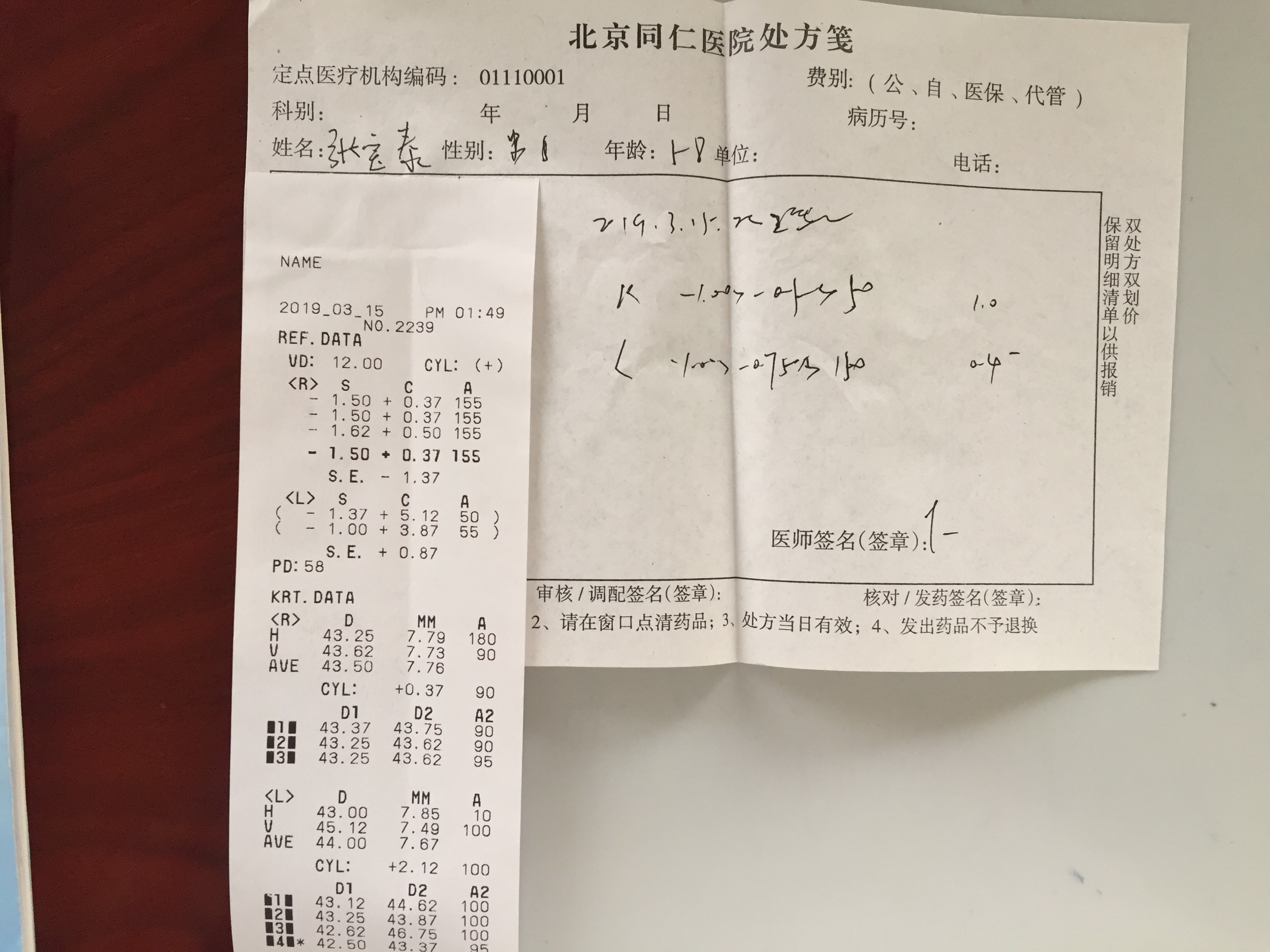

Supplement: Supplementary file 1 — Additional file 1: The raw data of this study. Table 1. The basic information of involved patients. [file 12886_2022_2598_MOESM1_ESM.zip › 2/IMG_8060.JPG]

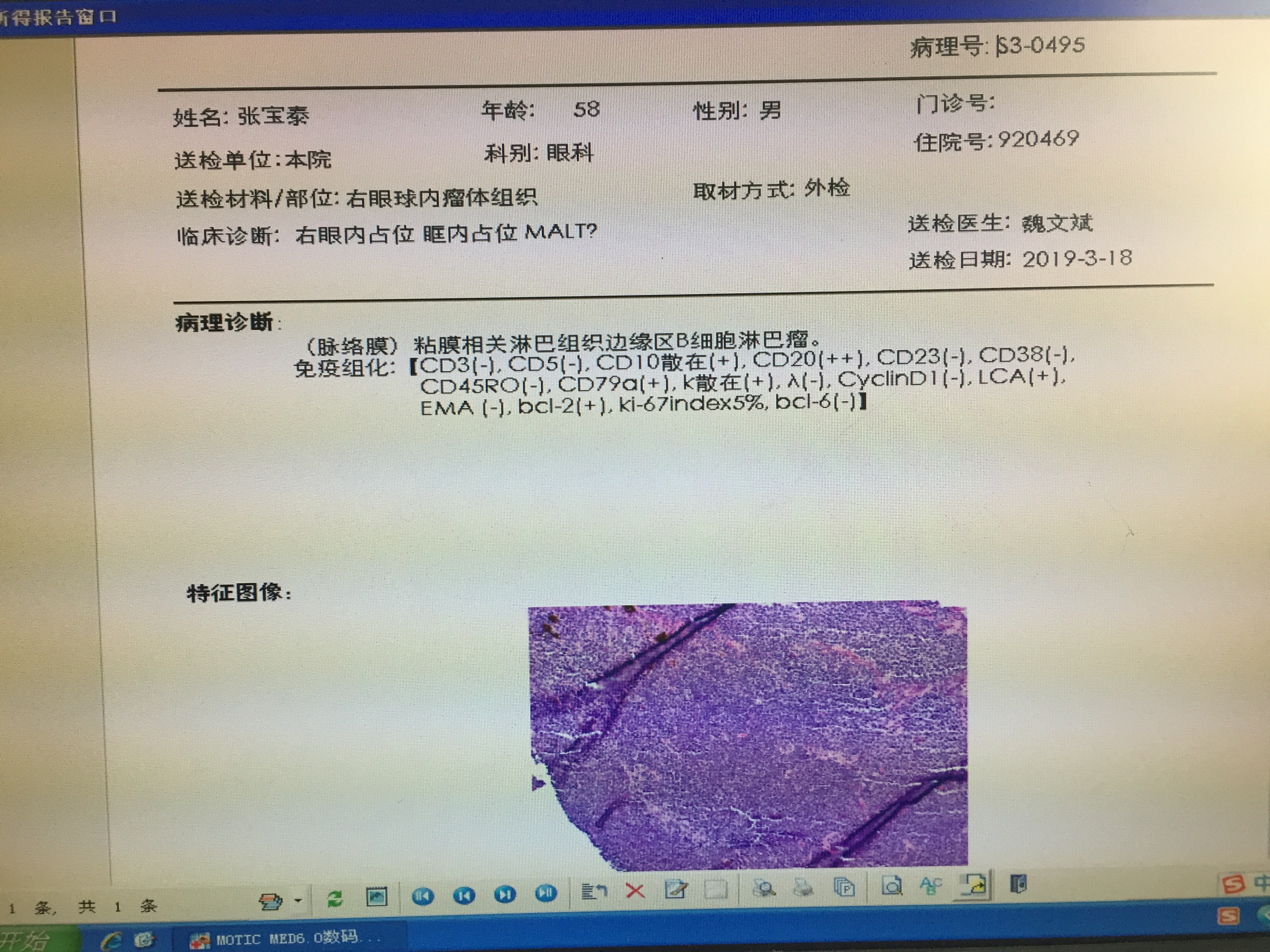

Supplement: Supplementary file 1 — Additional file 1: The raw data of this study. Table 1. The basic information of involved patients. [file 12886_2022_2598_MOESM1_ESM.zip › 2/IMG_9116.JPG]

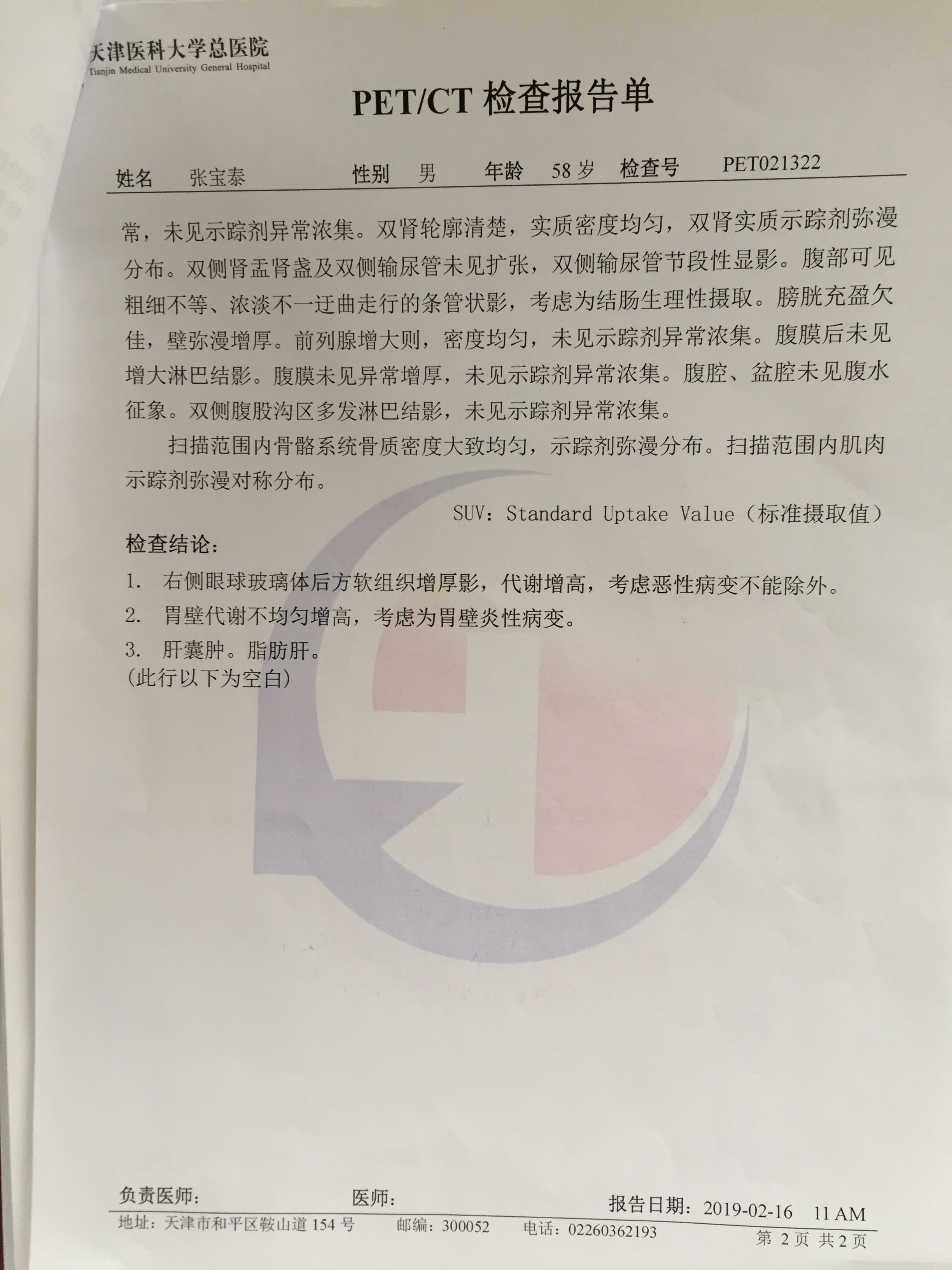

Supplement: Supplementary file 1 — Additional file 1: The raw data of this study. Table 1. The basic information of involved patients. [file 12886_2022_2598_MOESM1_ESM.zip › 2/IMG_8086.JPG]

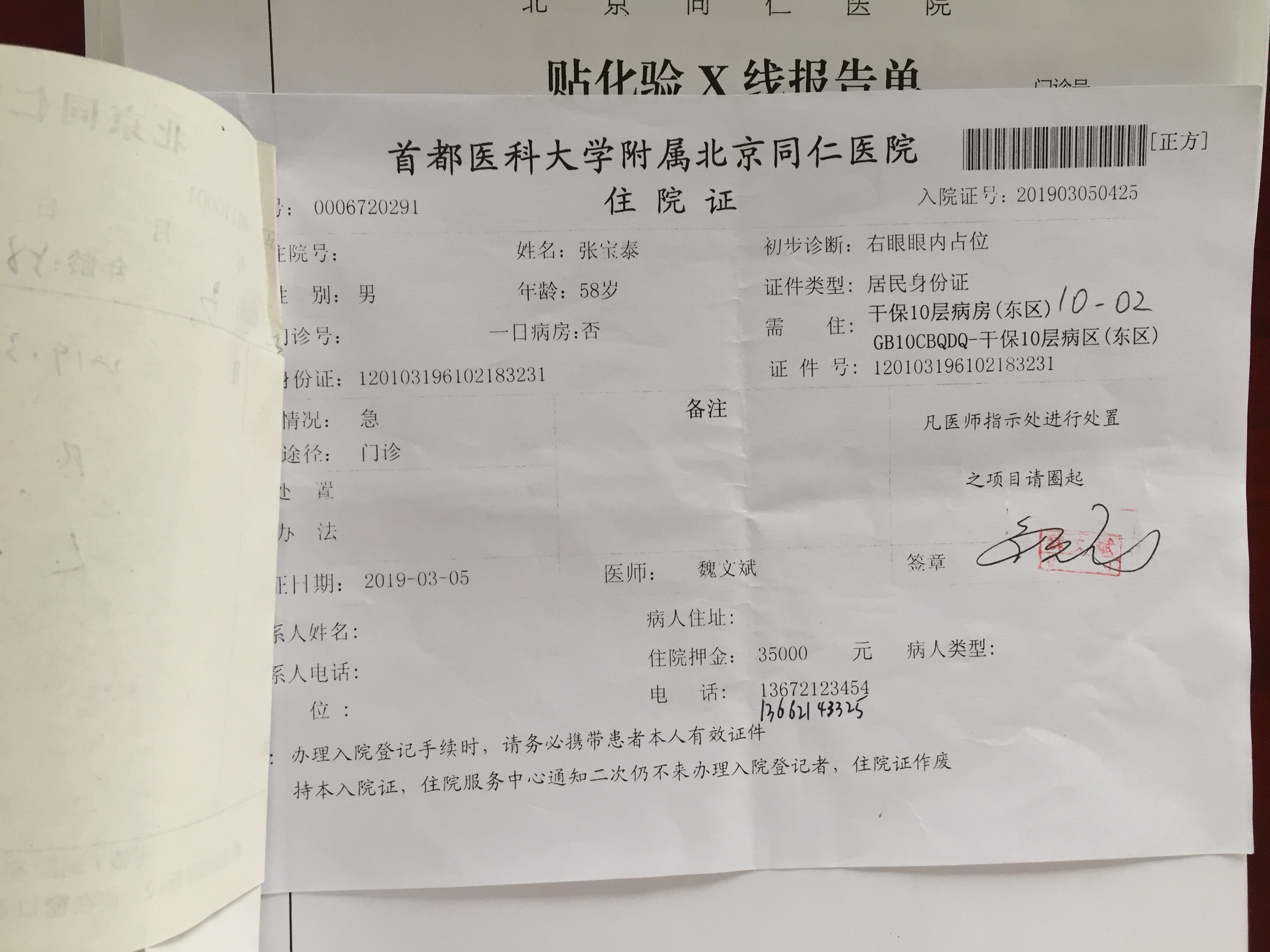

Supplement: Supplementary file 1 — Additional file 1: The raw data of this study. Table 1. The basic information of involved patients. [file 12886_2022_2598_MOESM1_ESM.zip › 2/IMG_8092.JPG]

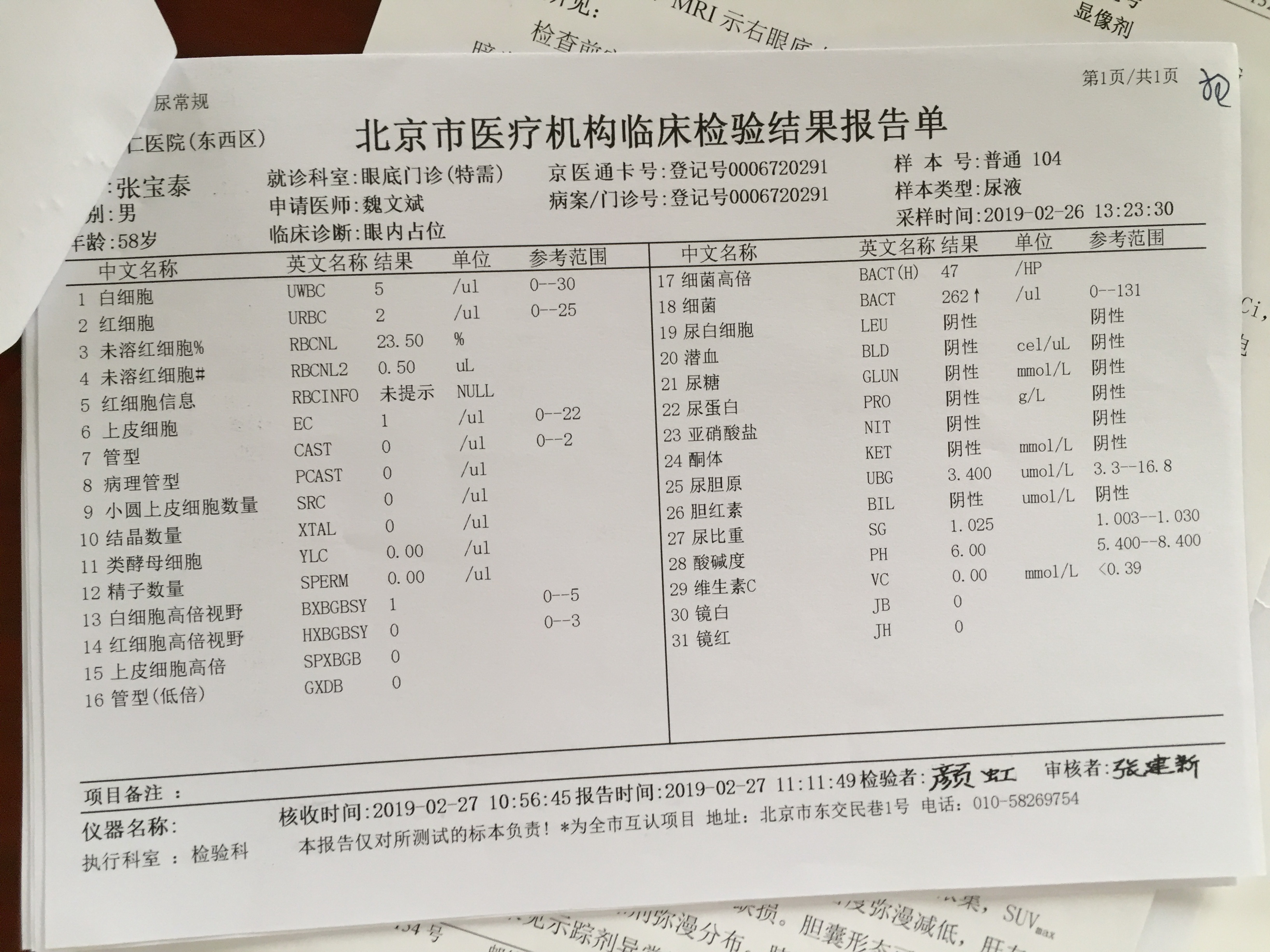

Supplement: Supplementary file 1 — Additional file 1: The raw data of this study. Table 1. The basic information of involved patients. [file 12886_2022_2598_MOESM1_ESM.zip › 2/IMG_8079.JPG]

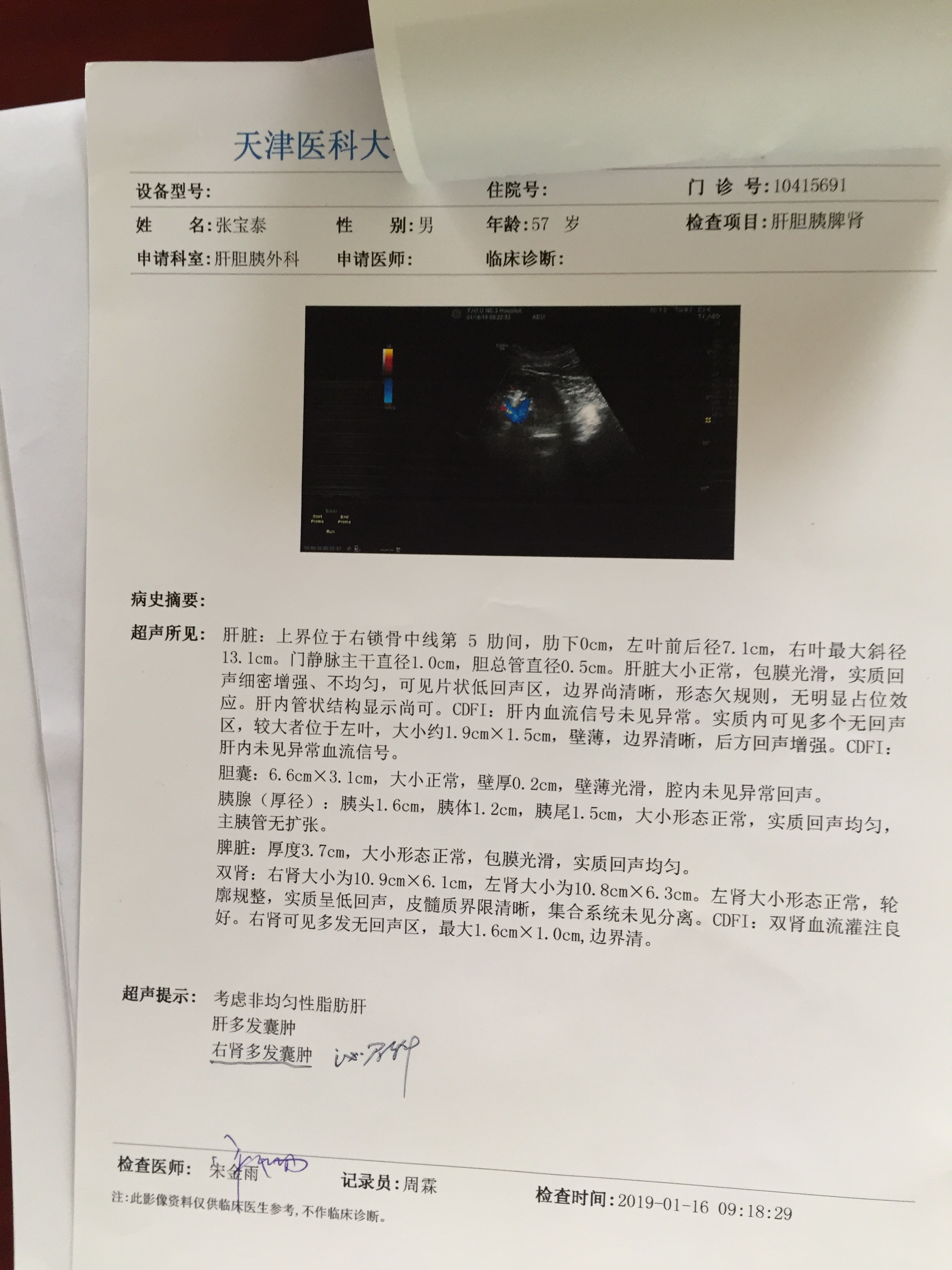

Supplement: Supplementary file 1 — Additional file 1: The raw data of this study. Table 1. The basic information of involved patients. [file 12886_2022_2598_MOESM1_ESM.zip › 2/IMG_8078.JPG]

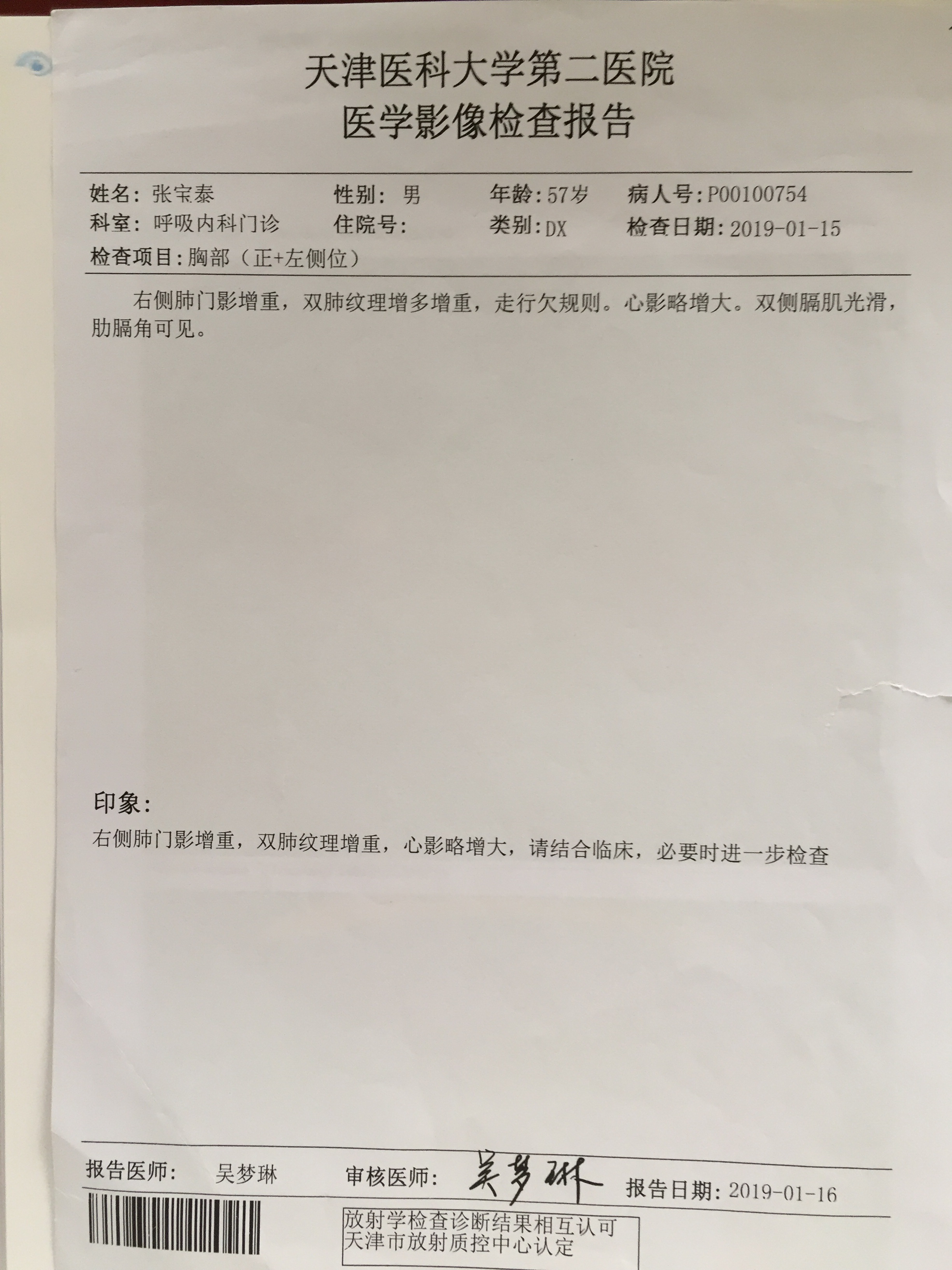

Supplement: Supplementary file 1 — Additional file 1: The raw data of this study. Table 1. The basic information of involved patients. [file 12886_2022_2598_MOESM1_ESM.zip › 2/IMG_8087.JPG]

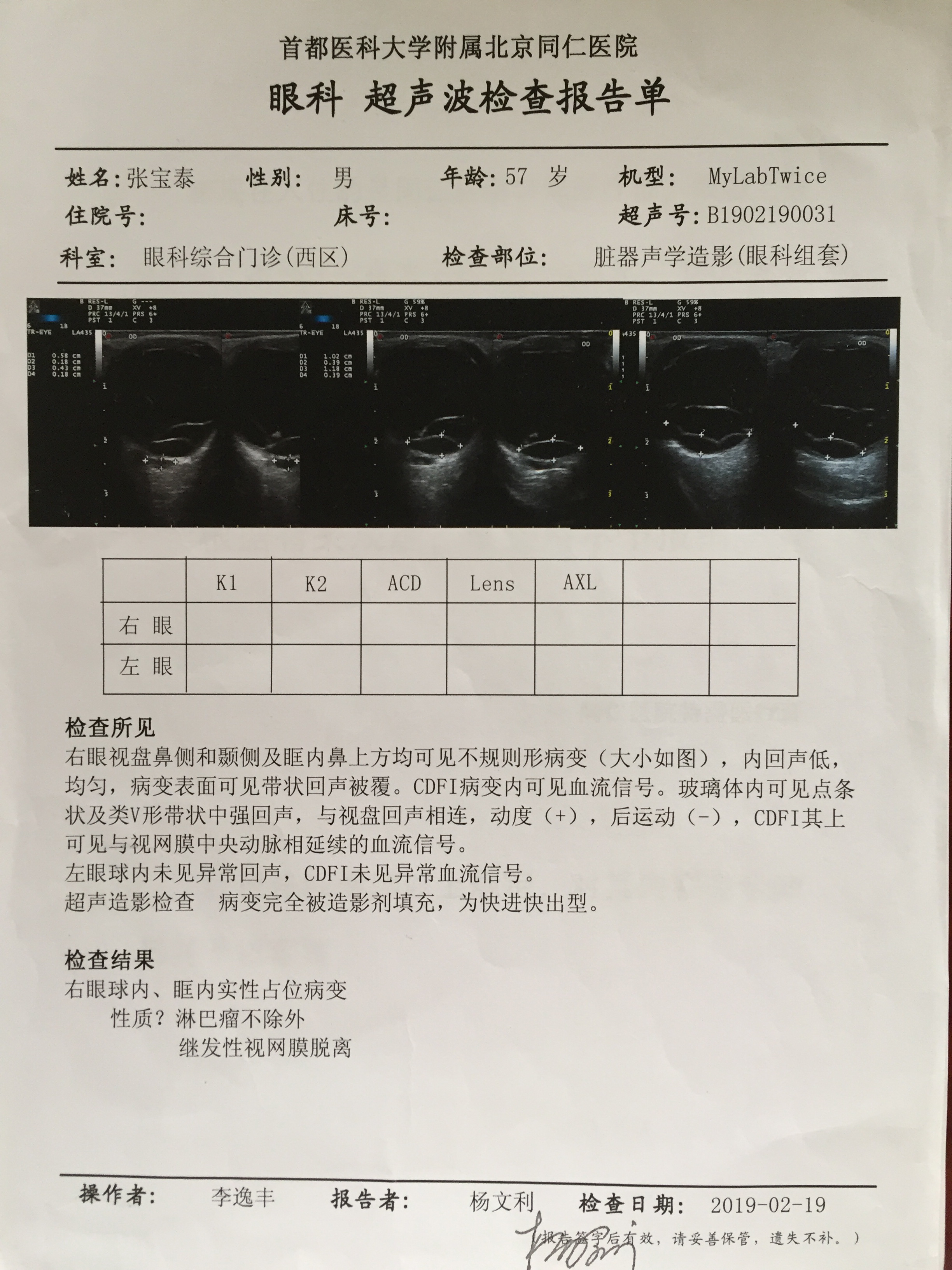

Supplement: Supplementary file 1 — Additional file 1: The raw data of this study. Table 1. The basic information of involved patients. [file 12886_2022_2598_MOESM1_ESM.zip › 2/IMG_8091.JPG]

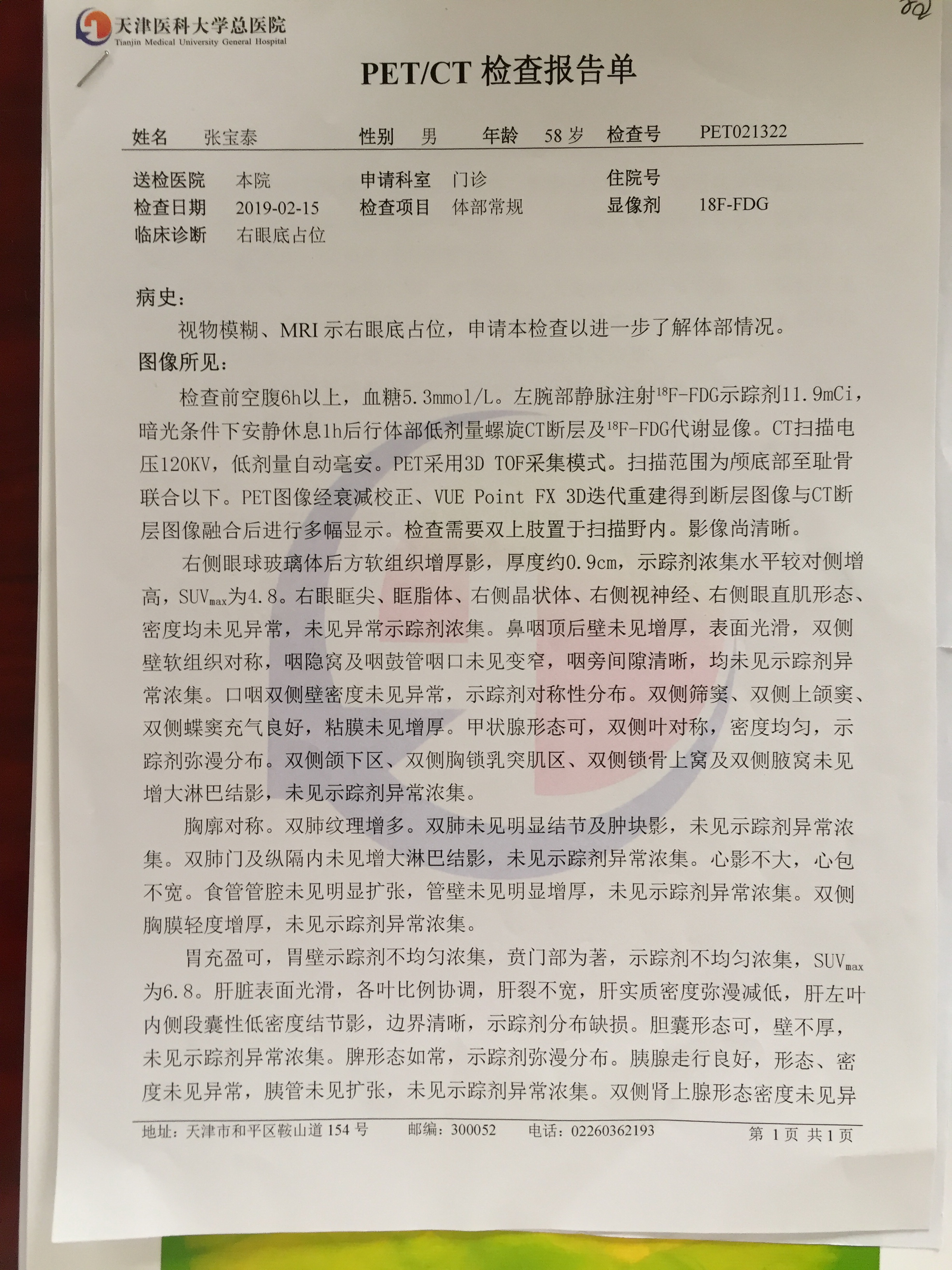

Supplement: Supplementary file 1 — Additional file 1: The raw data of this study. Table 1. The basic information of involved patients. [file 12886_2022_2598_MOESM1_ESM.zip › 2/IMG_8085.JPG]

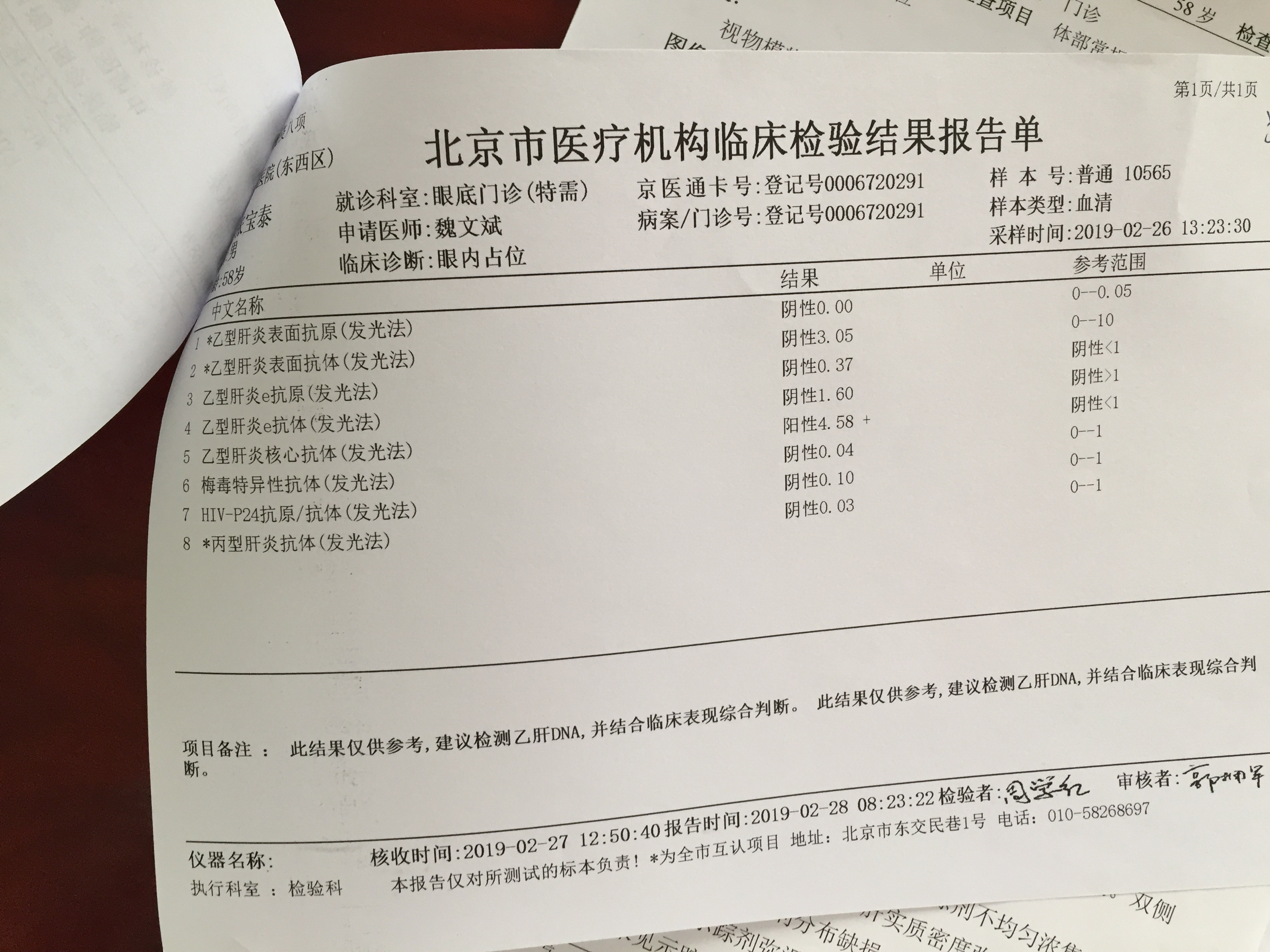

Supplement: Supplementary file 1 — Additional file 1: The raw data of this study. Table 1. The basic information of involved patients. [file 12886_2022_2598_MOESM1_ESM.zip › 2/IMG_8084.JPG]

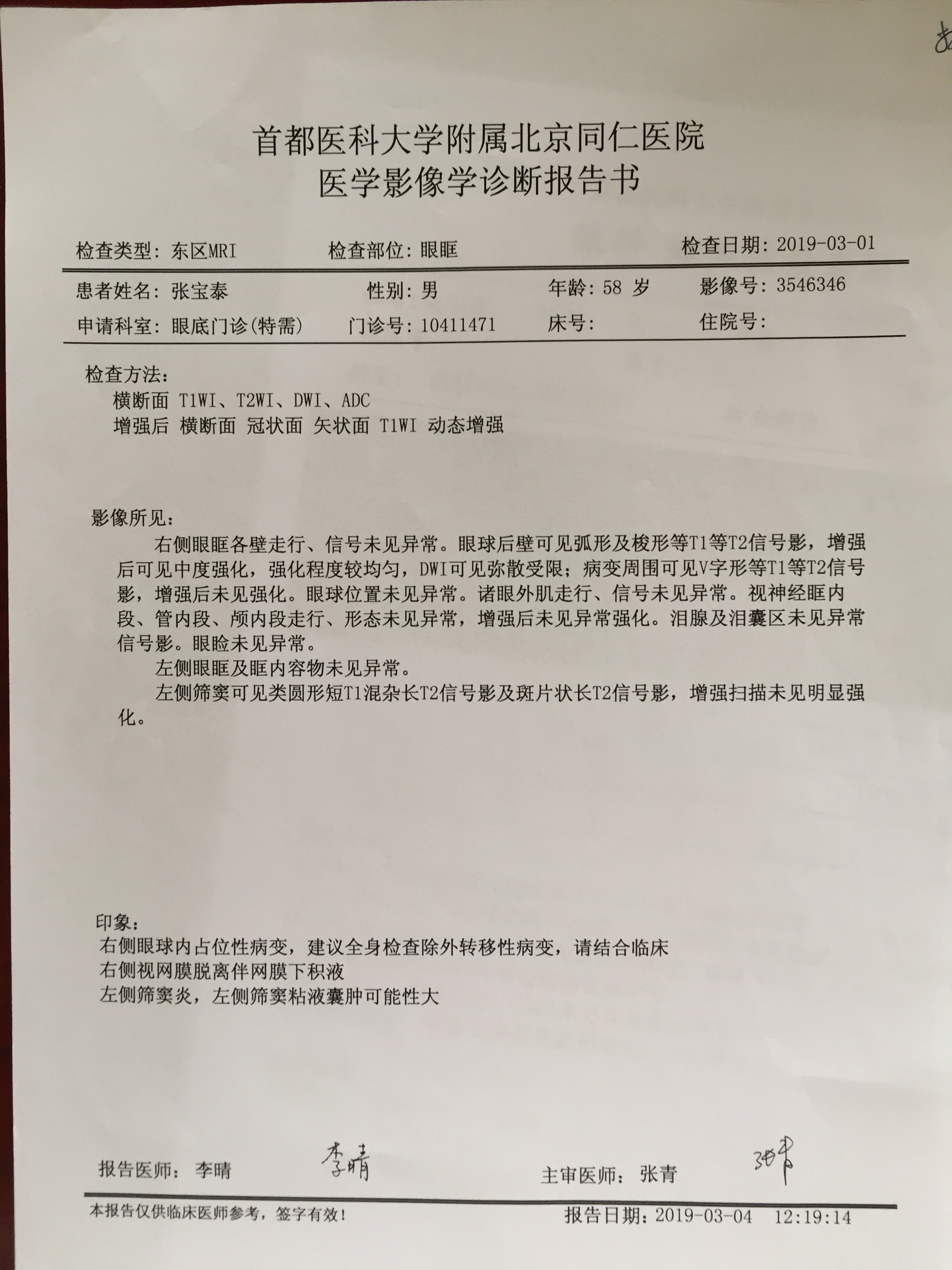

Supplement: Supplementary file 1 — Additional file 1: The raw data of this study. Table 1. The basic information of involved patients. [file 12886_2022_2598_MOESM1_ESM.zip › 2/IMG_8090.JPG]

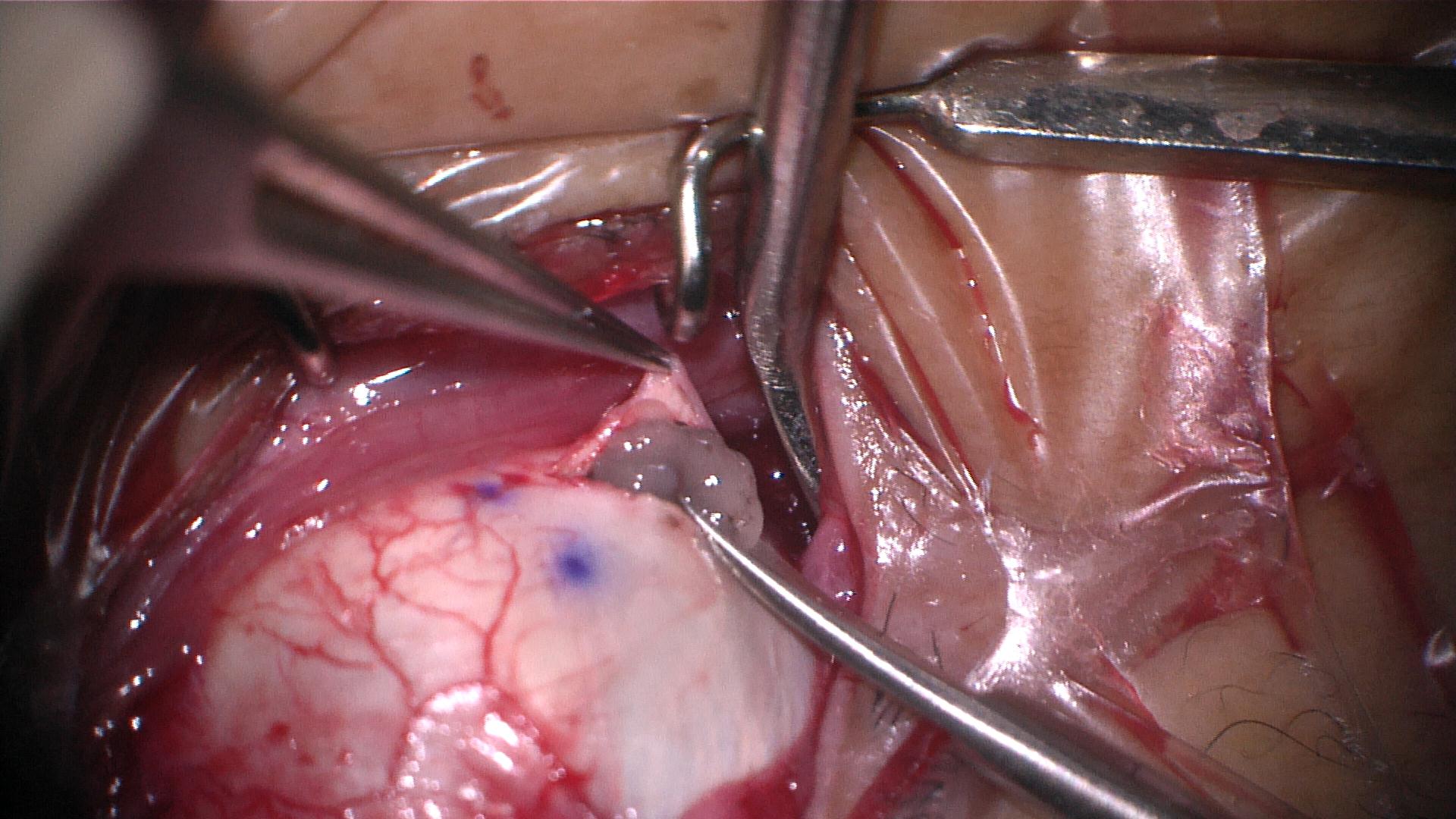

Supplement: Supplementary file 1 — Additional file 1: The raw data of this study. Table 1. The basic information of involved patients. [file 12886_2022_2598_MOESM1_ESM.zip › 2/0318101104396.jpg]

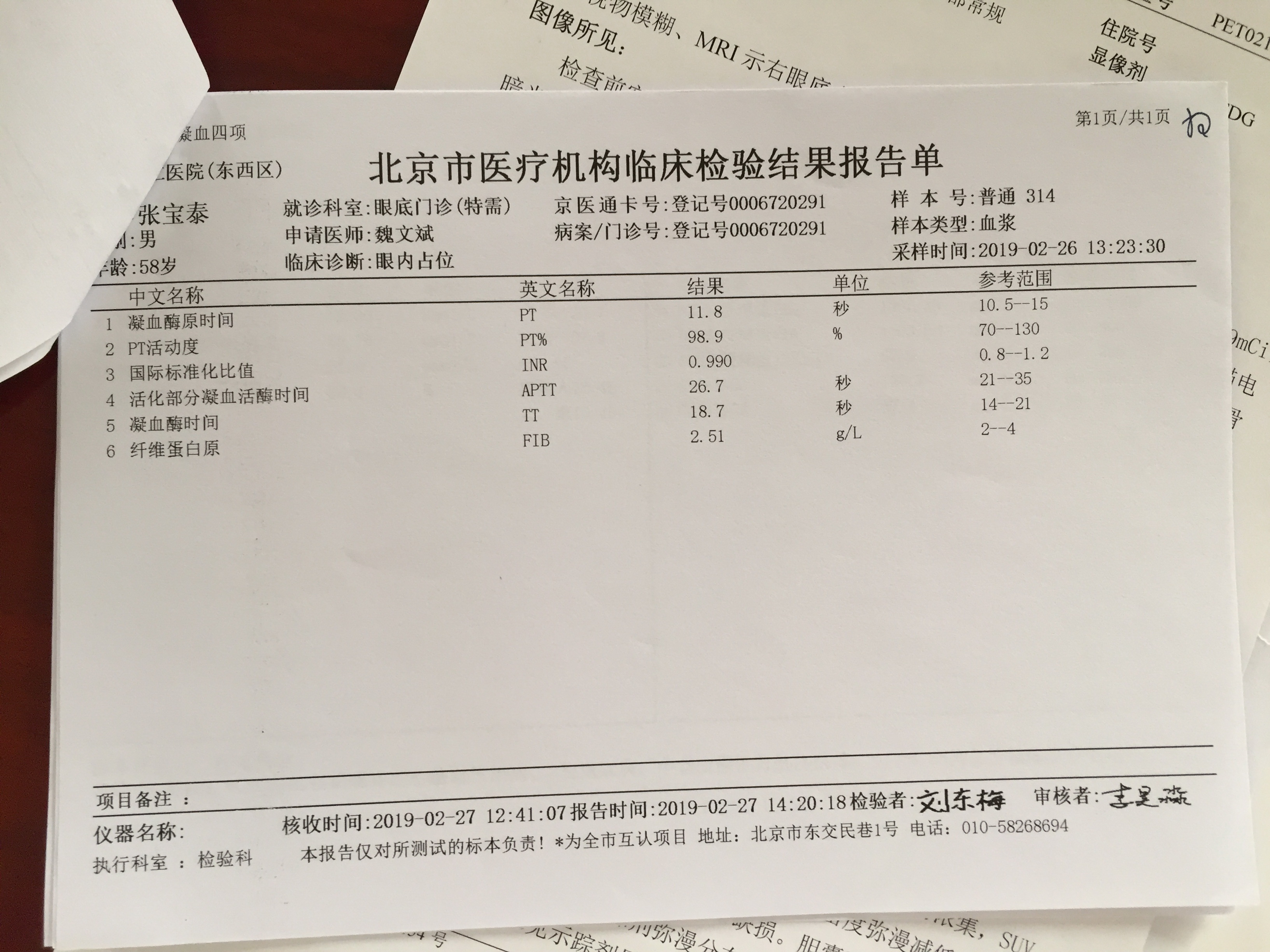

Supplement: Supplementary file 1 — Additional file 1: The raw data of this study. Table 1. The basic information of involved patients. [file 12886_2022_2598_MOESM1_ESM.zip › 2/IMG_8080.JPG]

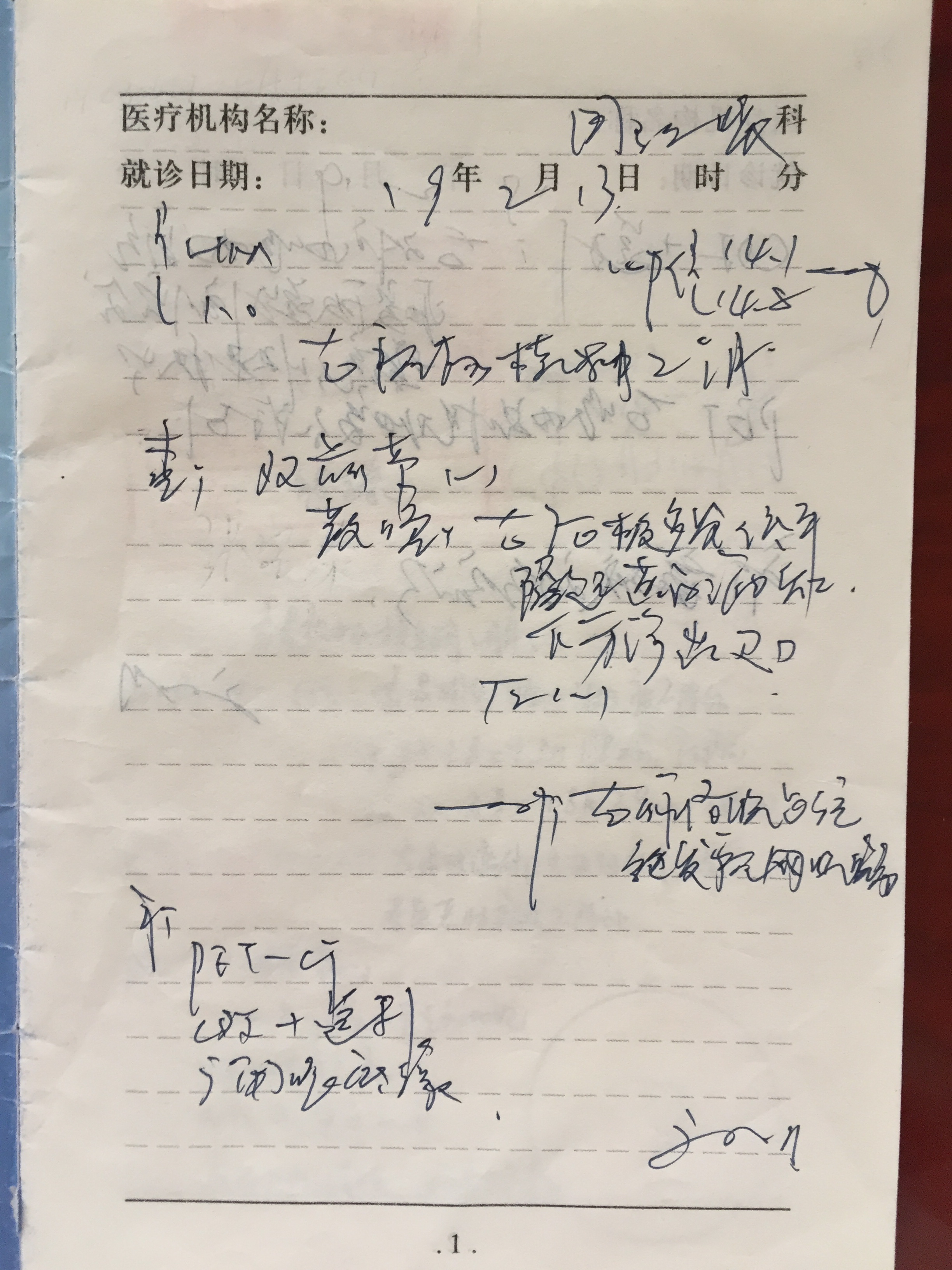

Supplement: Supplementary file 1 — Additional file 1: The raw data of this study. Table 1. The basic information of involved patients. [file 12886_2022_2598_MOESM1_ESM.zip › 2/IMG_8057.JPG]

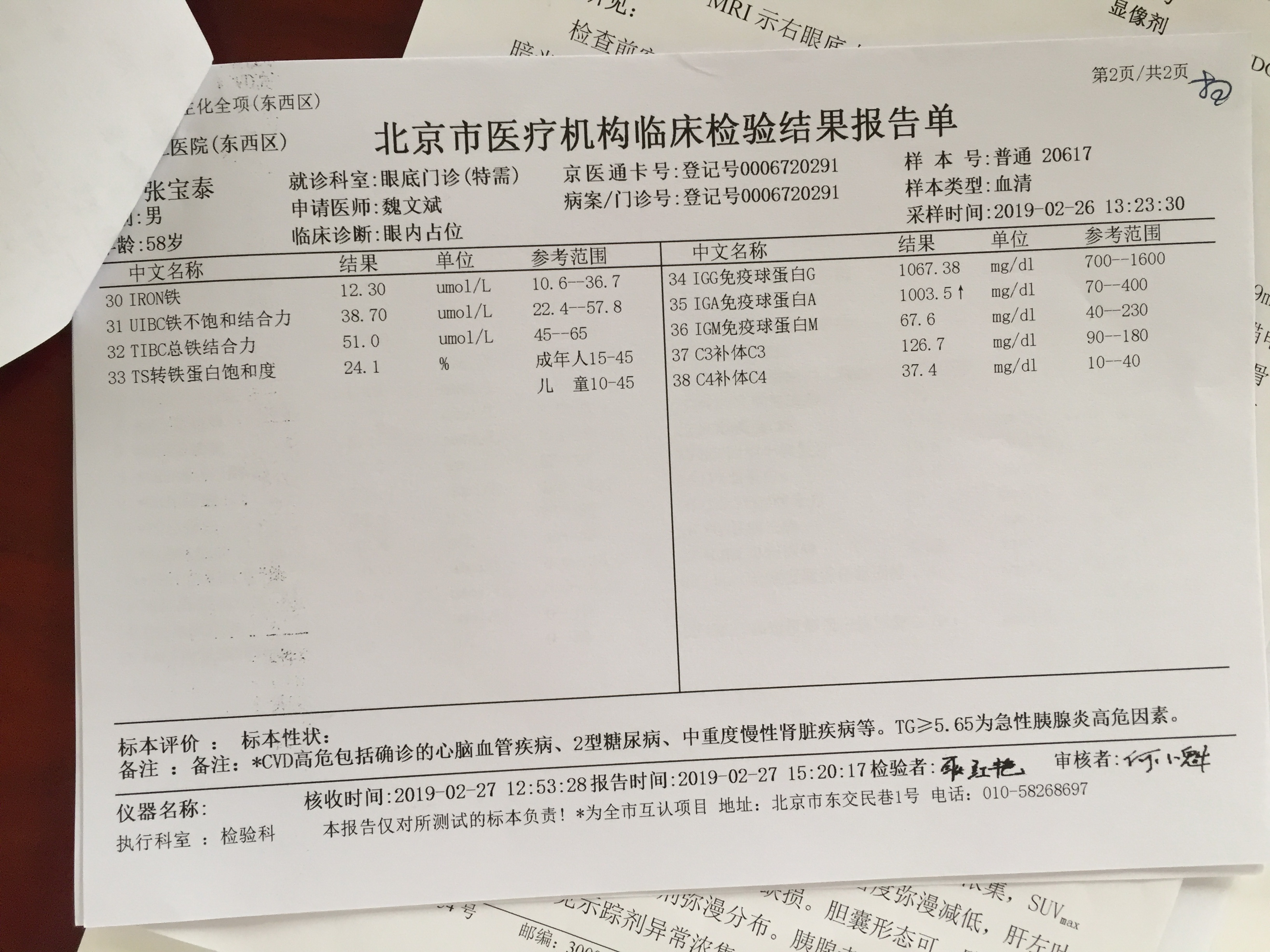

Supplement: Supplementary file 1 — Additional file 1: The raw data of this study. Table 1. The basic information of involved patients. [file 12886_2022_2598_MOESM1_ESM.zip › 2/IMG_8081.JPG]

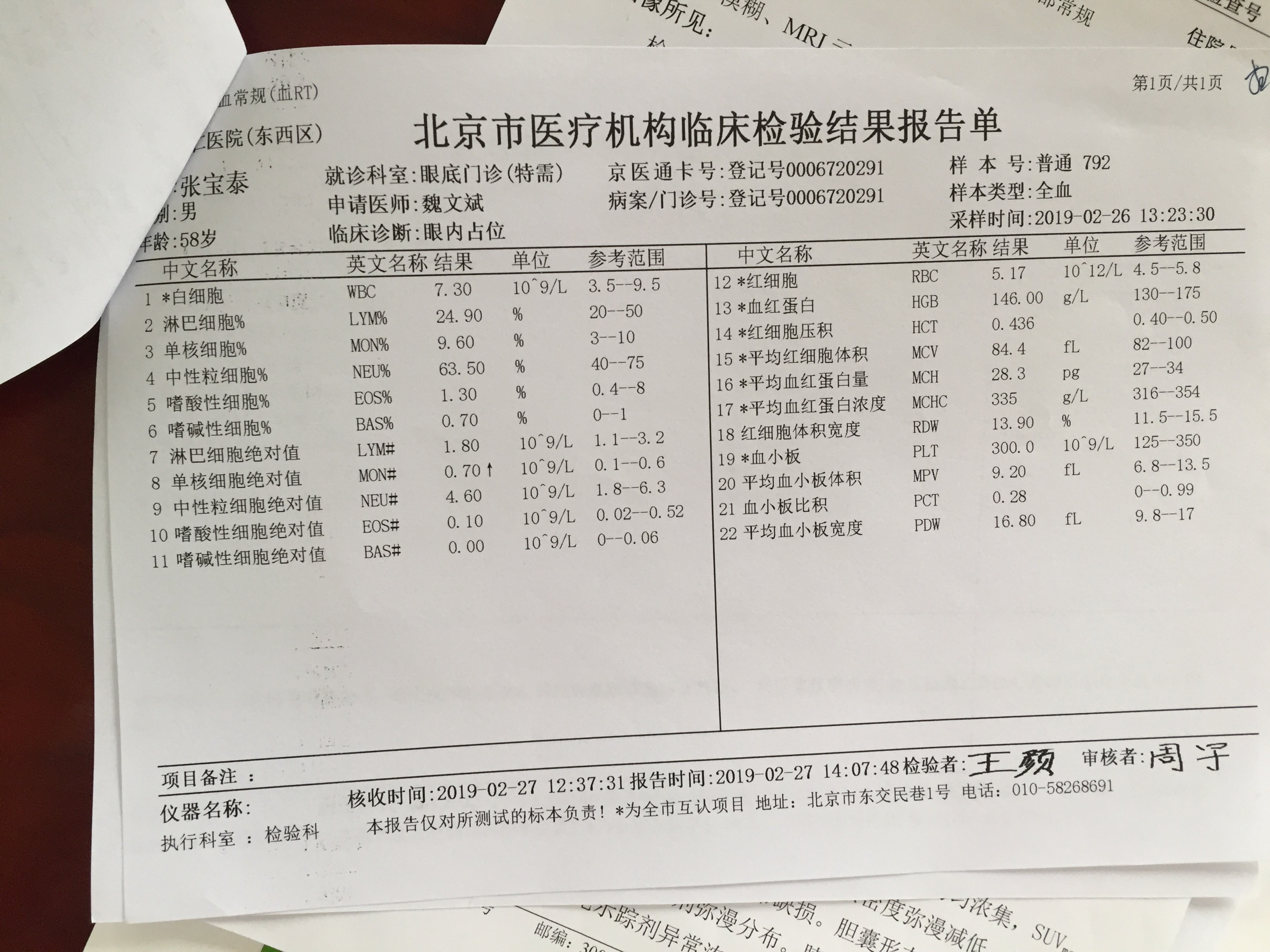

Supplement: Supplementary file 1 — Additional file 1: The raw data of this study. Table 1. The basic information of involved patients. [file 12886_2022_2598_MOESM1_ESM.zip › 2/IMG_8083.JPG]

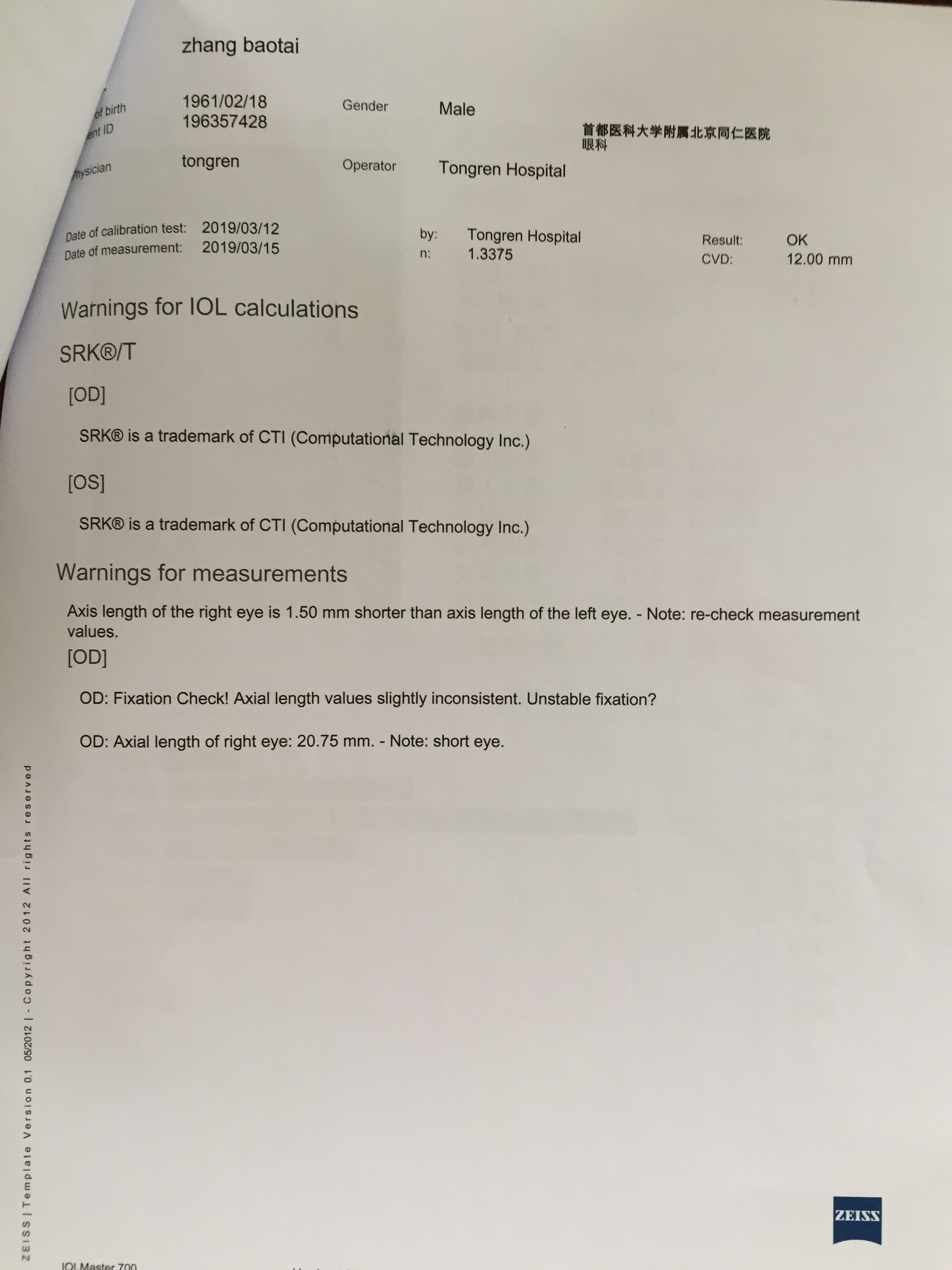

Supplement: Supplementary file 1 — Additional file 1: The raw data of this study. Table 1. The basic information of involved patients. [file 12886_2022_2598_MOESM1_ESM.zip › 2/IMG_8068.JPG]

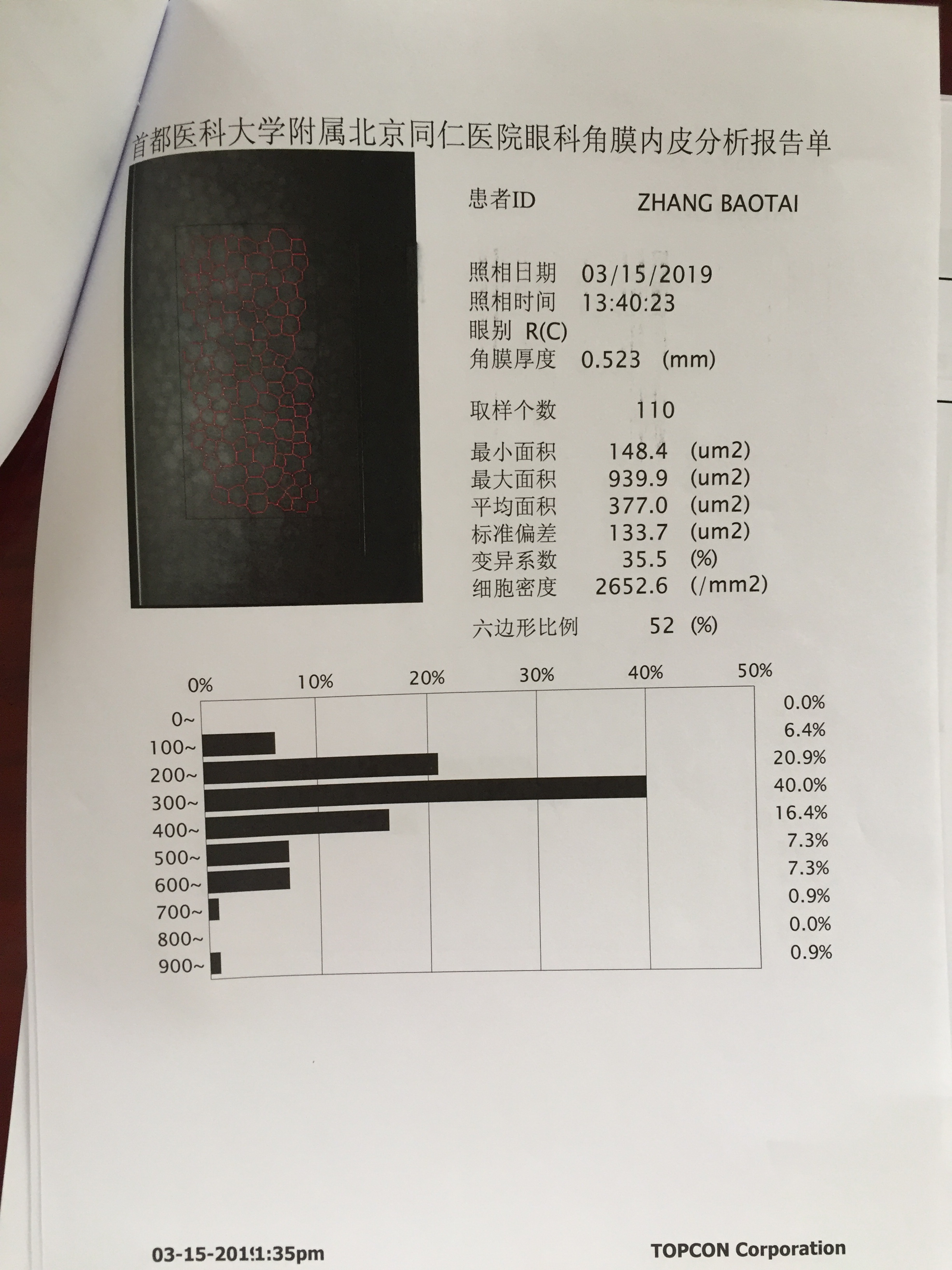

Supplement: Supplementary file 1 — Additional file 1: The raw data of this study. Table 1. The basic information of involved patients. [file 12886_2022_2598_MOESM1_ESM.zip › 2/IMG_8069.JPG]

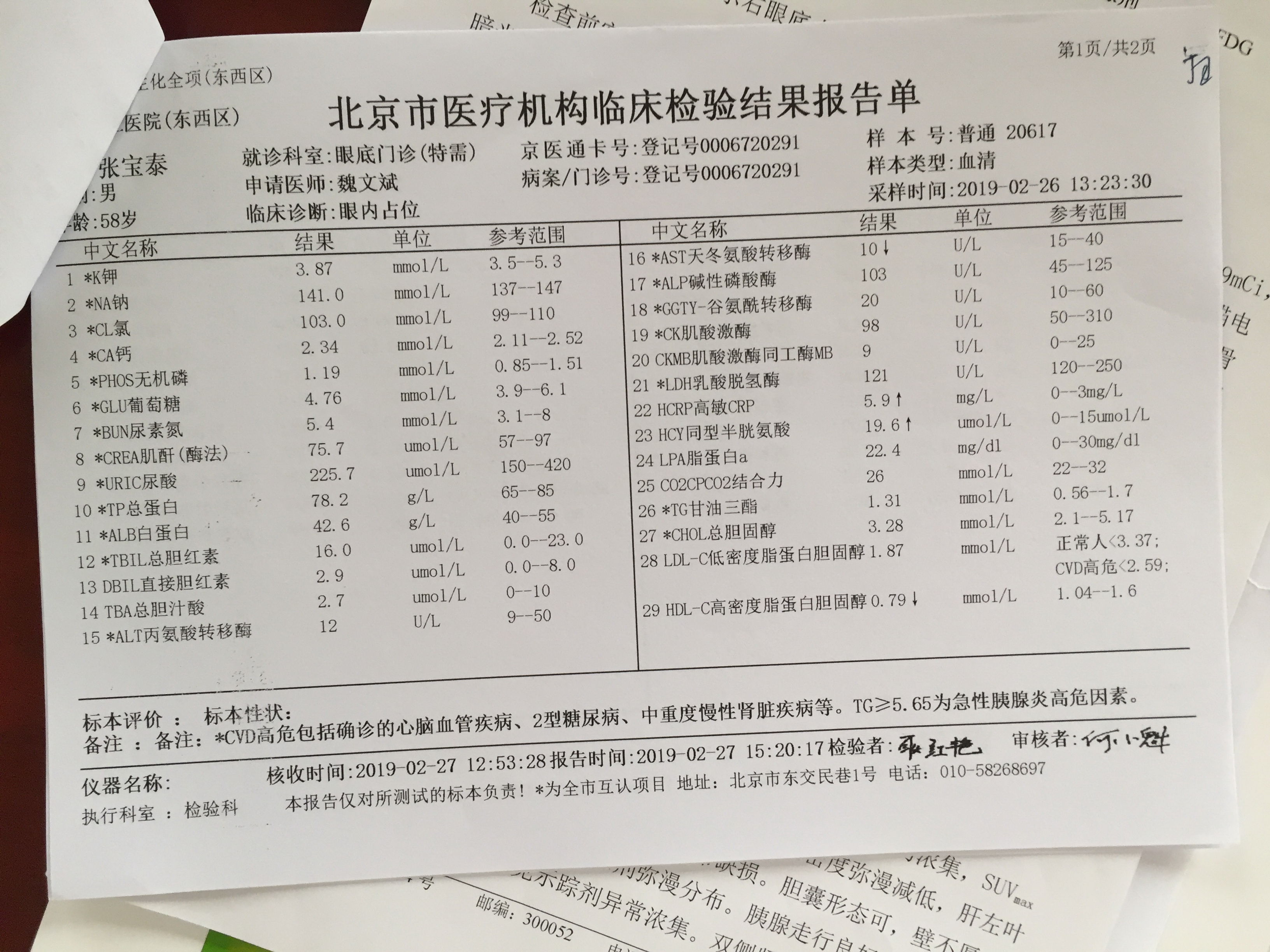

Supplement: Supplementary file 1 — Additional file 1: The raw data of this study. Table 1. The basic information of involved patients. [file 12886_2022_2598_MOESM1_ESM.zip › 2/IMG_8082.JPG]

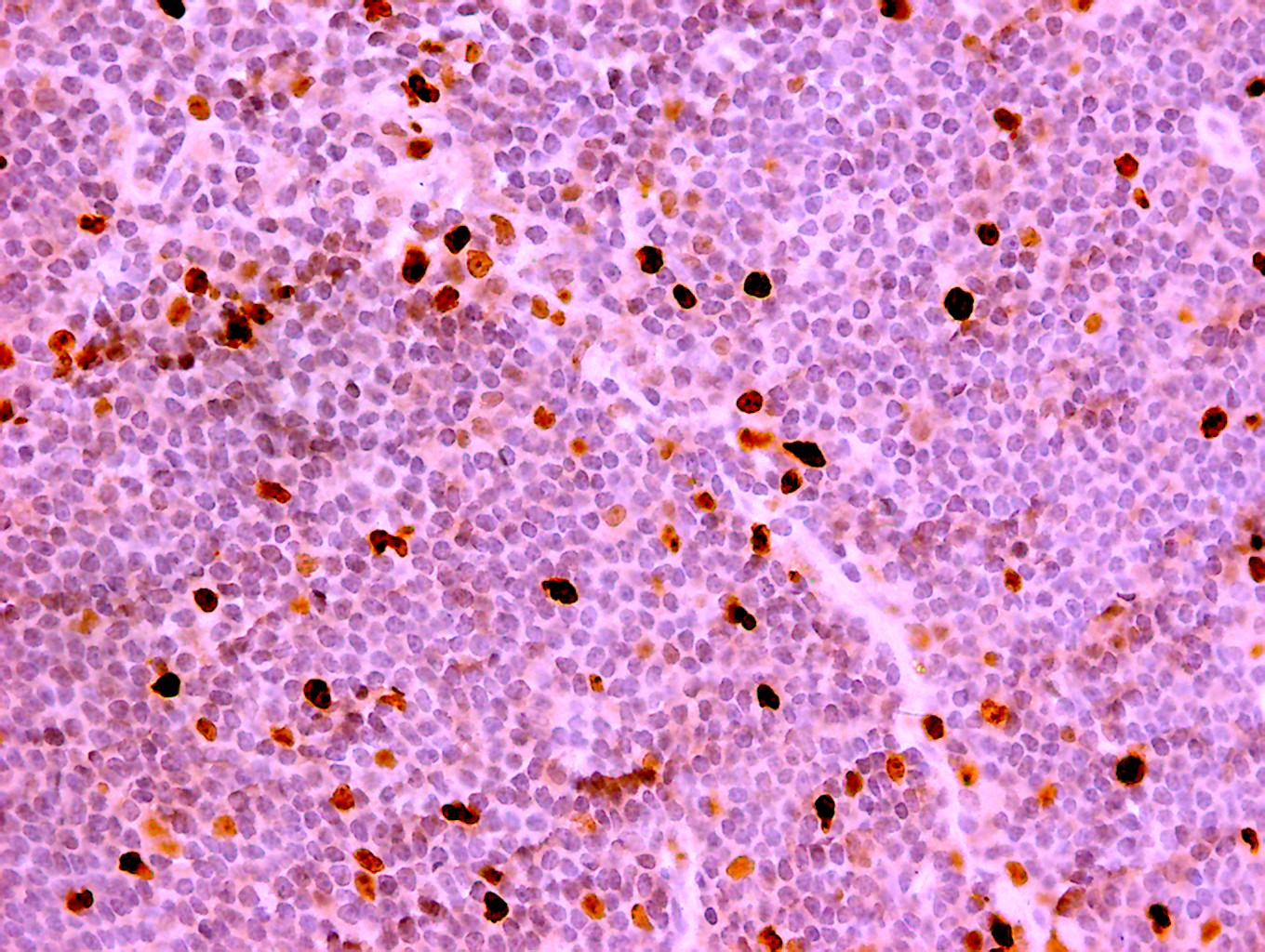

Supplement: Supplementary file 1 — Additional file 1: The raw data of this study. Table 1. The basic information of involved patients. [file 12886_2022_2598_MOESM1_ESM.zip › 2/σ╝áσ«¥μ│░/3-ki-67-IHC-40.jpg]

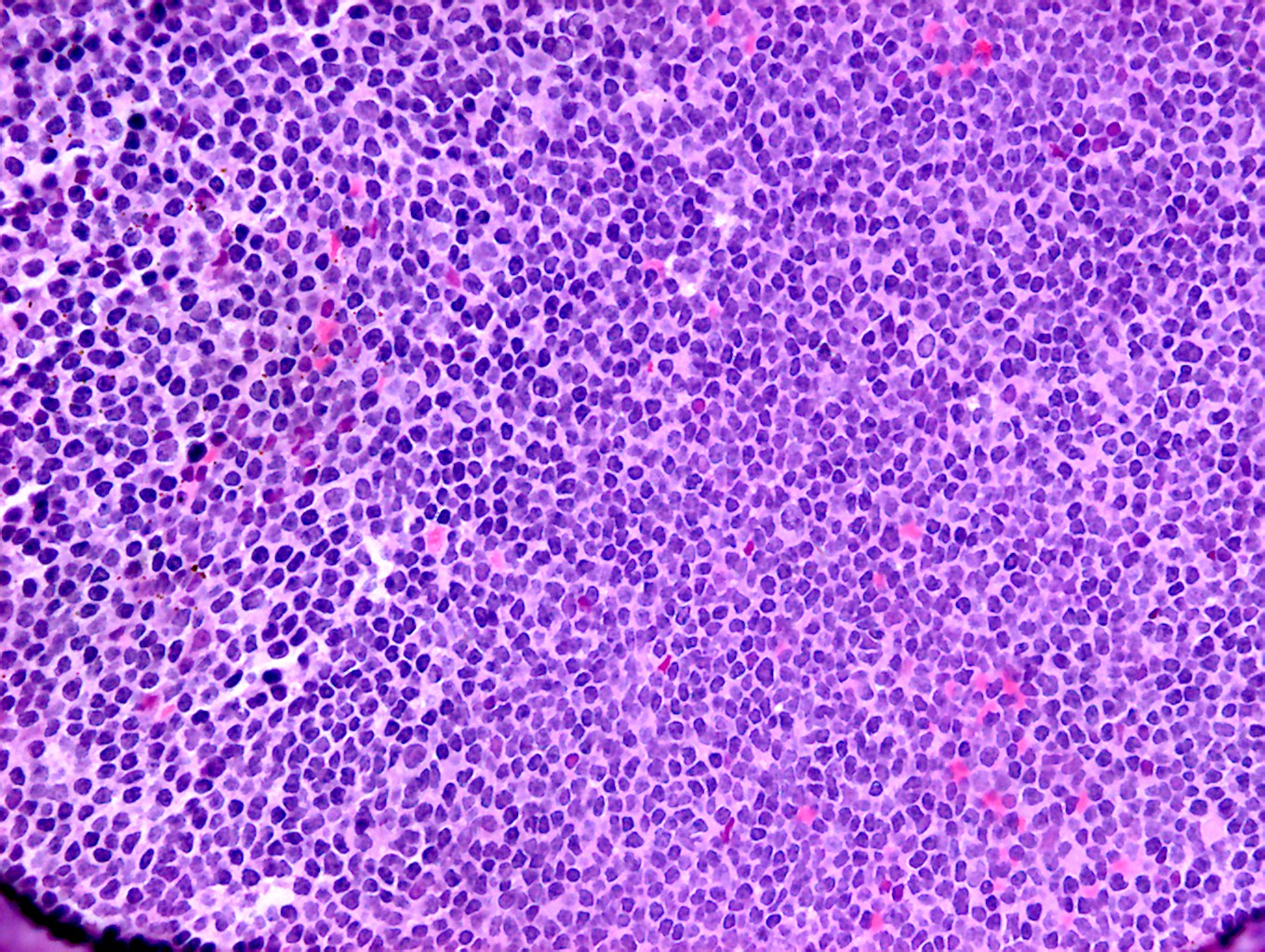

Supplement: Supplementary file 1 — Additional file 1: The raw data of this study. Table 1. The basic information of involved patients. [file 12886_2022_2598_MOESM1_ESM.zip › 2/σ╝áσ«¥μ│░/1-HE-40.jpg]

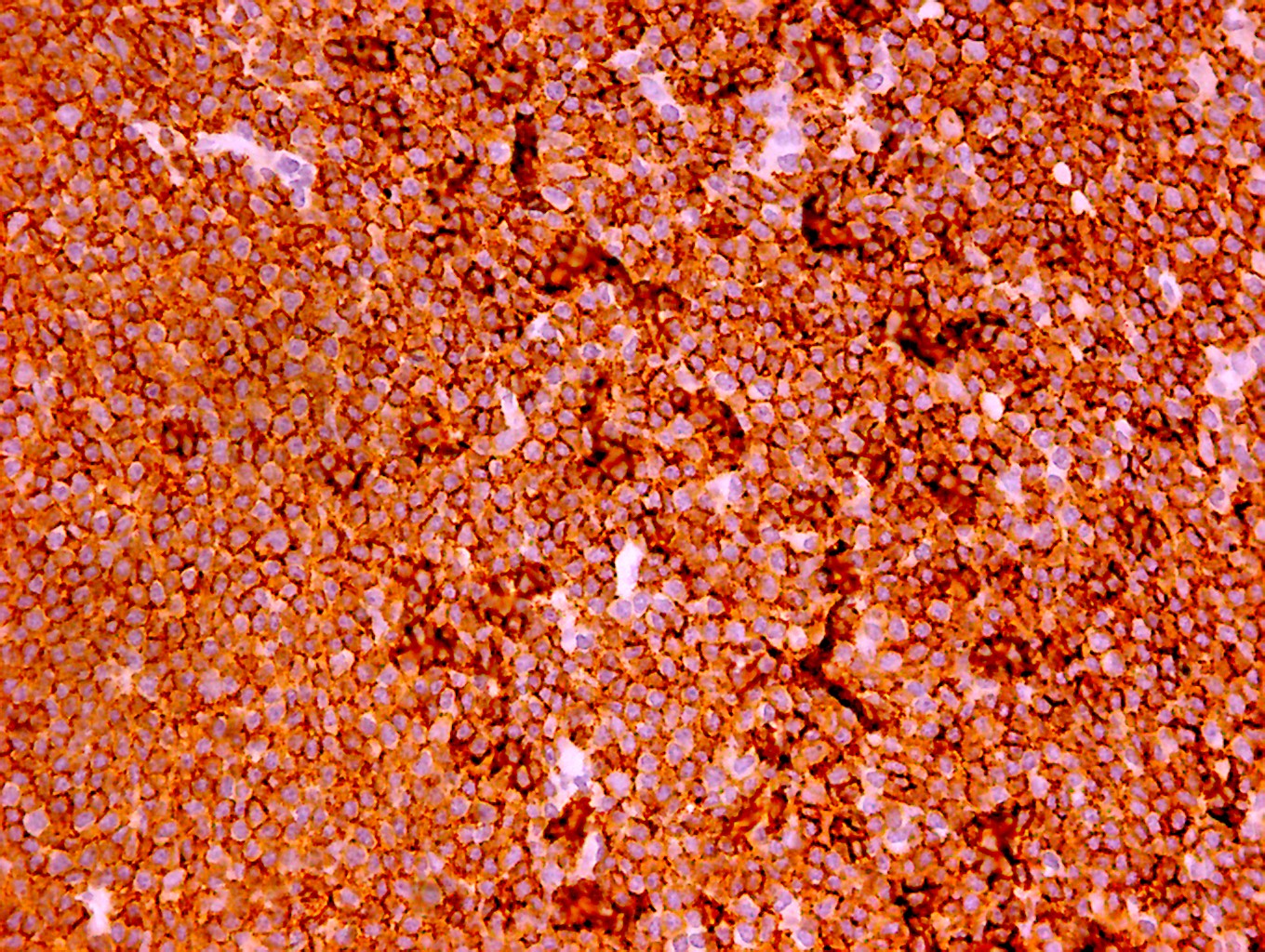

Supplement: Supplementary file 1 — Additional file 1: The raw data of this study. Table 1. The basic information of involved patients. [file 12886_2022_2598_MOESM1_ESM.zip › 2/σ╝áσ«¥μ│░/2-CD20-IHC-40.jpg]

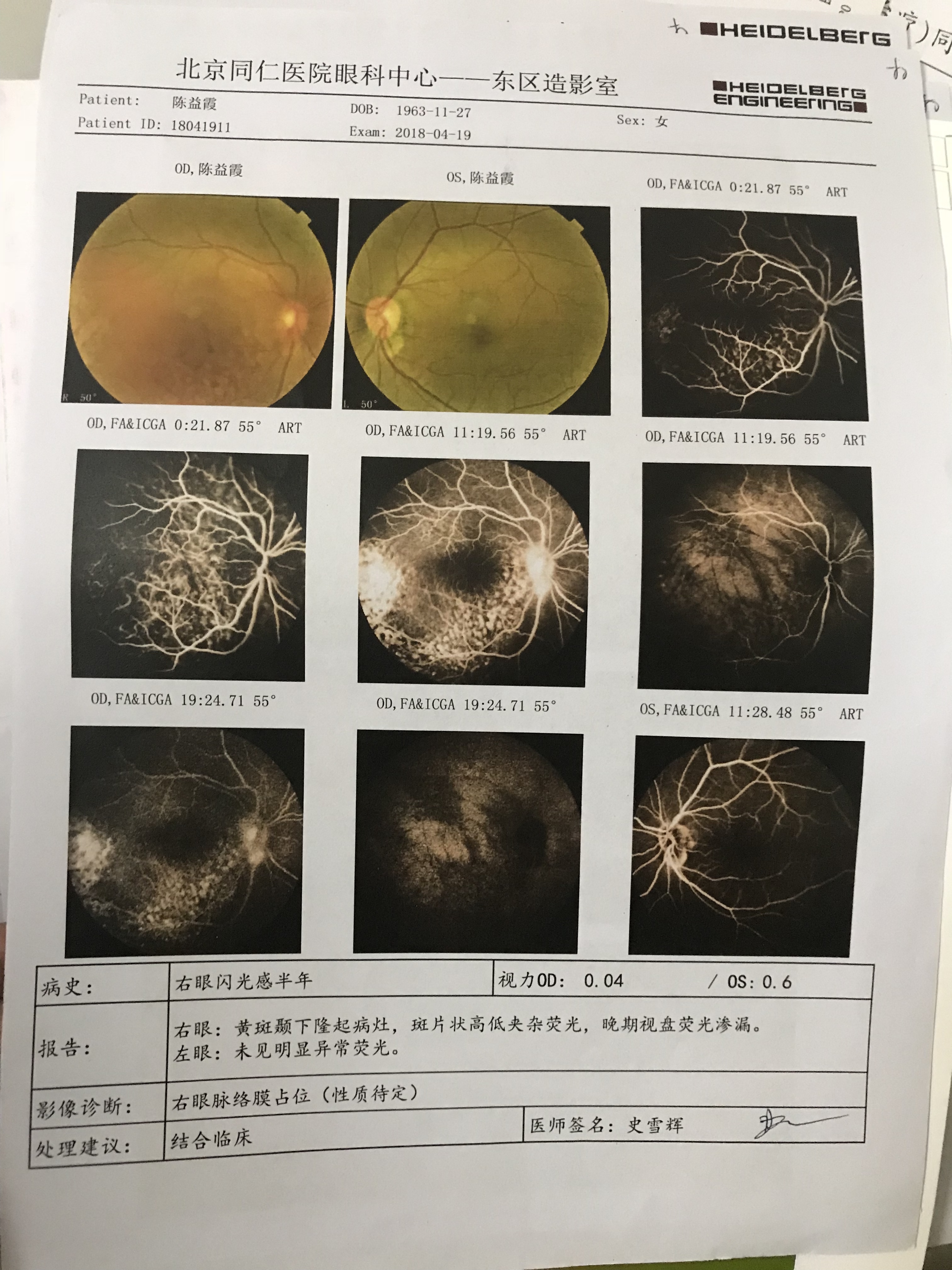

Supplement: Supplementary file 1 — Additional file 1: The raw data of this study. Table 1. The basic information of involved patients. [file 12886_2022_2598_MOESM1_ESM.zip › 3/IMG_4919.JPG]

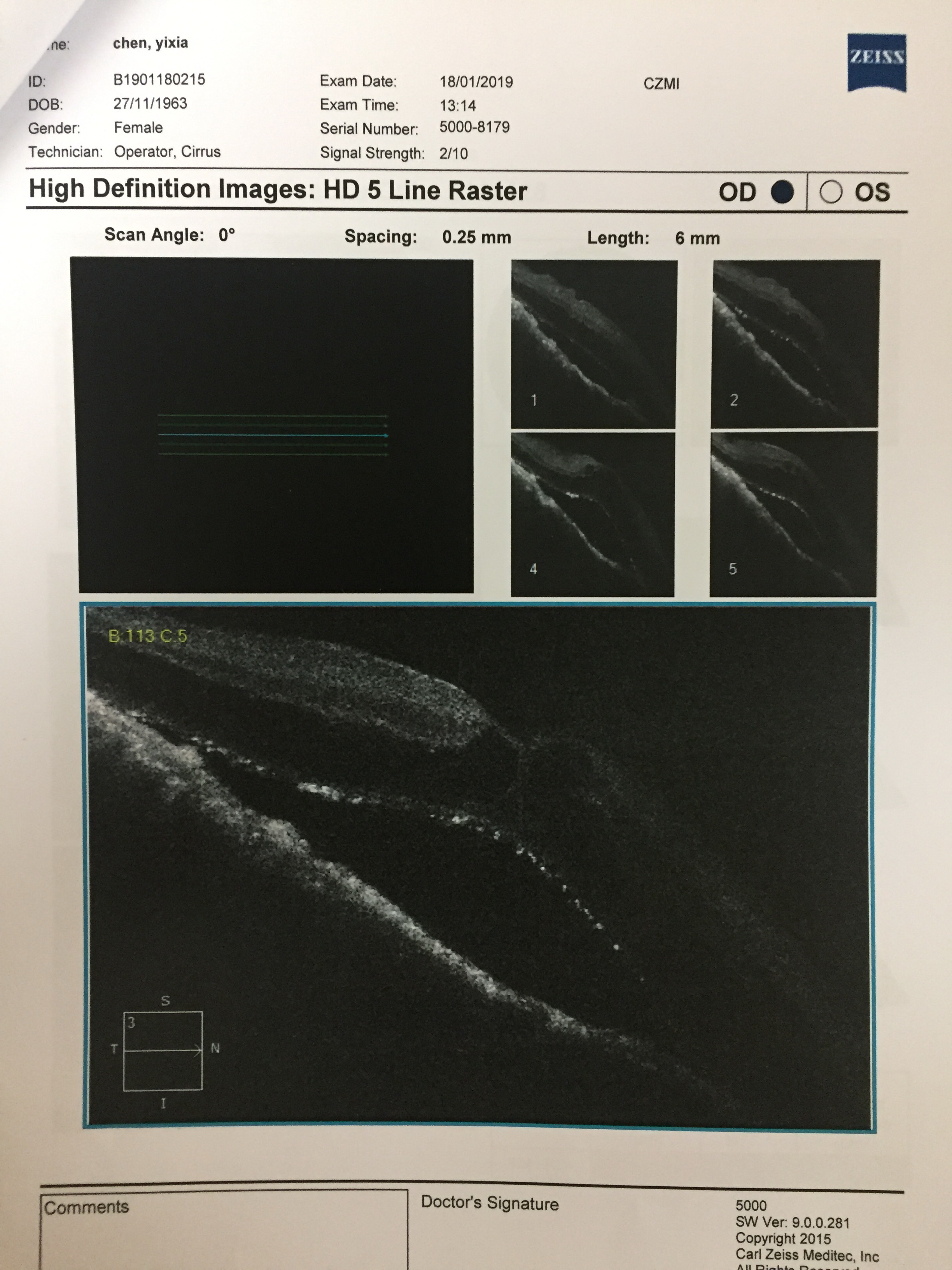

Supplement: Supplementary file 1 — Additional file 1: The raw data of this study. Table 1. The basic information of involved patients. [file 12886_2022_2598_MOESM1_ESM.zip › 3/IMG_6707.JPG]

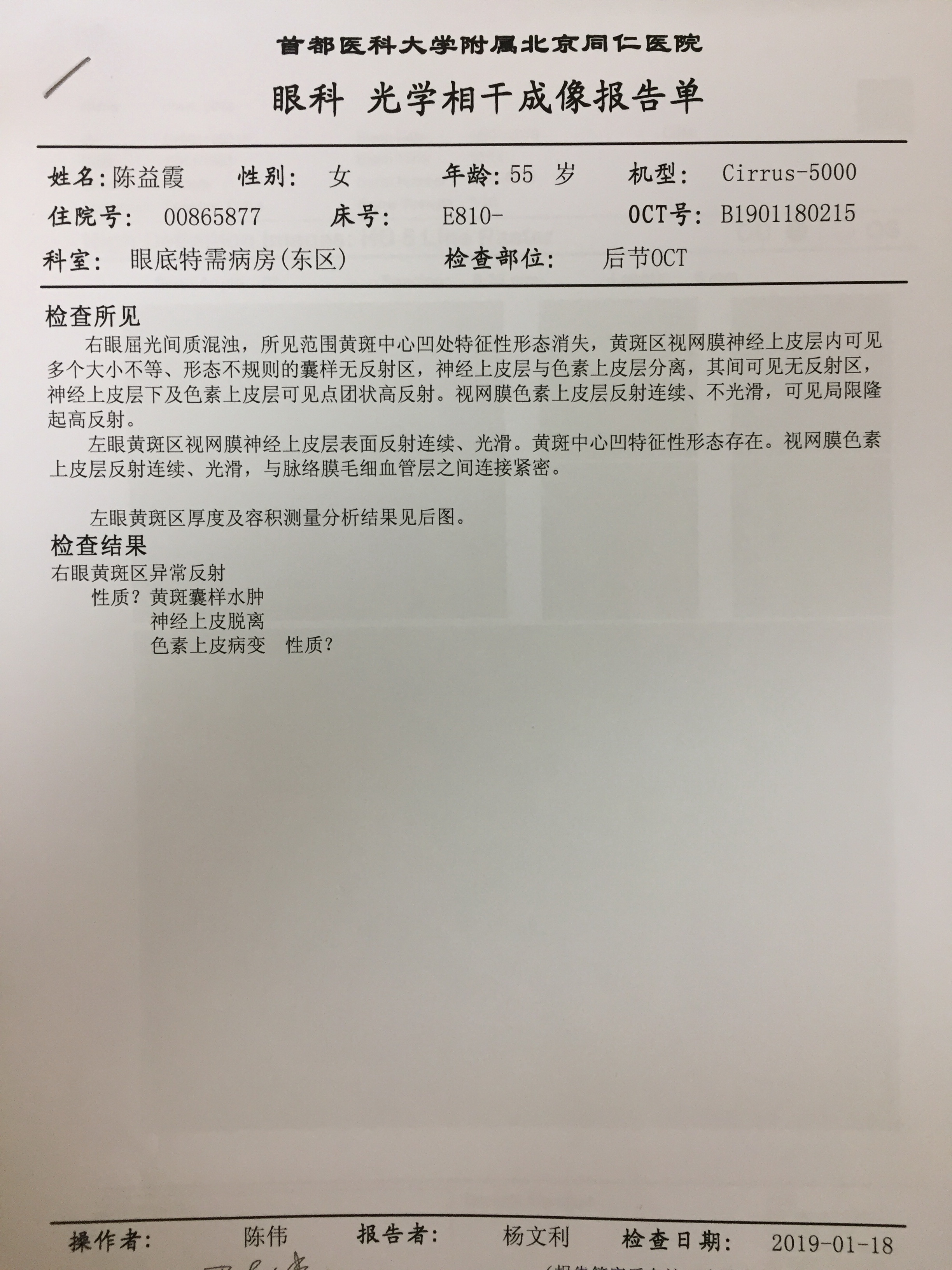

Supplement: Supplementary file 1 — Additional file 1: The raw data of this study. Table 1. The basic information of involved patients. [file 12886_2022_2598_MOESM1_ESM.zip › 3/IMG_6706.JPG]

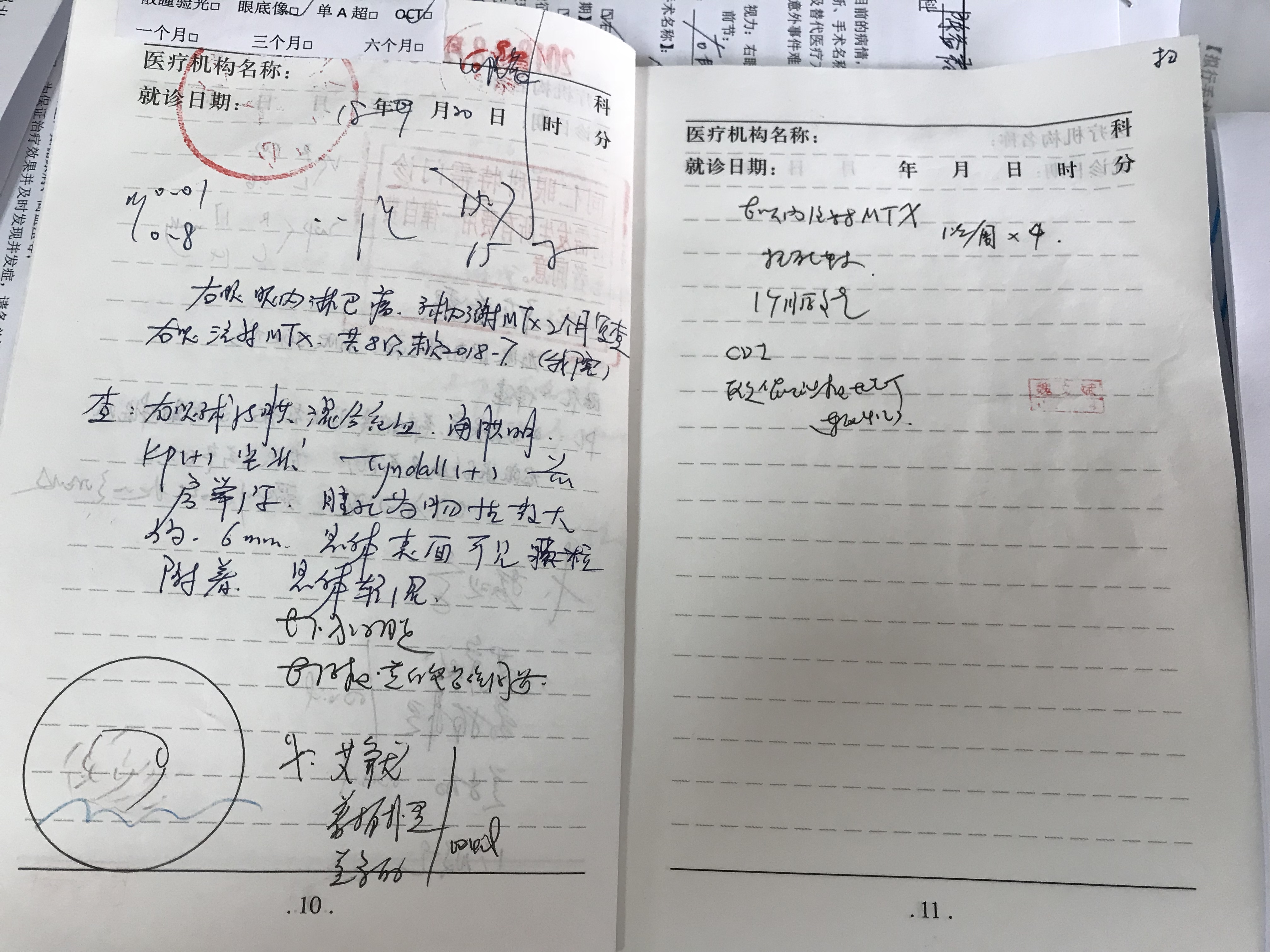

Supplement: Supplementary file 1 — Additional file 1: The raw data of this study. Table 1. The basic information of involved patients. [file 12886_2022_2598_MOESM1_ESM.zip › 3/20180920.JPG]

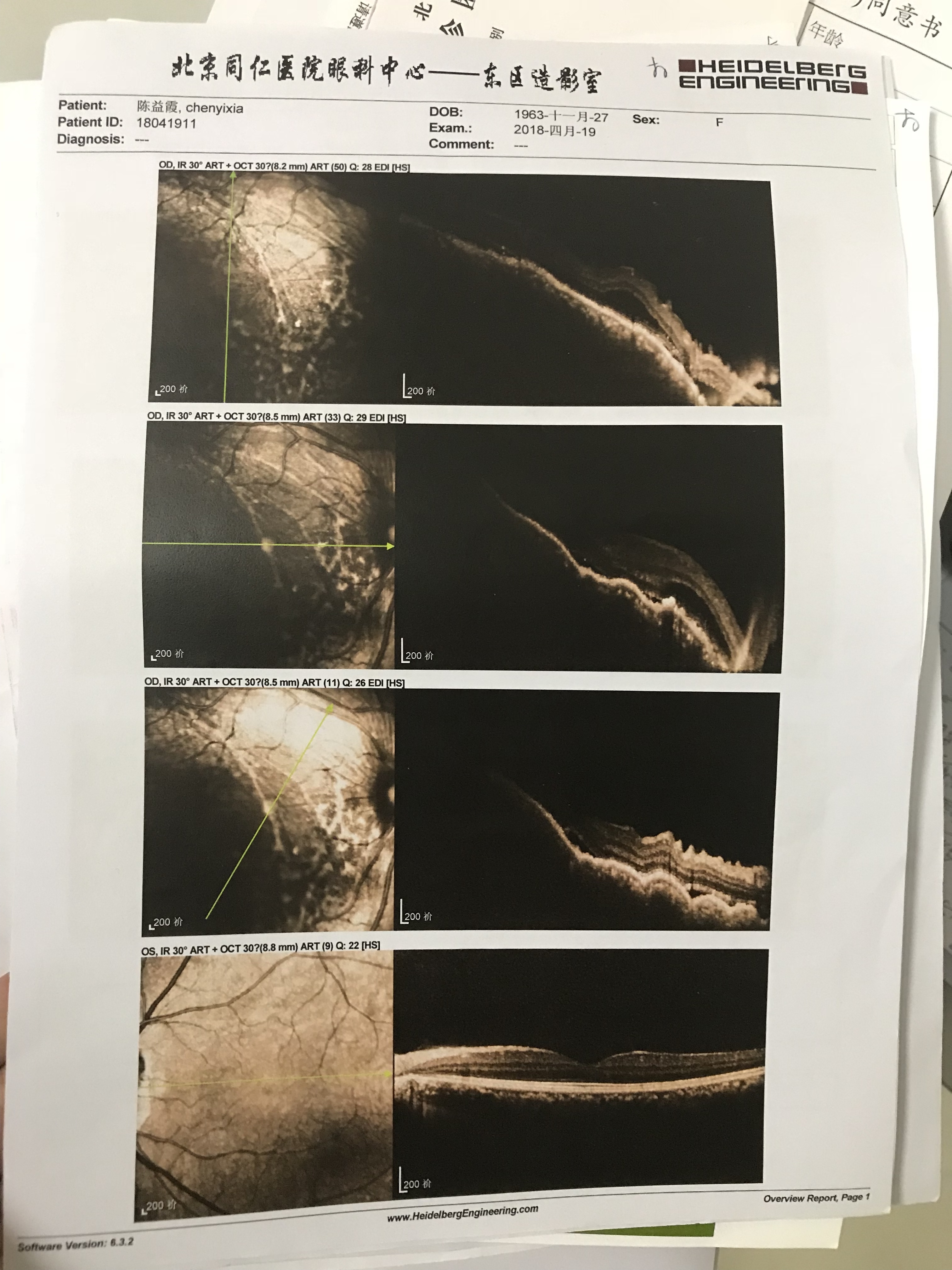

Supplement: Supplementary file 1 — Additional file 1: The raw data of this study. Table 1. The basic information of involved patients. [file 12886_2022_2598_MOESM1_ESM.zip › 3/IMG_4918.JPG]

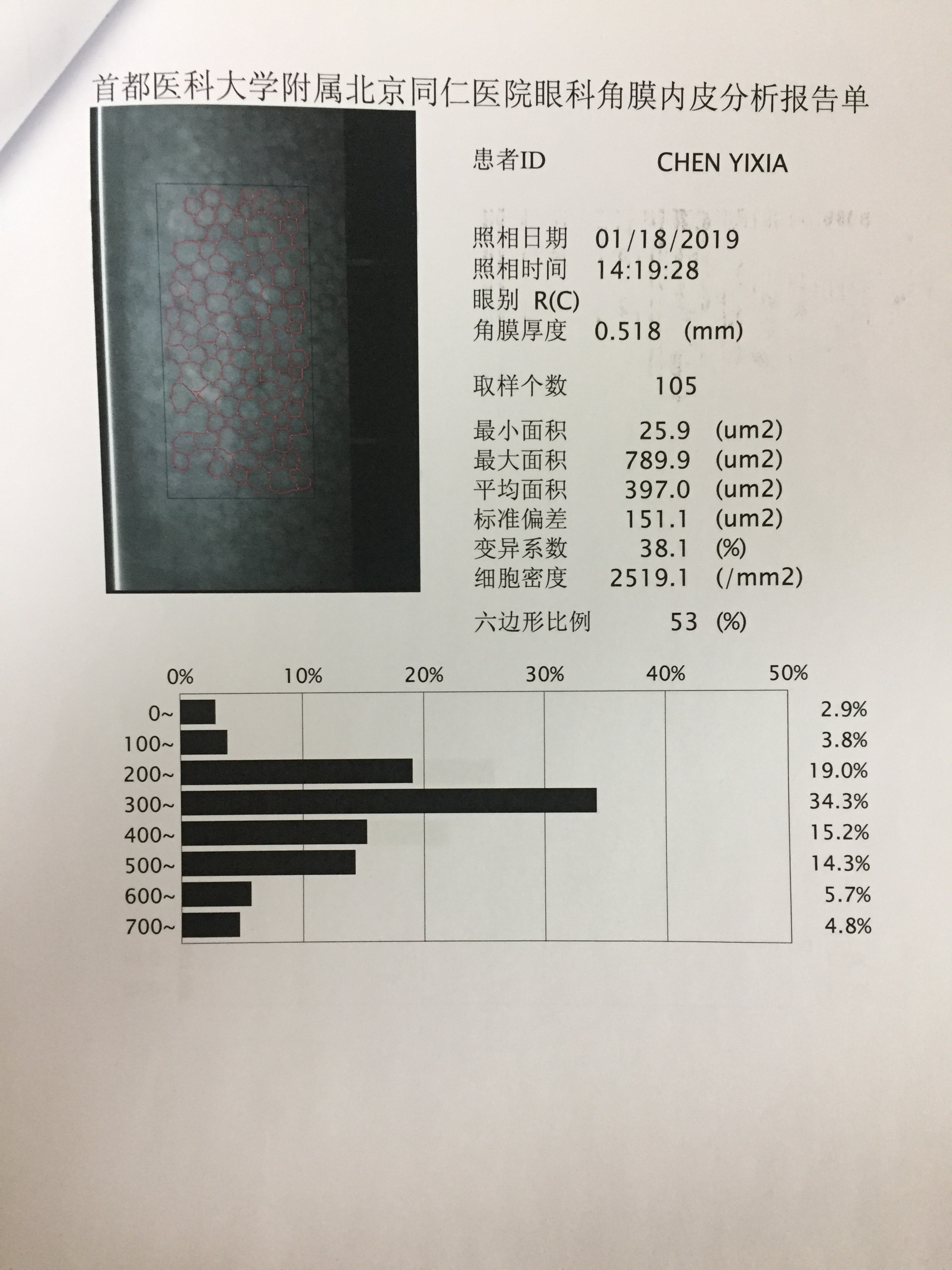

Supplement: Supplementary file 1 — Additional file 1: The raw data of this study. Table 1. The basic information of involved patients. [file 12886_2022_2598_MOESM1_ESM.zip › 3/IMG_6704.JPG]

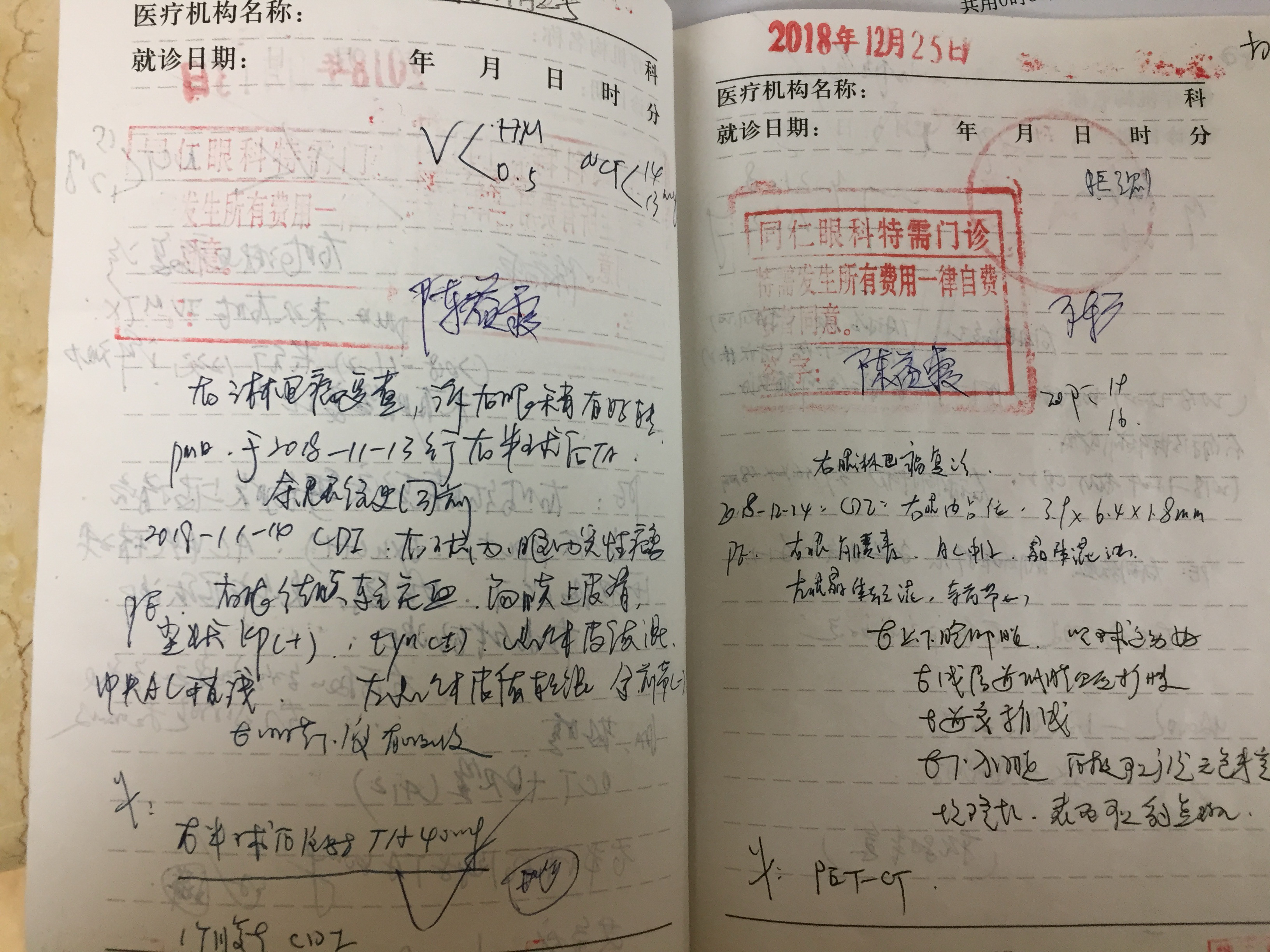

Supplement: Supplementary file 1 — Additional file 1: The raw data of this study. Table 1. The basic information of involved patients. [file 12886_2022_2598_MOESM1_ESM.zip › 3/IMG_6710.JPG]

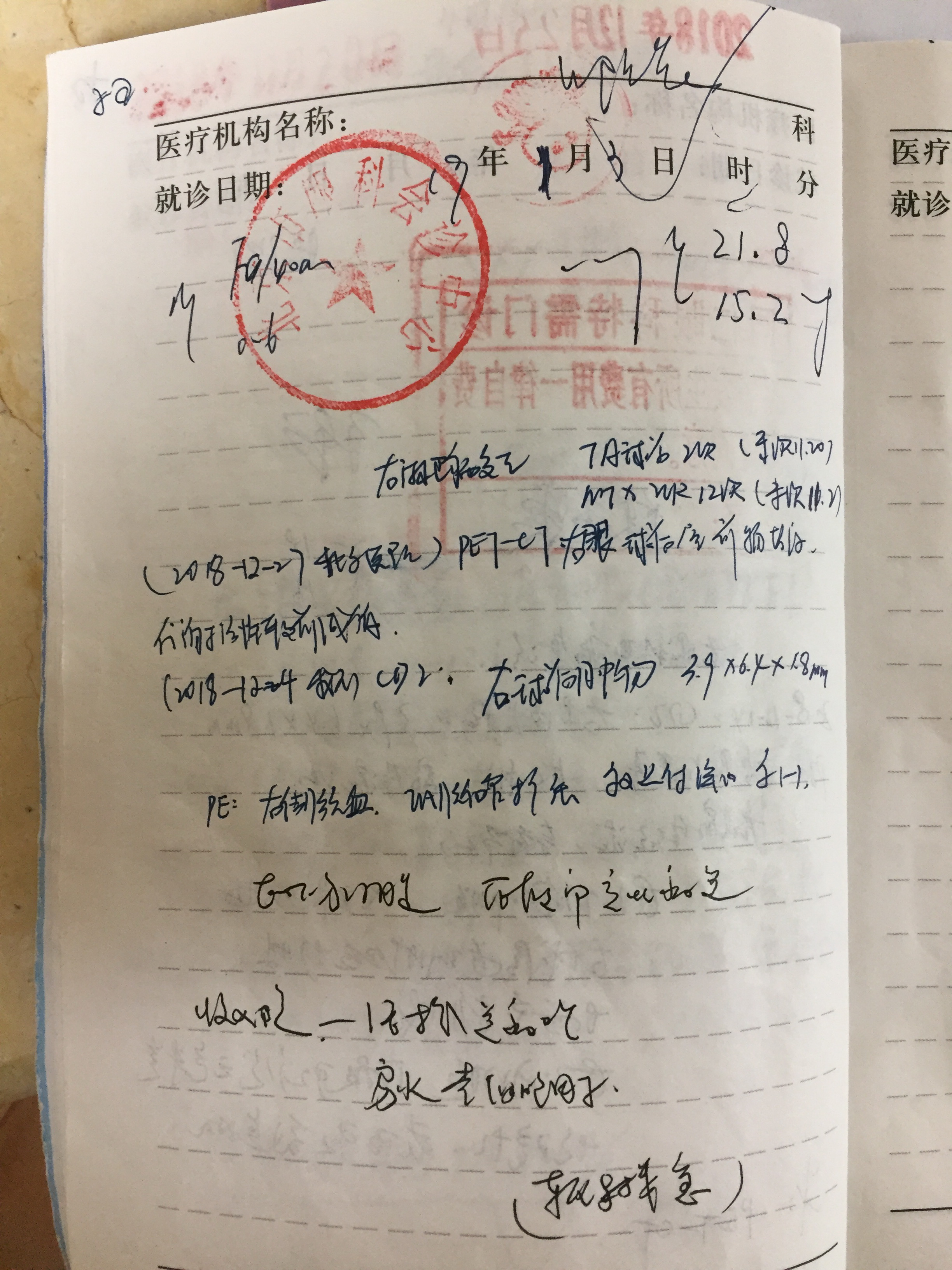

Supplement: Supplementary file 1 — Additional file 1: The raw data of this study. Table 1. The basic information of involved patients. [file 12886_2022_2598_MOESM1_ESM.zip › 3/IMG_6711.JPG]

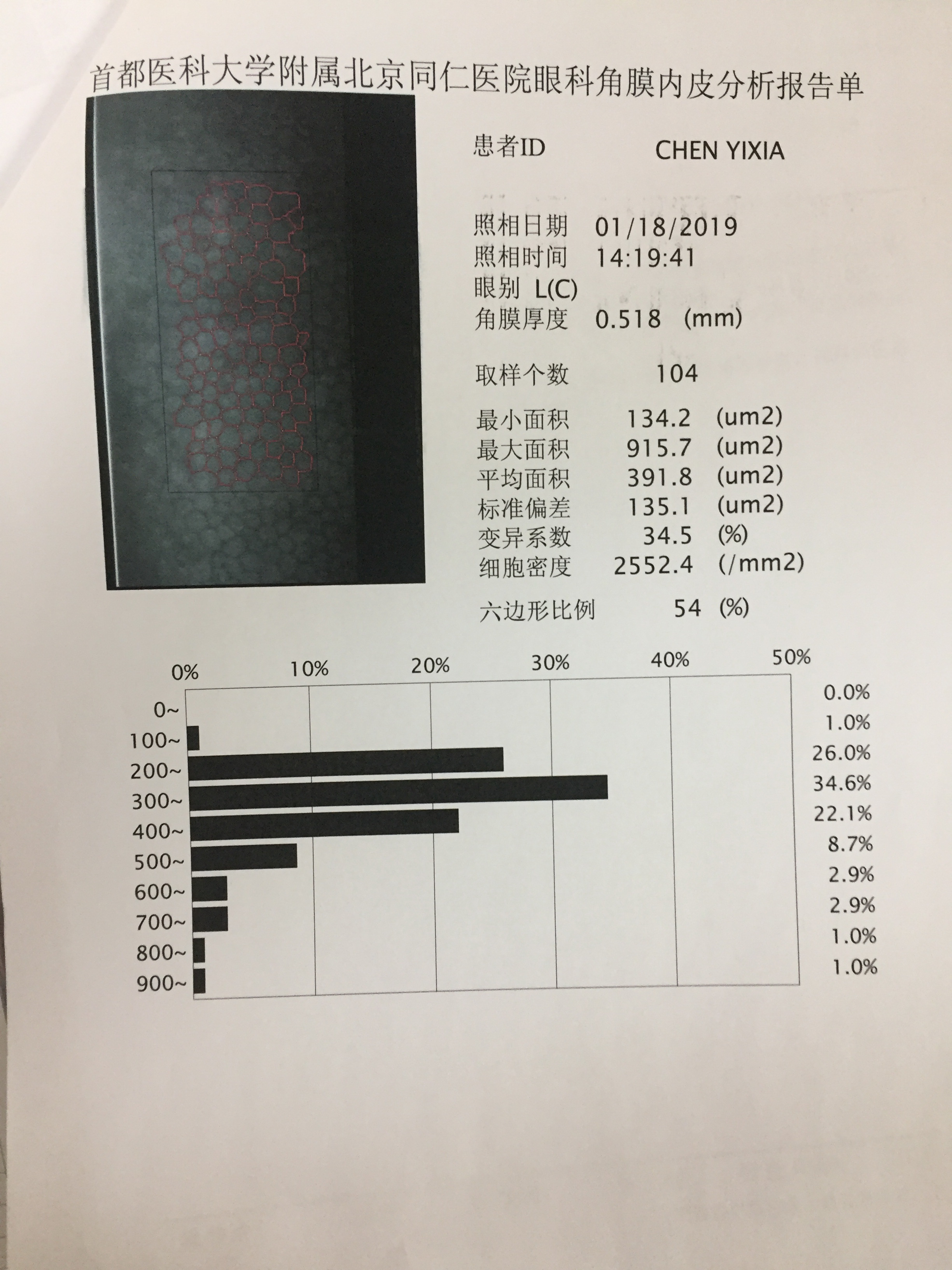

Supplement: Supplementary file 1 — Additional file 1: The raw data of this study. Table 1. The basic information of involved patients. [file 12886_2022_2598_MOESM1_ESM.zip › 3/IMG_6705.JPG]

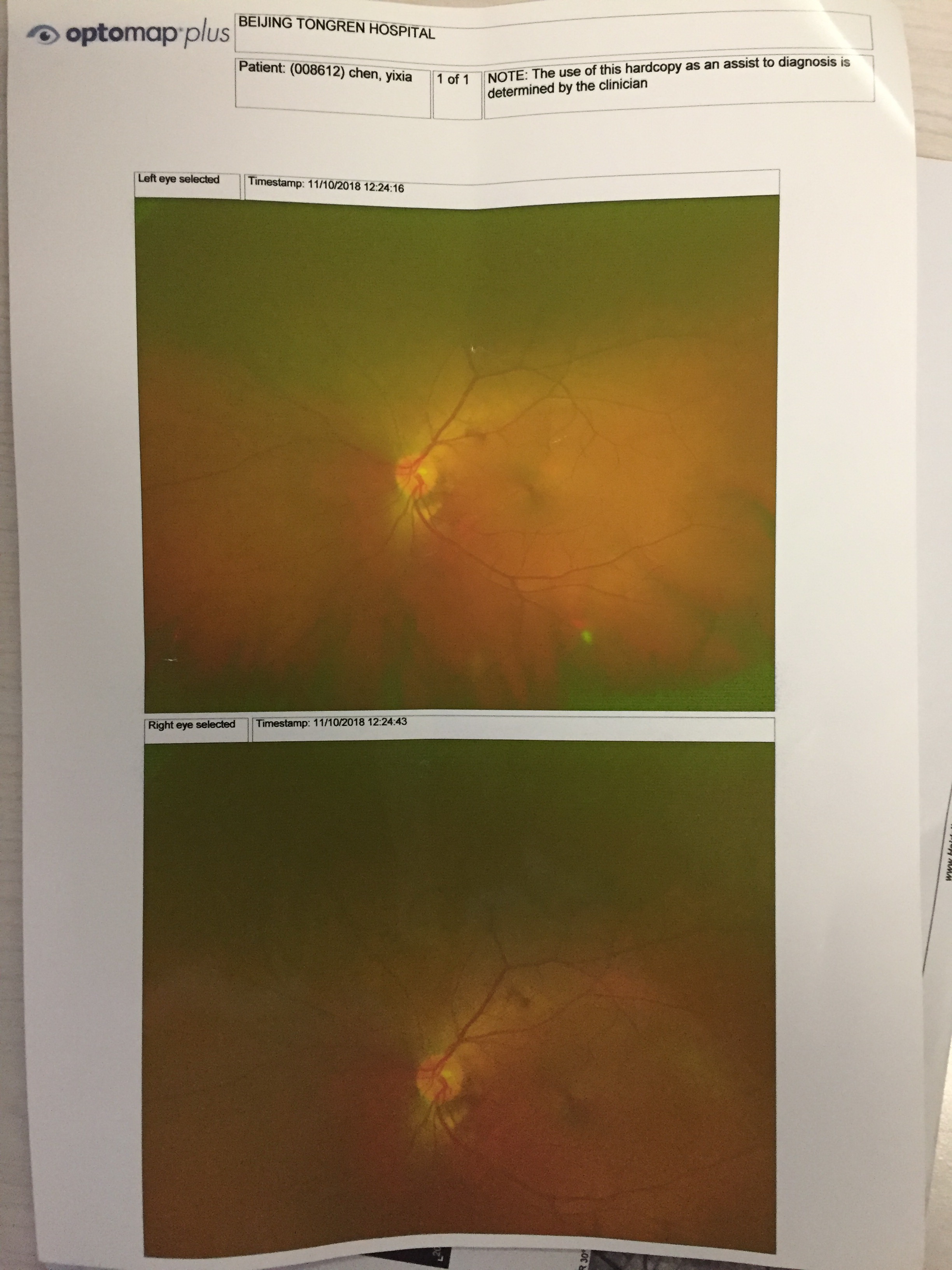

Supplement: Supplementary file 1 — Additional file 1: The raw data of this study. Table 1. The basic information of involved patients. [file 12886_2022_2598_MOESM1_ESM.zip › 3/20181011μ1⁄4oσáí.JPG]

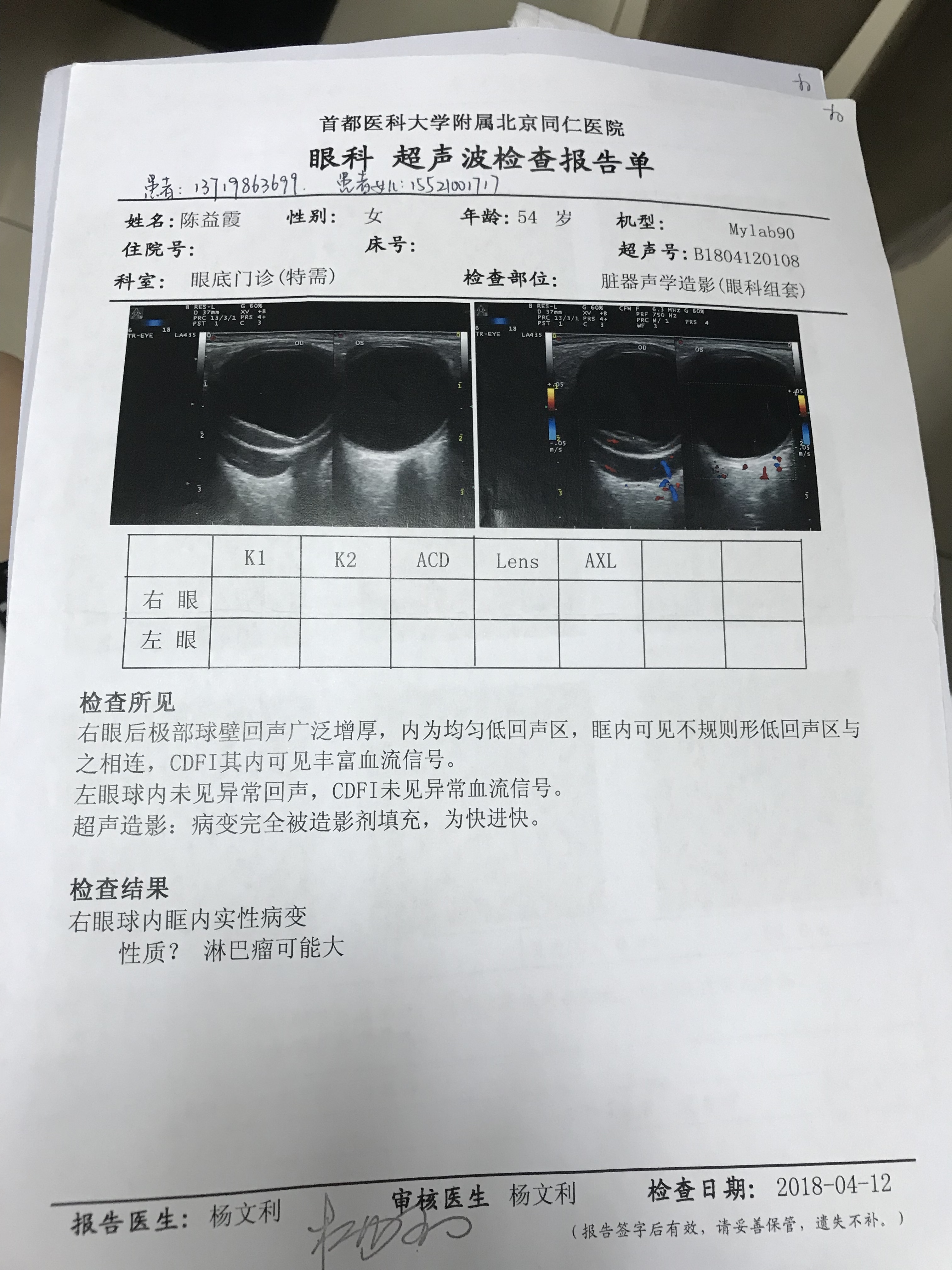

Supplement: Supplementary file 1 — Additional file 1: The raw data of this study. Table 1. The basic information of involved patients. [file 12886_2022_2598_MOESM1_ESM.zip › 3/IMG_4920.JPG]

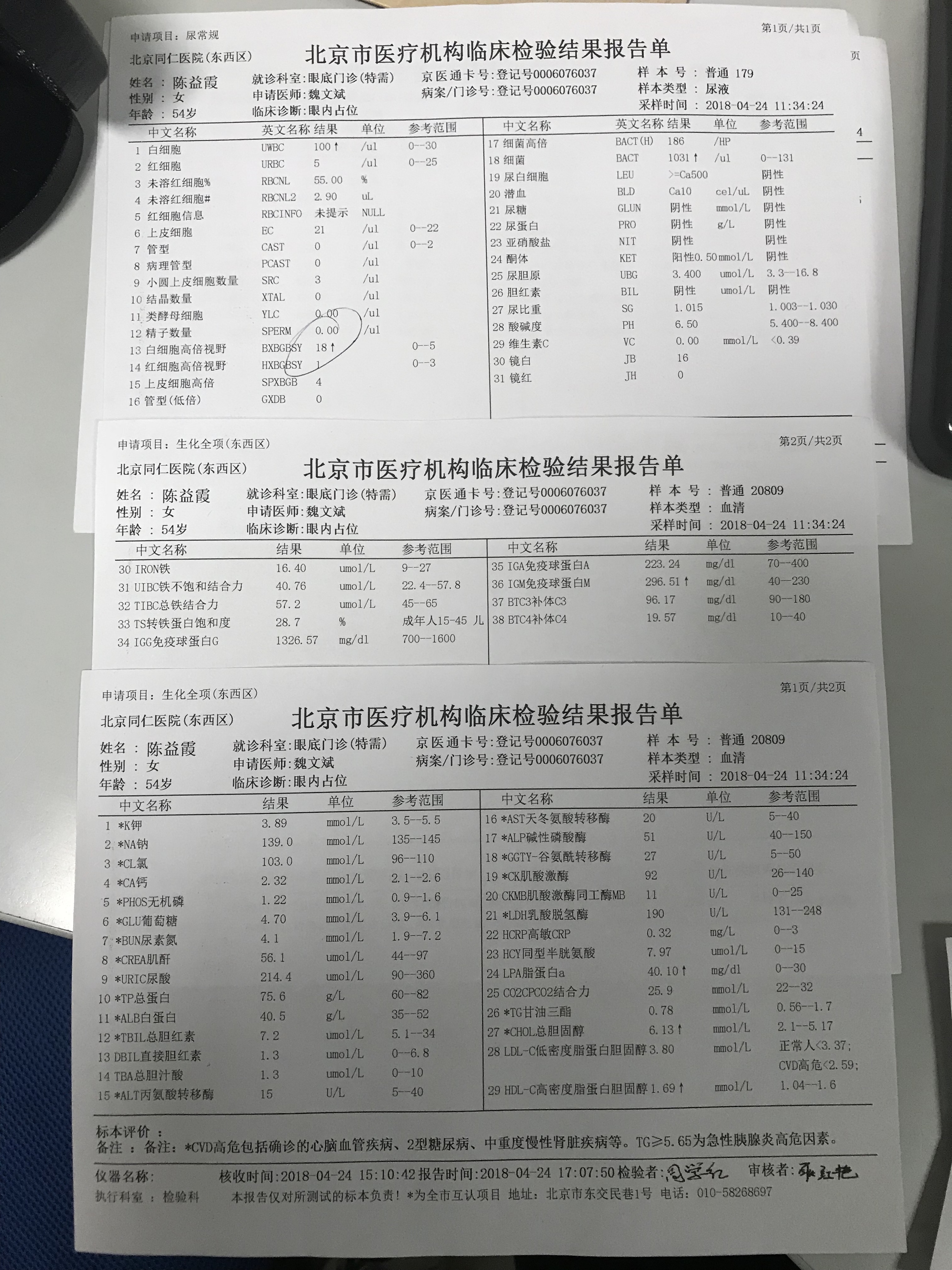

Supplement: Supplementary file 1 — Additional file 1: The raw data of this study. Table 1. The basic information of involved patients. [file 12886_2022_2598_MOESM1_ESM.zip › 3/IMG_4908.JPG]

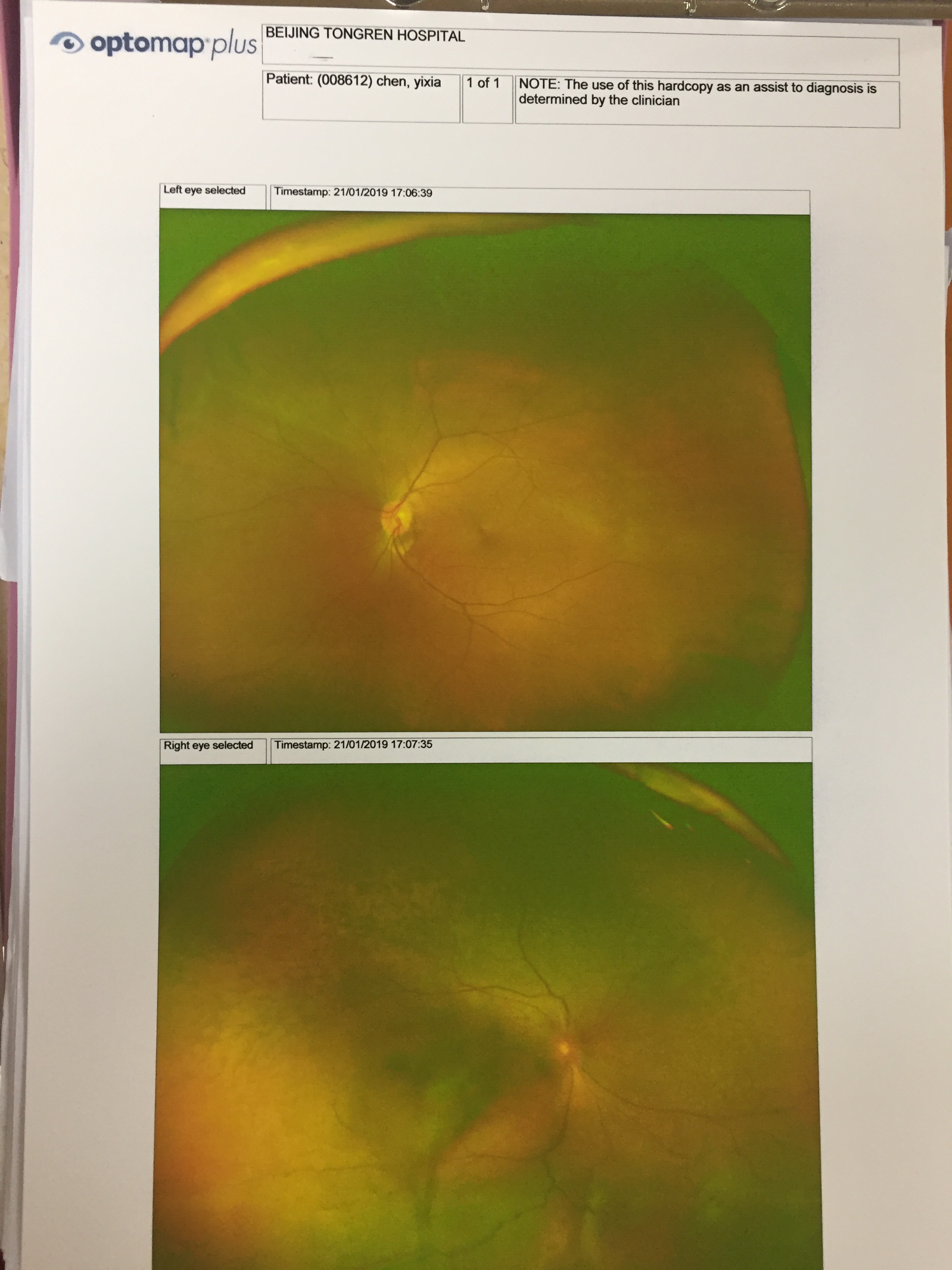

Supplement: Supplementary file 1 — Additional file 1: The raw data of this study. Table 1. The basic information of involved patients. [file 12886_2022_2598_MOESM1_ESM.zip › 3/IMG_6702.JPG]

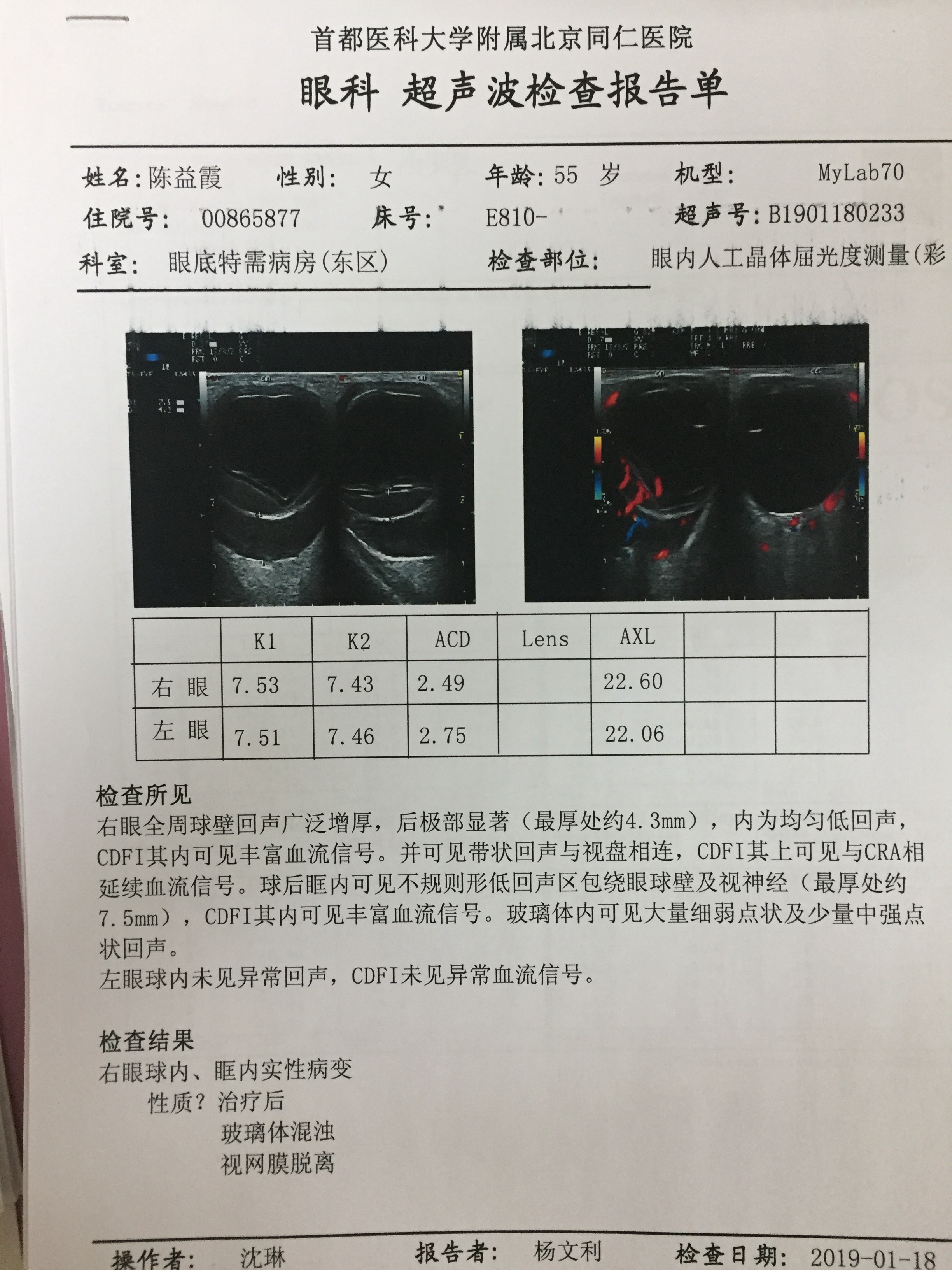

Supplement: Supplementary file 1 — Additional file 1: The raw data of this study. Table 1. The basic information of involved patients. [file 12886_2022_2598_MOESM1_ESM.zip › 3/IMG_6703.JPG]

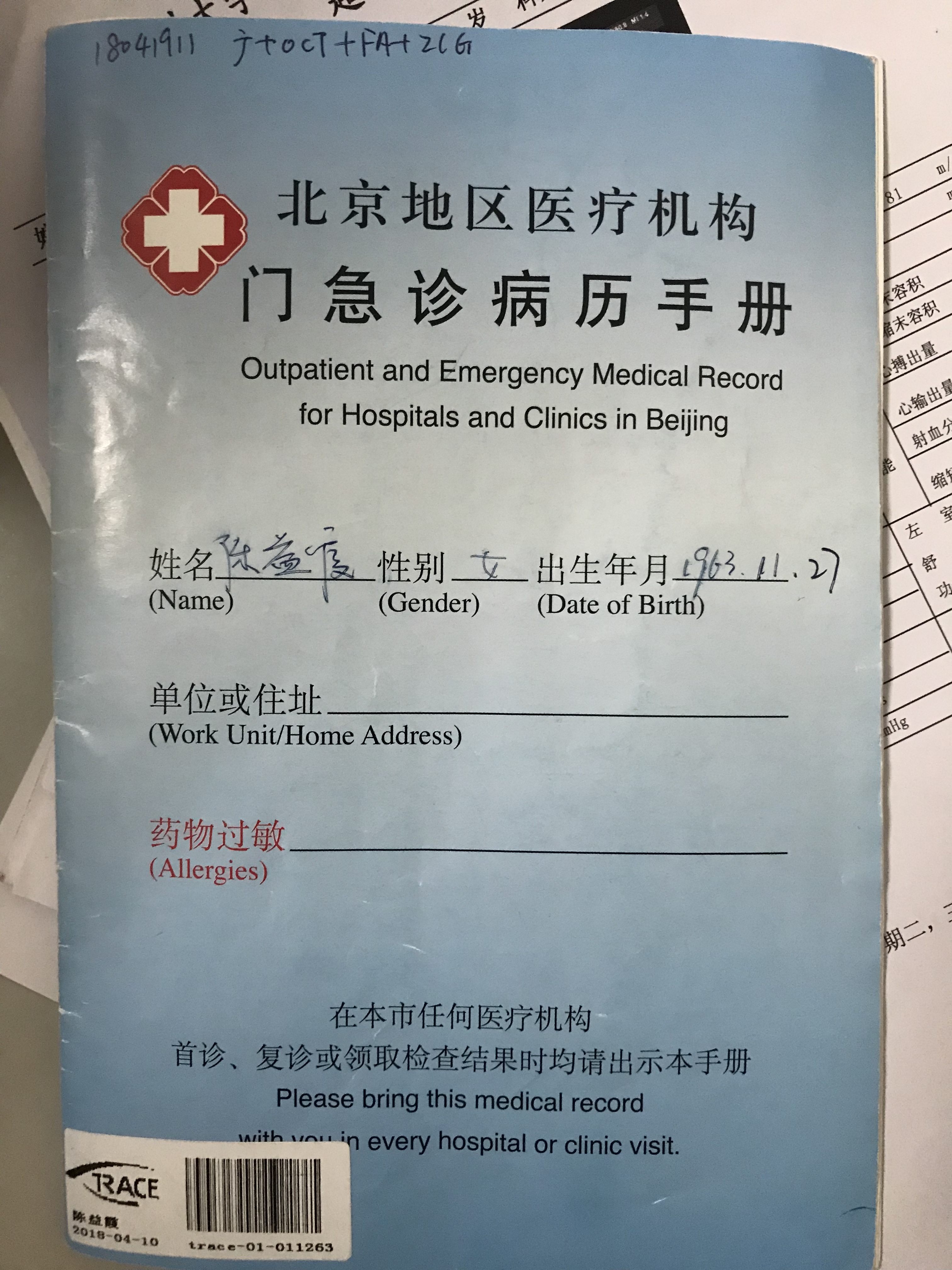

Supplement: Supplementary file 1 — Additional file 1: The raw data of this study. Table 1. The basic information of involved patients. [file 12886_2022_2598_MOESM1_ESM.zip › 3/IMG_4909.JPG]

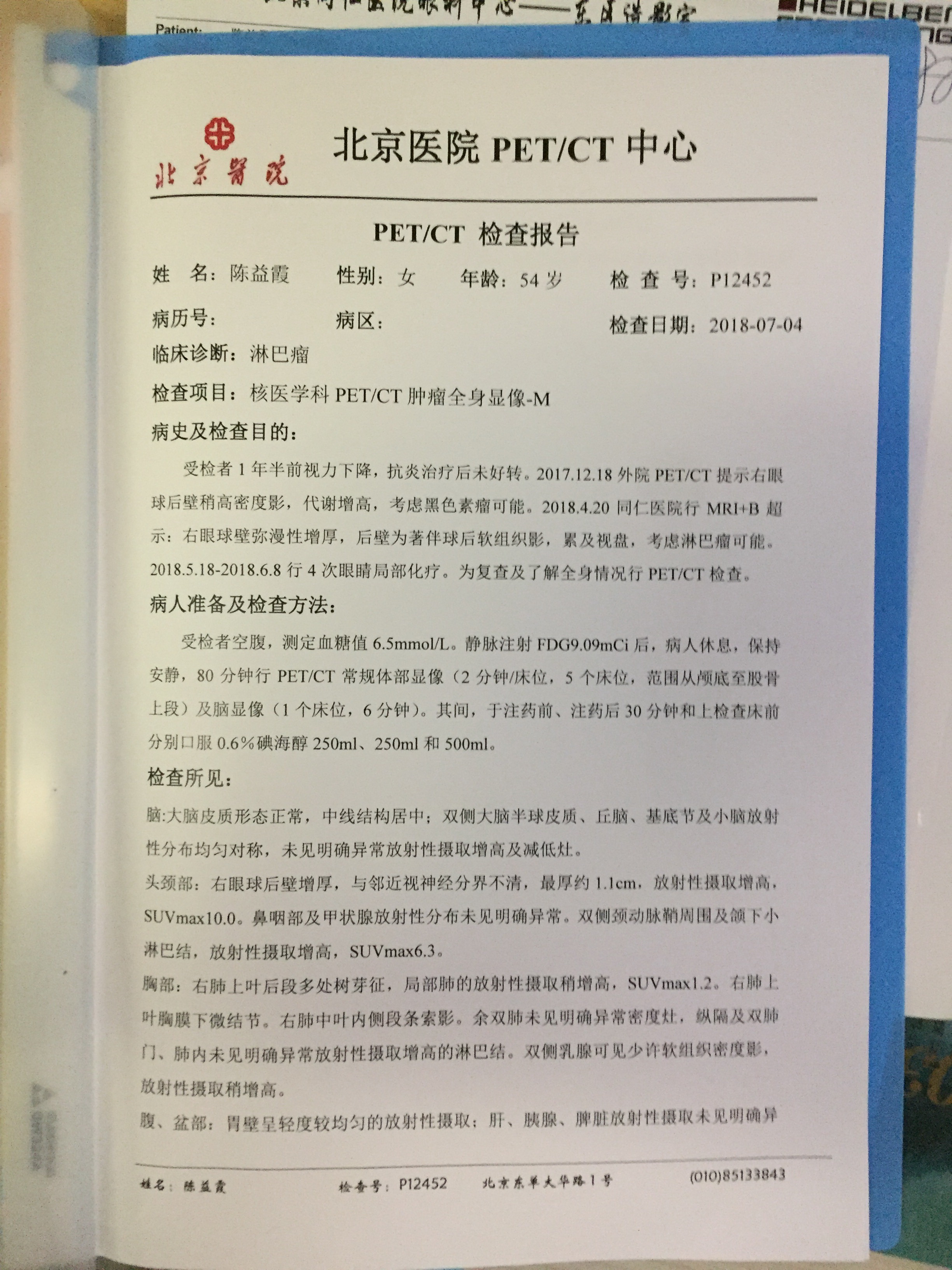

Supplement: Supplementary file 1 — Additional file 1: The raw data of this study. Table 1. The basic information of involved patients. [file 12886_2022_2598_MOESM1_ESM.zip › 3/IMG_7076.JPG]

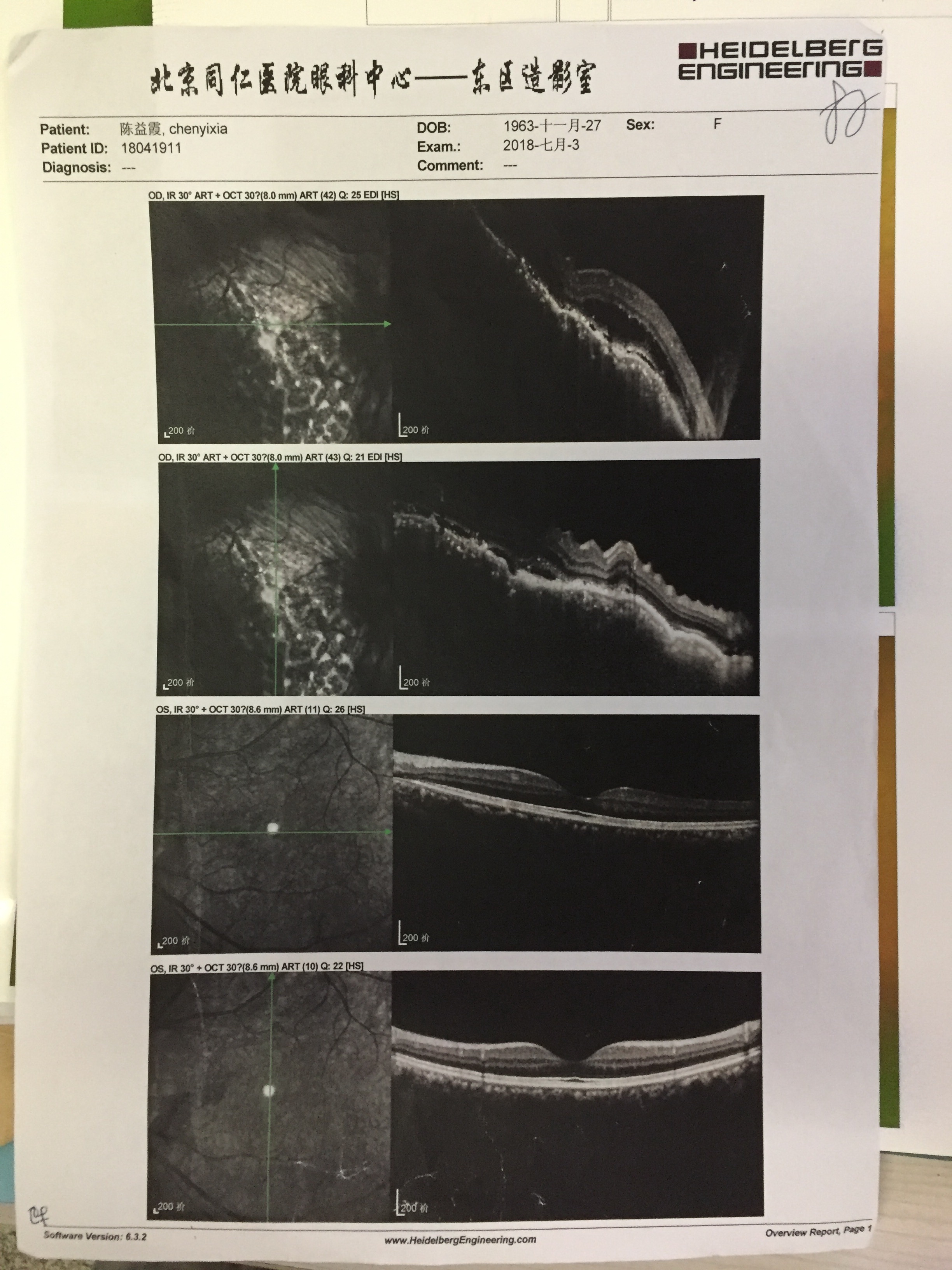

Supplement: Supplementary file 1 — Additional file 1: The raw data of this study. Table 1. The basic information of involved patients. [file 12886_2022_2598_MOESM1_ESM.zip › 3/IMG_7074.JPG]

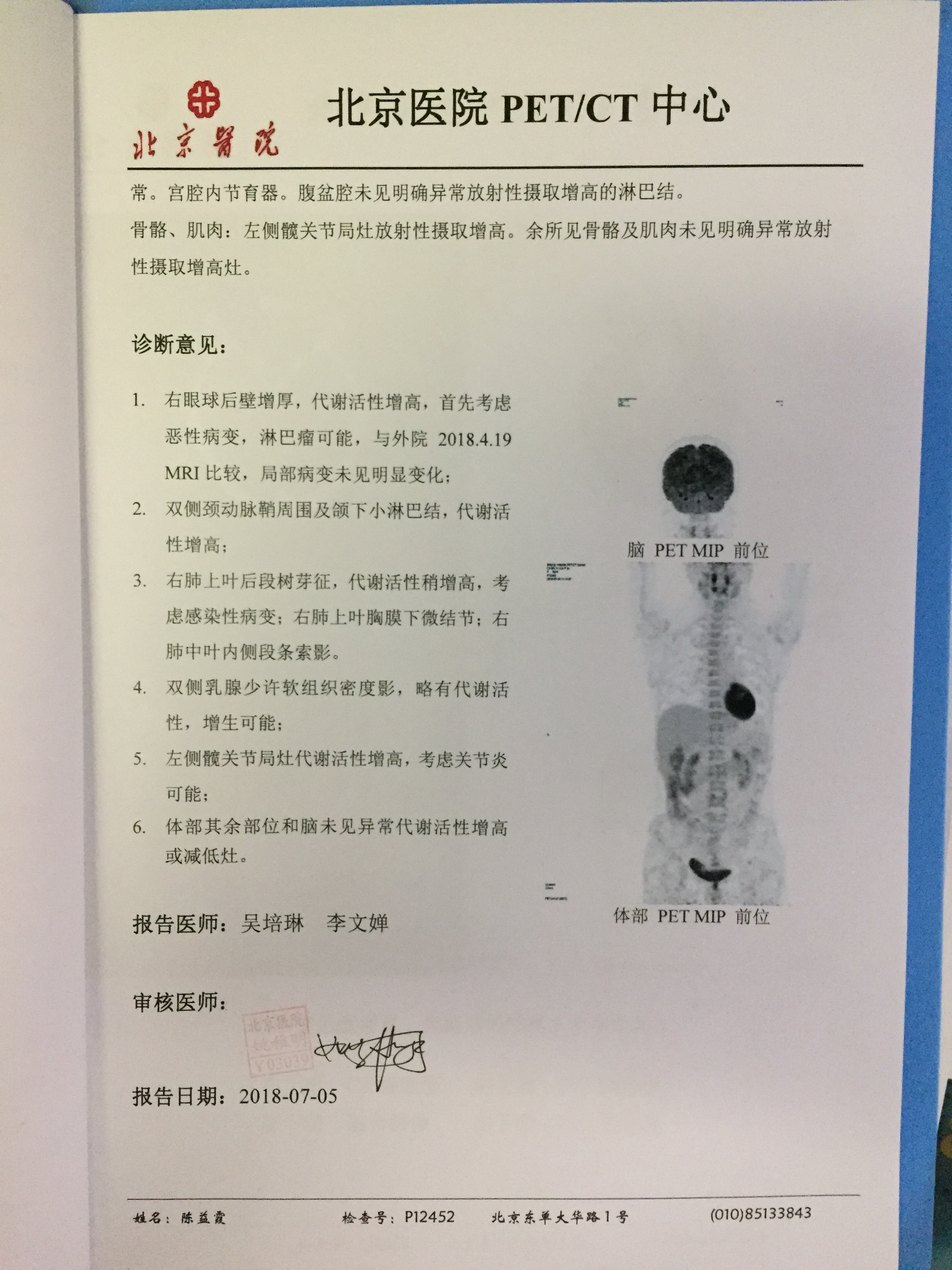

Supplement: Supplementary file 1 — Additional file 1: The raw data of this study. Table 1. The basic information of involved patients. [file 12886_2022_2598_MOESM1_ESM.zip › 3/IMG_7075.JPG]

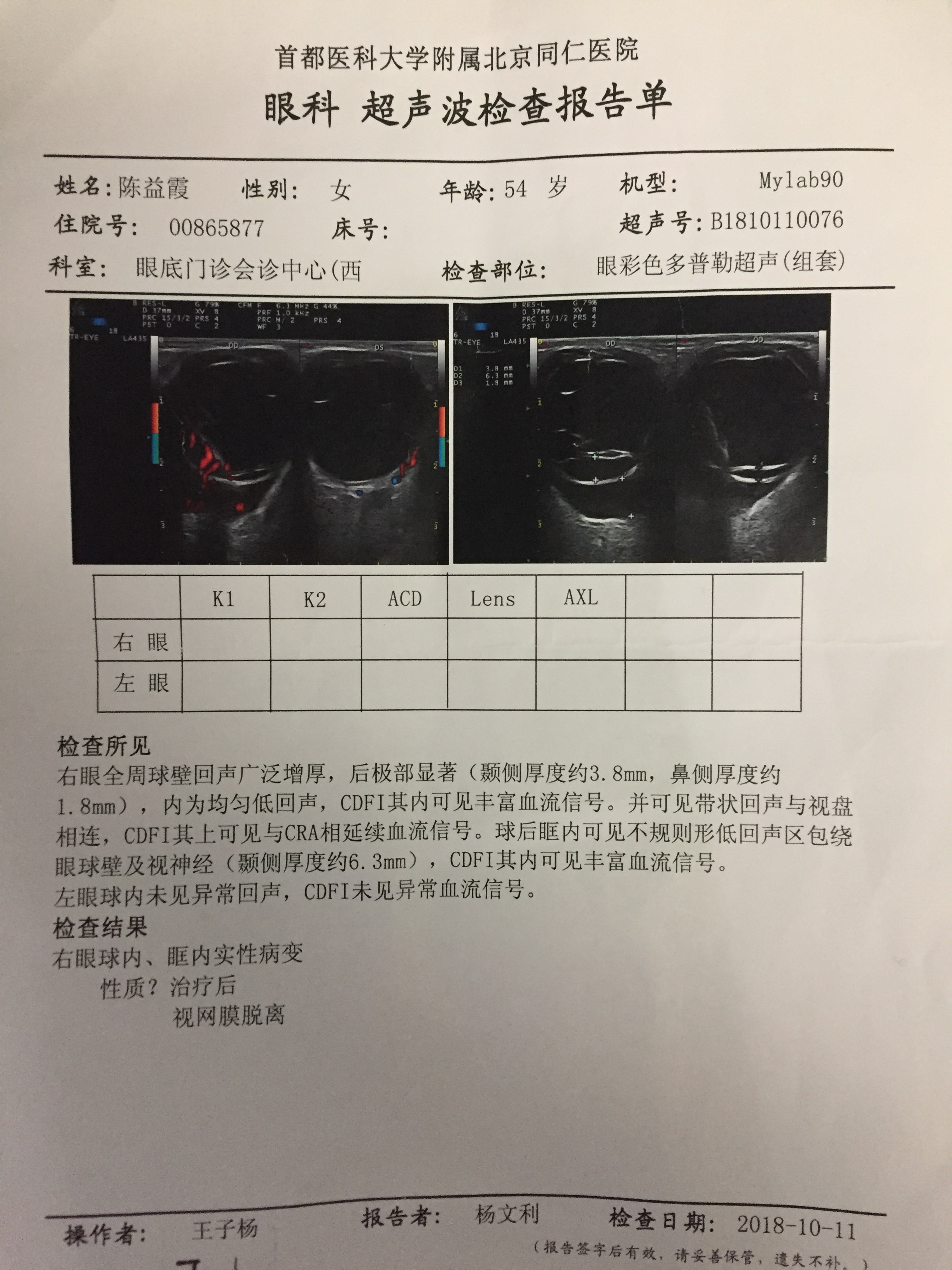

Supplement: Supplementary file 1 — Additional file 1: The raw data of this study. Table 1. The basic information of involved patients. [file 12886_2022_2598_MOESM1_ESM.zip › 3/20181011CDI.JPG]

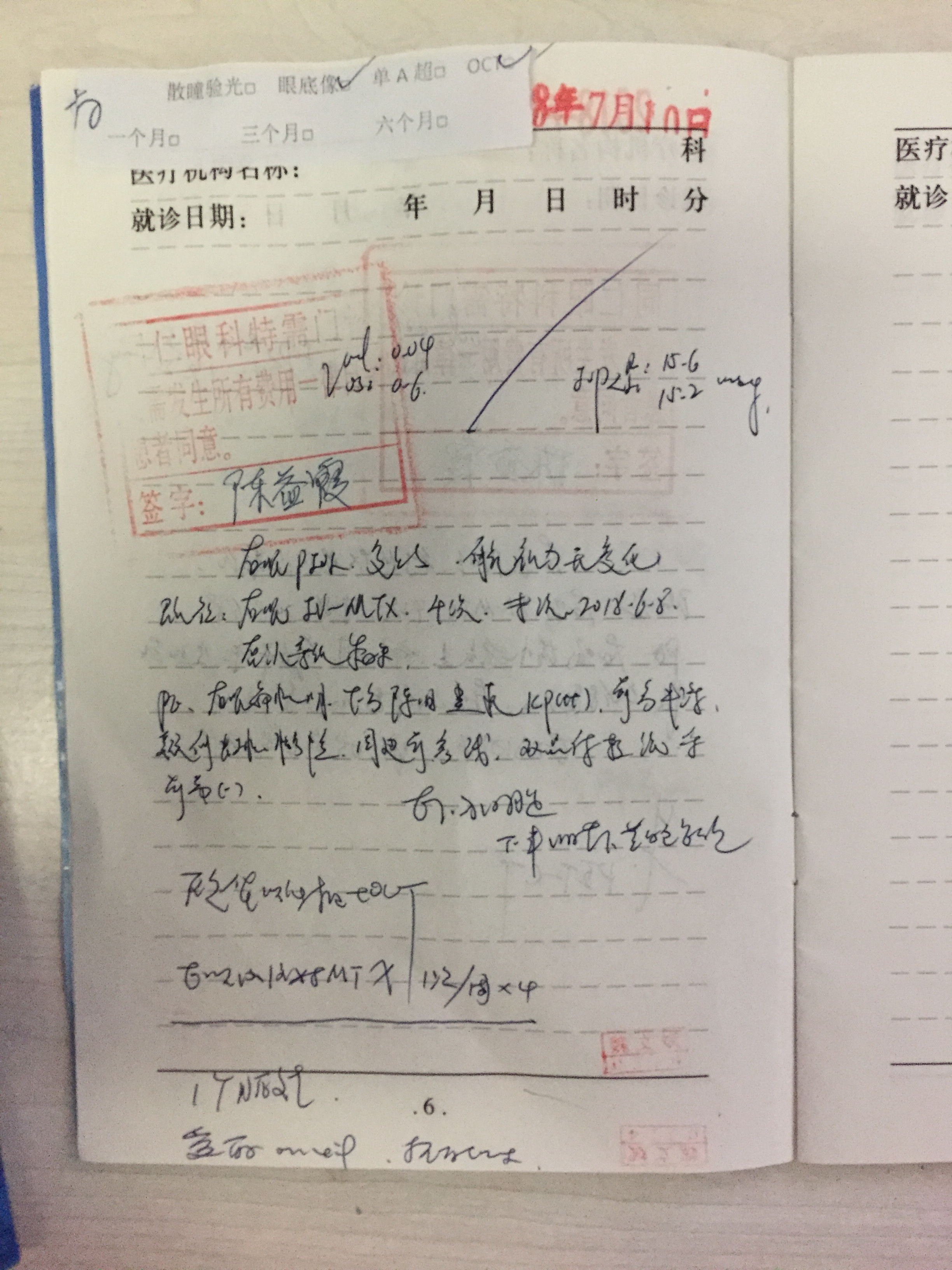

Supplement: Supplementary file 1 — Additional file 1: The raw data of this study. Table 1. The basic information of involved patients. [file 12886_2022_2598_MOESM1_ESM.zip › 3/IMG_7072.JPG]

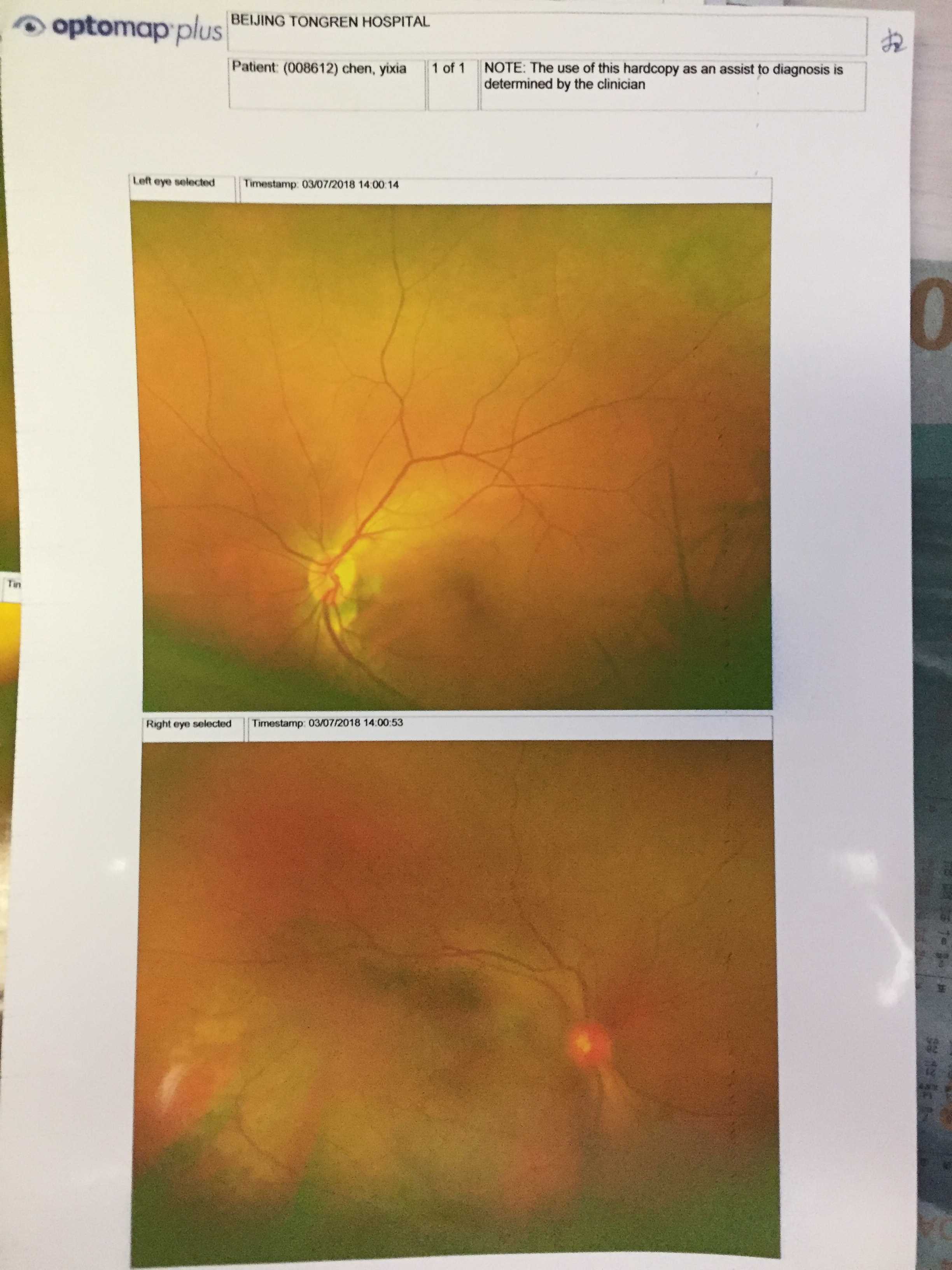

Supplement: Supplementary file 1 — Additional file 1: The raw data of this study. Table 1. The basic information of involved patients. [file 12886_2022_2598_MOESM1_ESM.zip › 3/IMG_7073.JPG]

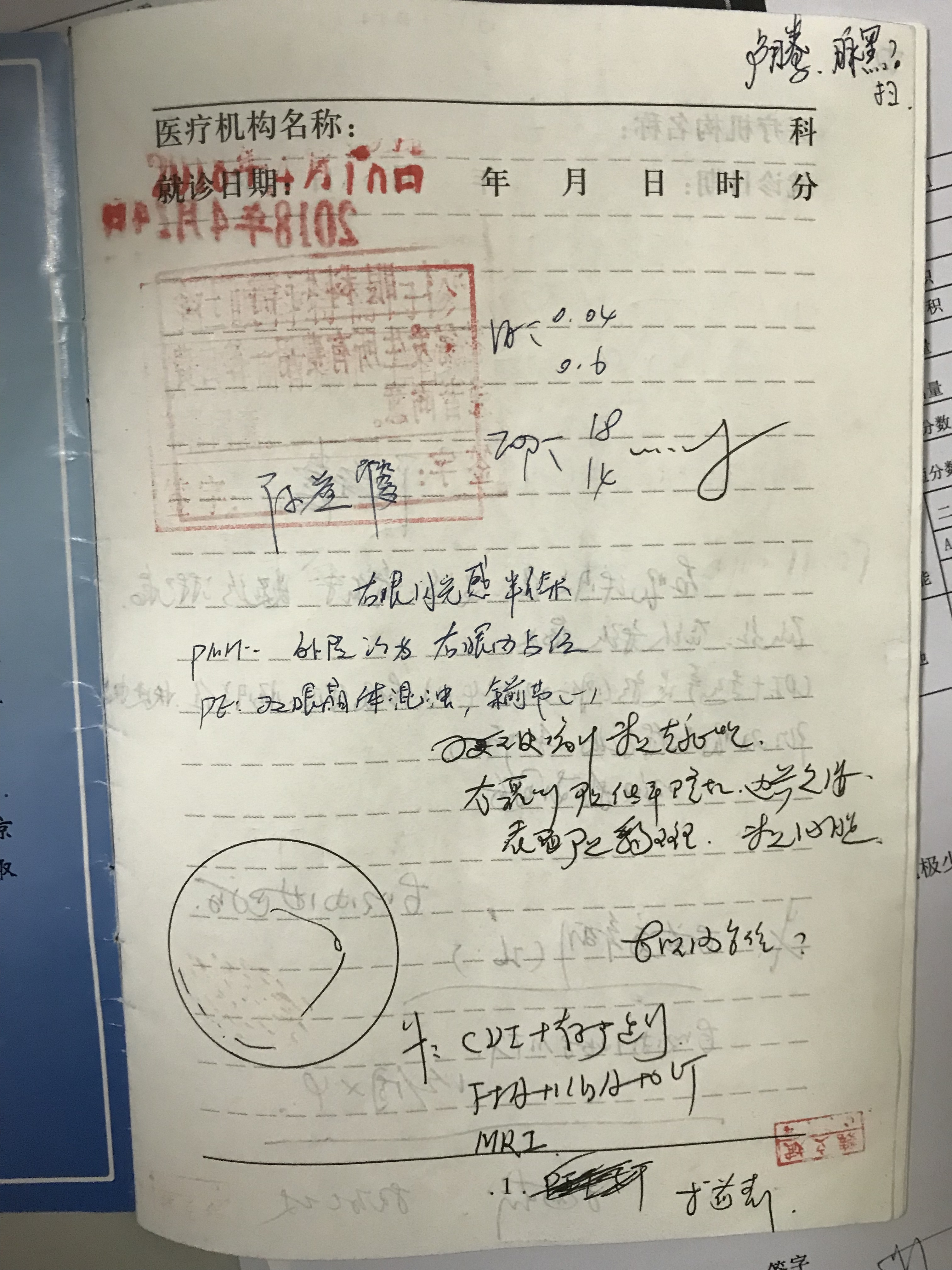

Supplement: Supplementary file 1 — Additional file 1: The raw data of this study. Table 1. The basic information of involved patients. [file 12886_2022_2598_MOESM1_ESM.zip › 3/IMG_4910.JPG]

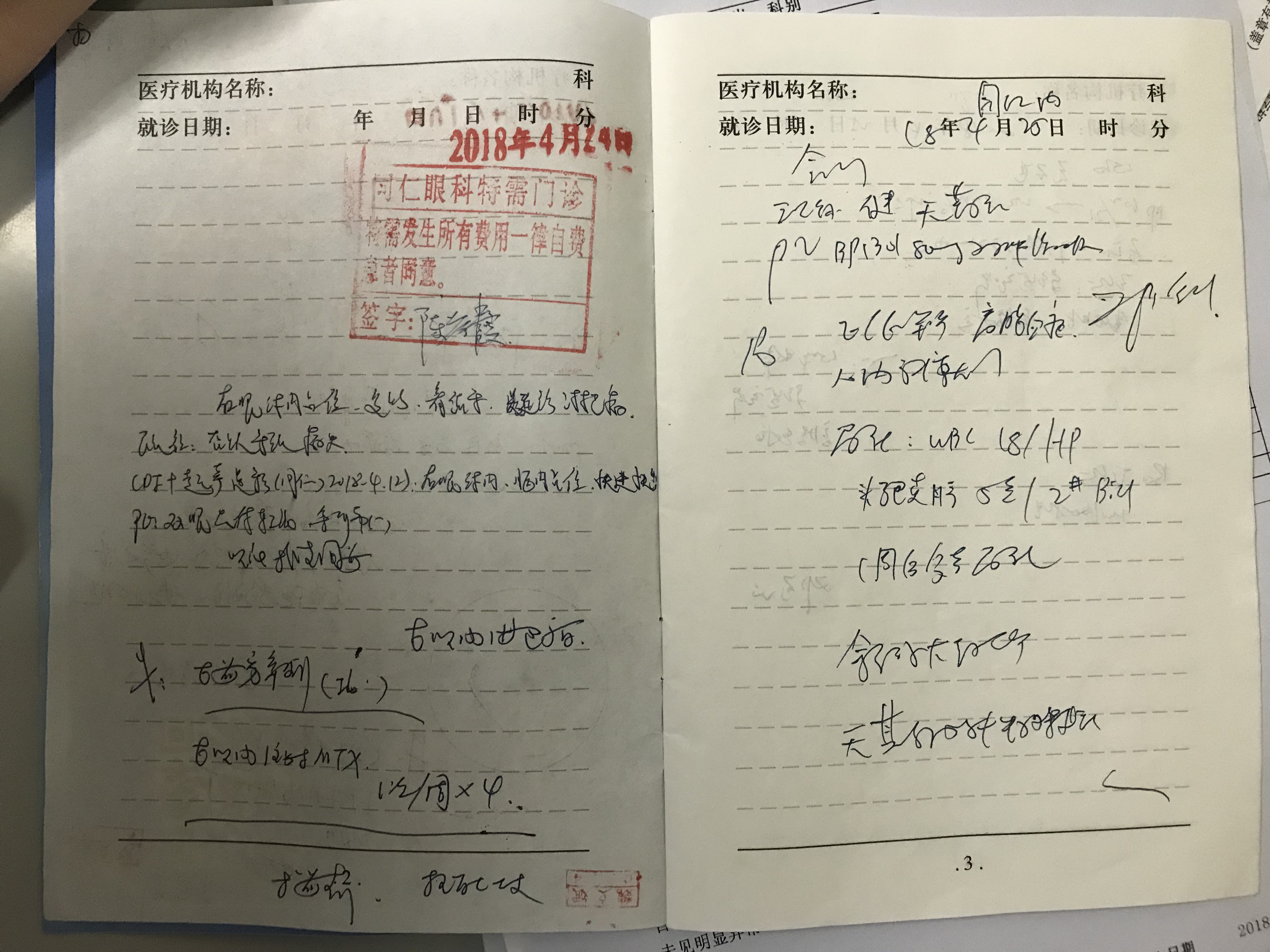

Supplement: Supplementary file 1 — Additional file 1: The raw data of this study. Table 1. The basic information of involved patients. [file 12886_2022_2598_MOESM1_ESM.zip › 3/IMG_4911.JPG]

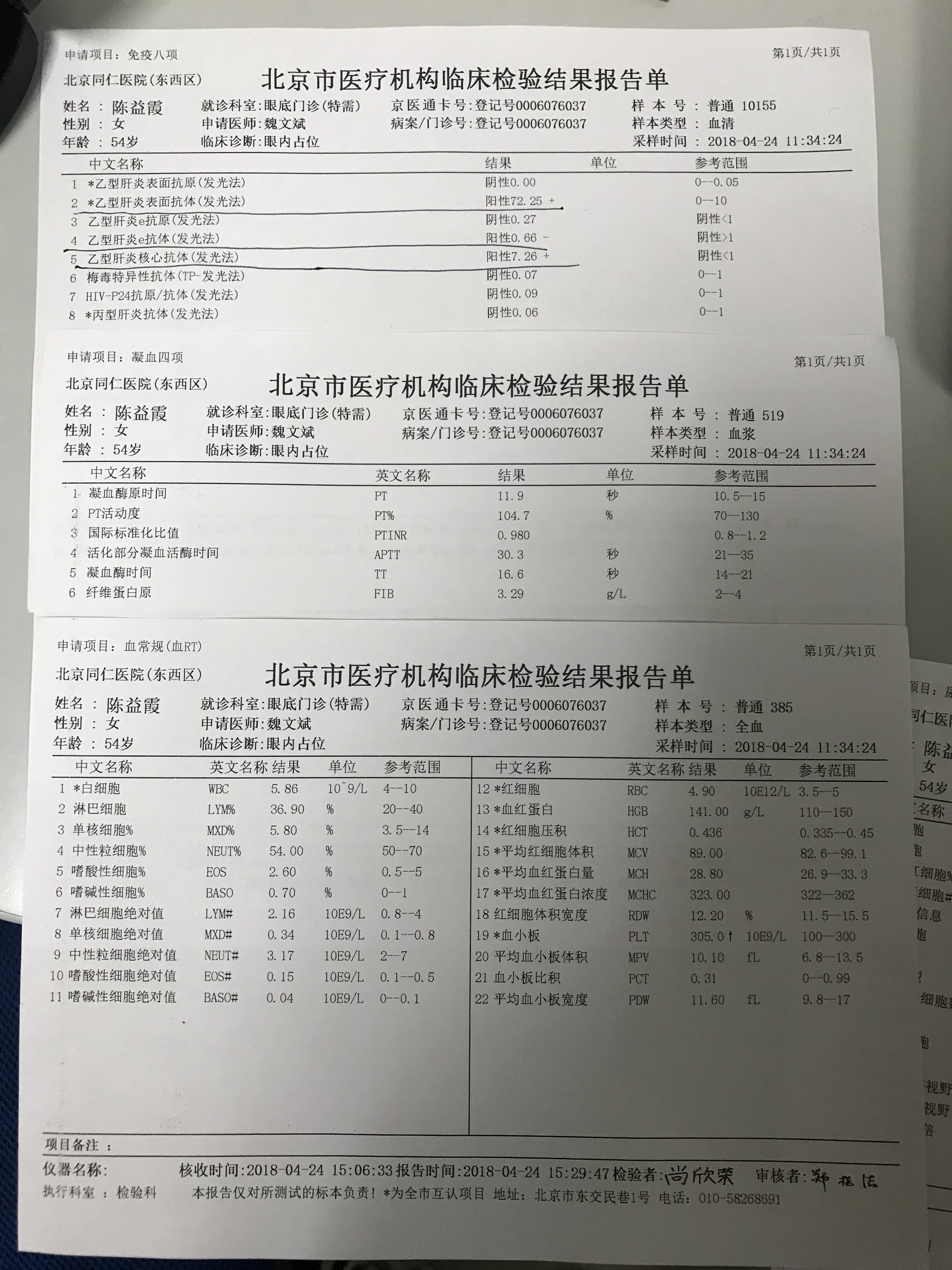

Supplement: Supplementary file 1 — Additional file 1: The raw data of this study. Table 1. The basic information of involved patients. [file 12886_2022_2598_MOESM1_ESM.zip › 3/IMG_4907.JPG]

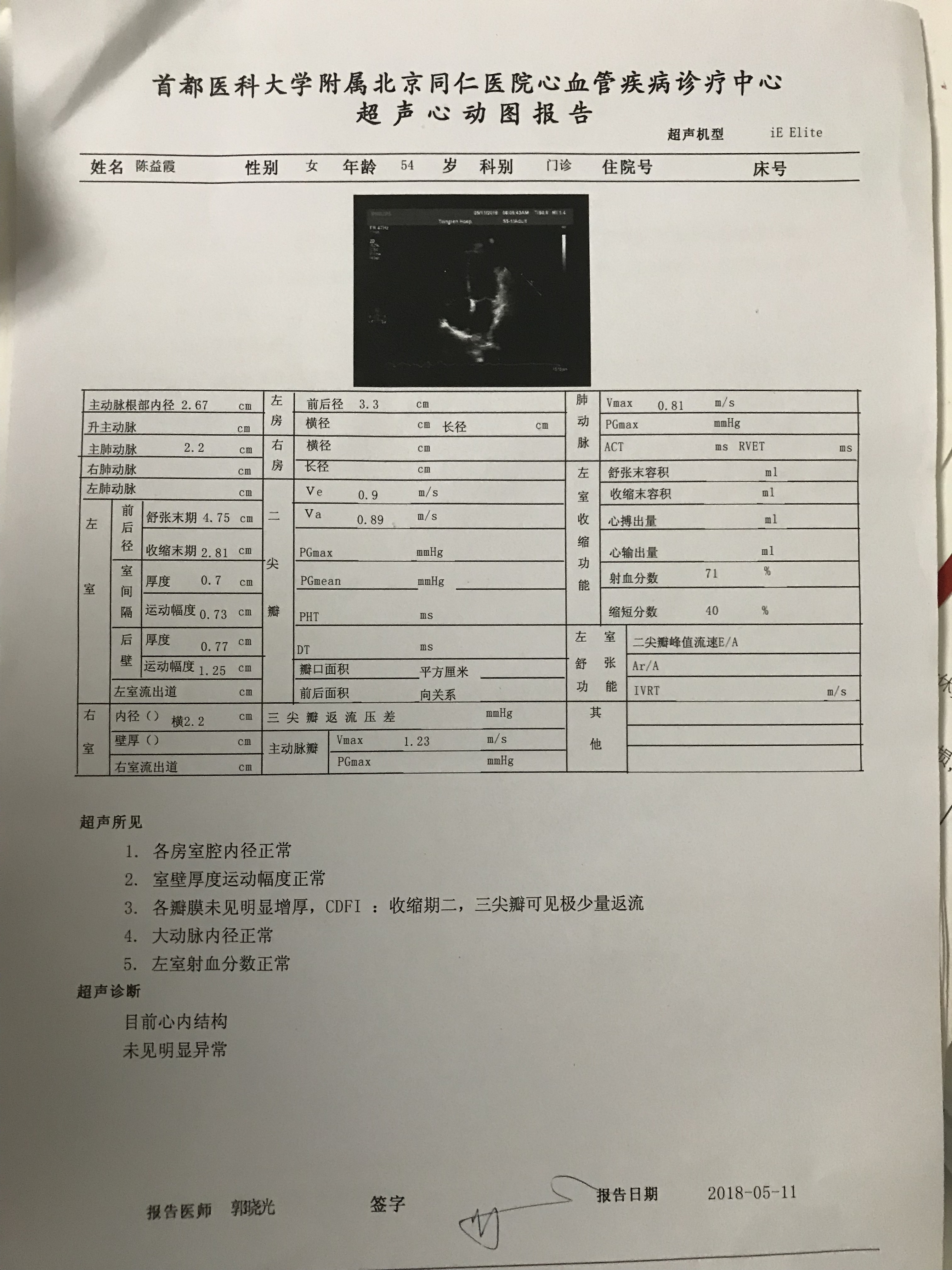

Supplement: Supplementary file 1 — Additional file 1: The raw data of this study. Table 1. The basic information of involved patients. [file 12886_2022_2598_MOESM1_ESM.zip › 3/IMG_4913.JPG]

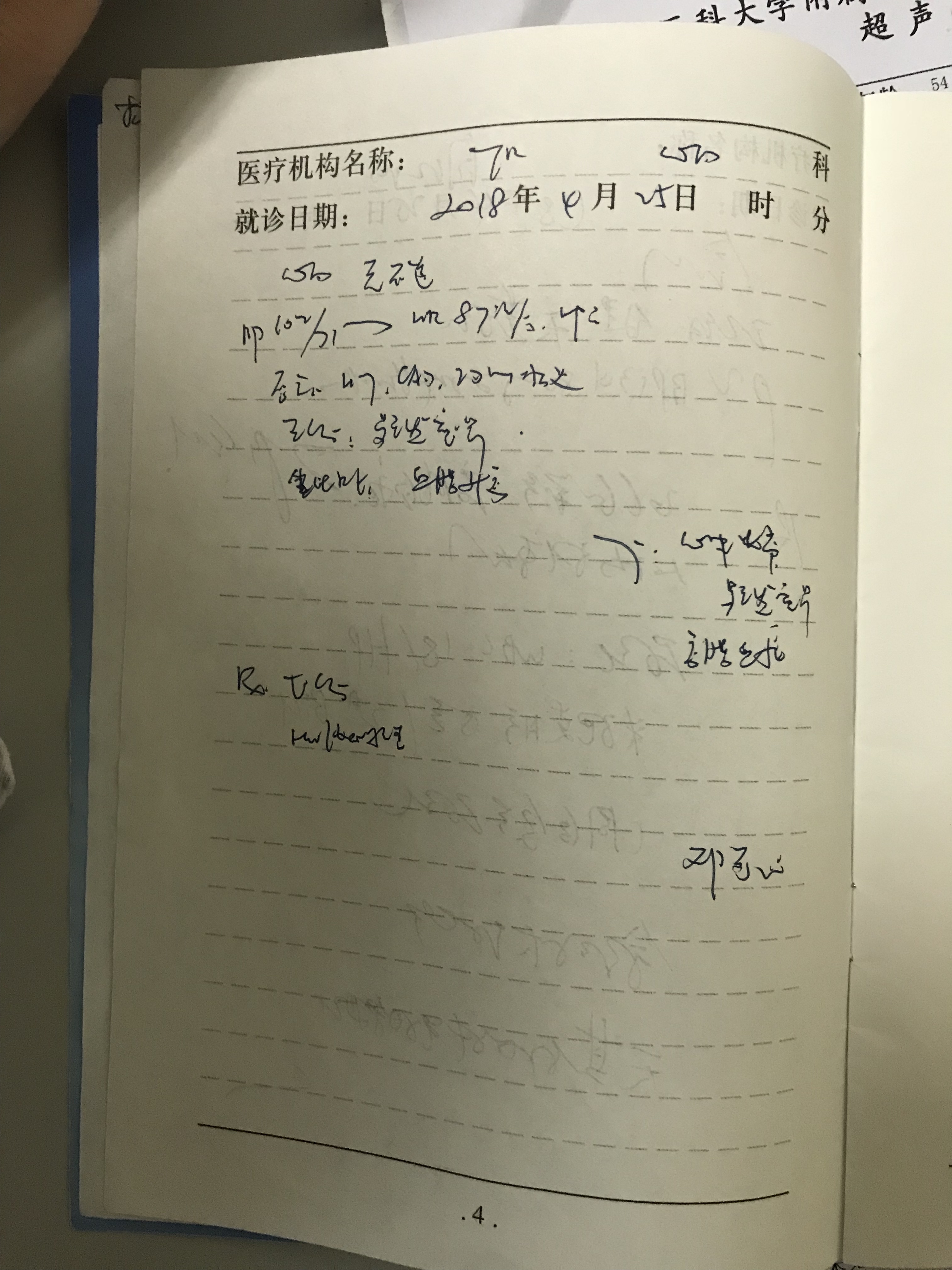

Supplement: Supplementary file 1 — Additional file 1: The raw data of this study. Table 1. The basic information of involved patients. [file 12886_2022_2598_MOESM1_ESM.zip › 3/IMG_4912.JPG]

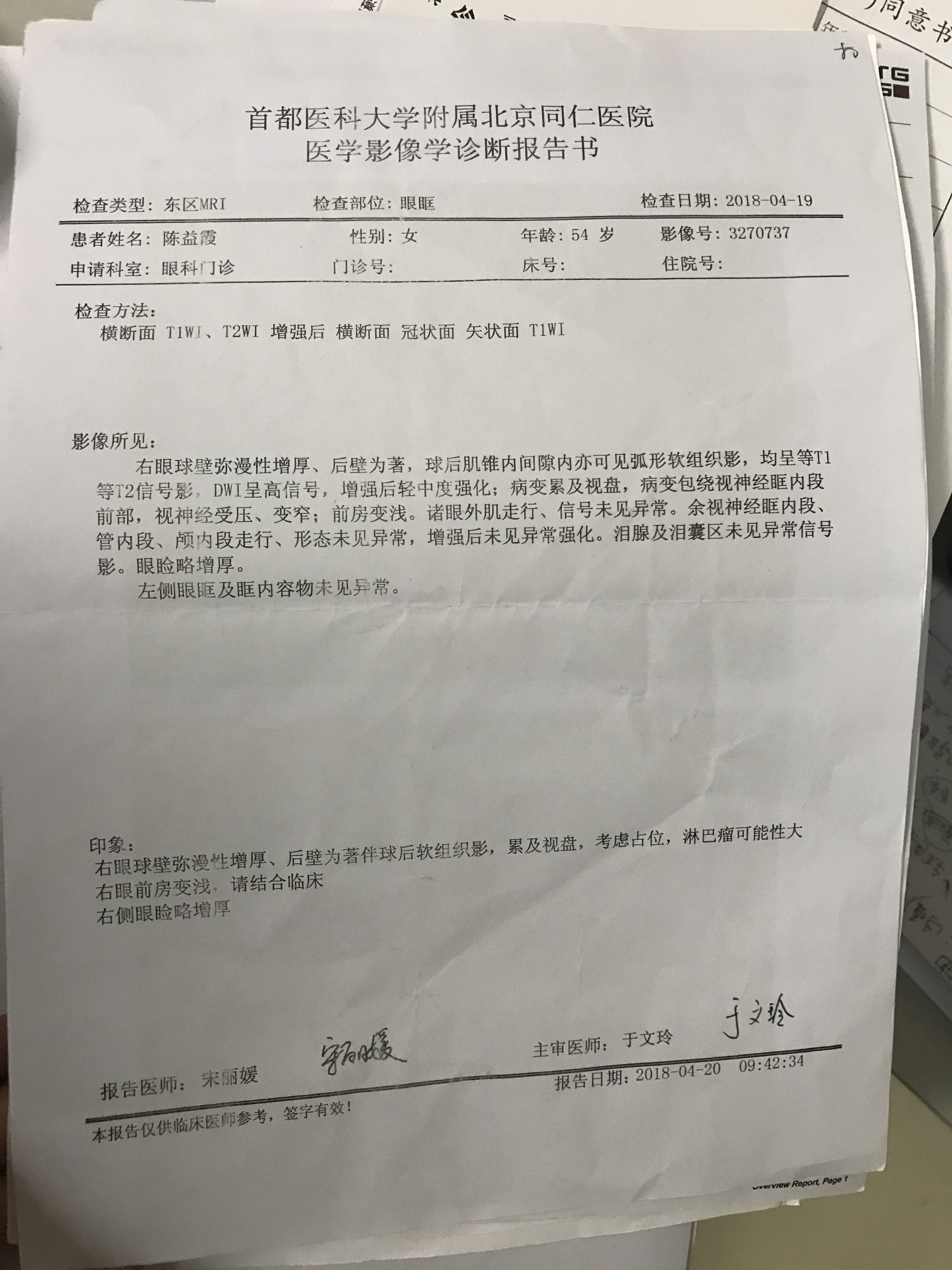

Supplement: Supplementary file 1 — Additional file 1: The raw data of this study. Table 1. The basic information of involved patients. [file 12886_2022_2598_MOESM1_ESM.zip › 3/IMG_4916.JPG]

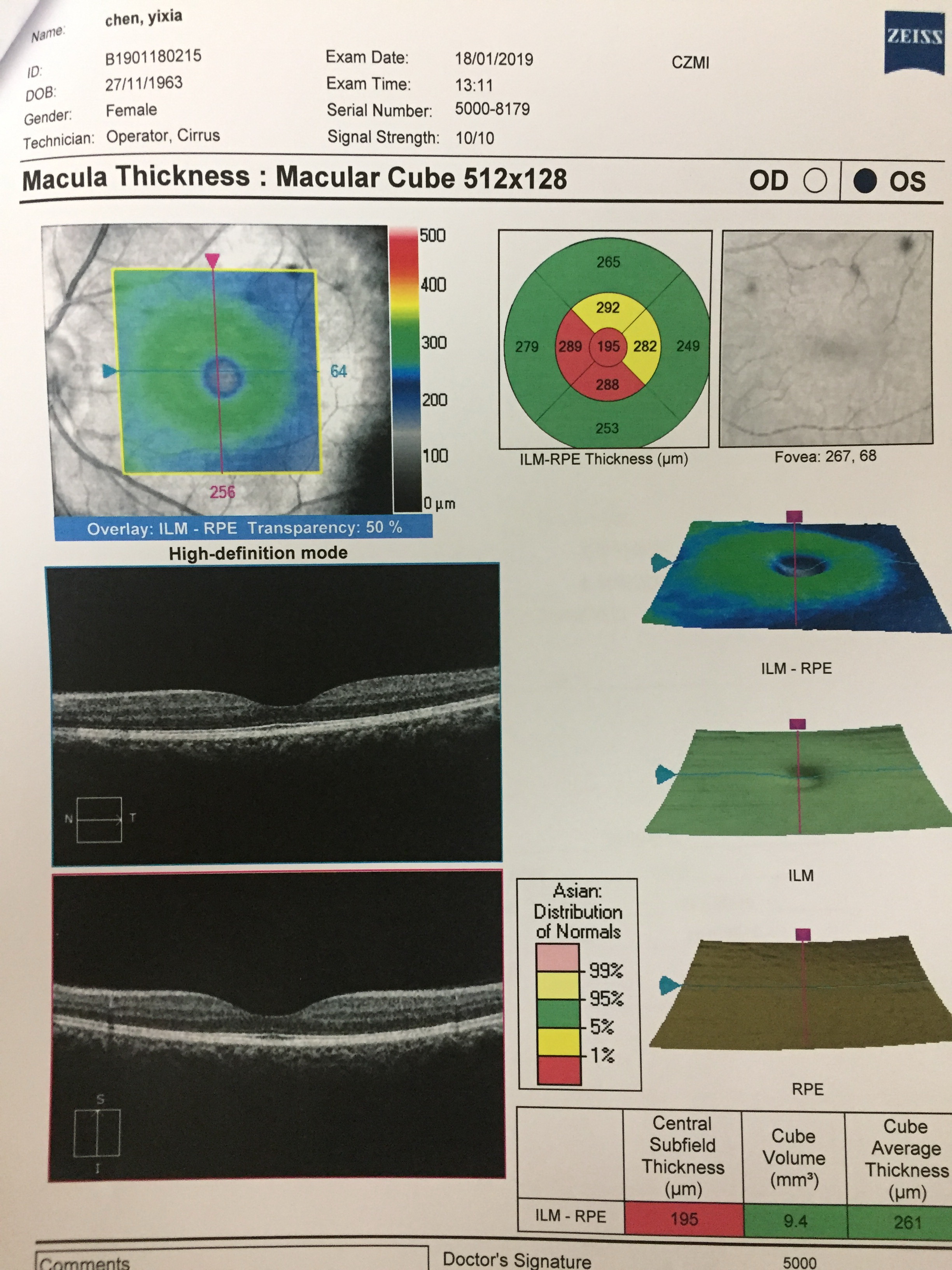

Supplement: Supplementary file 1 — Additional file 1: The raw data of this study. Table 1. The basic information of involved patients. [file 12886_2022_2598_MOESM1_ESM.zip › 3/IMG_6708.JPG]

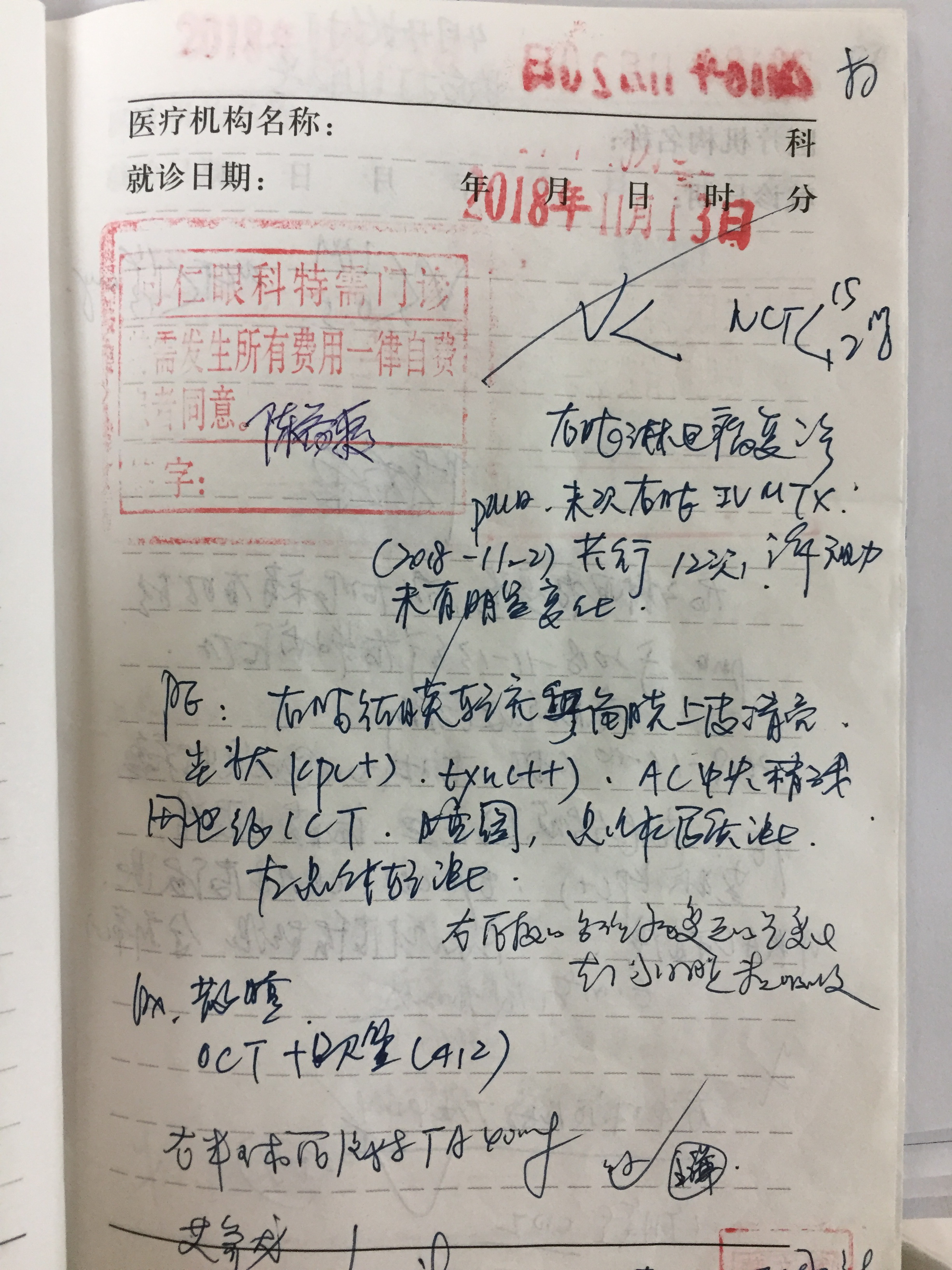

Supplement: Supplementary file 1 — Additional file 1: The raw data of this study. Table 1. The basic information of involved patients. [file 12886_2022_2598_MOESM1_ESM.zip › 3/IMG_6709.JPG]

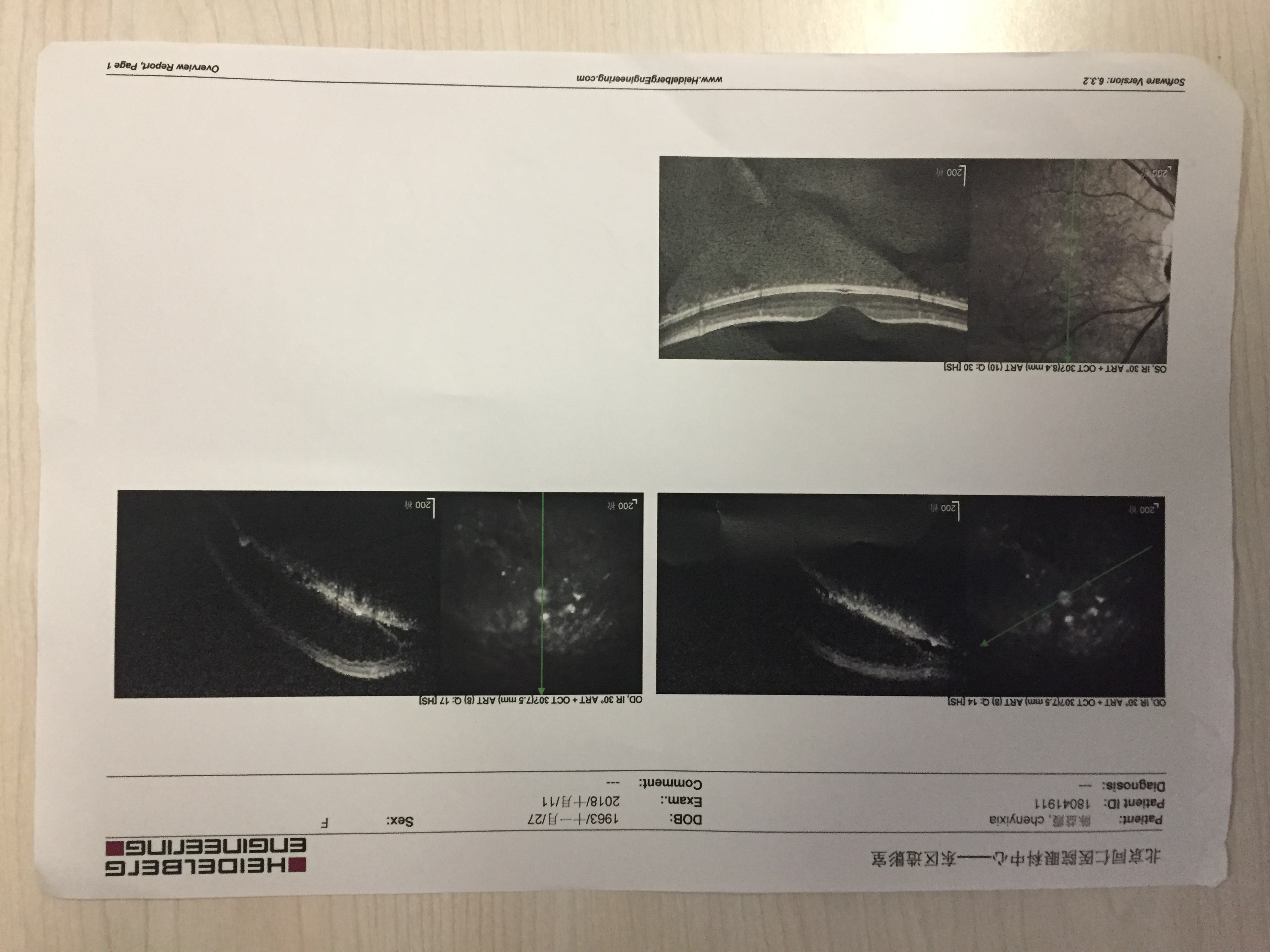

Supplement: Supplementary file 1 — Additional file 1: The raw data of this study. Table 1. The basic information of involved patients. [file 12886_2022_2598_MOESM1_ESM.zip › 3/20181011OCT.JPG]

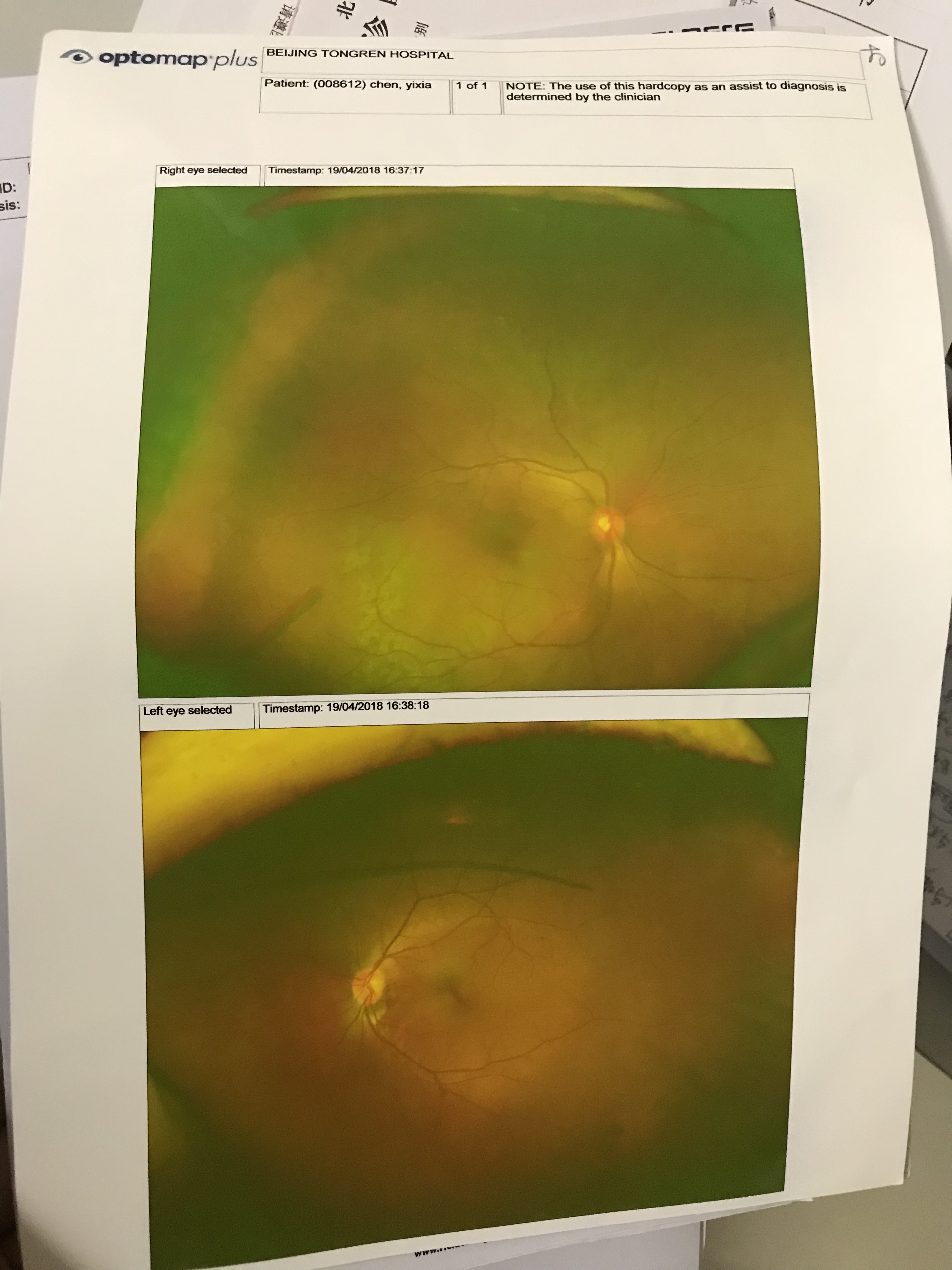

Supplement: Supplementary file 1 — Additional file 1: The raw data of this study. Table 1. The basic information of involved patients. [file 12886_2022_2598_MOESM1_ESM.zip › 3/IMG_4917.JPG]

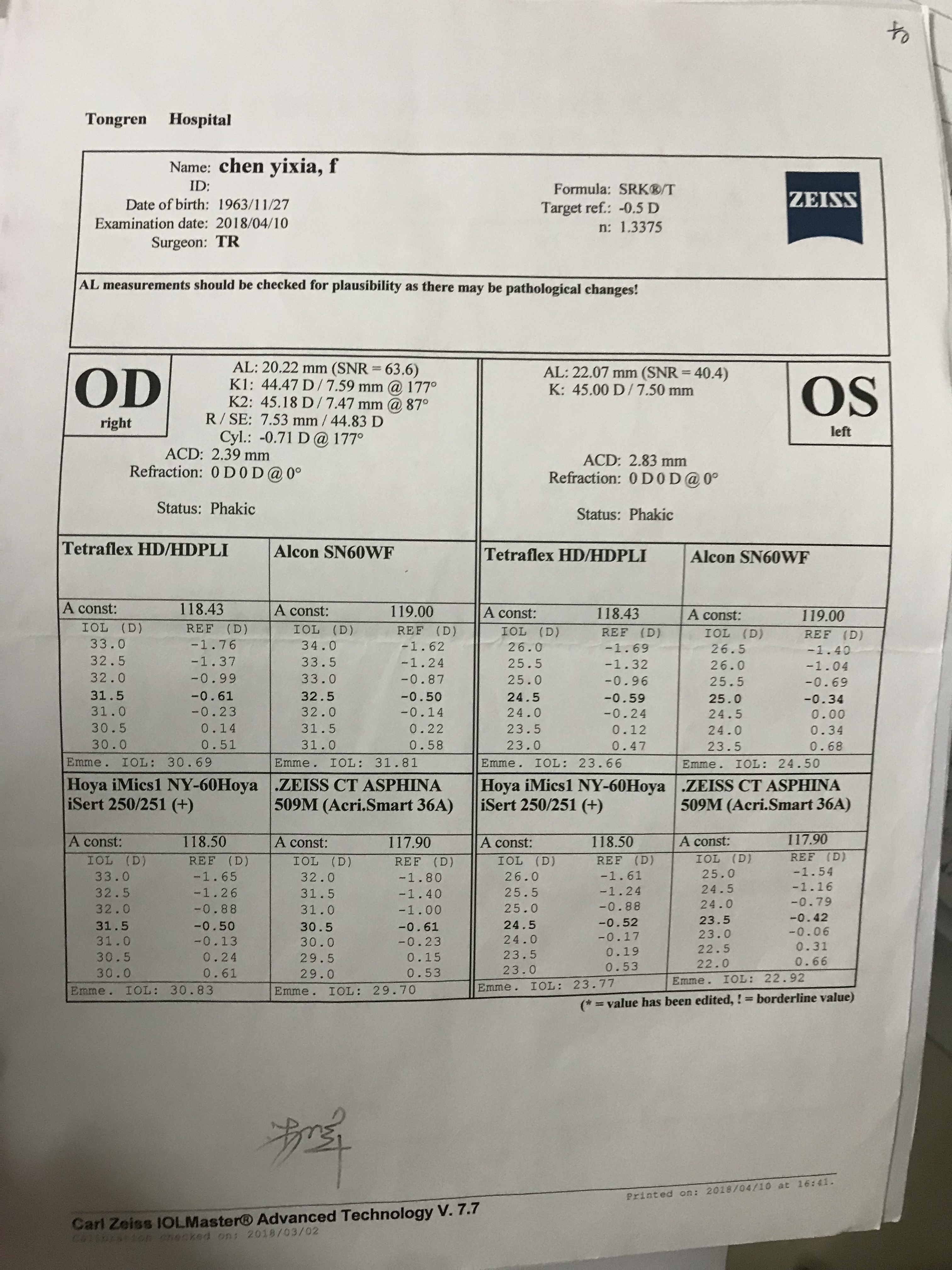

Supplement: Supplementary file 1 — Additional file 1: The raw data of this study. Table 1. The basic information of involved patients. [file 12886_2022_2598_MOESM1_ESM.zip › 3/IMG_4915.JPG]

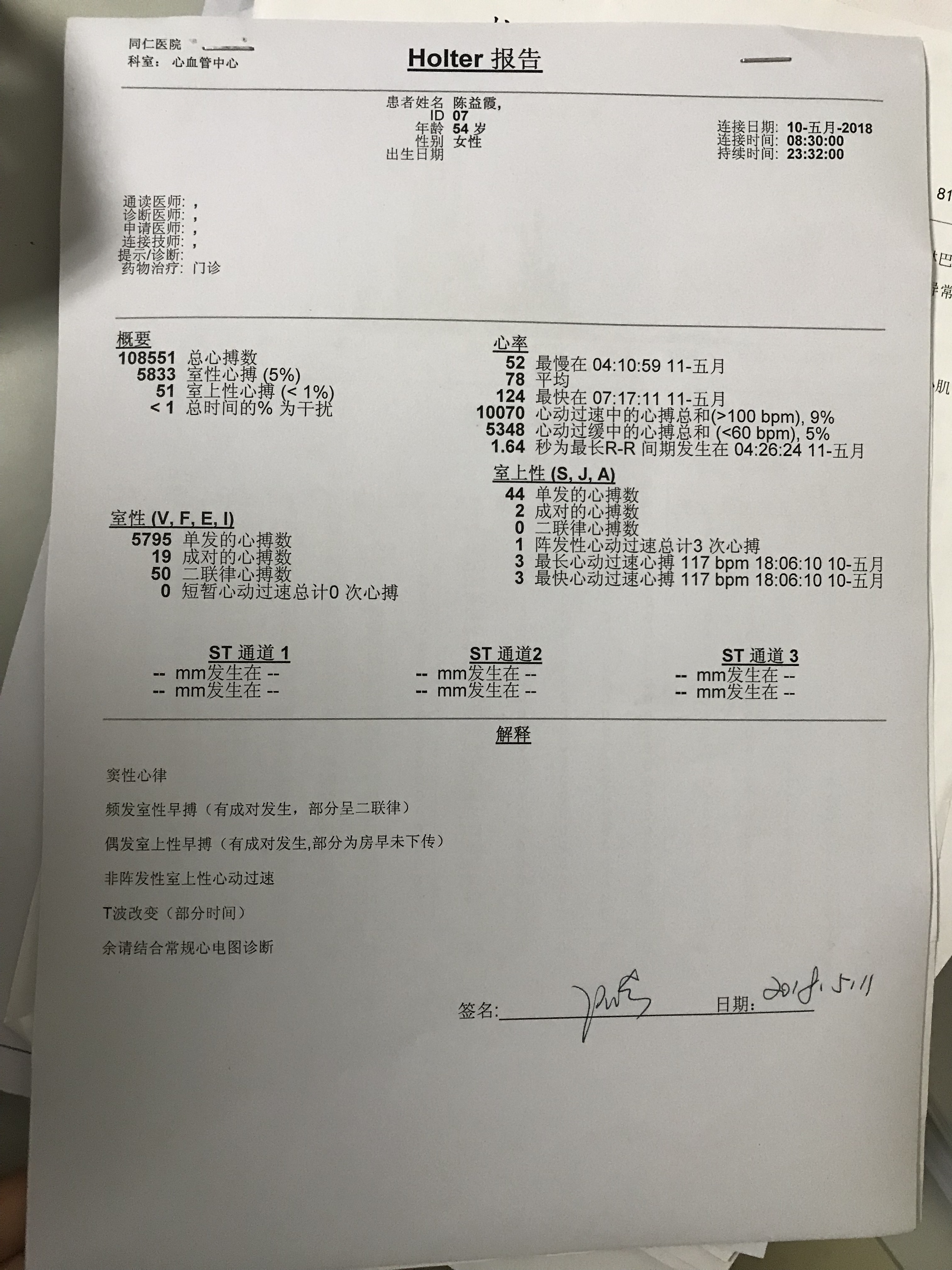

Supplement: Supplementary file 1 — Additional file 1: The raw data of this study. Table 1. The basic information of involved patients. [file 12886_2022_2598_MOESM1_ESM.zip › 3/IMG_4914.JPG]

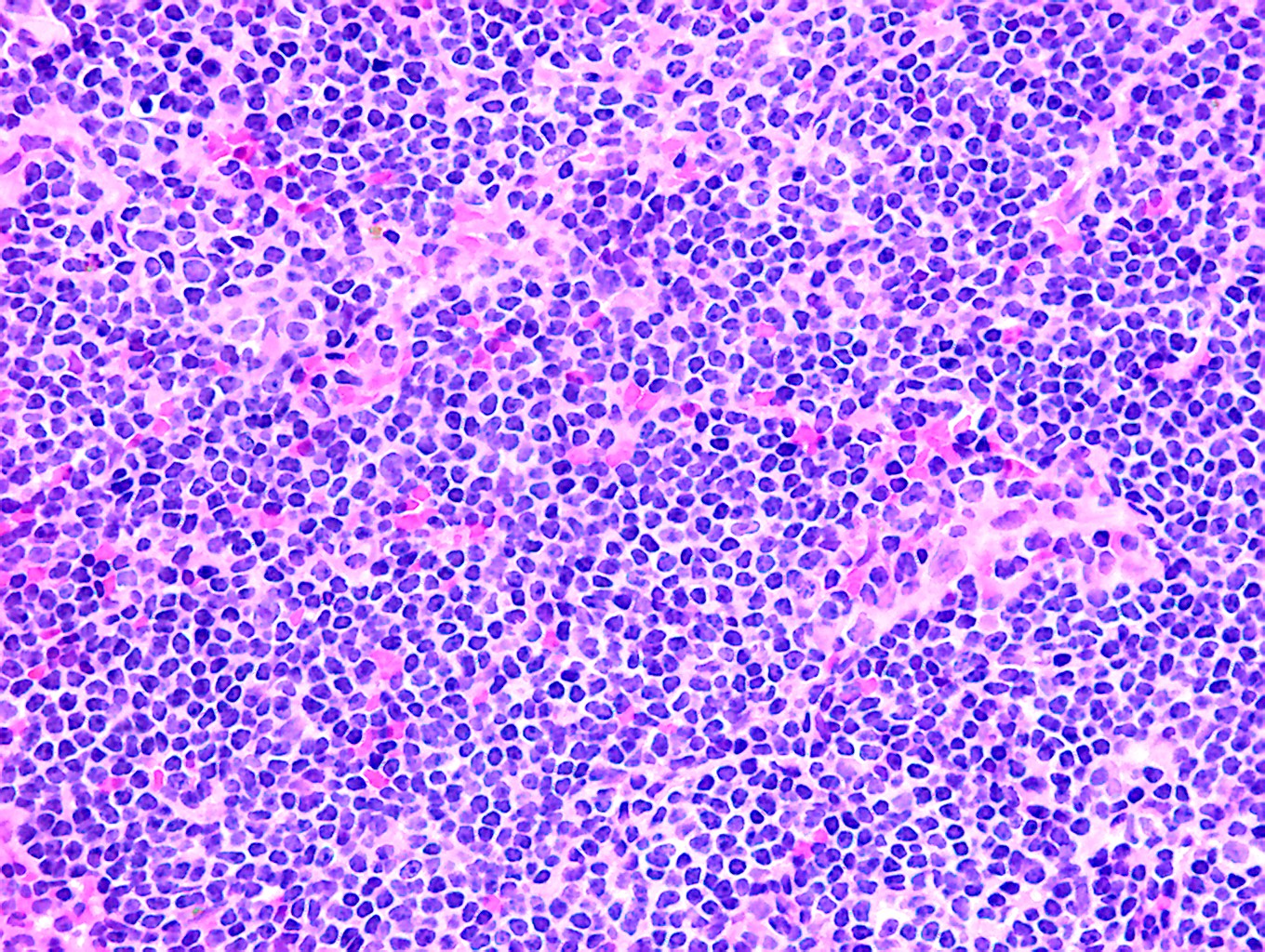

Supplement: Supplementary file 1 — Additional file 1: The raw data of this study. Table 1. The basic information of involved patients. [file 12886_2022_2598_MOESM1_ESM.zip › 3/ΘÖêτ¢èΘ£₧/2-HE-40.jpg]

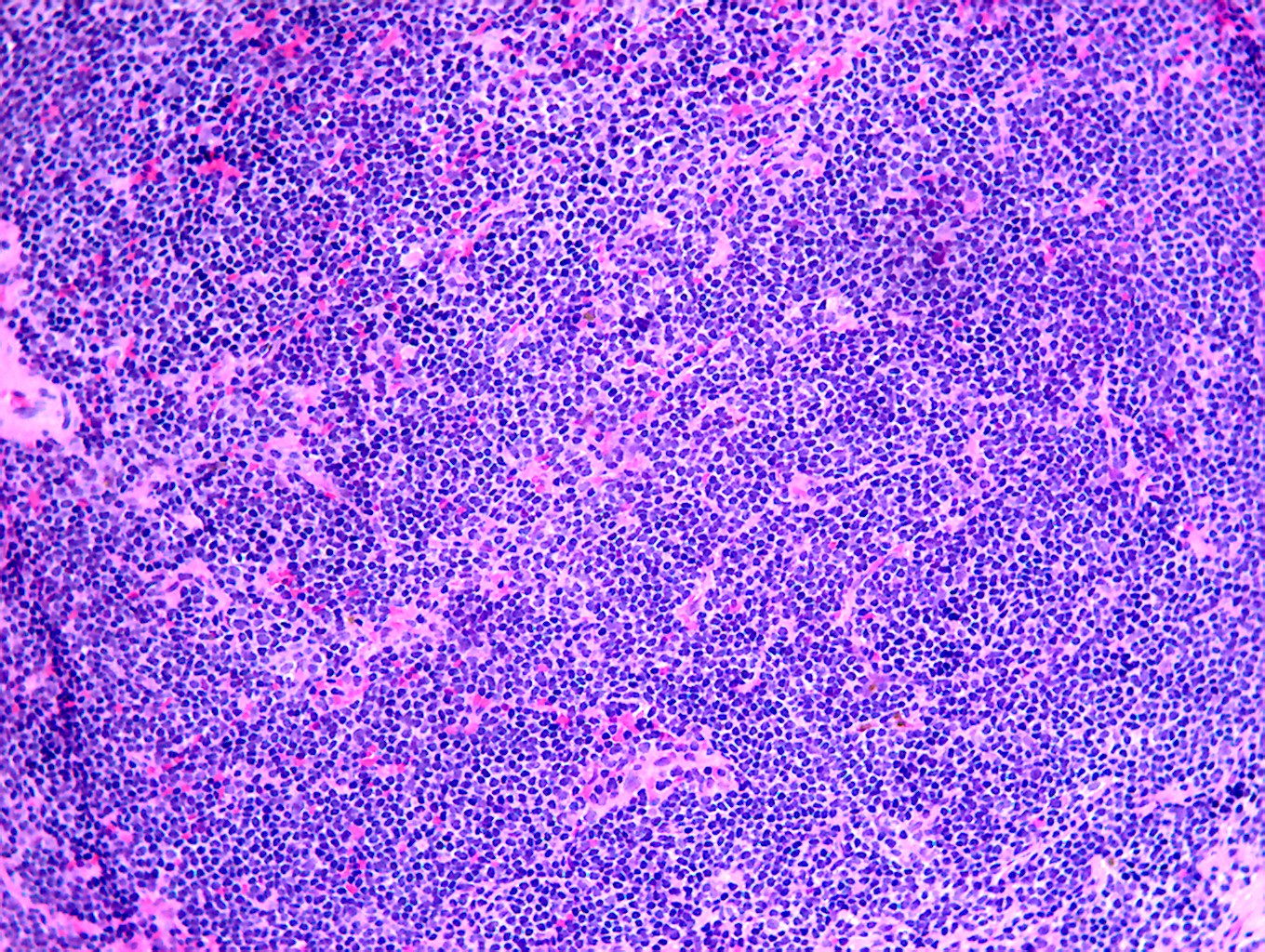

Supplement: Supplementary file 1 — Additional file 1: The raw data of this study. Table 1. The basic information of involved patients. [file 12886_2022_2598_MOESM1_ESM.zip › 3/ΘÖêτ¢èΘ£₧/1-HE-20.jpg]

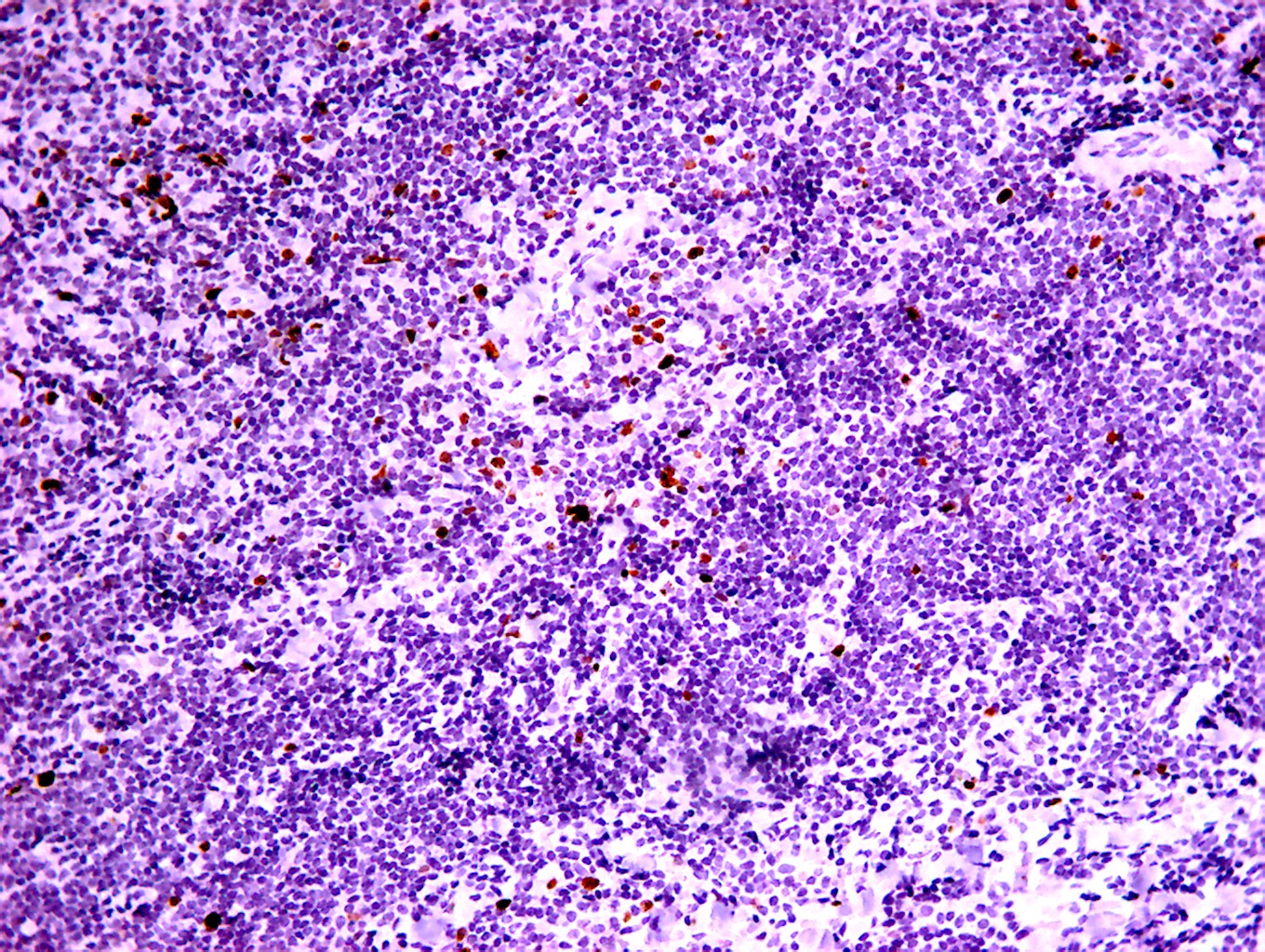

Supplement: Supplementary file 1 — Additional file 1: The raw data of this study. Table 1. The basic information of involved patients. [file 12886_2022_2598_MOESM1_ESM.zip › 3/ΘÖêτ¢èΘ£₧/4-KI-67-IHC-20.jpg]

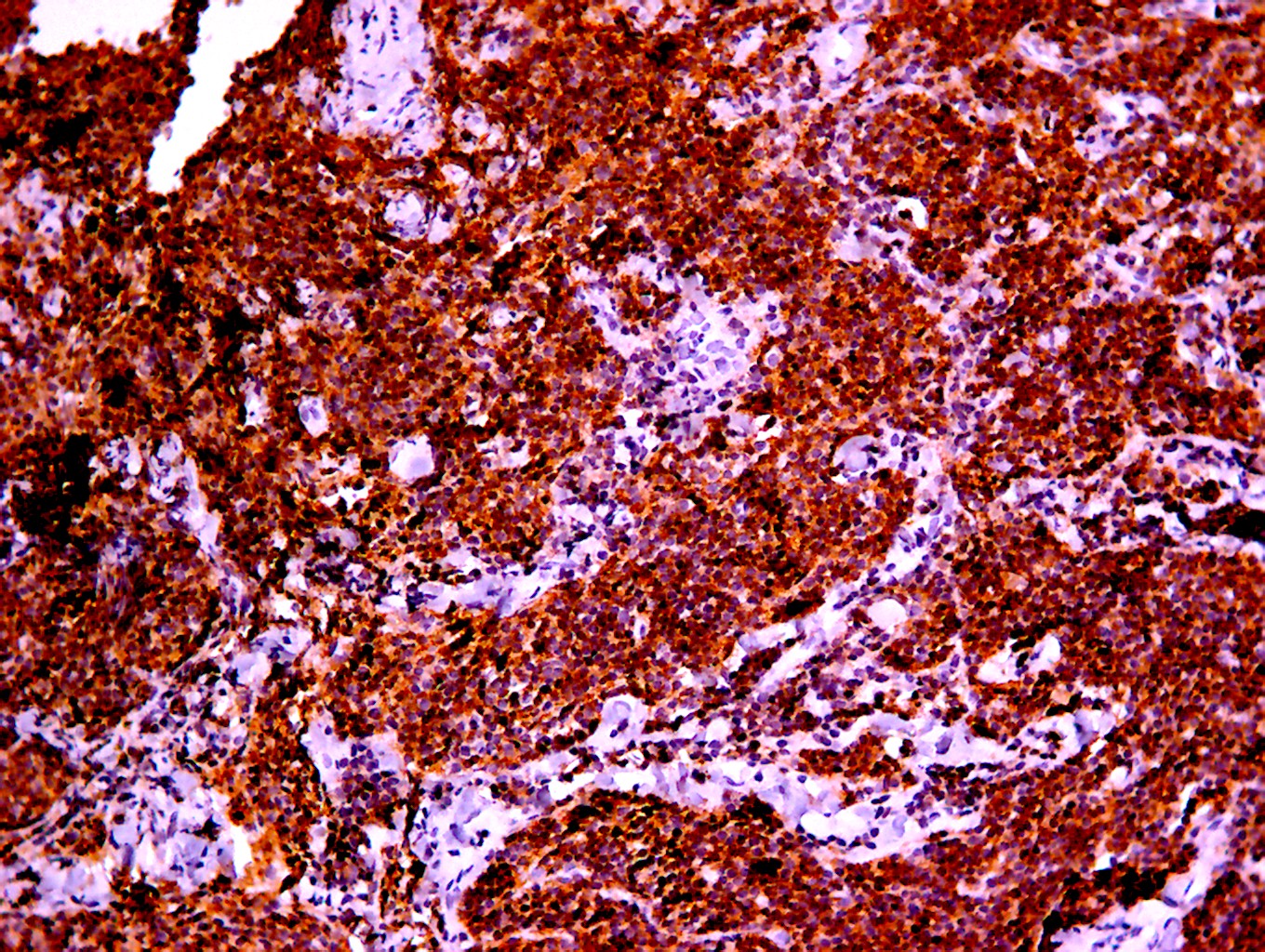

Supplement: Supplementary file 1 — Additional file 1: The raw data of this study. Table 1. The basic information of involved patients. [file 12886_2022_2598_MOESM1_ESM.zip › 3/ΘÖêτ¢èΘ£₧/3-CD20-IHC-20.jpg]

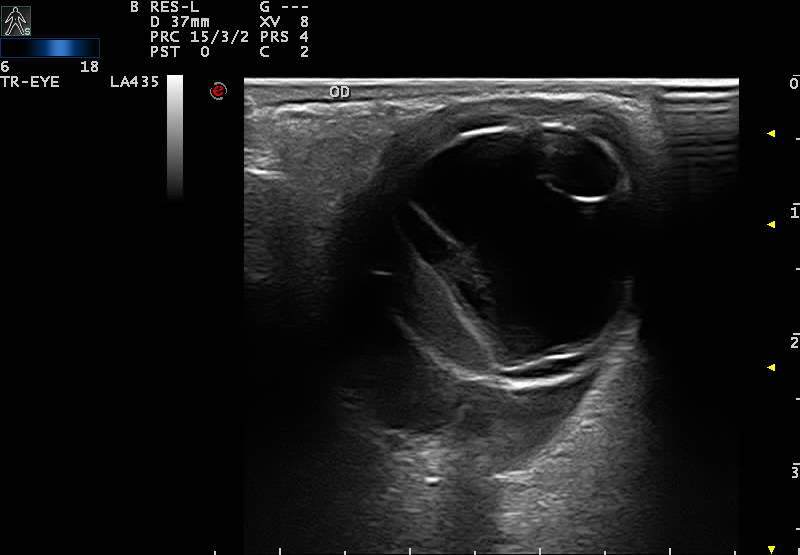

Supplement: Supplementary file 1 — Additional file 1: The raw data of this study. Table 1. The basic information of involved patients. [file 12886_2022_2598_MOESM1_ESM.zip › 3/ΘÖêτ¢èΘ£₧20181011/Image8.jpg]

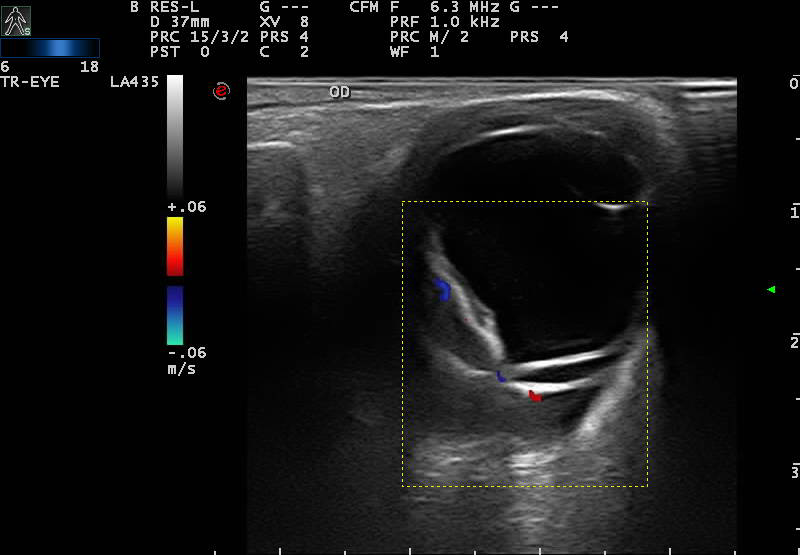

Supplement: Supplementary file 1 — Additional file 1: The raw data of this study. Table 1. The basic information of involved patients. [file 12886_2022_2598_MOESM1_ESM.zip › 3/ΘÖêτ¢èΘ£₧20181011/Image9.jpg]

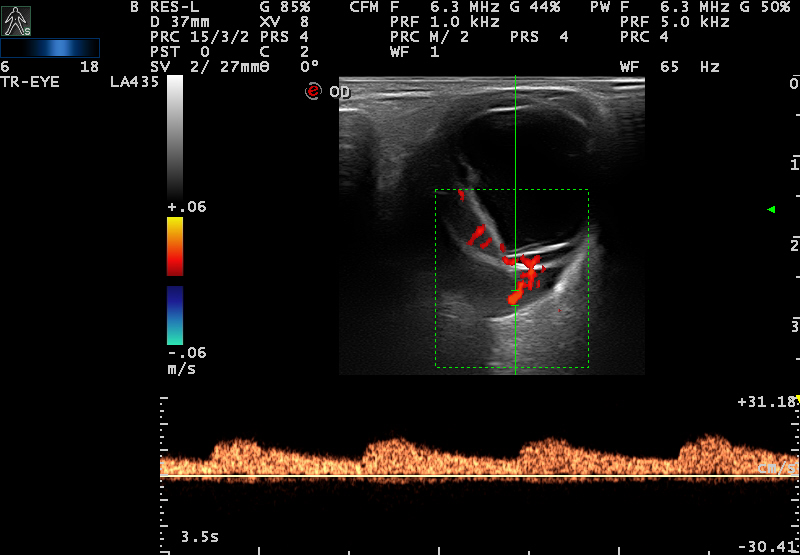

Supplement: Supplementary file 1 — Additional file 1: The raw data of this study. Table 1. The basic information of involved patients. [file 12886_2022_2598_MOESM1_ESM.zip › 3/ΘÖêτ¢èΘ£₧20181011/Image11.jpg]

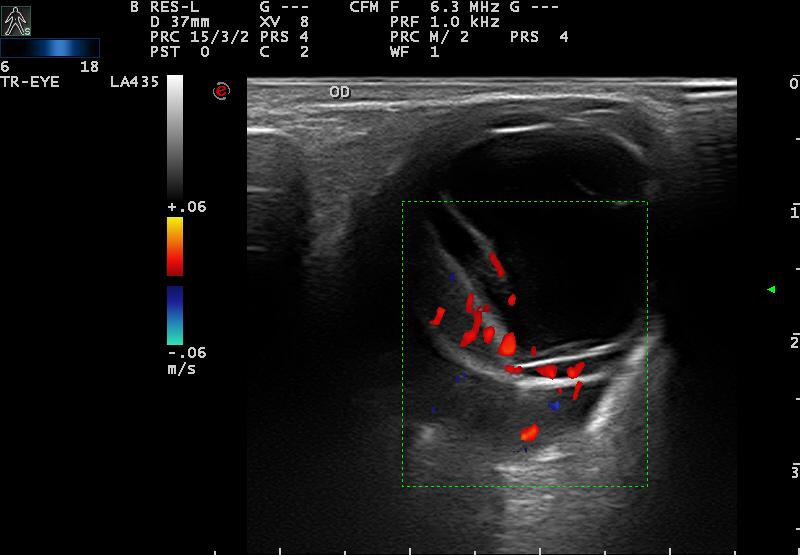

Supplement: Supplementary file 1 — Additional file 1: The raw data of this study. Table 1. The basic information of involved patients. [file 12886_2022_2598_MOESM1_ESM.zip › 3/ΘÖêτ¢èΘ£₧20181011/Image10.jpg]

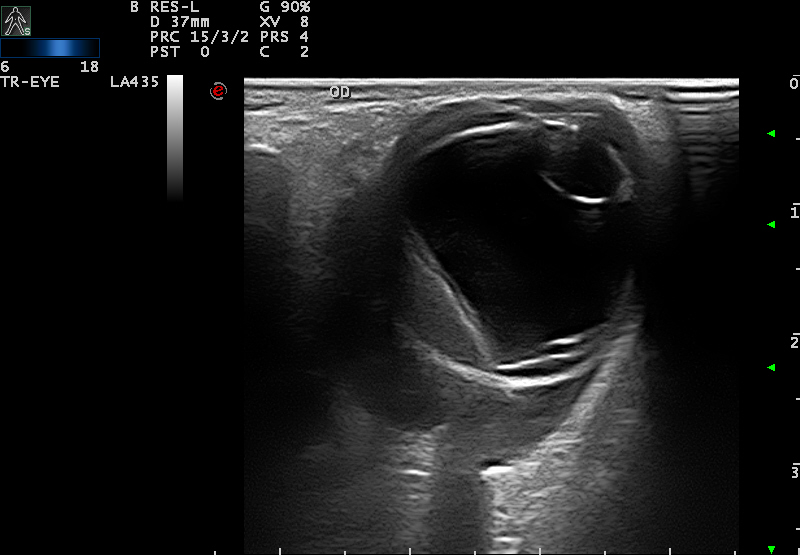

Supplement: Supplementary file 1 — Additional file 1: The raw data of this study. Table 1. The basic information of involved patients. [file 12886_2022_2598_MOESM1_ESM.zip › 3/ΘÖêτ¢èΘ£₧20181011/Image7.jpg]

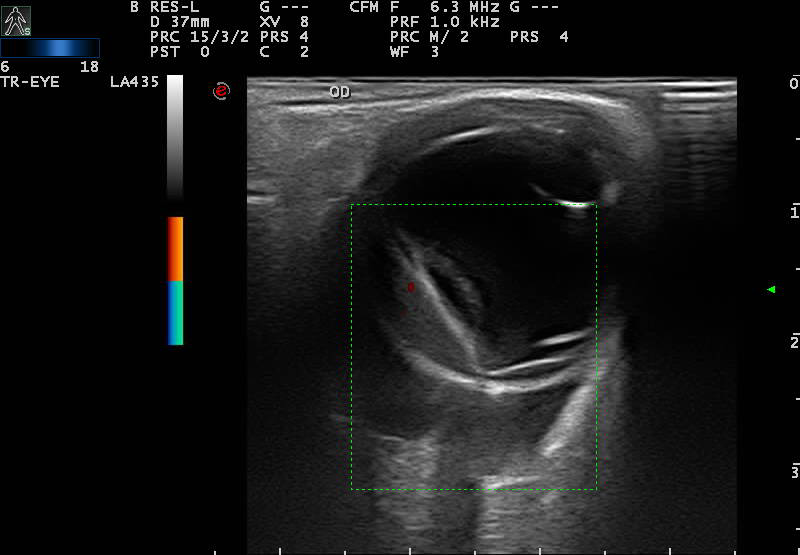

Supplement: Supplementary file 1 — Additional file 1: The raw data of this study. Table 1. The basic information of involved patients. [file 12886_2022_2598_MOESM1_ESM.zip › 3/ΘÖêτ¢èΘ£₧20181011/Image6.jpg]

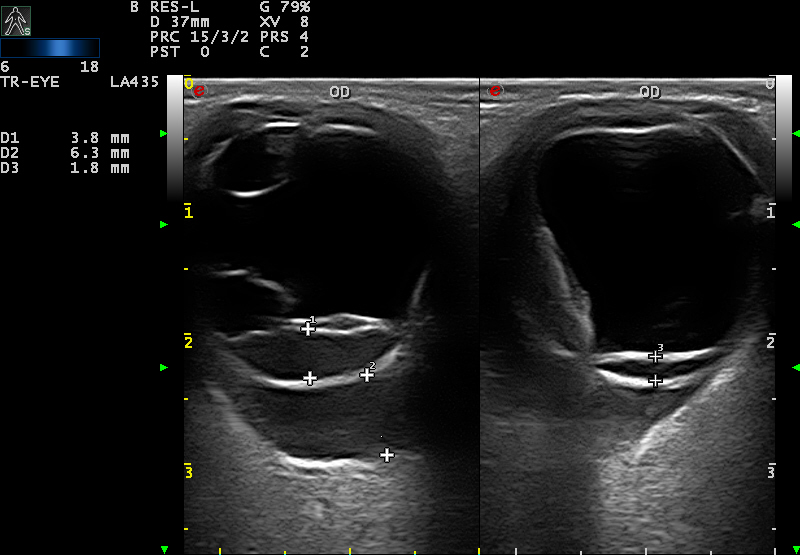

Supplement: Supplementary file 1 — Additional file 1: The raw data of this study. Table 1. The basic information of involved patients. [file 12886_2022_2598_MOESM1_ESM.zip › 3/ΘÖêτ¢èΘ£₧20181011/Image4.jpg]

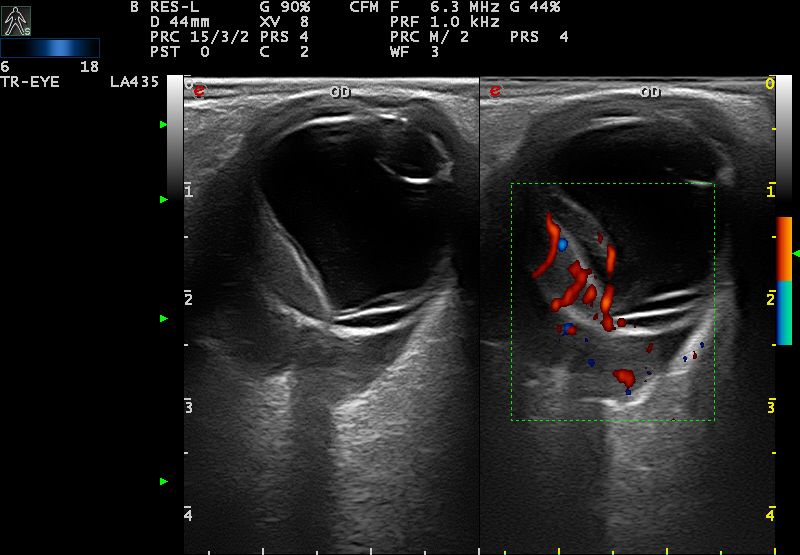

Supplement: Supplementary file 1 — Additional file 1: The raw data of this study. Table 1. The basic information of involved patients. [file 12886_2022_2598_MOESM1_ESM.zip › 3/ΘÖêτ¢èΘ£₧20181011/Image5.jpg]

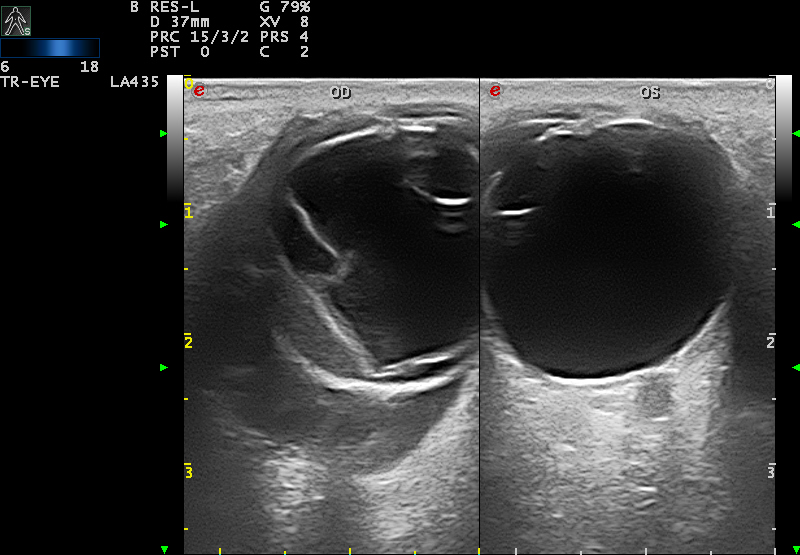

Supplement: Supplementary file 1 — Additional file 1: The raw data of this study. Table 1. The basic information of involved patients. [file 12886_2022_2598_MOESM1_ESM.zip › 3/ΘÖêτ¢èΘ£₧20181011/Image1.jpg]

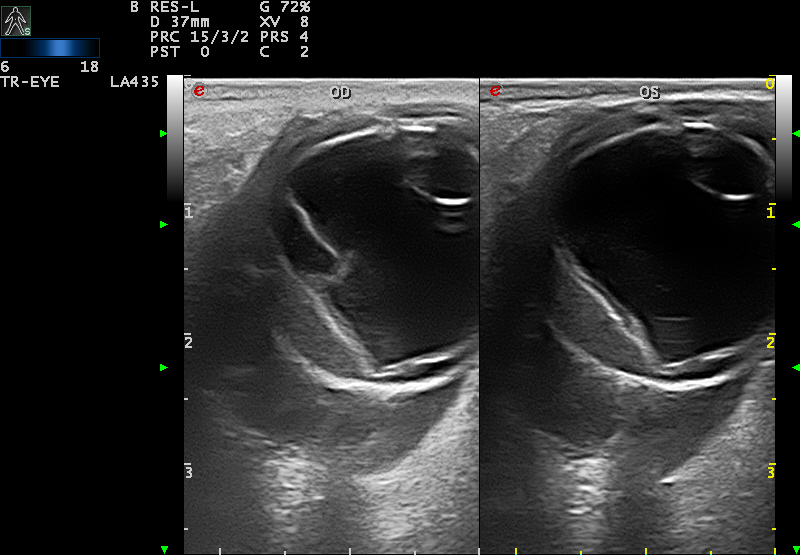

Supplement: Supplementary file 1 — Additional file 1: The raw data of this study. Table 1. The basic information of involved patients. [file 12886_2022_2598_MOESM1_ESM.zip › 3/ΘÖêτ¢èΘ£₧20181011/Image2.jpg]

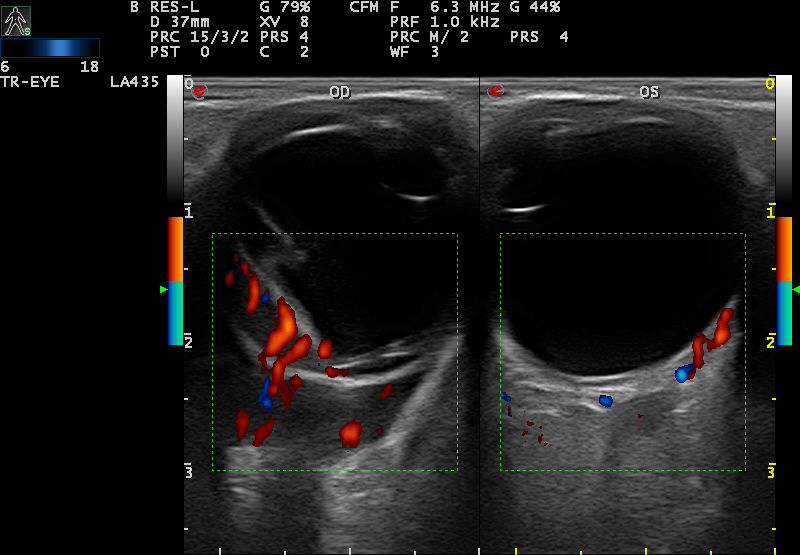

Supplement: Supplementary file 1 — Additional file 1: The raw data of this study. Table 1. The basic information of involved patients. [file 12886_2022_2598_MOESM1_ESM.zip › 3/ΘÖêτ¢èΘ£₧20181011/Image3.jpg]

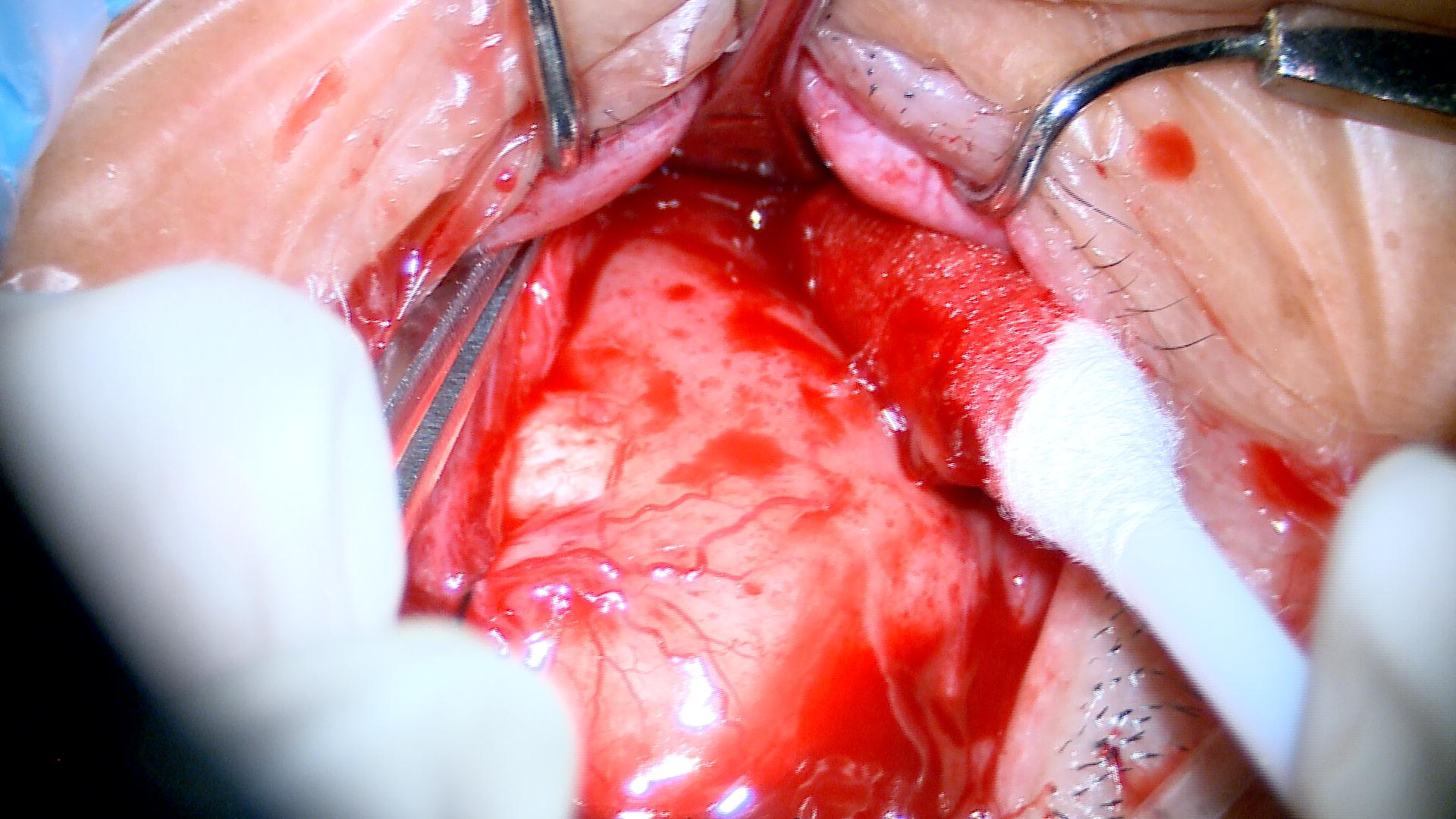

Supplement: Supplementary file 1 — Additional file 1: The raw data of this study. Table 1. The basic information of involved patients. [file 12886_2022_2598_MOESM1_ESM.zip › 3/μ£»Σ╕¡σñoΣ╜ôσâÅ/0123180313372.jpg]

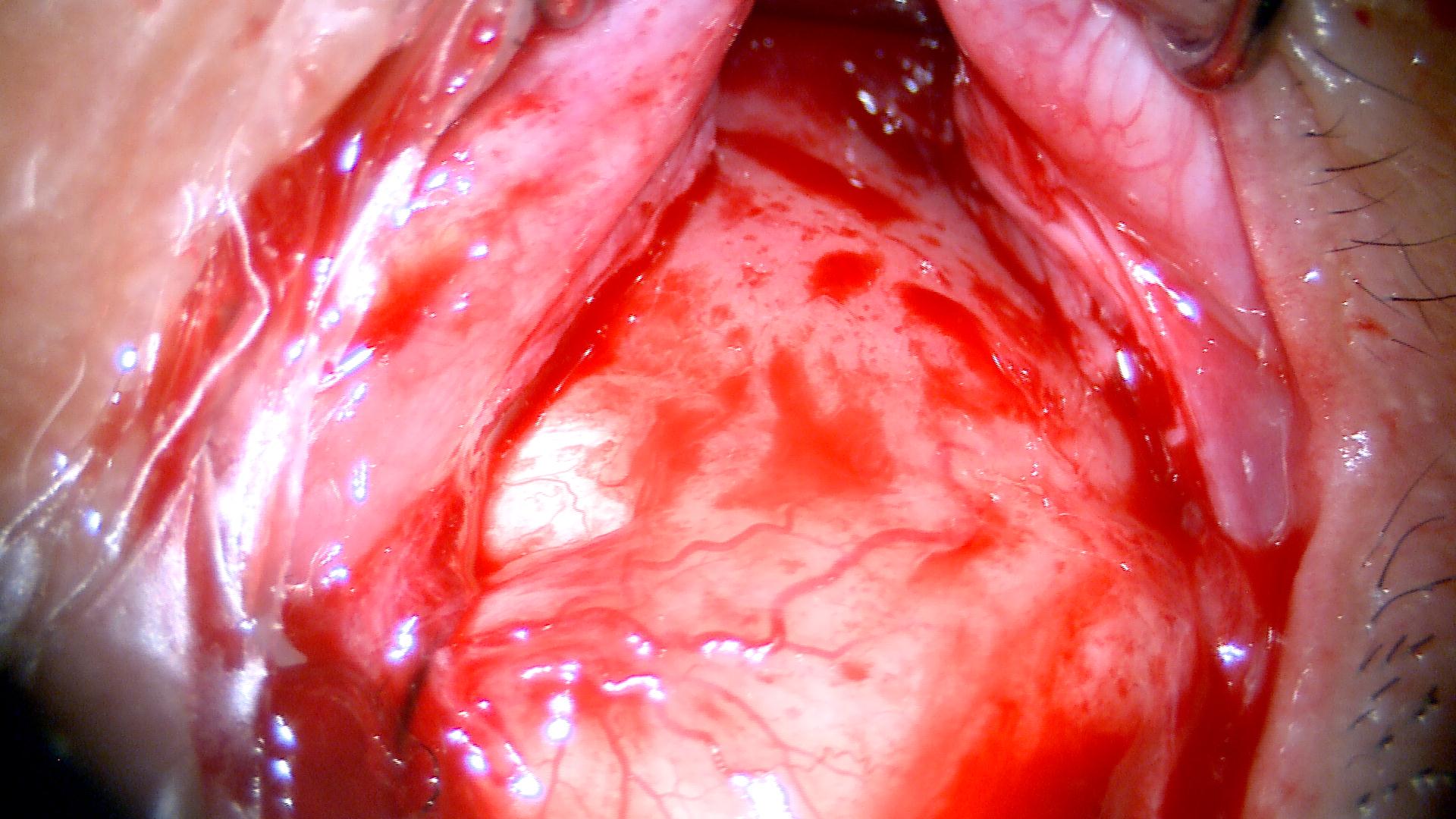

Supplement: Supplementary file 1 — Additional file 1: The raw data of this study. Table 1. The basic information of involved patients. [file 12886_2022_2598_MOESM1_ESM.zip › 3/μ£»Σ╕¡σñoΣ╜ôσâÅ/0123180328325.jpg]

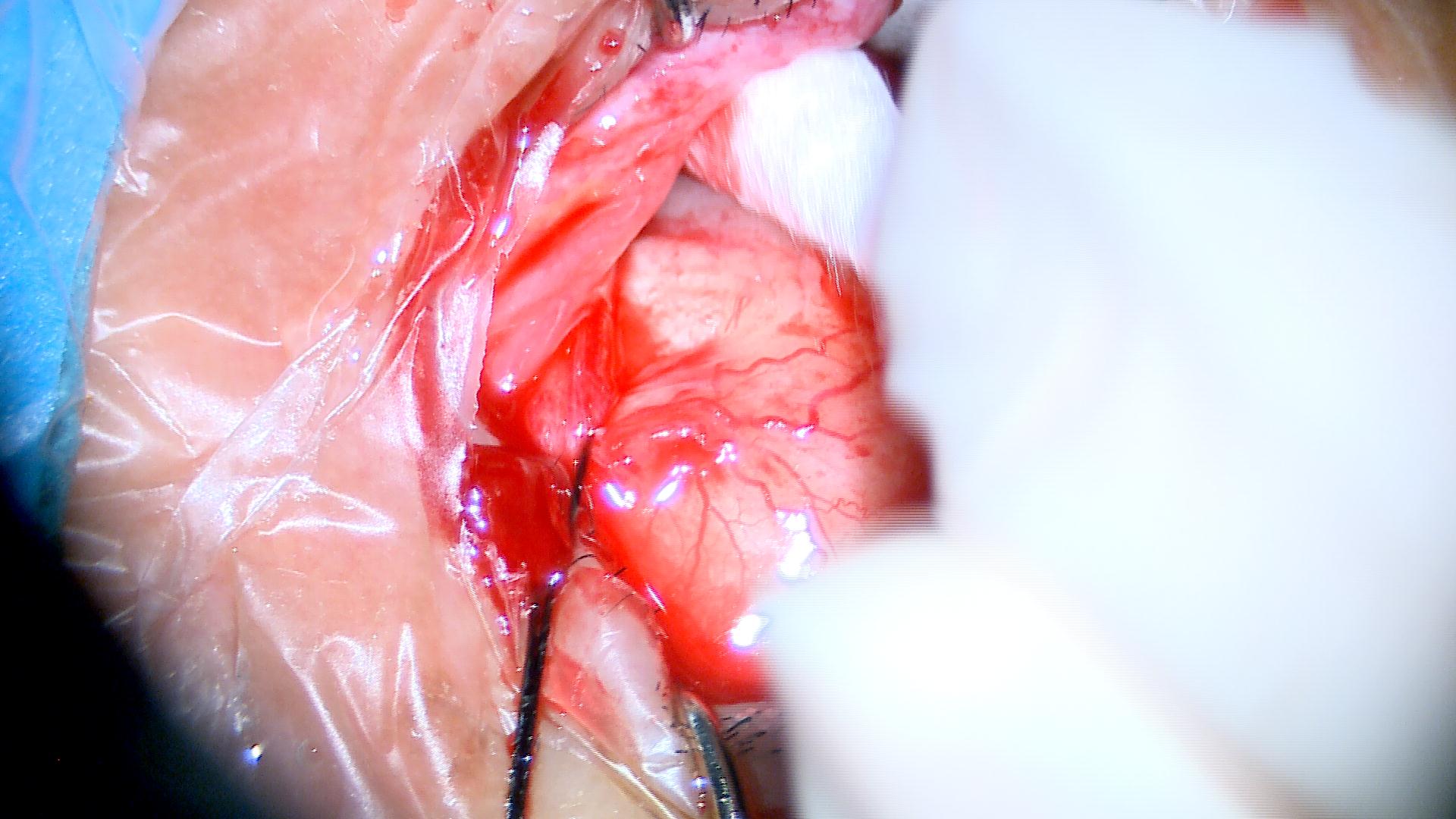

Supplement: Supplementary file 1 — Additional file 1: The raw data of this study. Table 1. The basic information of involved patients. [file 12886_2022_2598_MOESM1_ESM.zip › 3/μ£»Σ╕¡σñoΣ╜ôσâÅ/0123180229629.jpg]

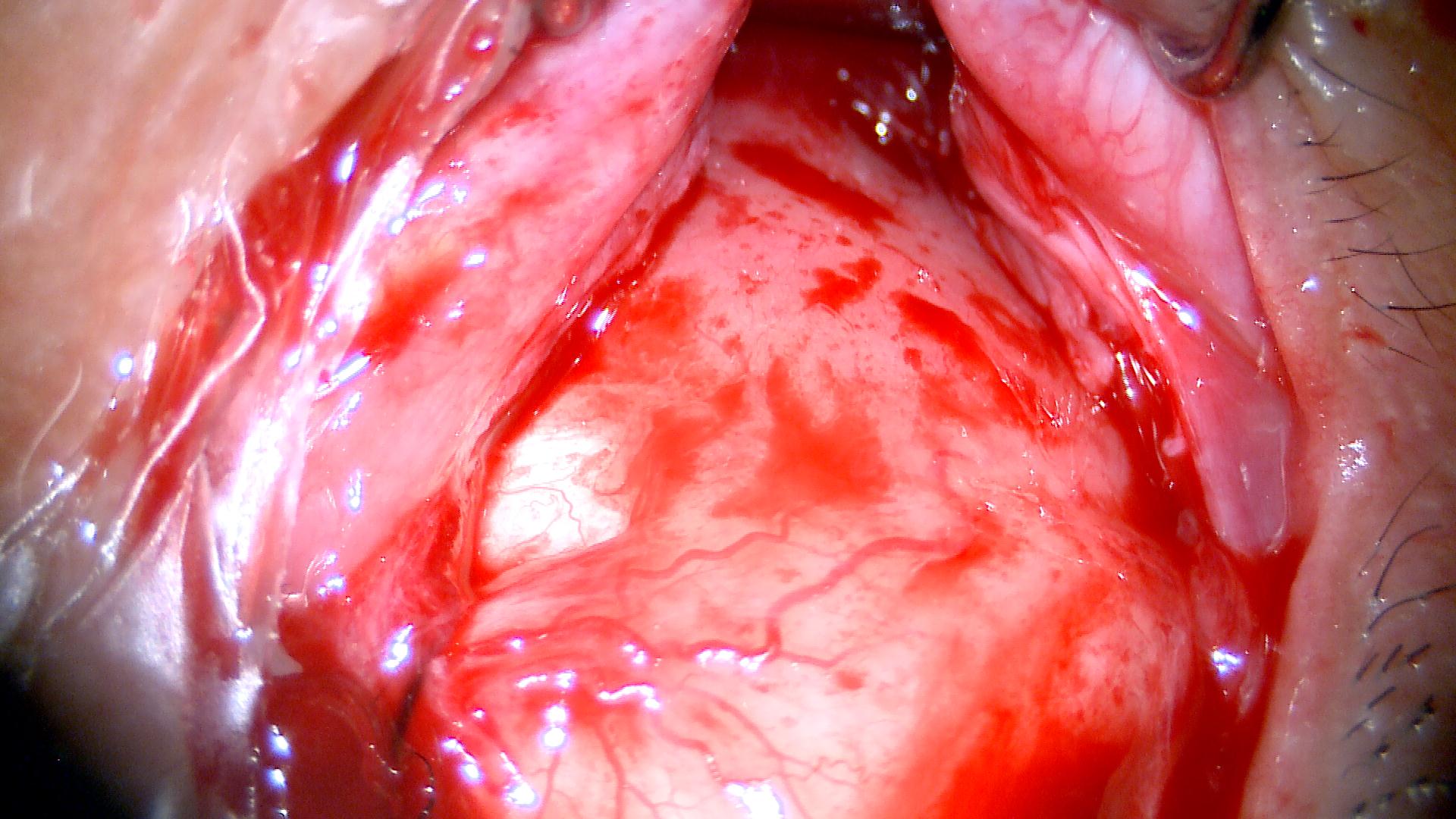

Supplement: Supplementary file 1 — Additional file 1: The raw data of this study. Table 1. The basic information of involved patients. [file 12886_2022_2598_MOESM1_ESM.zip › 3/μ£»Σ╕¡σñoΣ╜ôσâÅ/0123180327604.jpg]

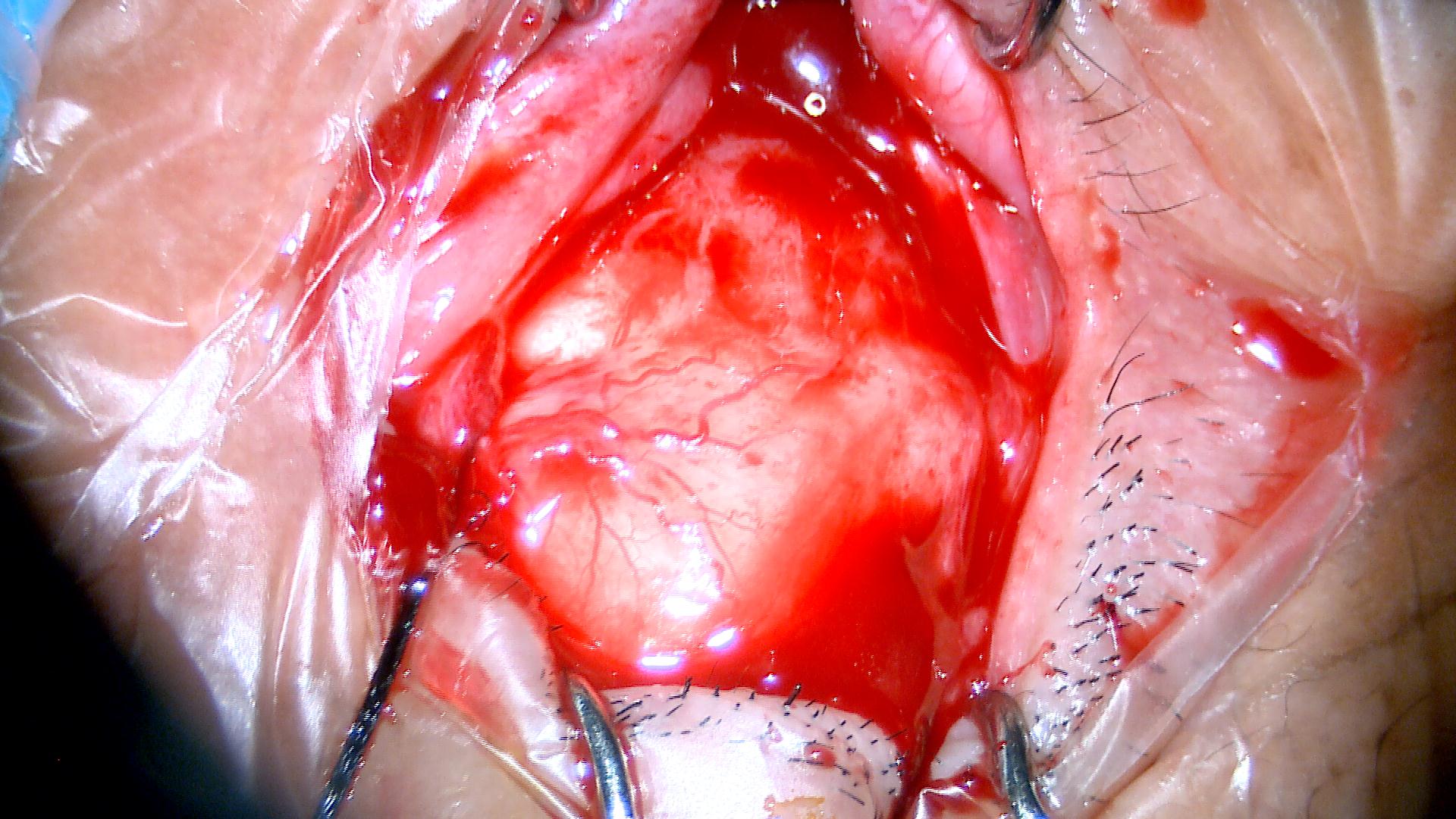

Supplement: Supplementary file 1 — Additional file 1: The raw data of this study. Table 1. The basic information of involved patients. [file 12886_2022_2598_MOESM1_ESM.zip › 3/μ£»Σ╕¡σñoΣ╜ôσâÅ/0123180250933.jpg]

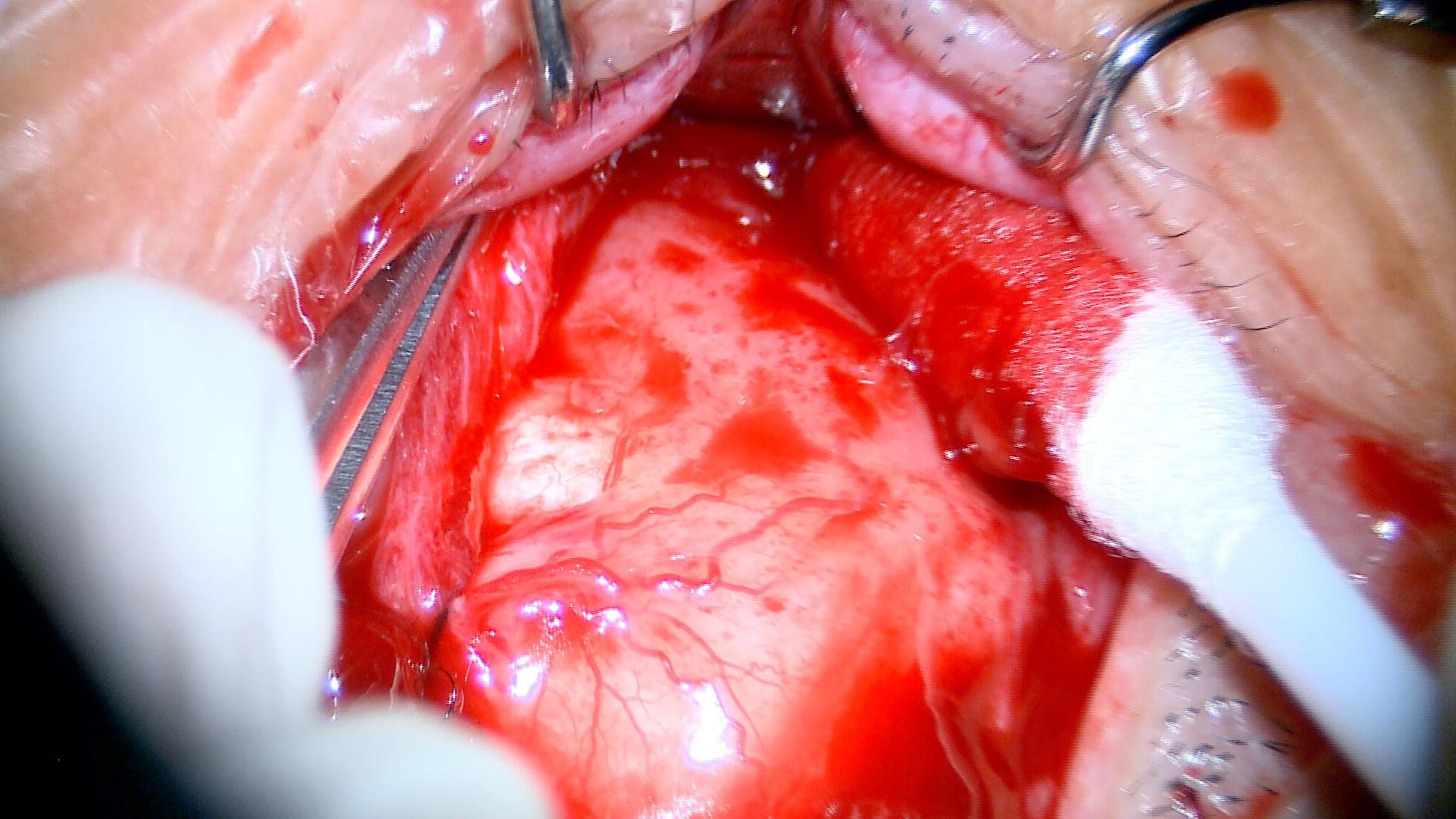

Supplement: Supplementary file 1 — Additional file 1: The raw data of this study. Table 1. The basic information of involved patients. [file 12886_2022_2598_MOESM1_ESM.zip › 3/μ£»Σ╕¡σñoΣ╜ôσâÅ/0123180314988.jpg]

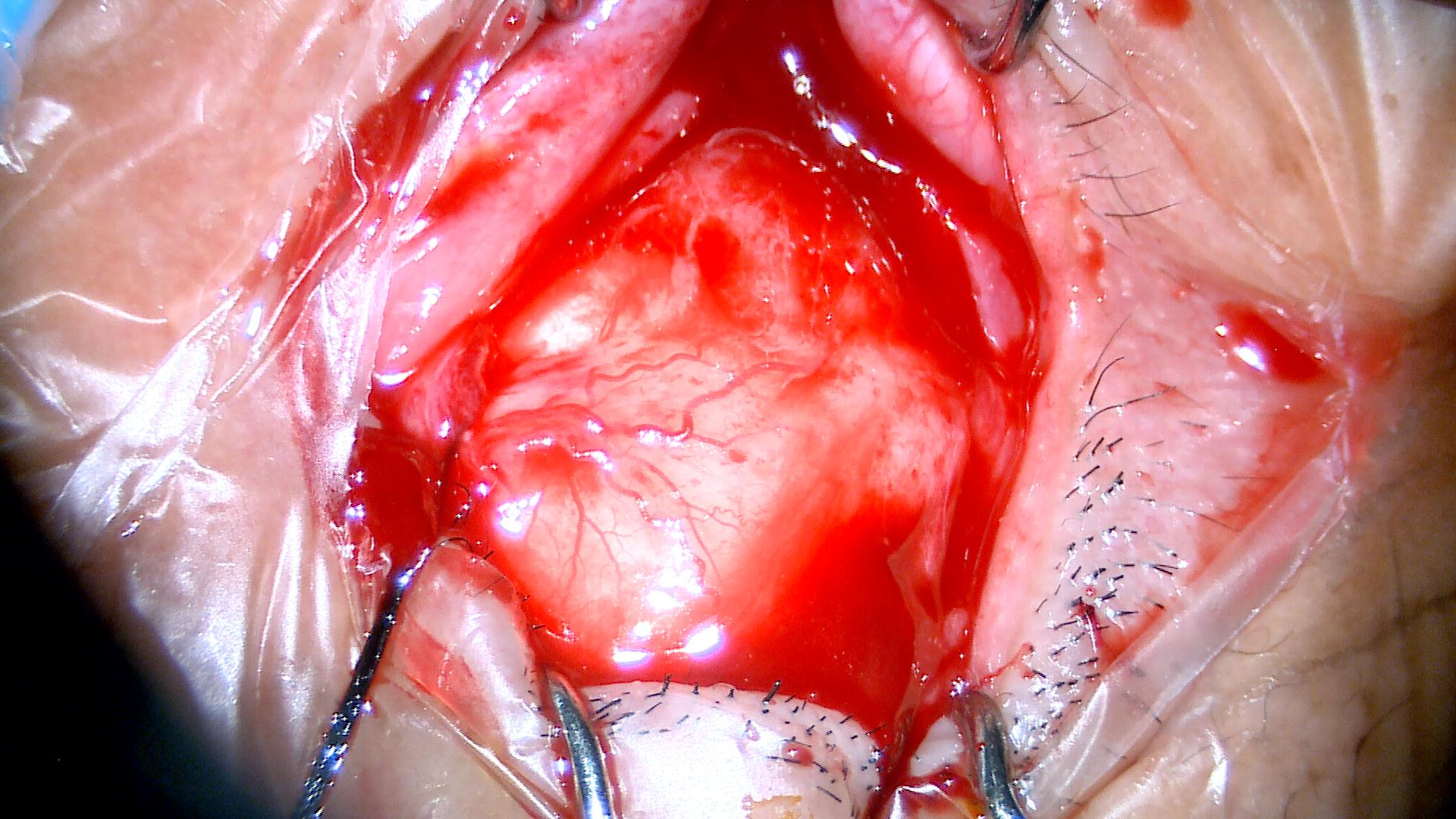

Supplement: Supplementary file 1 — Additional file 1: The raw data of this study. Table 1. The basic information of involved patients. [file 12886_2022_2598_MOESM1_ESM.zip › 3/μ£»Σ╕¡σñoΣ╜ôσâÅ/0123180253397.jpg]

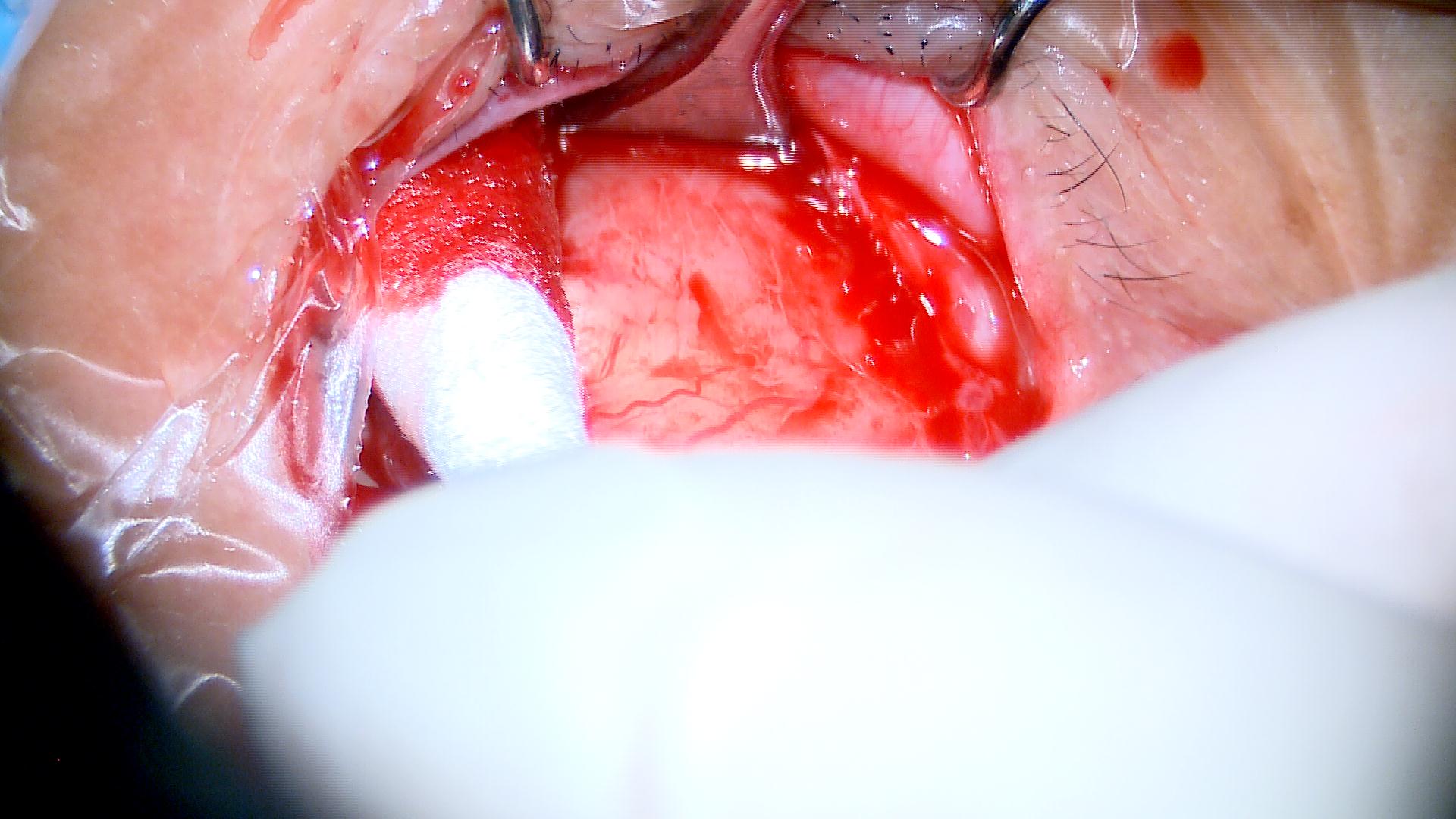

Supplement: Supplementary file 1 — Additional file 1: The raw data of this study. Table 1. The basic information of involved patients. [file 12886_2022_2598_MOESM1_ESM.zip › 3/μ£»Σ╕¡σñoΣ╜ôσâÅ/0123180231880.jpg]

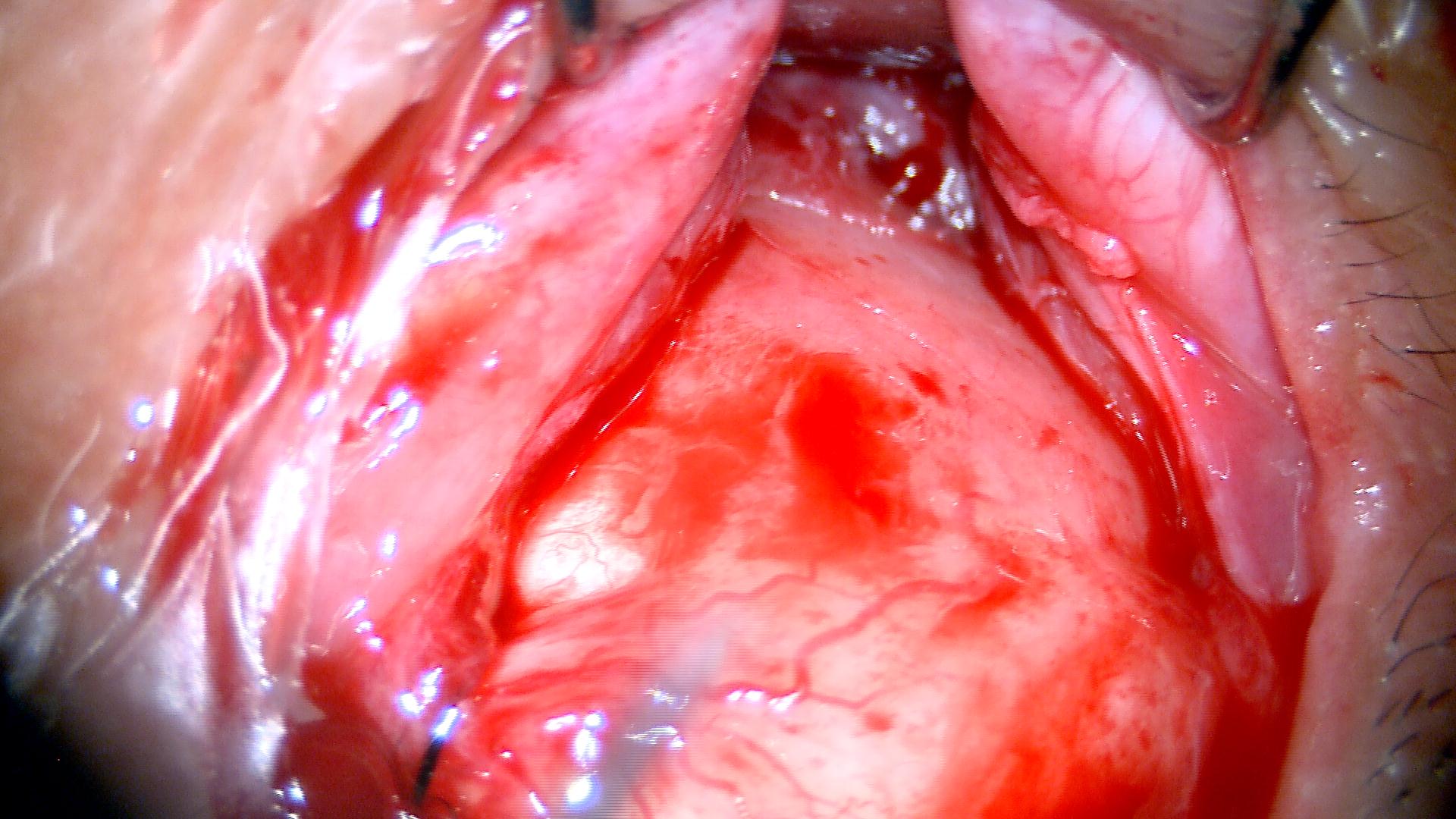

Supplement: Supplementary file 1 — Additional file 1: The raw data of this study. Table 1. The basic information of involved patients. [file 12886_2022_2598_MOESM1_ESM.zip › 3/μ£»Σ╕¡σñoΣ╜ôσâÅ/0123180342475.jpg]

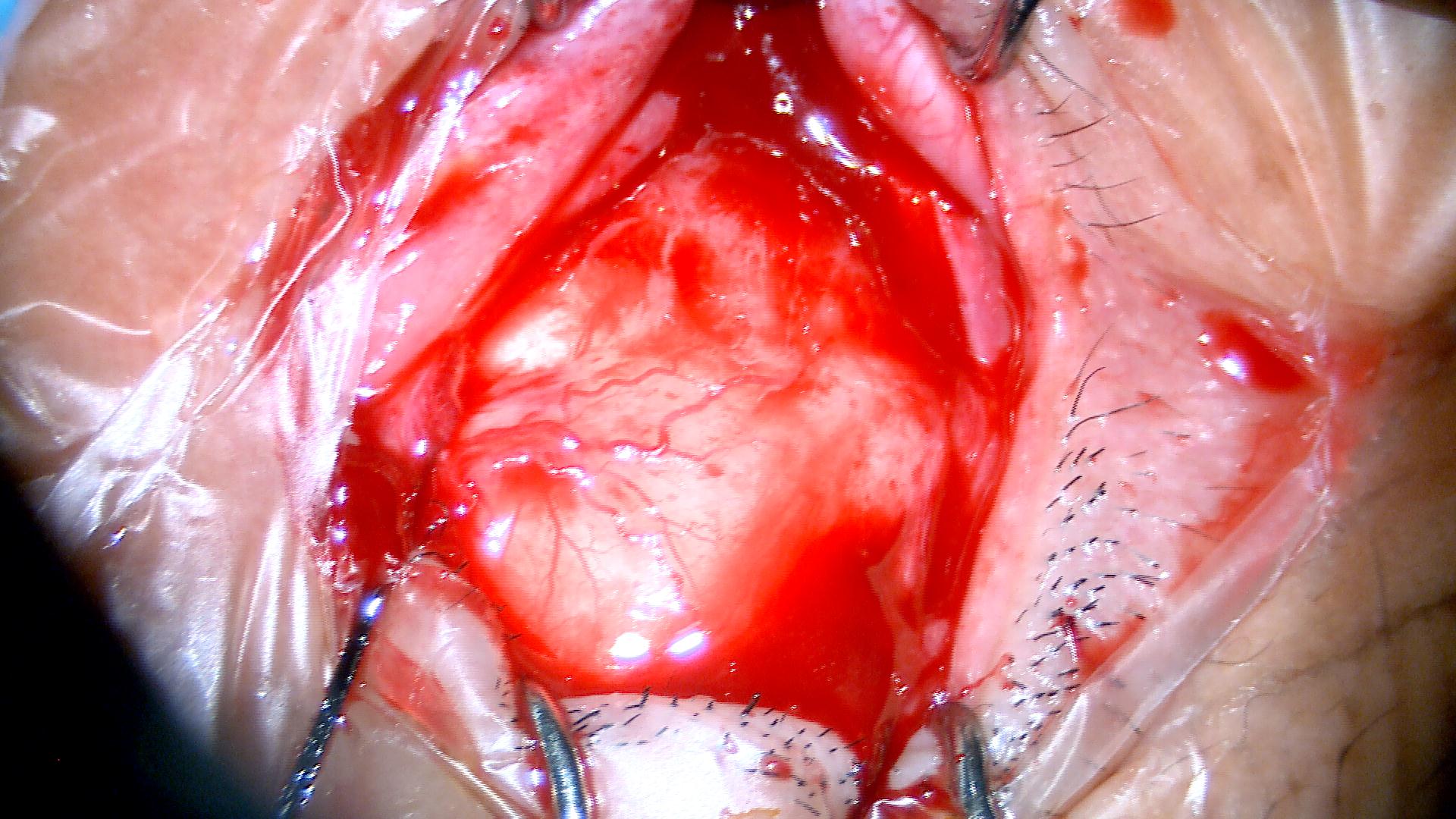

Supplement: Supplementary file 1 — Additional file 1: The raw data of this study. Table 1. The basic information of involved patients. [file 12886_2022_2598_MOESM1_ESM.zip › 3/μ£»Σ╕¡σñoΣ╜ôσâÅ/0123180252075.jpg]

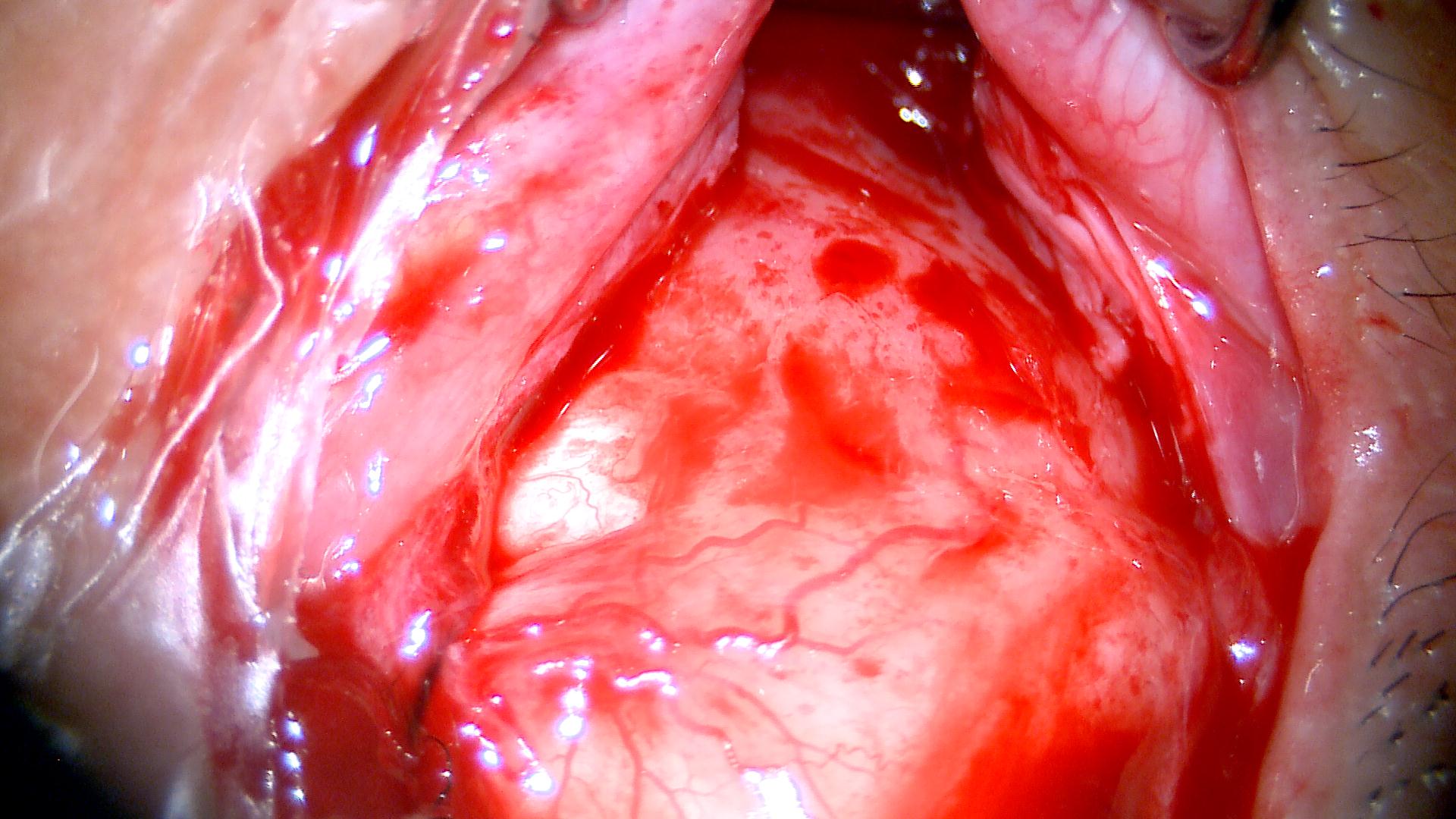

Supplement: Supplementary file 1 — Additional file 1: The raw data of this study. Table 1. The basic information of involved patients. [file 12886_2022_2598_MOESM1_ESM.zip › 3/μ£»Σ╕¡σñoΣ╜ôσâÅ/0123180330052.jpg]
